# Mechanism, reactivity, and regioselectivity in rhodium-catalyzed asymmetric ring-opening reactions of oxabicyclic alkenes: a DFT Investigation

**DOI:** 10.1038/srep40491

**Published:** 2017-01-11

**Authors:** Zheng-Hang Qi, Yi Zhang, Yun Gao, Ye Zhang, Xing-Wang Wang, Yong Wang

**Affiliations:** 1 College of Chemistry, Chemical Engineering and Materials Science, Soochow University, Suzhou 215123, People’s Republic of China

## Abstract

The origin of the enantio- and regioselectivity of ring-opening reaction of oxabicyclic alkenes catalyzed by rhodium/Josiphos has been examined using M06-2X density functional theory(DFT). DFT calculations predict a 98% ee for the enantioselectivity and only the 1,2-*trans* product as one regio- and diastereomer, in excellent agreement with experimental results. The solvent tetrahydrofuran(THF) plays a key role in assisting nucleophilic attack. Orbital composition analysis of the LUMO and the NPA atomic charge calculations were conducted to probe the origins of the regioselectivity. The orbital composition analysis reveals two potential electrophilic sites of the Rh–π-allyl intermediate M3 and the NPA atomic charges demonstrate that Cα carries more positive charges than Cγ, which suggests that Cα is the electrophilic site.

Ligands promoted oganometallic catalysis has consistently attracted considerable interest for both academia and industry, due to its broad applications in synthetic transformations^1^,^2^. Among enormous transition metal catalysts used in organic synthesis, catalytic active rhodium has been of great significance in this regard. Since the discovery of the Wilkinson complex [RhCl(PPh_3_)_3_] which was proved to be the harbinger of the development of modern organorhodium chemistry, the new field of homogeneous catalysis was opened. Particularly in the preparation of enantiomerically enriched compounds, numerous rhodium-catalyzed reactions have been developed, such as asymmetric hydrogenation^3^, isomerization of olefins^4^, hydroacylation^5^, cycloaddition^6^ and C–H insertion^7^, etc. Over the past decade, several metal-catalyzed asymmetric ring cleaving reactions have been developed that generate ring-opened products in high yield and enantiomeric excess. Generally, some transition metals, such as rhodium, copper as well as palladium, have been commonly employed for this type nucleophilic ring opening reactions^8^,^9^,^10^,^11^. Lautens reported in 2000 that rhodium/Josiphos catalyzed the asymmetric ring-opening (ARO) reaction of oxabicyclic alkenes involving heteroatom nucleophiles^8^. In this reaction, very high regio- and diastereoselectivity, and excellent enantioselectivity were observed (Fig. 1). This pioneering work has been regarded as a landmark in rapid access to chiral building blocks, as the resulting allylic compounds are important structural motifs in medicinal chemistry^12^. Since then, various nucleophiles were applied to this catalytic system including oxygen nucleophiles^13^, nitrogen nucleophiles^14^, sulfur nucleophiles^15^, carbon nucleophiles^16^ and halogen atoms^17^.

As depicted in Fig. 2, the commonly accepted mechanism for the ARO reaction of oxabicyclic alkenes is given by Lautens *et al*.^18^. Dimeric metal salt [Rh(cod)Cl]_2_ is cleaved by solvation, substrate binding, or reaction with the nucleophile to give monomeric complex **I**. Then, the π-allyl or enyl rhodium alkoxide complex **II** or **III** was generated by oxidative insertion with retention into a bridgehead C–O bond, which is regarded as the enantiodiscriminating step in the catalytic cycle. Subsequently, **II** or **III** could be protonated by the alcohol prenucleophile. After **II** or **III**, cationic rhodium complex **IV** and an alkoxide or phenoxide was proposed. Finally, the product was liberated and the rhodium catalyst was regenerated from nucleophilic attack with inversion. Inspired by Lautens’s pioneering efforts, we are interested in examining the mechanisms of rhodium-catalyzed ARO reactions. In this context, in view of the high importance of this methodology, in-depth mechanistic understandings of this reaction are in great demand. Through calculations, the structural information on the key transition states and intermediates as well as the energy profile could be investigated clearly. Herein, we report the full account of this study.

## Computational Details

Computations were performed using Gaussian 09 suite of quantum chemical program^19^. The M06-2X functional^20^,^21^ was used for the gas-phase geometry optimization of all species, which was demonstrated to be suitable for studying transition metal catalysis^22^,^23^. The LANL2DZ basis set^24^ was employed for Rh and Fe, and the 6–31 G(d) basis set was used for other atoms (GEN1). Frequency analysis at the same level was performed to ensure the stationary point as minimum or transition state. The intrinsic reaction coordinate (IRC) calculations were applied for each transition state to confirm that it connects the right reactant and product. For the solvent effect, the single-point calculations on the gas-phase optimized geometry with SMD solvation model^25^ (solvent = tetrahydrofuran) were applied. The single-point calculations were carried out using M06-2X with GEN2 (LANL2DZ for Rh and Fe, and 6–311 + G(d,p) for other atoms). The reported Gibbs free energy in this study was calculated by adding the gas-phase Gibbs free energy correction with the solution-phase single-point energy^26^. Fragment distortion and interaction energies were computed at the M06-2X/6–311 + G(d,p)-LANL2DZ level of theory using the M06-2X/6–31 G(d)-LANL2DZ geometries in the gas phase. Orbital composition analyses were conducted by the natural atomic orbital method with Multiwfn^27^. The 3D structures were prepared using CYLView^28^.

## Results and Discussion

**Figure 3:**
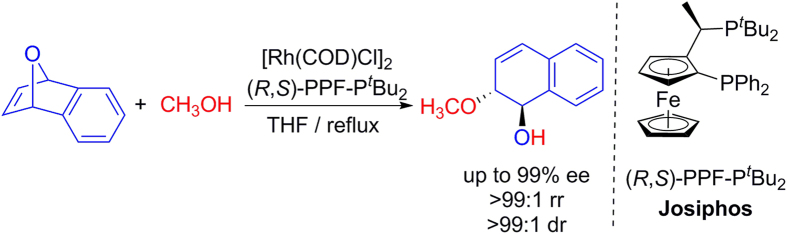
Computational model.

In this study, the ARO reactions between oxabicyclic alkene (**OA**) and methanol (**MeOH**) catalyzed by [Rh(cod)Cl]_2_/(*R*,*S*)PPF-P^*t*^Bu_2_ (Rh/Josiphos) were chosen as the benchmark model (Fig. 3)^8^. For the computational systems, the ligands are fully considered and optimized without any simplification.

We first turn our attention to the coordination of **OA** to **Rh**/**Josiphos** (Figure S1). There are four possible isomeric intermediates concerning different orientations of **OA** to **Josiphos** and Cl atom, which adopts a square-pyramidal geometry. The Rh(I) center of the formed intermediates is saturated coordinatively and electronically (i.e., an 18*e* Rh(I) complex). In **M1-a** and **M1-b**, the bridged oxygen of **OA** is positioned *cis* to the two tertiary butyl of the ligand, while in **M1-c** and **M1-d**, the alkenyl group of **OA** is positioned *cis* to the two tertiary butyl of the ligand. Our calculations show that two relatively lower energy structures **M1-a** and **M1-b** are about 4 and 10 kcal/mol more stable than **M1-c** and **M1-d**, respectively. This is due to the alkenyl group being positioned *cis* to the bulky *tert*-butyl group of the ligand in **M1-c** and **M1-d**, causing steric repulsion, while the small oxygen atom would not cause such larger steric effects.

**Figure 4:**
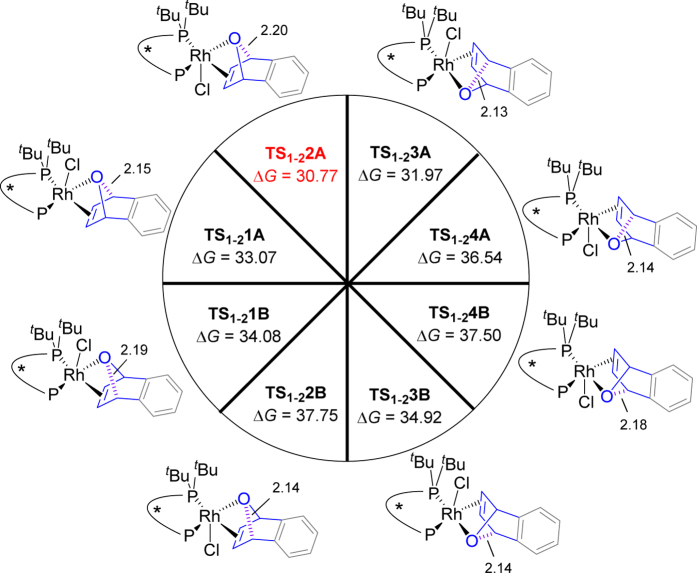
Eight possible transition states of ARO of **OA** (distances in Å, free energies in kcal/mol).

Once **OA** is coordinated to Rh (**M1-a**, **M1-b**, **M1-c**, **M1-d**), the ring-opening may occur in two directions through corresponding transition states, which is assumed as the enantio-determining step. As a result, there are eight possible transition states in total from the four intermediates as depicted in Fig. 4. The top four transition states can lead to the products experimentally obtained, while the bottom four transition states can result in the constitutional enantiomers. The lowest transition state is **TS**_**1-2**_**2A**.Taking all the eight transition states into consideration, the formation of (*R*,*R*)-product should be favored, and the calculated ee value is 98% by applying the Boltzmann distribution, which is in excellent agreement with the results obtained experimentally.

To gain further insights into the origins of enantioselectivity of Rh/Josiphos-catalyzed ARO reactions of **OA**, we analyzed the activation barriers using the distortion/interaction model^29^,^30^,^31^,^32^,^33^ (or activation strain model^34^,^35^,^36^), as listed in Table S2. In this model, the energy differences between the distorted transition structures and optimized ground-state structures are the distortion energies of the catalyst (*E*_dist-cat_) and **OA** (*E*_dist-**OA**_), respectively. The interaction energy (*E*_int_) is the difference between the activation energy (*E*_act_) and total distortion energy (*E*_dist_ = *E*_dist-cat_ + *E*_dist-**OA**_). It is obvious that although the distortion energy (*E*_dist-**OA**_ = 61.50 kcal/mol; Table S2, entry 3) is quite high in **TS**_**1-2**_**2A** with a longest breaking C–O bond of 2.20 Å (Figure S2), the lowest distortion energy of the catalyst (*E*_dist-cat_ = 31.90 kcal/mol; Table S2, entry 3) causes **TS**_**1-2**_**2A** to be the lowest one in energy.

**Figure 5:**
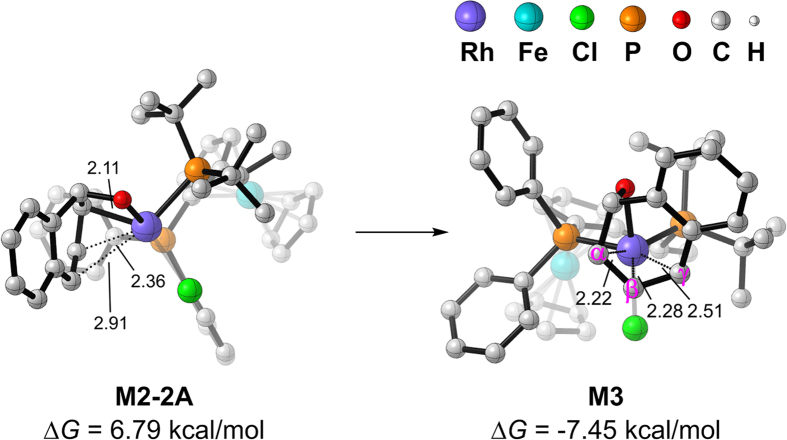
Optimized geometries of intermediates **M2-2A** and **M3**. Selected distances (in Å) and Gibbs free energies are shown.

After the ARO of **OA**, **M2-2A** is formed via **TS**_**1-2**_**2A**. **M2-2A** is a σ-allylrhodium(III) intermediate though the alkenyl part of **OA** coordinates to Rh weakly (2.36 Å, 2.91 Å, Fig. 5). To be coordinatively and electronically saturated, the **OA** moiety of **M2-2A** rotates around the Rh center and arrives at the π-allylrhodium(III) intermediate **M3**. **M3** is an octahedral intermediate and 14.24 kcal/mol more stable than **M2-2A** thermodynamically. Once **M3** has been formed, the rhodium alkoxide complex could be protonated by the alcohol prenucleophile to generate cationic rhodium complex and an alkoxide or phenoxide as proposed by Lautens *et al*. It was thought that the proton transfer has two effects: (i) the organorhodium species is made more electrophilic as a result of the obtained positive charge, and (ii) the nucleophile is rendered to be more nucleophilic by deprotonation^18^. Unfortunately, all of our efforts on locating such a transition state or intermediate concerning with proton transfer were failed. Starting from the two separated substrates **M3** and MeOH, we conducted a relaxed potential energy surface scan at M06-2X/6-31 G(d)–LANL2DZ level of theory (Figures S3–S6) and found no discernible peaks that would indicate a potential transition state and intermediate structure.

**Figure 6:**
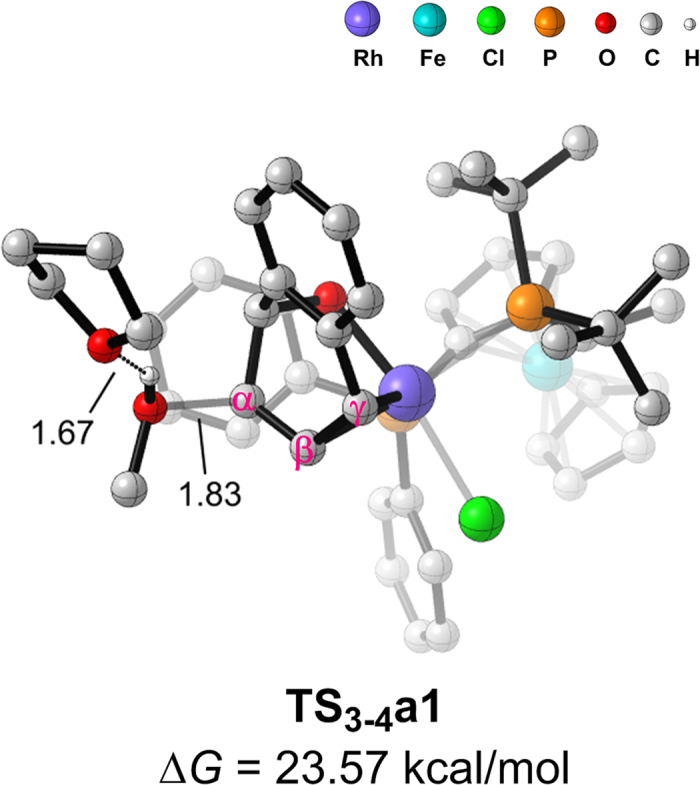
Optimized transition states of **TS**_**3-4**_**a1**. Selected distances are shown in Å.

Such repeated attempts suggest us that the methanol itself may act as a nucleophile in the assistance of some Lewis bases (i.e., the solvent THF). Fortunately, we have located such transitions states successfully (Fig. 6). The THF forms a hydrogen bond with the methanol (1.67 Å) to enhance the nucleophilicity of the nucleophile. In other words, the THF acts as a co-catalyst in the system. The successful application of aprotic silyl ketene acetals and enol ethers, acting as the nucleophiles, also supported such a process^16^. Though it was found that the reaction of an alkoxide or phenoxide does not give ring-opened products^18^, our calculations showed that the alkoxide can poison the catalyst by coordinating to the central Rh metal (Figure S9). As expected, the forming rhodium alkoxide complexes are quite stable thermodynamically, thus the substrates are inhibited from coordinating to the rhodium.

In general, the nucleophilic attack can take place either at α or γ carbon to the position of the alkoxy group. Thus, a detailed sampling of the conformers arising due to different orientations of methyl moiety of the methanol is carried out to identify energetically the regioselectivity (Figures S7 and S8). The results in these two figures clearly indicate that the nucleophilic attack at Cα is favored by more than 15 kcal/mol relative to Cγ, which indicates that Cα position is the only electrophilic site of **M3**. Unfortunately, we failed to locate **TS**_**3-4**_**b5** and **TS**_**3-4**_**b6** due to the steric repulsion between the methyl group of the methanol and the phenyl ring of **OA**.

**Figure 7:**
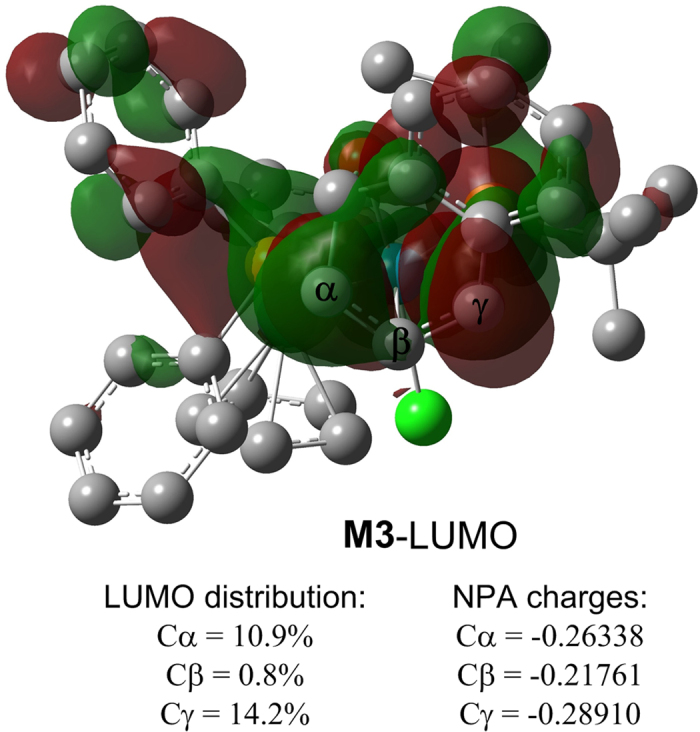
The LUMO distribution and NPA charges of **M3**.

In order to probe the origins of the regioselectivity among the potential electrophilic sites of **M3**, we conducted orbital composition analysis of the lowest unoccupied molecular orbital (LUMO) and calculated the natural population analysis (NPA) charges (Fig. 7). The LUMO distribution shows that there are two potential electrophilic sites in the allyl moiety of **M3** (10.9% for Cα; 14.2% for Cγ). Though the LUMO distribution of Cα is a bit lower than Cγ, conventional chemical wisdom would anticipate that the information of the atomic charge distribution in a molecule should suffice in quantifying the property^37^,^38^,^39^,^40^. As shown in Fig. 7, Cα carries more positive charge than Cγ indicating that Cα should be the electrophilic site. In addition, it is worth noting that nucleophilic attack at Cγ would destroy the π–π conjugation between the allyl group and the phenyl ring of **OA**. Comparatively, the same regioselectivity is also observed when phenol, *N*-methylaniline and mercaptan act as the nucleophilic reagents respectively (Figures S10–S12).

We concurrently studied the possible side reaction of the formation of the *syn*-1,2 diastereomers of the product. As shown in Figure S13, one methanol is pressed into **M3** to coordinate with Rh, which is endergonic by 8.38 kcal/mol and forms a σ-allylrhodium(III) intermediate **M3-syn**. Subsequently, the proton of the methanol transfers to the oxygen of the opening **OA**, crossing a barrier of 15.38 kcal/mol (**TS**_**3-4**_**syn** relative to **M3**). After that, **M4-syn** undergoes C−O reductive elimination at the Rh(III) center via **TS**_**4-5**_**syn**, leading to the *syn*-1,2 diastereomers of the product. The C−O reductive elimination bears an insurmountable barrier of about 50 kcal/mol (**TS**_**4-5**_**syn** relative to **M3**), which indicates that it is impossible to generate the *syn*-1,2 diastereomers of the product under the experimental conditions. The results are in quantitative agreement with the diastereoselectivity observed experimentally.

**Figure 8:**
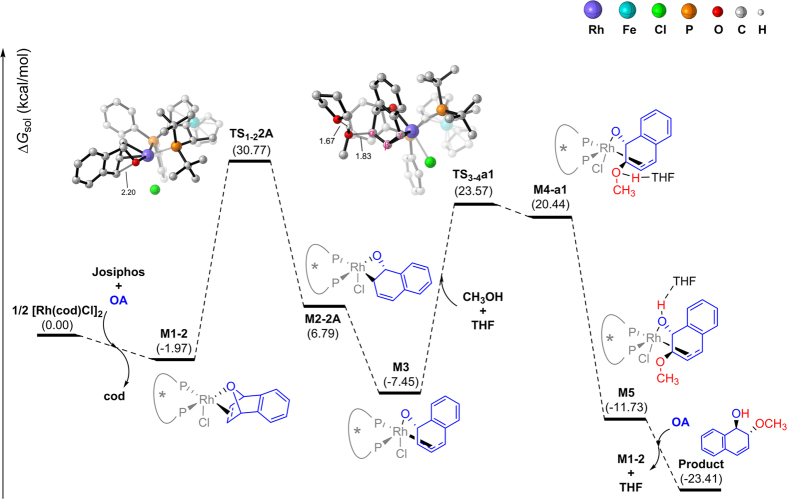
Gibbs free energy profiles for the most favored path of the ARO reaction.

Through our calculations, the computed Gibbs free energy surface for the most favored path of the ARO reaction of oxabicyclic alkene with methanol catalyzed by Rh/Josiphos is summarized in Fig. 8. [Rh(cod)Cl]_2_ first undergoes cleavage to coordinate with the ligand **Josiphos** and the substrate **OA** to form a relatively stable intermediate **M1-b** by releasing 1.97 kcal/mol of energy, which is a coordinatively and electronically saturated square-pyramidal structure. The ring-opening step via **TS**_**1-2**_**2A** has an activation energy of 32.74 kcal/mol, which is the rate- and enantio-determining step in the whole catalytic cycle and produces the σ-allylrhodium(III) complex **M2-2A**. We next considered the π-allylrhodium(III) complex **M3**, which is 14.24 kcal/mol more stable than **M2-2A**. **M3** then undergoes nucleophilic attack of one methanol in the assistance of THF via **TS**_**3-4**_**a1**, which is a late transition state, to form a new carbon−oxygen bond. The barrier for the nucleophilic attack is 31.02 kcal/mol, which is competitive with the ring-opening step. Finally, the proton transfer is completed by the solvent, which is about 32 kcal/mol exergonic and another molecule of the substrate **OA** replace the formed product to start the next catalytic cycle.

## Conclusions

In summary, the mechanism, stereo- and regioselectivity of the Rh/Josiphos-catalyzed ARO reactions of oxabicyclic alkenes with methanol have been studied by DFT calculations. Based on the model advanced by Lautens *et al*., we performed the M06-2X calculations to explore the detailed mechanism carefully. Through calculations, we have found that the ring-opening step via **TS**_**1-2**_**2A** is the rate- and enantio-determining step in the whole catalytic cycle with a lowest activation energy of 32.74 kcal/mol. The lower distortion energy of the catalyst in the ring-opening transition state makes the breaking C−O activation more favorable. In the nucleophlic attack step, the solvent THF forms a hydogen bond with the nuclephile, and assists the attack at the allyl group of the π-allylrhodium(III) intermediate **M3**. In terms of regioselectivity, the formed π-allylrhodium(III) **M3** is attacked by nucleophiles at the Cα site which bears more positive charge. Also, the formation of the *syn*-1,2 diastereomers of the product is inhibited by the high barrier of the C−O reductive elimination at the Rh(III) center. In the study of transition-metal-catalyzed reactions, most attention is paid on the metal center, experimentally and theoretically. In a reaction system, the most species is the solvent usually but it is often regarded as the reaction medium only. Though the solvent effects appear in many transition-metal-catalyzed reactions, less attention is paid on the association with reaction process. Here we present an example that how the solvent would play a role in the reaction mechanism explicitly. In combination with those experimental findings, the mechanistic studies presented herein are likely to induce a paradigm shift in the development of more active catalysts and may be helpful in the search for more selective catalysts.

## Additional Information

**How to cite this article**: Qi, Z.-H. *et al*. Mechanism, reactivity, and regioselectivity in rhodium-catalyzed asymmetric ring-opening reactions of oxabicyclic alkenes: a DFT Investigation. *Sci. Rep.* **7**, 40491; doi: 10.1038/srep40491 (2017).

**Publisher's note:** Springer Nature remains neutral with regard to jurisdictional claims in published maps and institutional affiliations.

## Supplementary Material

Supplementary Information

## Figures and Tables

**Figure 1:**
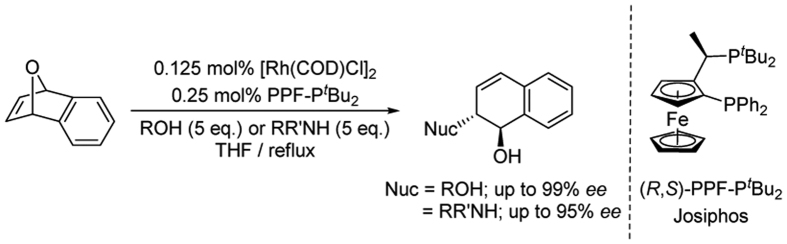
Rhodium-catalyzed asymmetric alcoholysis and aminolysis of oxabenzonorbornadiene^8^.

**Figure 2:**
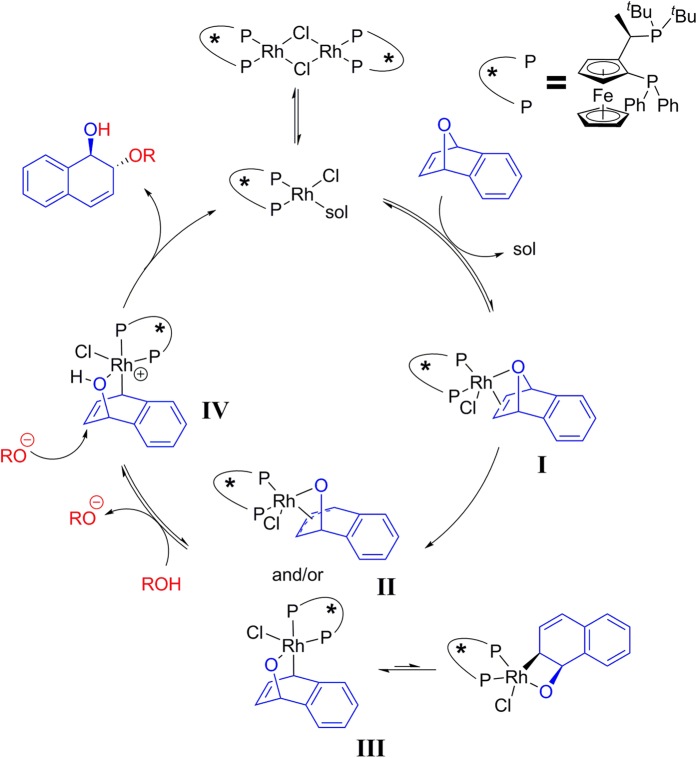
Lautens’s catalytic cycle^18^.
