# Supplementary material for: Mechanism, reactivity, and regioselectivity in rhodium-catalyzed asymmetric ring-opening reactions of oxabicyclic alkenes: a DFT Investigation
Source: Sci Rep. 2017 Jan 11;7:40491. doi: 10.1038/srep40491 (PMC5225450; doi:10.1038/srep40491)
Supplement: Supplementary Information [file srep40491-s1.doc]

**Supporting Information (SI)**

**Mechanism, Reactivity, and Regioselectivity in Rhodium-Catalyzed Asymmetric Ring-Opening Reactions of Oxabicyclic Alkenes: A DFT Investigation**

Zheng-Hang Qi, Yi Zhang, Yun Gao, Ye Zhang, Xing-Wang Wang,* and Yong Wang*

College of Chemistry, Chemical Engineering and Materials Science, Soochow University, Suzhou 215123, P. R. China.

Email: yowang*@suda.edu.cn*; wangxw*@suda.edu.cn*

**Contents**

1. Complete Gaussian 09 Reference ………………………………………………S2

2. Fig. S1–S18…………….……………………………………….………………S4

3. Table S4…………………………………………………………………………S11

4. Coordinates of DFT-Computed Stationary Points…………………....…………S13

**Complete Gaussian 09 Reference**

Frisch, M. J.; Trucks, G. W.; Schlegel, H.; Scuseria, G. E.; Robb, M. A.; Cheeseman, J. R.; Scalmani, G.; Barone, V.; Mennucci, B.; Petersson, G. A.; Nakatsuji, H.; Caricato, M.; Li, X.; Hratchian, H. P.; Izmaylov, A. F.; Bloino, J.; Zheng, G.; Sonnenberg, J. L.; Hada, M.; Ehara, M.; Toyota, K.; Fukuda, R.; Hasegawa, J.; Ishida, M.; Nakajima, T.; Honda, Y.; Kitao, O.; Nakai, H.; Vreven, T.; Montgomery, J. A.; Peralta, J. E.; Ogliaro, F.; Bearpark, M.; Heyd, J. J.; Brothers, E.; Kudin, K. N.; Staroverov, V. N.; Kobayashi, R.; Normand, J.; Raghavachari, K.; Rendell, A.; Burant, J. C.; Iyengar, S. S.; Tomasi, J.; Cossi, M.; Rega, N.; Millam, J. M.; Klene, M.; Knox, J. E.; Cross, J. B.; Bakken, V.; Adamo, C.; Jaramillo, J.; Gomperts, R.; Stratmann, R. E.; Yazyev, O.; Austin, A. J.; Cammi, R.; Pomelli, C.; Ochterski, J. W.; Martin, R. L.; Morokuma, K.; Zakrzewski, V. G.; Voth, G. A.; Salvador, P.; Dannenberg, J. J.; Dapprich, S.; Daniels, A. D.; Farkas; Foresman, J. B.; Ortiz, J. V.; Cioslowski, J.; Fox, D. J. *Gaussian 09, Revision C.01*, Gaussian Inc., Wallingford CT 2010.

**Fig. S1** Four types of coordination of oxabicyclic alkene to Rh. Gibbs free energies are relative to the separated catalysts and reactants.

**Fig. S2** Eight possible transition states of asymmetric ring-opening of **OA** (distances in Å).

| **Table S1.** Gibbs free energies of the transition states of asymmetric ring-opening of **OA** (in kcal/mol). | | | | | | | | | | |
| --- | --- | --- | --- | --- | --- | --- | --- | --- | --- | --- |
| TS | | **TS_1-2_1A** | | **TS_1-2_2A** | | | **TS_1-2_3A** | | **TS_1-2_4A** | |
| Δ*G*^‡^ | | 33.07 | | 30.77 | | | 31.97 | | 36.54 | |
| TS | | **TS_1-2_1B** | | **TS_1-2_2B** | | | **TS_1-2_3B** | | **TS_1-2_4B** | |
| Δ*G*^‡^ | | 34.08 | | 37.75 | | | 34.92 | | 37.50 | |
|  | | | | | | | | | | |
| **Table S2.** Distortion/Interaction analysis for the transition states of asymmetric ring opening of **OA** (energies are in kcal/mol). | | | | | | | | | | |
| entry | TS | | *E*_act_ | | *E*_dist-cat_ | *E*_dist-_**_OA_** | | *E*_dist_ | | *E*_int_ |
| 1 | **TS_1-2_1A** | | 24.47 | | 41.91 | 57.85 | | 99.76 | | -75.29 |
| 2 | **TS_1-2_1B** | | 24.33 | | 40.49 | 61.71 | | 102.20 | | -77.87 |
| 3 | **TS_1-2_2A** | | 21.91 | | 31.90 | 61.50 | | 93.40 | | -71.48 |
| 4 | **TS_1-2_2B** | | 30.57 | | 39.89 | 57.78 | | 97.67 | | -67.10 |
| 5 | **TS_1-2_3A** | | 25.49 | | 37.67 | 59.50 | | 97.17 | | -71.68 |
| 6 | **TS_1-2_3B** | | 28.67 | | 43.29 | 59.01 | | 102.30 | | -73.64 |
| 7 | **TS_1-2_4A** | | 25.34 | | 39.27 | 56.13 | | 95.40 | | -70.06 |
| 8 | **TS_1-2_4B** | | 28.39 | | 42.04 | 61.50 | | 103.54 | | -75.15 |

**Fig. S3** Protonation of **M3** by one molecule of methanol.

**Fig. S4** Relaxed potential energy surface scan for the protonation of **M3** by one molecule of methanol.

**Fig. S5** Protonation of **M3** by three molecules of methanol.

**Fig. S6** Relaxed potential energy surface scan for the protonation of **M3** by three molecules of methanol.

**Fig. S7** The Newman projection along the incipient C−C bond and Gibbs free energies of nucleophilic attack at Cα.

**Fig. S8** The Newman projection along the incipient C−C bond and Gibbs free energies of nucleophilic attack at Cγ.

**Fig. S9** Optimized rhodium(I) alkoxide complexes. Free energies of **cat-MeO-1** and **cat-MeO-2** are relative to the separated reactants and MeO^–^. Free energy of **cat-2MeO** is given by ½[Rh(cod)Cl]_2_ +**Josiphos** + MeONa +MeO^–^ → **cat-2MeO** + **cod** + NaCl. Selected distances are shown in angstrom.

**Fig. S10** Transition states for the nucleophilic attack of phenol at Cα (**PhO-TS-a**) and Cγ (**PhO-TS-b**) of the allyl group of **M3**. The bottom six structures are the possible intermediates of the nucleophilic attack at Cγ. Five of them could be located successfully and are more than 10 kcal/mol unfavored energetically compared with the transition states for the nucleophilic attack at Cα (**PhO-TS-a**).

**Fig. S11** Transition states for the nucleophilic attack of *N*-methylaniline at Cα (**PhN-TS-a**) and Cγ (**PhN-TS-b**) of the allyl group of **M3**. The bottom six structures are the possible intermediates of the nucleophilic attack at Cγ. All of them could be located successfully and are more than 7 kcal/mol unfavored energetically compared with the transition states for the nucleophilic attack at Cα (**PhN-TS-a**).

**Fig. S12** Transition states for the nucleophilic attack of mercaptan at Cα (**S-TS-a**) of the allyl group of **M3**. The bottom six structures are the possible intermediates of the nucleophilic attack at Cγ. All of them could be located successfully and are more than 10 kcal/mol unfavored energetically compared with the transition states for the nucleophilic attack at Cα (**S-TS-a**).

**Fig. S13** Gibbs free energy profiles for the formation of the *syn*-1,2 diastereomers of the product.

In order to test and compare with another DFT method B3LYP, which is widely used for studying the Rh-catalyzed reactions (Org. Biomol. Chem., 2015, 13, 6587–6597; RSC Adv., 2015, 5, 100147–100158), we re-optimized the key structures in the level of B3LYP/6-31G(d), Lanl2DZ, SMD(THF). We are pleased to find the same steric effects could be observed and the enantioselectivity is reproduced again (Figure S14 and S15). It is worth noting that the energy barrier of ring-opening is decreased to 20.73 kcal (**TS_1-2_A-b3lyp** in Table S3), which is much lower than the results obtained by M06-2X. However, the energy barrier of nucleophilic attack of methanol is increased to ~40 kcal/mol (Figure S16) which is much higher than the M06-2X-computed result. This may be due to the fact that B3LYP is not able to reliably model systems in which noncovalent interactions are important (J. Phys. Chem. Lett. 2012, 3, 1738−1744). When the canceling effect of the the individual errors is involved, the same regioselectivity could also be reproduced for the nucleophile phenol, *N*-methylaniline and mercaptan (Figure S17). In addition, the energy barrier of the formation of the *syn*-1,2 diastereomers of the product is also as high as 48.88 kcal/mol.

**Fig. S14** Four types of coordination of oxabicyclic alkene to Rh. Gibbs free energies are relative to the separated catalysts and reactants.

**Fig. S15** Eight possible transition states of asymmetric ring-opening of **OA** (distances in Å).

| **Table S3.** Gibbs free energies of the transition states of asymmetric ring-opening of **OA** (in kcal/mol). | | | | |
| --- | --- | --- | --- | --- |
| TS | **TS_1-2_1A-b3lyp** | **TS_1-2_2A-b3lyp** | **TS_1-2_3A-b3lyp** | **TS_1-2_4A-b3lyp** |
| Δ*G*^‡^ | 31.41 | 20.73 | 31.38 | 34.39 |
| TS | **TS_1-2_1B-b3lyp** | **TS_1-2_2B-b3lyp** | **TS_1-2_3B-b3lyp** | **TS_1-2_4B-b3lyp** |
| Δ*G*^‡^ | 32.02 | 28.14 | 33.25 | 37.09 |
|  | | | | |

**Fig. S16.** Gibbs free energy profiles for the most favored path of the ARO reaction.

**Fig. S17.** Transition states for the nucleophilic attack of phenol, *N*-methylaniline and mercaptan at Cα (**-TS-a**) of the allyl group of **M3** (left) and the intermediates of the nucleophilic attack at Cγ (right).

**Fig. S18** Gibbs free energy profiles for the formation of the *syn*-1,2 diastereomers of the product.

**Table S4. Thermal correction of Gibbs free energy (TCG, hartree) and total electronic energies (*E***, hartree) in THF solvent for all species involved in this study

| Species | TCG | *E* | Species | TCG | *E* |
| --- | --- | --- | --- | --- | --- |
| **MeOH** | 0.029676 | -115.7091978 | **TS_3-4_b2** | 0.894485 | -3428.1837839 |
| **THF** | 0.090672 | -232.4134492 | **TS_3-4_b3** | 0.892418 | -3428.1747312 |
| **cod** | 0.150964 | -311.9706210 | **TS_3-4_b4** | 0.893135 | -3428.1817429 |
| **OA** | 0.122342 | -460.9795950 | **M4-b1** | 0.893894 | -3428.1771479 |
| **Josiphos** | 0.579158 | -2049.3733084 | **M4-b2** | 0.893724 | -3428.1855077 |
| **[Rh(cod)Cl]_2_** | 0.324107 | -1763.3563389 | **M4-b3** | 0.893044 | -3428.1817495 |
| **M1-a** | 0.731261 | -3080.0739848 | **M4-b4** | 0.893240 | -3428.1835254 |
| **M1-b** | 0.729201 | -3080.0802093 | **M4-b5** | 0.894422 | -3428.1809115 |
| **M1-c** | 0.729758 | -3080.0653895 | **M5** | 0.892429 | -3428.2612796 |
| **M1-d** | 0.731274 | -3080.0662054 | **Product** | 0.172879 | -576.7438213 |
| **TS_1-2_1A** | 0.727706 | -3080.0228607 | **M3-syn** | 0.782771 | -3195.8086738 |
| **TS_1-2_1B** | 0.729188 | -3080.0227481 | **TS_3-4_syn** | 0.779440 | -3195.7941848 |
| **TS_1-2_2A** | 0.727785 | -3080.0266143 | **M4-syn** | 0.781934 | -3195.7950754 |
| **TS_1-2_2B** | 0.726865 | -3080.0145743 | **TS_4-5_syn** | 0.777042 | -3195.7352745 |
| **TS_1-2_3A** | 0.726449 | -3080.0233624 | **M5-syn** | 0.780652 | -3195.8201642 |
| **TS_1-2_3B** | 0.726056 | -3080.0182705 | **PhO-TS-a** | 0.944189 | -3619.9279813 |
| **TS_1-2_4A** | 0.728812 | -3080.0184497 | **PhO-TS-b** | 0.940885 | -3619.8934312 |
| **TS_1-2_4B** | 0.726230 | -3080.0143328 | **PhO-b2** | 0.941598 | -3619.9003557 |
| **M2-1A** | 0.731957 | -3080.0770695 | **PhO-b3** | 0.938239 | -3619.8910180 |
| **M2-1B** | 0.730685 | -3080.0755880 | **PhO-b4** | 0.939457 | -3619.8968334 |
| **M2-2A** | 0.730064 | -3080.0671127 | **PhO-b5** | 0.942888 | -3619.8972694 |
| **M2-2B** | 0.729076 | -3080.0840244 | **PhO-b6** | 0.939546 | -3619.9028547 |
| **M2-3A** | 0.725241 | -3080.0711195 | **PhN-TS-a** | 0.982013 | -3639.3641505 |
| **M2-3B** | 0.729411 | -3080.0733778 | **PhN-TS-b** | 0.981254 | -3639.3307541 |
| **M2-4A** | 0.732129 | -3080.0686701 | **PhN-b1** | 0.983307 | -3639.3538824 |
| **M2-4B** | 0.730122 | -3080.0861675 | **PhN-b2** | 0.980644 | -3639.3461451 |
| **M3** | 0.730194 | -3080.0899309 | **PhN-b3** | 0.982626 | -3639.3518387 |
| **TS_3-4_a1** | 0.890540 | -3428.2031343 | **PhN-b4** | 0.985126 | -3639.3451403 |
| **TS_3-4_a2** | 0.891031 | -3428.2011289 | **PhN-b5** | 0.986259 | -3639.3525742 |
| **TS_3-4_a3** | 0.894836 | -3428.2026658 | **PhN-b6** | 0.985867 | -3639.3430849 |
| **TS_3-4_a4** | 0.893461 | -3428.2060529 | **S-TS-a** | 0.885064 | -3751.1716239 |
| **TS_3-4_a5** | 0.895374 | -3428.2072628 | **S-b1** | 0.885003 | -3751.1457911 |
| **TS_3-4_a6** | 0.893578 | -3428.2044492 | **S-b2** | 0.884924 | -3751.1480601 |
| **M4-a1** | 0.893358 | -3428.2109439 | **S-b3** | 0.884078 | -3751.1439765 |
| **M4-a2** | 0.893051 | -3428.2088868 | **S-b4** | 0.888290 | -3751.1542534 |
| **M4-a3** | 0.895395 | -3428.2071429 | **S-b5** | 0.886665 | -3751.1518248 |
| **M4-a4** | 0.895586 | -3428.2175191 | **S-b6** | 0.887826 | -3751.1501417 |
| **M4-a5** | 0.896816 | -3428.2130610 | **cat-MeO-1** | 0.619473 | -2734.2963679 |
| **M4-a6** | 0.896389 | -3428.2111862 | **cat-MeO-2** | 0.619590 | -2734.2937806 |
| **TS_3-4_b1** | 0.893342 | -3428.1733903 | **cat-2MeO** | 0.656249 | -2389.1364745 |

**5. Coordinates and Energies of DFT-Computed Stationary Points**

**MeOH**

C -0.65922200 -0.02000200 0.00000800

H -1.02804100 -0.54450900 -0.89195100

H -1.02841900 -0.54397200 0.89215700

H -1.07898000 0.98780600 -0.00043000

O 0.74450900 0.12266800 0.00002700

H 1.13469700 -0.76065700 -0.00004200

**THF**

C 1.15774400 -0.42815000 0.13687000

O -0.00015500 -1.24303000 -0.00001900

C -1.15785800 -0.42787500 -0.13684500

C -0.72713200 0.99082100 0.23657700

C 0.72738900 0.99064000 -0.23659200

H 1.51243200 -0.46797000 1.17665200

H 1.94605500 -0.82490300 -0.51049600

H -1.51261100 -0.46764600 -1.17660300

H -1.94623100 -0.82443200 0.51057700

H -1.34330600 1.76028500 -0.23442600

H -0.77076400 1.12827200 1.32232600

H 0.77107200 1.12805300 -1.32234200

H 1.34374500 1.75996100 0.23440500

**cod**

C -1.08818600 -1.07628800 0.67598400

H -1.78123400 -1.87046400 0.97536400

H -0.63770800 -0.71648600 1.60195600

C -1.91938400 0.03513700 0.01436700

H -2.68954100 0.37648200 0.72114600

H -2.47191900 -0.40653200 -0.82573900

C 1.91938400 -0.03514000 0.01438200

H 2.47193200 0.40653200 -0.82571300

H 2.68953100 -0.37649200 0.72117000

C 1.08818400 1.07628500 0.67599800

H 0.63770200 0.71648200 1.60196900

H 1.78123200 1.87045800 0.97538200

C 0.05458000 1.67118300 -0.25011200

H 0.37994400 2.56205100 -0.78649500

C -1.17773100 1.23417400 -0.51663100

H -1.75645300 1.82360300 -1.22794200

C 1.17773400 -1.23417000 -0.51663400

H 1.75645900 -1.82359100 -1.22794900

C -0.05458000 -1.67118000 -0.25012600

H -0.37994400 -2.56204100 -0.78652200

**OA**

C -0.00376000 1.38098600 1.41642200

C -0.16334300 2.57654100 0.69401100

C -0.16334300 2.57654100 -0.69401100

C -0.00376000 1.38098600 -1.41642200

C 0.15964700 0.21664600 -0.70080100

H -0.00244500 1.38872400 2.50275900

H -0.28357200 3.51323100 1.22972700

H -0.28357200 3.51323100 -1.22972700

H -0.00244500 1.38872400 -2.50275900

C 0.15964700 0.21664600 0.70080100

C 0.35068600 -1.26258500 1.06690200

C -0.95058300 -1.98118300 0.66501000

H 0.78325900 -1.49087900 2.03988000

C 0.35068600 -1.26258500 -1.06690200

C -0.95058300 -1.98118300 -0.66501000

H 0.78325900 -1.49087900 -2.03988000

O 1.21600600 -1.66800300 0.00000000

H -1.71715100 -2.32149600 1.34879200

H -1.71715100 -2.32149600 -1.34879200

**Josiphos**

P -0.72283300 1.09939000 0.65537700

C -0.06896700 2.60220200 -0.19122700

C -0.17072100 3.82261000 0.48664000

C 0.57126600 2.57313800 -1.43222800

C 0.34750400 4.98895300 -0.06646800

H -0.66629000 3.86092900 1.45475300

C 1.09780500 3.74010300 -1.98369800

H 0.65913800 1.63169400 -1.96796000

C 0.98702300 4.94983300 -1.30375900

H 0.25615100 5.92857700 0.47022800

H 1.59374900 3.70136300 -2.94934900

H 1.39673300 5.85836600 -1.73440600

C -2.52217000 1.52685000 0.68325800

C -3.27200300 1.11369100 1.78847700

C -3.16451600 2.24674500 -0.33194000

C -4.64002600 1.37207300 1.86323000

H -2.77534600 0.59258500 2.60314700

C -4.53046100 2.50354000 -0.26305900

H -2.58464800 2.61629300 -1.17454500

C -5.27251900 2.05943300 0.83108200

H -5.20761900 1.04389700 2.72886400

H -5.01736100 3.05724600 -1.06030800

H -6.33736000 2.26448100 0.88536500

C 1.36508900 -1.51391800 0.27415500

H 0.90270600 -1.42324100 1.26827300

C 1.88077900 -2.94981400 0.12859200

H 2.34303700 -3.12355200 -0.84619800

H 2.61960700 -3.19721100 0.89117400

H 1.04340400 -3.65264900 0.21990700

P 2.58244100 -0.05339000 0.30250300

C 3.93605200 -0.44640600 -0.97031400

C 4.93574300 -1.56099900 -0.64667200

H 4.45084600 -2.52391500 -0.46718800

H 5.62353900 -1.68946800 -1.49395800

H 5.54544500 -1.31888000 0.22893300

C 3.20760300 -0.77232400 -2.28473100

H 2.68571800 -1.73072000 -2.25299000

H 2.46993500 -0.00071000 -2.53506700

H 3.94012800 -0.81576000 -3.10096400

C 4.70827800 0.86299900 -1.21551700

H 5.31327100 1.16143700 -0.35670000

H 5.38810200 0.72696200 -2.06687500

H 4.02325600 1.68456700 -1.45117800

C 3.30276000 -0.15495500 2.05904500

C 4.54872700 0.73739900 2.13797900

H 4.36282300 1.72672000 1.70551800

H 4.82390300 0.87848300 3.19100100

H 5.41045500 0.29280900 1.63170200

C 2.23224200 0.47694100 2.96640800

H 2.01324800 1.50671500 2.66609500

H 1.28813000 -0.07717000 2.94576800

H 2.59500500 0.48508400 4.00273100

C 3.64065900 -1.54180900 2.61436200

H 2.74087100 -2.15274000 2.73960900

H 4.34746600 -2.08966700 1.98503200

H 4.09494500 -1.43223100 3.60835300

C -3.60390100 -2.01731700 0.39902600

Fe -1.71940100 -1.98144700 -0.58951400

C -2.57355700 -2.30678400 1.33457500

C -3.57756100 -3.02486700 -0.60132100

C -2.53092800 -3.93241100 -0.28906700

C -1.91149800 -3.48906900 0.91056800

C -1.52598000 -0.26261600 -1.82347300

C -1.13345900 -1.40552300 -2.55846300

C -0.02758400 -1.99621000 -1.89038500

C 0.27464300 -1.22422800 -0.73487500

C -0.66654500 -0.13843800 -0.68897200

H -2.30720300 -1.70838500 2.19525500

H -4.20702100 -3.06112800 -1.48003400

H -2.23191100 -4.78674900 -0.88105400

H -1.06577400 -3.95261900 1.40018400

H -2.37577600 0.37060300 -2.03999500

H -1.62267800 -1.79464500 -3.44128800

H 0.46465900 -2.91365100 -2.18538000

H -4.24965600 -1.14903700 0.41536700

**[Rh(cod)Cl]_2_**

Rh 1.79782100 0.02876500 -0.09370500

Rh -1.79775100 0.02875400 -0.09347100

Cl -0.00007300 1.75225200 0.17717900

Cl 0.00005800 -1.55370400 -0.82915600

C 3.21205900 1.12731800 1.08825600

C 3.27181300 1.55624900 -0.23970600

C 4.31477000 1.14089900 -1.25557100

C 4.60883700 -0.37011100 -1.23890100

C 3.37512500 -1.18818100 -0.89039200

C 3.06454300 -1.60611400 0.40549100

C 3.84984400 -1.26394000 1.65416300

C 4.25045100 0.22057800 1.73017700

C -3.37513800 -1.18752300 -0.89123000

C -3.06485600 -1.60644700 0.40438600

C -3.85019600 -1.26494000 1.65319700

C -4.25070000 0.21955500 1.72996200

C -3.21193500 1.12657800 1.08904700

C -3.27152600 1.55642700 -0.23864700

C -4.31475000 1.14199400 -1.25463300

C -4.60865100 -0.36911000 -1.23955900

H 2.59200400 1.70939700 1.76722700

H 2.70698500 2.45065600 -0.49730700

H 3.92988800 1.41412800 -2.24341500

H 5.23961500 1.71766700 -1.11122700

H 4.97284200 -0.67545400 -2.22433500

H 5.41241300 -0.60454500 -0.53410500

H 2.90389800 -1.72561200 -1.71105100

H 2.38409700 -2.45045800 0.50372000

H 3.21249000 -1.50130000 2.51200100

H 4.73575800 -1.90974800 1.73684400

H 4.37193400 0.50496600 2.77970200

H 5.22321100 0.38862400 1.25815600

H -2.90359000 -1.72431200 -1.71212600

H -2.38441800 -2.45084900 0.50208900

H -4.73618300 -1.91068900 1.73556400

H -3.21287000 -1.50275100 2.51093200

H -5.22319800 0.38797900 1.25754900

H -4.37273200 0.50327200 2.77960500

H -2.59191500 1.70803500 1.76858200

H -2.70656300 2.45090500 -0.49566900

H -5.23964900 1.71841800 -1.10922700

H -3.93034900 1.41645000 -2.24230900

H -5.41260700 -0.60429600 -0.53544000

H -4.97211600 -0.67352500 -2.22548100

**M1-a**

P 0.42750400 -1.11599100 -0.19442200

C 0.11502200 -2.63378800 -1.17971700

C -0.40950900 -3.77286500 -0.55507300

C 0.23608300 -2.61765000 -2.57401800

C -0.77243300 -4.88756300 -1.30620000

H -0.54250800 -3.79567100 0.52281400

C -0.11598500 -3.74008300 -3.31867200

H 0.56417500 -1.71666700 -3.07933600

C -0.61632700 -4.87653200 -2.68962100

H -1.17734900 -5.76246000 -0.80708100

H -0.01523400 -3.71296600 -4.39889300

H -0.89569900 -5.74657200 -3.27588600

C 0.91294700 -1.81454800 1.43662600

C 0.48391800 -1.21677400 2.61934400

C 1.75177000 -2.93520100 1.50356700

C 0.88969100 -1.72082800 3.85659900

H -0.17717800 -0.36043500 2.56234400

C 2.14037700 -3.45066600 2.73418600

H 2.09660900 -3.40422100 0.58524400

C 1.71256000 -2.84082200 3.91502200

H 0.55088000 -1.24309800 4.77087900

H 2.78408100 -4.32425400 2.77505100

H 2.02218000 -3.24104200 4.87547500

C 1.88801200 2.03580400 0.22587100

H 1.87463900 1.58341300 1.22966800

C 2.86839600 3.21627100 0.26141200

H 2.96971400 3.68726600 -0.71835100

H 2.56246600 3.98687000 0.96579900

H 3.86128600 2.85760100 0.55667400

P 0.05293100 2.40568700 -0.03409800

C -0.07158600 3.64353800 -1.46557200

C 0.33153300 5.08924900 -1.14849800

H 1.34207800 5.17432800 -0.74007900

H 0.30342000 5.66354900 -2.08318600

H -0.36374900 5.57063400 -0.45457600

C 0.82235300 3.11196600 -2.60010200

H 1.88676600 3.23306100 -2.38138300

H 0.61402700 2.06100000 -2.82128600

H 0.60336400 3.69041300 -3.50633300

C -1.52281900 3.64071900 -1.98060500

H -2.23452600 4.01997200 -1.24351900

H -1.57391300 4.29977700 -2.85689500

H -1.82827100 2.63958400 -2.29597500

C -0.49289400 3.26644700 1.57934400

C -1.91429500 3.81009200 1.35662700

H -2.55705000 3.08930300 0.83865100

H -2.36634100 4.03415000 2.33165800

H -1.91340500 4.73682400 0.77721400

C -0.57467500 2.19258800 2.67338800

H -1.28046900 1.40249000 2.40299700

H 0.40054300 1.73197200 2.86864300

H -0.90907500 2.66044200 3.60840800

C 0.38902200 4.39350400 2.13843400

H 1.31981700 4.00015300 2.55820800

H 0.63285700 5.16305800 1.40469700

H -0.15006600 4.88003300 2.96185300

Rh -1.29627500 0.26212000 -0.34797500

C 4.84783000 -1.87232600 1.09326200

Fe 3.99013300 -0.33836900 -0.12486400

C 4.26111600 -0.88449500 1.93017500

C 5.86649900 -1.24984900 0.32484500

C 5.90865800 0.12322500 0.68158200

C 4.91792200 0.34595700 1.67618700

C 2.83152600 -1.04944900 -1.78532400

C 3.78551200 -0.11122500 -2.23473700

C 3.51844100 1.12255700 -1.59021400

C 2.39670500 0.96690000 -0.72805200

C 1.95183700 -0.39324000 -0.87108300

H 3.43594900 -1.03433000 2.61390100

H 6.46767600 -1.72636900 -0.43760400

H 6.55000600 0.87541400 0.24195100

H 4.68387600 1.29529500 2.13937700

H 2.77274700 -2.08988800 -2.07329400

H 4.60369700 -0.31043700 -2.91300100

H 4.11412600 2.01853800 -1.69466000

H 4.55300200 -2.91012500 1.03021300

C -5.97177900 -0.30201100 2.16325100

C -7.14249600 -0.99169400 1.82197700

C -7.31998500 -1.50764100 0.54105000

C -6.33451900 -1.35095200 -0.44248600

C -5.18655600 -0.66083900 -0.10335100

H -5.83793000 0.09923200 3.16386300

H -7.92350700 -1.12110900 2.56469800

H -8.23782700 -2.03444900 0.29918400

H -6.47694500 -1.75641300 -1.43976700

C -5.00711100 -0.14413700 1.18678200

C -3.62777500 0.48279800 1.16758900

C -2.55698400 -0.61886700 1.07205000

H -3.44452200 1.30866600 1.85494300

C -3.90405200 -0.33309500 -0.83756900

C -2.76351100 -1.17127900 -0.23789300

H -3.90806900 -0.22875100 -1.92088800

O -3.55431500 0.94938600 -0.21451500

H -2.23802600 -1.17978100 1.94470700

H -2.59888100 -2.19844300 -0.54233300

Cl -1.19042200 0.26099600 -2.97516900

**M1-b**

P -0.44544900 -1.07693200 0.44390300

C 0.10985600 -1.92335900 1.98910800

C 0.90805900 -3.06831700 1.85978400

C -0.16284700 -1.44188800 3.27313500

C 1.44201900 -3.69520700 2.98082000

H 1.09300000 -3.49054700 0.87657600

C 0.36710500 -2.07427700 4.39588500

H -0.80479000 -0.57922100 3.40604000

C 1.17786000 -3.19574900 4.25367800

H 2.05768100 -4.58091500 2.85818900

H 0.13909700 -1.68716900 5.38429900

H 1.59208400 -3.68545000 5.12948200

C -0.91496700 -2.55713000 -0.54782000

C -0.25699400 -2.85346600 -1.74288600

C -1.89016300 -3.44088200 -0.07023800

C -0.59350400 -3.99964100 -2.46307800

H 0.48669600 -2.16566700 -2.12606000

C -2.22865500 -4.57949200 -0.79312300

H -2.39711300 -3.24403400 0.86830600

C -1.58304300 -4.85805500 -1.99682700

H -0.08575300 -4.20866600 -3.39930100

H -2.99319000 -5.25120300 -0.41421200

H -1.84950700 -5.74430900 -2.56441600

C -1.78666000 2.19174500 -0.26431600

H -1.67648400 1.71608500 -1.24812100

C -2.73174900 3.38694600 -0.43522700

H -2.89118000 3.95154200 0.48652700

H -2.35834900 4.07908300 -1.18870400

H -3.70663900 3.02542300 -0.78472000

P 0.02882900 2.48648900 0.12874400

C 0.14033400 3.07536400 1.93317800

C -0.93787000 4.07826600 2.36188600

H -1.92695300 3.60942600 2.34824200

H -0.74112500 4.39582500 3.39463000

H -0.96775600 4.97376400 1.73768800

C -0.00056800 1.83602200 2.82735800

H -1.00667800 1.41411100 2.76499100

H 0.72522300 1.06114800 2.55502200

H 0.17741200 2.12326200 3.87238200

C 1.53562200 3.65209500 2.20590100

H 1.70518100 4.61414300 1.71846200

H 1.64725800 3.80627900 3.28641700

H 2.32016500 2.95586400 1.88716900

C 0.59651400 3.90163600 -1.01065600

C 2.13517200 3.86142200 -1.07620500

H 2.48990700 2.89372900 -1.44233300

H 2.47403700 4.63047400 -1.78206600

H 2.61205100 4.06945700 -0.11556100

C 0.10719100 3.60621400 -2.43907300

H 0.42496800 2.61869100 -2.78143000

H -0.97935200 3.65204200 -2.54599900

H 0.53567500 4.36355900 -3.10782200

C 0.15867100 5.31672300 -0.60867800

H -0.92598400 5.42253200 -0.52932400

H 0.60106200 5.63945900 0.33771200

H 0.50235400 6.01678100 -1.38103600

Rh 1.08996000 0.20911300 -0.49019100

C -4.76025200 -1.87094900 -1.06460000

Fe -3.85763300 -0.23826100 -0.03424200

C -3.77692600 -1.27608400 -1.89881000

C -5.74366700 -0.88613900 -0.77050500

C -5.36449200 0.31705900 -1.42341300

C -4.14624300 0.07616900 -2.11619400

C -2.93221100 -0.78584100 1.82504600

C -3.93405600 0.16906500 2.08425900

C -3.59468400 1.35146300 1.37826900

C -2.38393500 1.14779200 0.66083900

C -1.96207500 -0.20859500 0.94062700

H -2.86835700 -1.73704300 -2.26486100

H -6.60321600 -1.01620400 -0.12672400

H -5.88545900 1.26341700 -1.35924700

H -3.55744200 0.79563700 -2.67107900

H -2.89326000 -1.78424800 2.23757400

H -4.82112300 0.01522900 2.68329500

H -4.19709700 2.24796700 1.34260000

H -4.74169200 -2.88459600 -0.68821100

C 5.89559800 -1.71264800 -1.70065300

C 7.13011500 -1.68495400 -1.03889200

C 7.33315300 -0.85768300 0.06202800

C 6.30813800 -0.03086400 0.53985800

C 5.09617200 -0.05518700 -0.12265600

H 5.74215400 -2.36010400 -2.55927300

H 7.94180800 -2.31269800 -1.39328300

H 8.30089700 -0.84899000 0.55395300

H 6.47229500 0.61183900 1.40015500

C 4.89213100 -0.88643700 -1.23214900

C 3.44594500 -0.66362500 -1.62296900

C 2.53718700 -1.25819700 -0.53556000

H 3.15182500 -0.82411300 -2.65962100

C 3.76485600 0.63365200 0.10004400

C 2.73093300 -0.38804800 0.60327600

H 3.77261300 1.61677000 0.57160900

O 3.28133700 0.74690800 -1.27331100

H 2.36476700 -2.32740400 -0.49006500

H 2.69699600 -0.70114700 1.64230600

Cl -0.39189700 0.20134000 -2.64571800

**M1-c**

P 0.51040200 -1.15920500 -0.07200500

C 0.03125000 -2.64960000 -1.03477900

C -0.48306600 -3.75643600 -0.34722100

C 0.03681700 -2.66301700 -2.43355400

C -0.95633000 -4.86510600 -1.04163100

H -0.50917000 -3.76125000 0.73914000

C -0.42903000 -3.77952800 -3.12314200

H 0.36747600 -1.78903600 -2.98247100

C -0.92323100 -4.88243700 -2.43326100

H -1.34966200 -5.71535300 -0.49261600

H -0.41650900 -3.77562400 -4.20859700

H -1.28874200 -5.74855400 -2.97674300

C 1.24171700 -1.95866700 1.41818900

C 0.86917300 -1.53122700 2.69097600

C 2.15631300 -3.01318300 1.29435400

C 1.42724500 -2.11925800 3.82915300

H 0.12863500 -0.74393400 2.79038100

C 2.70160900 -3.61007800 2.42369900

H 2.43387200 -3.36413900 0.30320900

C 2.34424900 -3.15577600 3.69573700

H 1.13377300 -1.77330400 4.81559100

H 3.41066500 -4.42585300 2.31761100

H 2.77627600 -3.61775400 4.57810300

C 1.76043700 1.94719000 0.21311600

H 1.84491400 1.43100800 1.18090500

C 2.70586000 3.15965000 0.25542300

H 2.71986700 3.69952700 -0.69147900

H 2.44796300 3.87119700 1.03430400

H 3.72510000 2.80515700 0.44009400

P -0.09792400 2.34060000 0.17732900

C -0.34214000 3.71153700 -1.12626900

C 0.04165000 5.12195800 -0.65430100

H 1.07935900 5.19302700 -0.31985300

H -0.07260200 5.79893300 -1.50978000

H -0.60928000 5.49494800 0.14058600

C 0.49783400 3.38170200 -2.37390900

H 1.56343700 3.56101700 -2.21768800

H 0.34911700 2.35554300 -2.71539400

H 0.17030800 4.05272300 -3.17796600

C -1.81978500 3.72815500 -1.54914000

H -2.48715500 3.94907000 -0.71012900

H -1.95173200 4.52145500 -2.29528400

H -2.10398700 2.77382700 -2.00039100

C -0.42748300 3.04736000 1.92607600

C -1.85407700 3.62410600 1.94457700

H -2.58803900 2.95417500 1.49532700

H -2.15094700 3.80041700 2.98618300

H -1.90864400 4.58382700 1.42368200

C -0.33857700 1.88196100 2.92413000

H -0.99533600 1.05756400 2.64968700

H 0.68405200 1.49516700 2.99941900

H -0.62049600 2.24913800 3.91916800

C 0.52038300 4.13011300 2.47475500

H 1.49366300 3.71334500 2.74720100

H 0.67128700 4.97545800 1.80365500

H 0.07593900 4.52067700 3.39901300

Rh -1.37035500 0.45583000 -0.21667600

C 5.11852400 -1.70426400 0.57988000

Fe 4.00145300 -0.20174300 -0.45351700

C 4.54396400 -0.82059900 1.53283300

C 5.98783800 -0.94993400 -0.25205800

C 5.94753900 0.40124200 0.18114700

C 5.05676000 0.47782700 1.28564300

C 2.71954000 -0.98966300 -1.97919900

C 3.52861600 0.03455500 -2.52126400

C 3.23925100 1.23620300 -1.82506600

C 2.24287500 0.96901500 -0.84420500

C 1.91412100 -0.42314600 -0.94999900

H 3.81682400 -1.08277000 2.29029900

H 6.54143300 -1.32725200 -1.10126900

H 6.46594100 1.23169100 -0.27905400

H 4.79653400 1.37269200 1.83494200

H 2.70746500 -2.02787800 -2.28284200

H 4.27211200 -0.08495600 -3.29732800

H 3.74274300 2.17995500 -1.98271200

H 4.90759600 -2.76017600 0.48361200

C -6.55745100 -0.41260800 -0.61427700

C -7.52113500 -0.91312300 0.27054200

C -7.14972400 -1.49578900 1.47932000

C -5.80125000 -1.59751900 1.84383500

C -4.85491200 -1.10922400 0.96314000

H -6.85259700 0.04034700 -1.55626600

H -8.57258400 -0.85205300 0.00728600

H -7.91475500 -1.88211000 2.14562700

H -5.51625200 -2.05392300 2.78766200

C -5.22800000 -0.52307600 -0.25341900

C -3.93157400 -0.06726200 -0.88670100

C -3.34312900 1.09496100 -0.06511600

H -3.85674800 0.01186100 -1.96968200

C -3.34654800 -0.98787600 1.00806600

C -2.94354200 0.48710600 1.17429600

H -2.78915900 -1.72630100 1.58533700

O -3.01198200 -1.10717600 -0.40865000

H -3.69820500 2.10943500 -0.19757200

H -2.97421700 0.96300200 2.14751500

Cl -1.05047800 0.40458900 -2.79126200

**M1-d**

P -0.63991900 -0.99972900 0.61802900

C -0.26301700 -1.76255800 2.25833600

C 0.81113800 -2.66455300 2.26891500

C -0.94815100 -1.52501700 3.45121600

C 1.20633600 -3.29052100 3.44424700

H 1.31908300 -2.89869300 1.33477200

C -0.54871900 -2.15126600 4.63334300

H -1.80369000 -0.85676700 3.46603200

C 0.52945900 -3.02824800 4.63574700

H 2.03697500 -3.98976300 3.43025900

H -1.09165600 -1.95280700 5.55251300

H 0.83626100 -3.51494600 5.55630600

C -1.06322800 -2.54648300 -0.29424300

C -0.67142900 -2.70700500 -1.62440300

C -1.79016300 -3.56603800 0.33653300

C -1.03107200 -3.86127900 -2.32234400

H -0.11191100 -1.92034500 -2.12328400

C -2.14709400 -4.71322300 -0.36315500

H -2.06030400 -3.47491200 1.38555200

C -1.77065200 -4.86061100 -1.69877700

H -0.72766800 -3.97133500 -3.35881500

H -2.71029500 -5.49630400 0.13557800

H -2.04738000 -5.75695600 -2.24566000

C -1.57456100 2.18610400 -0.29325800

H -1.33870100 1.71322400 -1.25866100

C -2.33465300 3.47885800 -0.60081400

H -2.56885000 4.05997900 0.29802700

H -1.77856400 4.11004500 -1.29129700

H -3.27940000 3.21858700 -1.09001500

P 0.19484600 2.35177200 0.36373200

C 0.20332300 2.74321000 2.23486400

C -0.05030100 4.21508600 2.58773800

H -1.04194800 4.54632100 2.26388400

H -0.01895400 4.30764700 3.68031200

H 0.69597200 4.89954400 2.17999200

C -0.86519800 1.92267200 2.96339200

H -1.88353700 2.21353600 2.69385500

H -0.74144800 0.85844500 2.77131100

H -0.73865700 2.07909300 4.04238300

C 1.55740500 2.30627700 2.81604900

H 2.40539500 2.82248300 2.36046400

H 1.56555200 2.53395400 3.88910700

H 1.69836900 1.22884200 2.68832700

C 0.95360700 3.86772400 -0.51157300

C 2.38243500 4.06089100 0.03543900

H 2.94301300 3.12894500 0.11534500

H 2.92648400 4.72512900 -0.64746400

H 2.38106700 4.53824700 1.01913100

C 1.03636100 3.54528900 -2.01350600

H 1.60993800 2.64398700 -2.22660500

H 0.04668500 3.38744100 -2.45378500

H 1.50231800 4.40085700 -2.51872500

C 0.20823500 5.22759200 -0.36449600

H -0.20980800 5.53927700 -1.32557100

H -0.60515000 5.23247600 0.35621100

H 0.91802700 6.00397900 -0.05788000

Rh 1.17954400 0.38281800 -0.31135000

C -4.05626200 -1.71801900 -1.90117800

Fe -3.82769100 -0.08265700 -0.55686100

C -3.28355700 -0.70119900 -2.52338200

C -5.33153800 -1.17314900 -1.58910100

C -5.34698500 0.18315600 -2.01403000

C -4.07921300 0.47138400 -2.59150000

C -3.39919400 -0.66381800 1.45226100

C -4.37001000 0.35947900 1.47374000

C -3.80077100 1.51385700 0.87403500

C -2.46676700 1.22133900 0.47120400

C -2.21581300 -0.14566200 0.84051100

H -2.24600600 -0.76371200 -2.82849800

H -6.13228300 -1.68334100 -1.07028900

H -6.16013200 0.88339000 -1.87763000

H -3.74943100 1.42763800 -2.97650100

H -3.53305600 -1.68009500 1.79821200

H -5.38643500 0.26741400 1.83154400

H -4.32284600 2.44527800 0.70820300

H -3.71062800 -2.71614200 -1.66249700

C 6.03030600 -1.65675000 0.41544400

C 7.17077400 -1.70288800 -0.39709100

C 7.10966800 -1.32878900 -1.73672500

C 5.90731500 -0.89591400 -2.31047200

C 4.78395400 -0.86079600 -1.50655200

H 6.08290700 -1.94944800 1.46028800

H 8.11352300 -2.04101800 0.02166700

H 8.00619400 -1.37760400 -2.34690300

H 5.86482300 -0.60521600 -3.35598800

C 4.84568200 -1.23664400 -0.15837800

C 3.44609600 -1.01340100 0.37508800

C 3.14054400 0.49109100 0.42940700

H 3.12001900 -1.61207900 1.22359500

C 3.35297800 -0.41935600 -1.72593200

C 3.10601300 0.88280400 -0.94473400

H 2.91365200 -0.48196500 -2.72018800

O 2.64477100 -1.32378100 -0.81204200

H 3.46516400 1.10497800 1.26031500

H 3.40720800 1.83623700 -1.35745400

Cl 0.10934300 0.40685900 -2.79656400

**TS_1-2_1A**

P 0.62898800 -1.15564900 -0.13855900

C 0.48377300 -2.75825000 -1.02535800

C 0.03455400 -3.87656800 -0.31025000

C 0.64775800 -2.86245500 -2.41087700

C -0.21399100 -5.08203500 -0.96023200

H -0.12269300 -3.81260000 0.76323600

C 0.41108200 -4.07422200 -3.05468400

H 0.92265300 -1.98981100 -2.99025400

C -0.01641700 -5.18570600 -2.33447100

H -0.55949200 -5.93855300 -0.38965400

H 0.54539100 -4.13946600 -4.12972900

H -0.20538000 -6.12631000 -2.84265800

C 1.27290600 -1.72184500 1.49297600

C 0.80375100 -1.14524100 2.67203300

C 2.25323100 -2.72055800 1.56981500

C 1.30982000 -1.54377200 3.91118500

H 0.03397900 -0.38355000 2.61846100

C 2.74703300 -3.13071600 2.80224300

H 2.62376400 -3.18064500 0.65712300

C 2.27832900 -2.53925600 3.97724400

H 0.93653000 -1.08264600 4.82050000

H 3.50240500 -3.90950300 2.85089100

H 2.66769000 -2.85934000 4.93865600

C 1.59676600 2.18040700 0.09331300

H 1.69025500 1.77607200 1.11288100

C 2.38115900 3.49951800 0.04873400

H 2.37110700 3.94259000 -0.94902200

H 1.98253300 4.23851900 0.74051600

H 3.42802500 3.31207300 0.31473500

P -0.28190200 2.27008900 -0.09863800

C -0.64229500 3.39157300 -1.58404000

C -0.47730900 4.89683900 -1.33941100

H 0.51859300 5.16736400 -0.97722400

H -0.63076300 5.41373600 -2.29521300

H -1.22117900 5.28756000 -0.63934500

C 0.30333800 2.96774700 -2.72142500

H 1.33290000 3.29155900 -2.54761200

H 0.28804000 1.88648300 -2.88229300

H -0.04229200 3.44680700 -3.64607300

C -2.08471100 3.12279400 -2.04906200

H -2.82887000 3.38097300 -1.29225600

H -2.27542100 3.74415100 -2.93393400

H -2.22621800 2.07520400 -2.32342900

C -0.88717100 3.11937700 1.49928800

C -2.39056700 3.39509900 1.31961800

H -2.92089000 2.54406700 0.87661100

H -2.83181700 3.60726800 2.30260900

H -2.56888100 4.27037300 0.68865200

C -0.73151900 2.10849600 2.64455700

H -1.28145600 1.18808400 2.43568500

H 0.31742700 1.84936100 2.82886400

H -1.12669000 2.54993100 3.56856500

C -0.19157400 4.40655300 1.96760400

H 0.80598900 4.19898800 2.36627500

H -0.10766200 5.17064400 1.19411400

H -0.77902800 4.83395500 2.79082700

Rh -1.36461100 -0.01225300 -0.26141500

C 5.14433400 -1.16011600 1.04342200

Fe 4.02515700 0.15532800 -0.21499900

C 4.40448900 -0.25598800 1.85340200

C 6.03354500 -0.40145500 0.23713500

C 5.84099500 0.97128100 0.54277000

C 4.83613700 1.05844400 1.54439300

C 2.96849600 -0.81309100 -1.80581900

C 3.75148800 0.24027300 -2.32648600

C 3.30240800 1.44809600 -1.73540600

C 2.23646100 1.16066200 -0.83688000

C 2.01335400 -0.25914900 -0.90099800

H 3.62209000 -0.51797500 2.55352600

H 6.70231500 -0.79566000 -0.51592300

H 6.33551500 1.80490100 0.06237700

H 4.44146600 1.97055800 1.97251100

H 3.07312400 -1.86340600 -2.03959000

H 4.57780400 0.13876500 -3.01653000

H 3.74702700 2.42014200 -1.89609400

H 5.02773100 -2.23447400 1.02117700

C -5.98482000 -0.29437000 2.11114500

C -7.26194600 -0.66195300 1.67686700

C -7.46157600 -1.11033500 0.37460300

C -6.39294700 -1.19195900 -0.52819000

C -5.13060300 -0.83247500 -0.09861600

H -5.82401900 0.04749600 3.13030100

H -8.10345200 -0.59083400 2.35866100

H -8.46124200 -1.38061700 0.04841800

H -6.55853700 -1.50922100 -1.55386100

C -4.92638900 -0.39814700 1.21896900

C -3.50706700 -0.17550400 1.54520000

C -2.55860600 -1.17186700 1.15157600

H -3.27841900 0.49456600 2.37441300

C -3.82100500 -0.69414300 -0.83744900

C -2.72720700 -1.58284200 -0.20788800

H -3.87827900 -0.80279800 -1.92447100

O -3.36097100 0.60127900 -0.45545200

H -1.88874700 -1.62785300 1.87402100

H -2.42601000 -2.54091400 -0.61848100

Cl -1.10570200 -0.21875600 -2.80523900

**TS_1-2_1B**

P 0.59516800 -1.16865200 -0.09368900

C 0.45264300 -2.80684600 -0.92020900

C 0.10398200 -3.93512000 -0.16429000

C 0.54004900 -2.92794000 -2.31210500

C -0.12465100 -5.16184500 -0.78225500

H 0.01091800 -3.86432800 0.91601400

C 0.32340100 -4.15994900 -2.92378000

H 0.74126100 -2.05334100 -2.91882700

C -0.00635300 -5.27885500 -2.16413600

H -0.39236300 -6.02423400 -0.17972600

H 0.39794100 -4.23615600 -4.00391200

H -0.17892600 -6.23586500 -2.64693900

C 1.24038500 -1.65014700 1.56148500

C 0.77168900 -1.00127100 2.70183400

C 2.22540800 -2.63797600 1.69605800

C 1.28316000 -1.31932300 3.96163600

H -0.00080300 -0.24915400 2.59442300

C 2.72154700 -2.97066100 2.95072400

H 2.59835200 -3.14828900 0.81142600

C 2.25364700 -2.30759100 4.08723500

H 0.91263900 -0.80144200 4.84118500

H 3.48064900 -3.74130600 3.04624300

H 2.64688200 -2.56484400 5.06591700

C 1.61344400 2.14704400 0.01857300

H 1.72885000 1.77978700 1.05032000

C 2.41776900 3.45046100 -0.10018200

H 2.39044700 3.85004100 -1.11554600

H 2.05532600 4.22648200 0.56873700

H 3.46710200 3.25153000 0.14552800

P -0.26954300 2.26466300 -0.12843600

C -0.63473900 3.32487500 -1.66131300

C -0.41571500 4.83372600 -1.48221400

H 0.59744400 5.08419800 -1.15673300

H -0.57537500 5.31408200 -2.45576700

H -1.12660600 5.27982300 -0.78220800

C 0.27439700 2.83319400 -2.80133900

H 1.31468500 3.13891300 -2.66316300

H 0.22829500 1.74806700 -2.92495500

H -0.08180900 3.28675700 -3.73474400

C -2.09373900 3.08344600 -2.08791600

H -2.81602700 3.38754900 -1.32790600

H -2.28197700 3.67990800 -2.99019700

H -2.26685300 2.03276700 -2.33099400

C -0.82186300 3.21202900 1.43381800

C -2.29907300 3.60154000 1.24329700

H -2.88528200 2.79476900 0.79268200

H -2.72642800 3.84733100 2.22455700

H -2.40391200 4.48900600 0.61337400

C -0.73468600 2.23919700 2.61866300

H -1.36719900 1.36191400 2.46399200

H 0.29374400 1.90154300 2.79397000

H -1.06910200 2.75601400 3.52740000

C -0.03138300 4.46332100 1.84724800

H 0.95472100 4.20508900 2.24547200

H 0.09068200 5.19157800 1.04415000

H -0.58174400 4.95779400 2.65826000

Rh -1.36509900 0.02907700 -0.21615100

C 5.12474300 -1.18984300 1.02721500

Fe 4.01152800 0.08626800 -0.27859600

C 4.42943400 -0.22110100 1.80109500

C 6.01432000 -0.50322100 0.15903200

C 5.86709400 0.88943000 0.39123100

C 4.89003300 1.06088500 1.40910700

C 2.90580300 -0.92070400 -1.81654000

C 3.68999700 0.10496400 -2.38743100

C 3.27053200 1.33663100 -1.82508600

C 2.22096400 1.09192400 -0.89383700

C 1.97950200 -0.32444800 -0.90902800

H 3.65786800 -0.42030200 2.53343200

H 6.65345800 -0.95769200 -0.58583100

H 6.37389600 1.68124600 -0.14411000

H 4.53114400 2.00560600 1.79583300

H 2.98849900 -1.97900400 -2.02134600

H 4.49767900 -0.02935900 -3.09368900

H 3.72504400 2.29595200 -2.02792400

H 4.98196500 -2.26023100 1.06906900

C -6.14320800 -0.03138600 1.83578300

C -7.33890200 -0.62010000 1.39954800

C -7.37493100 -1.38849200 0.24026600

C -6.21153800 -1.59775100 -0.50656100

C -5.02661100 -1.03496600 -0.05352700

H -6.13133400 0.60082500 2.71930200

H -8.25383400 -0.45340200 1.95998200

H -8.31336400 -1.81949000 -0.09342700

H -6.23088200 -2.20037300 -1.41009400

C -4.99169200 -0.24426800 1.10529200

C -3.60781400 0.35579700 1.21807900

C -2.52149900 -0.72162200 1.38493300

H -3.54725000 1.15136600 1.97357300

C -3.70034100 -1.27373500 -0.63373700

C -2.62762100 -1.60457800 0.25833300

H -3.59350300 -1.50971400 -1.69303700

O -3.33510600 0.82283200 -0.09656800

H -2.11115000 -1.00951100 2.34811500

H -2.07263700 -2.52345000 0.09839400

Cl -1.22532100 -0.29182500 -2.82797500

**TS_1-2_2A**

P 0.64781300 -1.12827300 -0.02939100

C 0.22603400 -2.49332600 -1.19647000

C -0.05147400 -3.77669300 -0.71532800

C 0.04080600 -2.23194900 -2.55986500

C -0.48883300 -4.77983700 -1.57885200

H 0.08462600 -4.00057400 0.33965500

C -0.38804000 -3.23577600 -3.42270800

H 0.22697400 -1.23401100 -2.94830900

C -0.65376000 -4.51365400 -2.93428700

H -0.69606100 -5.77077900 -1.18696900

H -0.51998800 -3.01722400 -4.47793900

H -0.99084000 -5.29574900 -3.60726800

C 1.62588500 -2.02244400 1.24132400

C 1.43990100 -1.73722800 2.59390100

C 2.59051000 -2.96472800 0.85536300

C 2.21995200 -2.39042600 3.55295200

H 0.67823100 -1.02275200 2.90108400

C 3.35570400 -3.61681500 1.81346500

H 2.74309300 -3.18696300 -0.19842600

C 3.17249800 -3.32643400 3.16782200

H 2.06707700 -2.16413000 4.60354700

H 4.09789100 -4.34866200 1.50766400

H 3.77285700 -3.83444600 3.91656600

C 1.55742900 2.18313000 0.07311600

H 1.68766500 1.72568200 1.06458200

C 2.36327200 3.49044100 0.06143500

H 2.21487000 4.06945000 -0.85152400

H 2.11329100 4.13015300 0.90560400

H 3.43028200 3.24839600 0.13441700

P -0.32669700 2.25038000 -0.03485600

C -0.78288400 2.83509600 -1.78330100

C 0.09514600 3.94849400 -2.36684900

H 1.10655300 3.58499200 -2.57402600

H -0.33705900 4.26889600 -3.32399300

H 0.16008300 4.82751700 -1.72203400

C -0.67914600 1.62629300 -2.73134300

H 0.34930100 1.26015900 -2.81777300

H -1.31360500 0.80095300 -2.39662400

H -1.00745700 1.93851900 -3.73120000

C -2.25151300 3.29178700 -1.76166800

H -2.36583200 4.28802800 -1.32819700

H -2.62382900 3.33773000 -2.79310900

H -2.88233100 2.60520700 -1.19039700

C -0.81442700 3.56754900 1.24709000

C -2.29433200 3.37400800 1.62586100

H -2.46982000 2.38051100 2.04524100

H -2.54152000 4.12197700 2.39045300

H -2.97554700 3.51654500 0.78502700

C 0.00033300 3.31053700 2.52940000

H -0.11014300 2.27892000 2.87485900

H 1.06129100 3.55091400 2.42595700

H -0.40255700 3.96155400 3.31475600

C -0.60431000 5.02366600 0.80464300

H 0.42386000 5.24566600 0.50950700

H -1.26582700 5.31266000 -0.01550200

H -0.84703100 5.67086800 1.65674200

Rh -1.31460700 0.04367000 0.46239300

C 5.37190100 -1.13202600 0.27376800

Fe 3.97512400 0.21034700 -0.62074200

C 4.67698500 -0.43558700 1.29783200

C 6.06535400 -0.17659100 -0.51662900

C 5.79397700 1.11124200 0.01478300

C 4.93539200 0.94961300 1.13651500

C 2.68844100 -0.72459200 -2.05918700

C 3.37974100 0.34637700 -2.66910600

C 3.02649000 1.53902800 -1.98365000

C 2.11175900 1.21494700 -0.94285100

C 1.88352800 -0.19879000 -1.00053900

H 4.02804800 -0.87807900 2.04165400

H 6.65290200 -0.38842700 -1.39966900

H 6.14224400 2.05223900 -0.39039400

H 4.52329700 1.74403000 1.74482500

H 2.75097700 -1.76674900 -2.34263600

H 4.09363100 0.26450300 -3.47725100

H 3.43698900 2.52046500 -2.17911400

H 5.34754600 -2.19975800 0.10662700

C -6.15494200 -1.54972200 1.25484500

C -7.35274600 -1.44375700 0.54051500

C -7.37410500 -0.84349100 -0.71403800

C -6.20191200 -0.32594600 -1.28364100

C -5.01684600 -0.44206600 -0.58612500

H -6.13065600 -2.01790100 2.23484700

H -8.27291700 -1.82252900 0.97350600

H -8.31493500 -0.75425200 -1.24864800

H -6.23269900 0.17801000 -2.24579500

C -4.99371500 -1.06083800 0.67208500

C -3.64140100 -1.20908900 1.22286000

C -2.60192900 -1.65951500 0.34466600

H -3.48916800 -1.27796800 2.29997100

C -3.64304500 0.12773600 -0.85478800

C -2.56483100 -0.96299900 -0.90751800

H -3.61972900 0.79103400 -1.72972800

O -3.29942800 0.78552300 0.35629500

H -2.01532300 -2.53101600 0.61472600

H -2.18584100 -1.38111400 -1.83236800

Cl -1.32948100 0.15022700 3.14876700

**TS_1-2_2B**

P 0.65100300 -1.16237500 0.14011100

C 0.22133200 -2.84549300 -0.46450900

C 0.01163200 -3.83229800 0.50990000

C -0.08885600 -3.13740800 -1.79622900

C -0.46631500 -5.08823900 0.15079200

H 0.20459200 -3.60725700 1.55570500

C -0.56388500 -4.39817500 -2.15346400

H 0.03323000 -2.38061400 -2.56523200

C -0.74876600 -5.37745500 -1.18296400

H -0.62318600 -5.84014800 0.91760100

H -0.79299000 -4.60979400 -3.19331700

H -1.12026300 -6.35832500 -1.46246400

C 1.90035200 -1.56436400 1.42449300

C 1.93616600 -0.87551000 2.63447100

C 2.85421800 -2.55878700 1.16766100

C 2.92525000 -1.16645800 3.57587800

H 1.16090100 -0.15592800 2.86007600

C 3.82626300 -2.85999400 2.11406500

H 2.83042700 -3.10176000 0.22554200

C 3.86537700 -2.15898800 3.32096100

H 2.93834300 -0.62848600 4.51854600

H 4.55627900 -3.63836100 1.91218200

H 4.62532100 -2.39339300 4.06025200

C 1.52823300 2.15469800 -0.32043200

H 2.02784900 1.83366500 0.60672900

C 2.20968300 3.44649600 -0.79228500

H 1.82769500 3.76810600 -1.76401000

H 2.06854800 4.26812800 -0.09412600

H 3.28562300 3.27402900 -0.90873200

P -0.26503200 2.27816300 0.29104800

C -1.20000800 3.31415900 -1.00610900

C -0.83376200 4.79987900 -1.13436700

H 0.22471300 4.98129900 -1.32365800

H -1.39938900 5.22015600 -1.97685500

H -1.12851100 5.35974600 -0.24264900

C -0.91826400 2.61540300 -2.34924400

H 0.10657100 2.78236900 -2.69347500

H -1.06652300 1.53004300 -2.28716200

H -1.59547700 3.01577000 -3.11406600

C -2.71579800 3.26490400 -0.71841400

H -3.00972800 4.04931500 -0.01757200

H -3.25813800 3.45748000 -1.65425800

H -3.06041000 2.31828000 -0.30198200

C -0.15557900 3.26018400 1.91124500

C -1.60479200 3.55023400 2.33835500

H -2.25156800 2.67140400 2.22623800

H -1.60712500 3.83701700 3.39688800

H -2.03323300 4.38323500 1.77330600

C 0.46715500 2.34181000 2.97136100

H -0.13941500 1.44745600 3.13975800

H 1.49064500 2.04704000 2.70933700

H 0.52605000 2.89673100 3.91657000

C 0.65180800 4.56734600 1.92361700

H 1.72814200 4.37067400 1.90139900

H 0.40453700 5.25467800 1.11298600

H 0.44472600 5.08397900 2.86957100

Rh -1.34734500 -0.04917400 0.52713900

C 5.28680900 -1.06700500 -0.54876700

Fe 3.71775900 0.12294900 -1.37626400

C 4.88831700 -0.09595100 0.40892700

C 5.76130500 -0.38236200 -1.69906100

C 5.65085100 1.01198500 -1.45658500

C 5.11292000 1.18607500 -0.15244400

C 2.17547900 -1.00472600 -2.33499800

C 2.64984900 -0.00448300 -3.21356000

C 2.40998800 1.25423700 -2.60651600

C 1.78035500 1.05316800 -1.34254300

C 1.60827100 -0.36688400 -1.18881200

H 4.45009000 -0.29439200 1.37843000

H 6.09832900 -0.84101400 -2.61889800

H 5.88951300 1.80073100 -2.15760300

H 4.88585800 2.13117200 0.32280600

H 2.24792600 -2.07296600 -2.48588800

H 3.15715700 -0.16994900 -4.15421800

H 2.72614700 2.20422800 -3.01354600

H 5.21072700 -2.13909000 -0.43341500

C -6.38994400 -1.26182000 0.45683200

C -7.43832800 -1.04047800 -0.44755100

C -7.20993100 -0.39989200 -1.66120600

C -5.92298600 0.02580500 -2.00592300

C -4.88613700 -0.21662900 -1.11629100

H -6.57838300 -1.72925500 1.41907300

H -8.44516500 -1.35316500 -0.18798900

H -8.03588700 -0.22193900 -2.34247100

H -5.73927800 0.51954000 -2.95656100

C -5.11871900 -0.84580500 0.11501100

C -3.82717800 -0.82103000 0.89876500

C -2.70686300 -1.59372000 0.17340200

H -3.90915400 -1.09074400 1.95476300

C -3.46015200 0.05913300 -1.36411000

C -2.50821200 -0.97180100 -1.09866900

H -3.21116700 0.84610000 -2.07638600

O -3.36198200 0.51661000 0.72332200

H -2.41285400 -2.60513000 0.43037900

H -1.81266000 -1.28985700 -1.86581400

Cl -1.32865700 -0.73546400 2.99104500

**TS_1-2_3A**

P 0.31951100 -1.05905700 0.22059800

C -0.01612600 -2.77690900 -0.29263600

C 0.47686200 -3.87326500 0.42318900

C -0.75309300 -2.97980300 -1.46634900

C 0.23434100 -5.16669900 -0.02987600

H 1.05096600 -3.71866800 1.33255700

C -0.98393000 -4.27810800 -1.91429600

H -1.12868700 -2.12467400 -2.02254700

C -0.49691200 -5.36887400 -1.19833500

H 0.61839000 -6.01504700 0.52855700

H -1.55308700 -4.43381200 -2.82534700

H -0.68631700 -6.37860600 -1.55069500

C 0.83870500 -1.22838600 1.96927200

C -0.13733800 -1.59288500 2.90460100

C 2.12926100 -0.94939400 2.41520000

C 0.17722200 -1.68210600 4.25544700

H -1.15094100 -1.80081600 2.56930800

C 2.44544900 -1.02972400 3.77158900

H 2.88638100 -0.66430500 1.69424900

C 1.47064100 -1.39509100 4.69386900

H -0.58834600 -1.96866000 4.97001100

H 3.45592600 -0.80730000 4.10319100

H 1.71313700 -1.45679400 5.75016800

C 1.93442500 1.83705200 0.22473500

H 1.97635900 1.35896900 1.21285900

C 3.01160600 2.93112900 0.20690000

H 3.09207100 3.41425200 -0.76731300

H 2.84475700 3.70952100 0.94885000

H 3.98019100 2.46151700 0.41767100

P 0.10039400 2.35618100 0.16621000

C -0.04353000 3.76461900 -1.11026400

C 0.44815100 5.13667300 -0.63079800

H 1.49500300 5.11967500 -0.31569400

H 0.37301900 5.83453300 -1.47416500

H -0.15543100 5.54725100 0.18330500

C 0.75196500 3.38539900 -2.37226100

H 1.82801700 3.49963900 -2.22851300

H 0.53444900 2.36943100 -2.71104100

H 0.45800700 4.07846300 -3.17080800

C -1.51781300 3.88291700 -1.52896400

H -2.17396800 4.13359400 -0.68931000

H -1.60419200 4.69108900 -2.26563100

H -1.85837600 2.95426100 -1.99850500

C -0.18208700 3.03970800 1.92814500

C -1.55615100 3.72568500 1.97742300

H -2.35781300 3.06094600 1.64673900

H -1.77192300 4.00730200 3.01548800

H -1.59251100 4.63911400 1.37765200

C -0.21174600 1.84561900 2.89654000

H -0.91047400 1.07328800 2.57597200

H 0.77252200 1.37969700 3.01292000

H -0.51611200 2.20732000 3.88711600

C 0.87231800 4.01233000 2.48051700

H 1.82031100 3.50313300 2.67443400

H 1.05967600 4.87566400 1.84102000

H 0.51180200 4.38874200 3.44613700

Rh -1.39290900 0.48100300 -0.30009300

C 4.82816000 -1.94548500 0.59878100

Fe 3.89687500 -0.65022700 -0.83485100

C 5.23281600 -0.60390500 0.84314400

C 5.24870100 -2.29736600 -0.70874700

C 5.91391300 -1.17658500 -1.27350000

C 5.90413900 -0.13046400 -0.31488400

C 2.17491200 -1.21696800 -1.96729800

C 2.96000500 -0.30592000 -2.70706100

C 3.01236600 0.91739000 -1.98871100

C 2.23957700 0.77850900 -0.80578600

C 1.73681000 -0.56003900 -0.78111300

H 5.05129700 -0.03340300 1.74518400

H 5.05176800 -3.23736500 -1.20558500

H 6.31000400 -1.11260700 -2.27780700

H 6.30283000 0.86507100 -0.45643600

H 1.94130500 -2.23774300 -2.23798800

H 3.47459400 -0.51817500 -3.63398100

H 3.57795400 1.79205100 -2.28083000

H 4.25991000 -2.57438100 1.27126300

C -6.43565400 -0.28504500 -1.21108800

C -7.41791000 -1.08389300 -0.61617100

C -7.12106100 -1.85206200 0.50519100

C -5.83107000 -1.85018600 1.05259500

C -4.86294500 -1.05918100 0.46711300

H -6.66780900 0.32058600 -2.08265900

H -8.41732800 -1.11047900 -1.03872800

H -7.89124100 -2.47675700 0.94721400

H -5.59237600 -2.47869200 1.90625600

C -5.16614700 -0.27227800 -0.65197200

C -4.05121800 0.60585200 -1.05597800

C -3.37955000 1.33890800 -0.03235900

H -3.98519700 0.94520900 -2.08969900

C -3.38264300 -0.92643300 0.73134300

C -2.98060900 0.52008200 1.08504400

H -2.99537800 -1.67755800 1.43257700

O -2.79418900 -1.03510500 -0.55774600

H -3.26908300 2.41207100 -0.11508000

H -2.90305100 0.87149900 2.10889400

Cl -0.84369300 0.37575400 -2.81907300

**TS_1-2_3B**

P 0.34585200 -1.07702300 0.17167100

C 0.13885400 -2.79693900 -0.40824700

C 0.89943200 -3.83747200 0.13765400

C -0.73110400 -3.04820500 -1.47369600

C 0.78709000 -5.12585000 -0.37455400

H 1.57893000 -3.63714000 0.96254300

C -0.83476100 -4.34226500 -1.98212100

H -1.31432800 -2.23397500 -1.89512200

C -0.08180000 -5.37759300 -1.43566500

H 1.37591400 -5.93174200 0.05321200

H -1.51084300 -4.53789100 -2.80851800

H -0.17100700 -6.38345800 -1.83530000

C 0.79357200 -1.29845800 1.93637700

C -0.19532800 -1.83302100 2.77292500

C 2.00178400 -0.88711100 2.49753800

C 0.02370600 -1.95086400 4.14018000

H -1.13654500 -2.16831200 2.34031100

C 2.22069800 -0.99879300 3.87133400

H 2.77585500 -0.48511600 1.85312100

C 1.23233000 -1.52690600 4.69507400

H -0.74743400 -2.37316800 4.77733300

H 3.16764000 -0.67385200 4.29354200

H 1.40060700 -1.61385000 5.76392400

C 1.89829900 1.87863500 0.26183300

H 1.89170700 1.40754800 1.25426400

C 2.96704600 2.98122500 0.29133900

H 3.09004100 3.45917900 -0.68129200

H 2.76022900 3.76211300 1.01976000

H 3.92733500 2.51798800 0.55002300

P 0.06169700 2.37932600 0.10380400

C -0.01838600 3.76292700 -1.19854000

C 0.47578300 5.13659200 -0.72463600

H 1.50640100 5.11138300 -0.36183300

H 0.45042400 5.81754800 -1.58463500

H -0.15679300 5.57437700 0.05121100

C 0.82265600 3.34960800 -2.41990800

H 1.89316600 3.45873500 -2.23662600

H 0.61019100 2.32739000 -2.74201800

H 0.56516000 4.02556000 -3.24521400

C -1.47279400 3.88819500 -1.67917300

H -2.16678600 4.13436200 -0.86950300

H -1.52334200 4.69964700 -2.41596600

H -1.79357200 2.96117900 -2.16386600

C -0.31398800 3.09391000 1.83438600

C -1.64087200 3.87310300 1.77729900

H -2.40605400 3.38263700 1.17271600

H -2.03430900 3.98702900 2.79524600

H -1.50119000 4.87745000 1.36929200

C -0.45423500 1.90152900 2.80269200

H -1.18425200 1.16048900 2.47233300

H 0.49657400 1.37619500 2.94321900

H -0.76197000 2.28246500 3.78493500

C 0.74126400 4.01811600 2.46198600

H 1.65283700 3.47094200 2.71843400

H 1.00299700 4.87161200 1.83374400

H 0.33192000 4.41325200 3.40063600

Rh -1.46021000 0.48493400 -0.32668900

C 4.78648200 -1.84216100 0.83924800

Fe 3.94489600 -0.58758300 -0.68160200

C 5.18695100 -0.49613800 1.06309700

C 5.27350100 -2.23789800 -0.43252900

C 5.97712300 -1.13940500 -0.99514200

C 5.92384600 -0.06339800 -0.07124700

C 2.30289200 -1.16789600 -1.91886900

C 3.12051100 -0.24552900 -2.60743300

C 3.11763700 0.97389500 -1.88129500

C 2.27442200 0.82217700 -0.74729000

C 1.79058800 -0.52239200 -0.75650000

H 4.95999300 0.10310500 1.93570100

H 5.09593800 -3.19187000 -0.90983300

H 6.42612100 -1.10887900 -1.97854200

H 6.33647400 0.92505400 -0.22246000

H 2.09642300 -2.19058900 -2.20405400

H 3.69170600 -0.44851000 -3.50289800

H 3.69030800 1.85494600 -2.13774100

H 4.17995300 -2.44509600 1.50184300

C -6.34875800 -0.88615500 -1.29417000

C -7.35505400 -1.49921200 -0.53589900

C -7.14473100 -1.81954900 0.80197800

C -5.92476300 -1.52379100 1.41723900

C -4.93816600 -0.89786300 0.66707700

H -6.50514400 -0.66962100 -2.34713900

H -8.30337200 -1.74599400 -1.00356400

H -7.92992200 -2.30866200 1.36959500

H -5.76023100 -1.76362800 2.46445300

C -5.14424800 -0.59096500 -0.68644600

C -3.84714200 -0.05516100 -1.24481300

C -3.41856100 1.24451600 -0.53434900

H -3.79699500 0.01445400 -2.33574200

C -3.65611200 -0.39733800 1.18852500

C -3.26137600 0.93344800 0.85223400

H -3.24480800 -0.86070000 2.08630200

O -2.87176000 -0.95786100 -0.72592700

H -3.60986500 2.23321700 -0.93441800

H -3.03233800 1.63109700 1.64491000

Cl -0.83567600 0.34219400 -2.78490200

**TS_1-2_4A**

P -0.38606400 -0.95543900 0.64262800

C 0.21895700 -1.51214900 2.29452600

C 1.46734000 -2.15384400 2.28404000

C -0.44346600 -1.34611800 3.51140700

C 2.04336900 -2.60171500 3.46620300

H 1.97726800 -2.30900700 1.33316500

C 0.14016700 -1.79219500 4.69912500

H -1.42072600 -0.87399000 3.54343700

C 1.38161100 -2.41586200 4.68143500

H 3.00626800 -3.10364700 3.43944700

H -0.38607000 -1.65359800 5.63875500

H 1.83022100 -2.76519100 5.60624200

C -0.55618000 -2.60230300 -0.16082200

C -0.18685000 -2.77555700 -1.49574200

C -1.07016600 -3.68780600 0.56074500

C -0.37259200 -4.01432500 -2.11118300

H 0.23580400 -1.94329600 -2.04984500

C -1.25188700 -4.92040400 -0.05705000

H -1.30772300 -3.57766200 1.61576000

C -0.90924100 -5.08254600 -1.39975500

H -0.08955000 -4.13874300 -3.15178200

H -1.65170000 -5.75581300 0.50998900

H -1.05011500 -6.04493200 -1.88272500

C -1.95263400 1.97837900 -0.34378500

H -1.69585000 1.51655500 -1.30833900

C -2.94912000 3.10406100 -0.62778900

H -3.22147300 3.66589200 0.27325400

H -2.55895600 3.80059600 -1.36834800

H -3.86431000 2.66990700 -1.04436300

P -0.20758000 2.47508900 0.18941800

C -0.18032700 2.98438000 2.03739900

C -0.58357700 4.43520200 2.33093400

H -1.62911700 4.62741100 2.07184900

H -0.48143200 4.60269000 3.41053400

H 0.04120600 5.17301800 1.82414000

C -1.11624500 2.09042200 2.85740800

H -2.16873300 2.24186500 2.60427300

H -0.87573800 1.03556400 2.72972500

H -0.98104900 2.33394100 3.91915100

C 1.24024500 2.74464700 2.57496900

H 1.99258400 3.35779500 2.07029600

H 1.26264500 3.00660300 3.64001000

H 1.52224500 1.69013000 2.47754200

C 0.22913700 4.03965700 -0.81272700

C 1.65638300 4.46568700 -0.42071900

H 2.37335000 3.64518800 -0.49450800

H 1.98304100 5.25151000 -1.11279300

H 1.70623600 4.88286500 0.58865900

C 0.25971400 3.64886300 -2.29963300

H 0.96946500 2.84700400 -2.50558400

H -0.71299000 3.30086200 -2.66026500

H 0.54172500 4.53719400 -2.87885100

C -0.70324700 5.27610100 -0.65481000

H -1.49977500 5.16165500 0.07577800

H -0.11795000 6.15342800 -0.35879800

H -1.17747600 5.51406200 -1.61184200

Rh 1.13984500 0.58386100 -0.42246600

C -3.72439500 -2.40872000 -1.64432800

Fe -3.74822000 -0.69176200 -0.38500000

C -3.21569900 -1.29263200 -2.36081100

C -5.06078300 -2.11443700 -1.26134700

C -5.37937900 -0.81387300 -1.73735300

C -4.23685400 -0.30869300 -2.41725400

C -3.10951300 -1.07930900 1.61386200

C -4.25394300 -0.25591800 1.65355600

C -3.94511400 0.95221400 0.97449200

C -2.60266200 0.89573300 0.50371000

C -2.07772300 -0.38130300 0.91154200

H -2.20714900 -1.16997600 -2.73650900

H -5.70564300 -2.74604100 -0.66473100

H -6.31000400 -0.28733000 -1.57263400

H -4.13935800 0.67059000 -2.86776900

H -3.03025000 -2.08444700 2.00583600

H -5.21492200 -0.51816000 2.07484900

H -4.64323000 1.75905700 0.80349700

H -3.16720000 -3.30127300 -1.38925400

C 5.88845900 -1.19052700 0.51525800

C 6.94441600 -1.61894200 -0.29515400

C 6.84481200 -1.54332400 -1.68103300

C 5.68426100 -1.04956100 -2.29243200

C 4.64217000 -0.62330600 -1.49316600

H 5.96627800 -1.23825200 1.59835000

H 7.84493200 -2.01869400 0.15996100

H 7.66906400 -1.88963300 -2.29700800

H 5.59768800 -1.02519400 -3.37496100

C 4.75045500 -0.68182600 -0.09495700

C 3.60568500 -0.06820600 0.60053100

C 3.14870600 1.20806200 0.13161200

H 3.39622800 -0.36172300 1.62944900

C 3.23970900 -0.16677400 -1.82730700

C 2.97063100 1.24836100 -1.27735700

H 2.93944100 -0.28492600 -2.87285400

O 2.43327600 -0.95011500 -0.95132200

H 3.09285300 2.05102300 0.80754000

H 3.00546800 2.13778900 -1.89483800

Cl -0.05259800 0.38576300 -2.75408700

**TS_1-2_4B**

P -0.38349400 -0.97417200 0.63741100

C 0.09311700 -1.69737400 2.26417100

C 1.23141500 -2.51857500 2.24758900

C -0.56854900 -1.48442800 3.47543100

C 1.70680400 -3.09095900 3.42099400

H 1.73310300 -2.70919400 1.30040800

C -0.09074000 -2.06496200 4.65106700

H -1.46742400 -0.87713800 3.50988400

C 1.04718200 -2.86327900 4.62883200

H 2.58787400 -3.72495000 3.39188100

H -0.61805100 -1.89373900 5.58466800

H 1.41471300 -3.31474400 5.54520100

C -0.47841900 -2.54123700 -0.32991300

C 0.24295900 -2.71873000 -1.51187100

C -1.27468500 -3.58908400 0.14916100

C 0.12398200 -3.91173400 -2.22619600

H 0.87818300 -1.92614500 -1.88277200

C -1.39465700 -4.77363500 -0.56781500

H -1.79407200 -3.49118100 1.09761400

C -0.70082200 -4.93245800 -1.76706700

H 0.67908500 -4.03268000 -3.15111800

H -2.02267400 -5.57399300 -0.18746200

H -0.79454500 -5.85562300 -2.33123600

C -1.91951700 1.96958400 -0.38846300

H -1.60710900 1.49351900 -1.32793800

C -2.91958100 3.06791200 -0.75441300

H -3.23778200 3.66786200 0.10532000

H -2.51324100 3.73339000 -1.51288100

H -3.80953200 2.59758800 -1.18719300

P -0.21373600 2.47836300 0.24338100

C -0.31238800 2.98124300 2.08527000

C -0.91312700 4.37009500 2.33599200

H -1.93810000 4.44427500 1.95778500

H -0.95366800 4.53426900 3.41992600

H -0.32411100 5.18177400 1.90386700

C -1.15473500 1.97396000 2.87653900

H -2.21247100 1.99054200 2.60624300

H -0.77131400 0.96126900 2.74718300

H -1.06924000 2.22356700 3.94204000

C 1.09509600 2.90751800 2.70145700

H 1.83655300 3.52412200 2.19035900

H 1.03664000 3.25134500 3.74169800

H 1.44494000 1.87135700 2.70729400

C 0.27717700 4.05067100 -0.72538300

C 1.60517500 4.57584900 -0.14217400

H 2.32935700 3.78895900 0.07604800

H 2.05750400 5.26087200 -0.86973400

H 1.44725400 5.13958200 0.78118800

C 0.50222200 3.63586500 -2.18957300

H 1.25942900 2.85912900 -2.30817100

H -0.41024100 3.23656300 -2.64269400

H 0.80824400 4.52560300 -2.75473200

C -0.71840300 5.24518300 -0.73879000

H -1.24604200 5.30123000 -1.69380700

H -1.46880200 5.22607300 0.04820000

H -0.15648300 6.18061900 -0.63430400

Rh 1.15533200 0.58939900 -0.29856400

C -3.16519500 -1.66979400 -2.26430000

Fe -3.73789600 -0.66640700 -0.47281600

C -3.73142400 -0.40864100 -2.58181200

C -4.14879700 -2.43447600 -1.58277300

C -5.32828200 -1.64596900 -1.48248300

C -5.07029800 -0.39340900 -2.10254100

C -3.14021100 -1.07101800 1.54463800

C -4.27586600 -0.23556000 1.56324700

C -3.94180000 0.96696100 0.88857900

C -2.59257500 0.89296400 0.44145500

C -2.08582400 -0.39086400 0.85506600

H -3.19886900 0.40336800 -3.06040500

H -4.00413300 -3.42368200 -1.17013700

H -6.24393600 -1.92984500 -0.98132400

H -5.75740800 0.44092000 -2.15463000

H -3.08164100 -2.06365900 1.96822700

H -5.24463200 -0.48616800 1.97353800

H -4.62533500 1.78331000 0.70461700

H -2.13884400 -1.95258300 -2.45745800

C 5.90304400 -1.40885500 0.42881000

C 7.00826600 -1.58232400 -0.41628300

C 6.97337600 -1.14177200 -1.73551000

C 5.83514500 -0.50535500 -2.24004400

C 4.75433900 -0.31439200 -1.39042500

H 5.92259300 -1.78781500 1.44693700

H 7.89479000 -2.08610900 -0.04321600

H 7.83130000 -1.30084900 -2.38092200

H 5.80504200 -0.15389900 -3.26765700

C 4.78108400 -0.77472000 -0.06579900

C 3.41016500 -0.55761800 0.53054200

C 3.03131800 0.92927300 0.58552000

H 3.26045300 -1.06789600 1.48892800

C 3.54496500 0.45601400 -1.71086800

C 3.09301500 1.42377400 -0.76306600

H 3.21405000 0.53258800 -2.74743900

O 2.52086800 -1.02103500 -0.48595500

H 3.16867000 1.53791500 1.47149200

H 2.93550700 2.43971100 -1.09601500

Cl -0.05014400 0.32798500 -2.67316200

**M2-1A**

P -0.83302600 1.15158600 -0.13644100

C -1.22719000 2.75582000 -0.94981100

C -1.11930900 3.94760100 -0.21804400

C -1.50344000 2.83369100 -2.32040600

C -1.30258000 5.18219600 -0.83352800

H -0.89965700 3.92096100 0.84566800

C -1.69585900 4.07133300 -2.92987600

H -1.53119100 1.93101300 -2.91743100

C -1.59751900 5.24709400 -2.19229500

H -1.21655400 6.09156500 -0.24693300

H -1.90980000 4.10995300 -3.99322800

H -1.74303300 6.20920400 -2.67358300

C -1.43967300 1.43107900 1.58146700

C -0.78238200 0.81315800 2.64486100

C -2.58160400 2.19871300 1.84825500

C -1.23698600 0.96805200 3.95541800

H 0.09187300 0.20689600 2.44185100

C -3.02944800 2.36596200 3.15374700

H -3.11563800 2.67315400 1.02949200

C -2.35520000 1.75414900 4.21219500

H -0.71050400 0.47971200 4.76977200

H -3.91120600 2.96898600 3.34879900

H -2.70816900 1.88695200 5.23004500

C -0.81338600 -2.24754600 0.13481200

H -0.87610000 -1.80654100 1.14088800

C -1.23273400 -3.71951000 0.24305700

H -1.19693100 -4.22226100 -0.72541300

H -0.60363100 -4.28011600 0.93074200

H -2.26598500 -3.77360100 0.60241500

P 0.99700100 -1.91519100 -0.31225100

C 1.38617500 -2.94120200 -1.87424200

C 1.65467000 -4.42996300 -1.61048000

H 0.84168100 -4.93385800 -1.08131200

H 1.76340700 -4.91878400 -2.58654200

H 2.58756700 -4.59124000 -1.06443700

C 0.19880500 -2.84063800 -2.84673400

H -0.65549100 -3.43916000 -2.52052400

H -0.12109400 -1.80952400 -3.00866200

H 0.52987500 -3.24212200 -3.81253200

C 2.62414200 -2.35764100 -2.57453600

H 3.50744500 -2.34381000 -1.93355300

H 2.83761000 -2.99447400 -3.44333900

H 2.45043700 -1.33757000 -2.91646400

C 2.01826400 -2.59782300 1.15288700

C 3.49982100 -2.42871800 0.76292200

H 3.69874900 -1.46701100 0.27747600

H 4.11141800 -2.49391800 1.67194600

H 3.82548300 -3.22725200 0.08907600

C 1.74231700 -1.72852400 2.38532200

H 1.98140700 -0.68404000 2.19666300

H 0.70194300 -1.81495400 2.72127200

H 2.37947900 -2.07860600 3.20713700

C 1.78350300 -4.04608100 1.62310400

H 0.87322400 -4.13179500 2.22341000

H 1.74719200 -4.78574200 0.82643200

H 2.61968000 -4.31309600 2.28168200

Rh 1.42863700 0.49969200 -0.59037400

C -5.06515100 0.03701000 1.33858300

Fe -3.76776900 -0.99451400 -0.01050000

C -4.12342700 -0.72041800 2.08623100

C -5.82316000 -0.86368900 0.54483000

C -5.34850200 -2.17740600 0.79673800

C -4.30092000 -2.08686100 1.75285300

C -3.10222000 0.17997400 -1.67119700

C -3.59415400 -1.06269800 -2.13000800

C -2.80113700 -2.08625400 -1.55266400

C -1.79927500 -1.49090000 -0.73632400

C -1.98249400 -0.07051200 -0.82412300

H -3.37861700 -0.32425400 2.76327300

H -6.59131500 -0.59239900 -0.16679300

H -5.69518900 -3.08218000 0.31570500

H -3.71878400 -2.91265000 2.13967600

H -3.50948500 1.15452300 -1.90182500

H -4.45797200 -1.20972400 -2.76379600

H -2.96966500 -3.14701500 -1.67584100

H -5.16705400 1.11305000 1.34673600

C 4.29463300 0.74371200 2.67913700

C 5.66105700 0.50120300 2.81030500

C 6.50218600 0.62937700 1.70805000

C 5.97365800 0.98366700 0.46561200

C 4.61291600 1.21738300 0.32658000

H 3.64012500 0.66984100 3.54515400

H 6.06971000 0.22387900 3.77733500

H 7.56701600 0.44516800 1.81261700

H 6.62013000 1.05515500 -0.40569200

C 3.76424100 1.10825800 1.44157100

C 2.34526600 1.46802000 1.25312300

C 2.06089700 2.46232300 0.25791800

H 1.74020600 1.49304600 2.15588900

C 3.91222600 1.33877600 -1.00678500

C 2.73558800 2.33378700 -0.94239200

H 4.61384300 1.61508700 -1.80699000

O 3.32654300 0.09643800 -1.24335100

H 1.24700900 3.17153500 0.37141500

H 2.47509000 2.93799500 -1.80552400

Cl 0.67983200 0.56459300 -2.98658500

**M2-1B**

P 0.45315900 -1.17545700 0.06564500

C 0.12588200 -2.86000400 -0.59152400

C -0.12383900 -3.91276800 0.29977300

C -0.06047100 -3.07823800 -1.96277700

C -0.53040800 -5.15930400 -0.16874600

H -0.00417800 -3.76749400 1.36944100

C -0.45887600 -4.32913800 -2.42615000

H 0.07050400 -2.26198600 -2.66247000

C -0.69504600 -5.37186000 -1.53439400

H -0.71944400 -5.96103100 0.53821200

H -0.59887600 -4.48024100 -3.49180600

H -1.01367700 -6.34272800 -1.90088400

C 1.32232200 -1.55413100 1.64022400

C 1.00184200 -0.83946800 2.79316900

C 2.31226800 -2.54341900 1.70415600

C 1.67648700 -1.08334200 3.99094800

H 0.21487900 -0.09336600 2.74890000

C 2.97038700 -2.80330500 2.90009600

H 2.55939500 -3.11363500 0.81210400

C 2.65886900 -2.06620200 4.04500900

H 1.42174600 -0.51336200 4.87913100

H 3.73385300 -3.57458300 2.94201600

H 3.17931400 -2.26586700 4.97647700

C 1.61072800 2.07020300 -0.15485100

H 1.85177600 1.70939900 0.85731800

C 2.48313600 3.30647600 -0.42531100

H 2.31543600 3.71075400 -1.42462000

H 2.30492200 4.10719200 0.28670600

H 3.53923400 3.02334700 -0.36181000

P -0.26215200 2.35098500 -0.01423300

C -0.76965300 3.40233700 -1.51827900

C -0.44046100 4.89712100 -1.39681100

H 0.62040500 5.08970000 -1.21707000

H -0.70451600 5.37300700 -2.34951800

H -1.02356100 5.39265100 -0.61687400

C -0.04939200 2.85930800 -2.76511200

H 1.00987800 3.12810400 -2.78244700

H -0.14619200 1.77670400 -2.86232200

H -0.51765600 3.31730000 -3.64520300

C -2.28448600 3.25605800 -1.73822600

H -2.86750700 3.60330400 -0.88334600

H -2.55260200 3.86488500 -2.61152900

H -2.56659300 2.21951700 -1.93164000

C -0.45762200 3.40062700 1.57594900

C -1.92629400 3.85463500 1.65439700

H -2.60993400 3.04374000 1.38524200

H -2.13347900 4.19013400 2.67892700

H -2.12461300 4.70028500 0.98971000

C -0.18120000 2.49167600 2.78019000

H -0.90218000 1.67682200 2.83247100

H 0.83083100 2.07086500 2.76320000

H -0.27199400 3.08660600 3.69773100

C 0.45083600 4.62808100 1.77130400

H 1.46929000 4.33723600 2.04449000

H 0.49252900 5.29854600 0.91373600

H 0.04975900 5.20176100 2.61668800

Rh -1.50747200 0.15521100 0.13729300

C 5.08302300 -1.40800400 0.36948800

Fe 3.83501100 -0.11856000 -0.79495700

C 4.63840200 -0.33117100 1.18443100

C 5.83273700 -0.86470000 -0.70669200

C 5.84868700 0.54704600 -0.56244100

C 5.11392800 0.87360800 0.60906900

C 2.41826800 -1.11998400 -2.05272500

C 3.12352900 -0.15573900 -2.80492800

C 2.86894400 1.11534700 -2.23135100

C 2.00058300 0.95780400 -1.11489100

C 1.70614700 -0.44409500 -1.01708300

H 4.01880500 -0.40846100 2.06842600

H 6.27097000 -1.42373900 -1.52242400

H 6.29944600 1.25181900 -1.24813100

H 4.92591500 1.86947000 0.98685200

H 2.41951600 -2.18834100 -2.21763100

H 3.78092800 -0.35654100 -3.63967200

H 3.31302100 2.04409600 -2.56124300

H 4.87094100 -2.45623200 0.52499100

C -6.15893500 -0.01165800 0.62967200

C -6.95751500 -0.83201100 -0.16838800

C -6.38840000 -1.89222700 -0.87049500

C -5.02563600 -2.15584400 -0.75603900

C -4.23225800 -1.35662100 0.06558800

H -6.59120500 0.84292200 1.14449300

H -8.02116000 -0.63225000 -0.25647600

H -7.00842100 -2.51875800 -1.50411700

H -4.57559800 -2.99009200 -1.28799700

C -4.79967600 -0.26496700 0.74125200

C -3.80512300 0.69502200 1.34518800

C -2.66529300 -0.04383700 2.08268000

H -4.30004300 1.41547200 2.01637500

C -2.82927700 -1.68277600 0.34971300

C -2.30081300 -1.30742200 1.61124000

H -2.41353400 -2.54487100 -0.16341900

O -3.19457100 1.33345600 0.26177600

H -2.31415700 0.30656900 3.04777300

H -1.52624600 -1.89123600 2.10477100

Cl -1.52214700 -0.19815300 -2.32447800

**M2-2A**

P 0.76347800 -1.18697400 -0.04973700

C 0.58688200 -2.50556900 -1.33003600

C 0.60408500 -3.86515900 -1.00002200

C 0.29508200 -2.13653900 -2.64887700

C 0.34528400 -4.83188000 -1.96987200

H 0.81338900 -4.17439400 0.01971900

C 0.03884500 -3.10306600 -3.61666700

H 0.27161300 -1.08378800 -2.91918600

C 0.06350600 -4.45455000 -3.27936400

H 0.36382000 -5.88236500 -1.69677000

H -0.18249800 -2.79908200 -4.63514400

H -0.13711000 -5.20879000 -4.03366900

C 1.78676600 -2.03663200 1.21632800

C 1.46623200 -1.91118200 2.56820500

C 2.90964200 -2.78720800 0.83525100

C 2.27105100 -2.52845800 3.53078200

H 0.59173900 -1.33719900 2.86739200

C 3.69635800 -3.40991100 1.79486300

H 3.16828900 -2.88082500 -0.21697800

C 3.37865300 -3.27651400 3.14899500

H 2.01834600 -2.42276600 4.58113500

H 4.56087000 -3.99372800 1.49246000

H 3.99777300 -3.75773000 3.90014600

C 1.22179400 2.18982300 0.19624200

H 1.29232100 1.68643200 1.17186600

C 1.92075000 3.55519100 0.32718100

H 1.82345200 4.16783500 -0.56956200

H 1.55193900 4.13524900 1.16855100

H 2.98905100 3.37739200 0.49199700

P -0.65205800 2.13480800 -0.07625400

C -0.99598200 2.78268000 -1.82974500

C -0.10490400 3.95705500 -2.26131900

H 0.93372600 3.63958600 -2.39026600

H -0.46186400 4.30758200 -3.23786100

H -0.13835100 4.80600800 -1.57684800

C -0.73461200 1.63949500 -2.82337500

H 0.28479100 1.24715100 -2.74784400

H -1.44008500 0.82355700 -2.67122600

H -0.87046400 2.02947000 -3.83989500

C -2.47312800 3.18890800 -1.96034000

H -2.68064800 4.15278700 -1.49045400

H -2.70900200 3.28941600 -3.02736800

H -3.13011800 2.44247700 -1.51119900

C -1.30857800 3.37570200 1.23003500

C -2.79646300 3.12206600 1.51858600

H -2.95747500 2.12427100 1.93053200

H -3.11294200 3.86778700 2.25918100

H -3.42168600 3.22652800 0.63094600

C -0.55880600 3.16899700 2.56092200

H -0.60891400 2.13200200 2.89551700

H 0.48314800 3.49176000 2.53639800

H -1.06573900 3.78261700 3.31528900

C -1.15057300 4.84744400 0.80698500

H -0.12453600 5.13010900 0.56595200

H -1.78999200 5.11533100 -0.03627300

H -1.46619500 5.46307900 1.65791300

Rh -1.42753500 -0.07322600 0.31328000

C 5.45092300 -0.59689100 0.52057500

Fe 3.91634000 0.57418900 -0.39339000

C 4.59640800 -0.08626500 1.53274900

C 6.03060300 0.50533900 -0.16439500

C 5.52856400 1.69733800 0.42092700

C 4.64144100 1.32949500 1.46896800

C 2.85049700 -0.46085600 -1.94470600

C 3.43549800 0.71187600 -2.47232800

C 2.89090600 1.82244100 -1.77541900

C 1.96117900 1.34330600 -0.80913800

C 1.92656100 -0.08290700 -0.92117600

H 3.98537600 -0.67118900 2.20712400

H 6.69682800 0.44857000 -1.01462500

H 5.74600200 2.70582700 0.09440600

H 4.07360400 2.00630500 2.09403200

H 3.05903100 -1.47450200 -2.25989900

H 4.20104700 0.75520200 -3.23501700

H 3.18689200 2.85344300 -1.91414600

H 5.60626200 -1.64231600 0.29252800

C -5.67986200 -2.10167000 1.38129300

C -6.96893100 -1.61759300 1.19306200

C -7.21874000 -0.67607200 0.19443100

C -6.17603200 -0.19540100 -0.59665300

C -4.88411600 -0.66894400 -0.41236300

H -5.47712900 -2.84757300 2.14544600

H -7.78025000 -1.97523100 1.81888800

H -8.22755300 -0.30391300 0.04297600

H -6.36297700 0.56817500 -1.34749300

C -4.63594100 -1.64595200 0.56989300

C -3.32424700 -2.26263200 0.60684000

C -2.41835600 -2.09090100 -0.40958100

H -3.13965500 -3.01886400 1.36612200

C -3.67628200 -0.00944200 -1.02737200

C -2.48857600 -0.94551900 -1.28678100

H -3.95411500 0.55115900 -1.93616800

O -3.21815100 0.84488600 -0.00717100

H -1.62102200 -2.81422000 -0.51873200

H -2.06751000 -1.03747800 -2.28288900

Cl -1.41786200 -0.07695200 2.89054600

**M2-2B**

P -0.98847700 1.13827900 0.05226200

C -0.73039000 2.68507000 -0.90404400

C -0.75089100 3.90665600 -0.21968500

C -0.38022600 2.67001400 -2.25812800

C -0.43626400 5.08754400 -0.88544800

H -0.98721400 3.92869900 0.83907900

C -0.06459700 3.85392900 -2.91990400

H -0.35904600 1.73367800 -2.80684100

C -0.09240600 5.06491900 -2.23488200

H -0.45257700 6.02656800 -0.34160700

H 0.20523400 3.82580400 -3.97103300

H 0.15641000 5.98754900 -2.74992900

C -2.34335800 1.61048700 1.19072900

C -2.24564300 1.31908900 2.55138000

C -3.48910800 2.24853700 0.70206200

C -3.30259400 1.62511400 3.40990900

H -1.32728500 0.89498000 2.94507400

C -4.53538800 2.56577600 1.56032500

H -3.56083900 2.49623000 -0.35466300

C -4.44694100 2.24276800 2.91531500

H -3.21901700 1.39700100 4.46773000

H -5.42073000 3.06373400 1.17599500

H -5.26596200 2.48716400 3.58471000

C -0.94758700 -2.31100900 -0.15088200

H -1.47517600 -2.05985400 0.78154400

C -1.28344700 -3.77291300 -0.48338900

H -0.86957100 -4.07006000 -1.44949600

H -0.91257600 -4.47049500 0.26323700

H -2.37241900 -3.88351900 -0.54554100

P 0.83582600 -1.92193100 0.35241200

C 1.99046000 -2.88855600 -0.78043700

C 1.96526100 -4.41736000 -0.63424200

H 0.99792400 -4.86707500 -0.86331400

H 2.69628700 -4.82870200 -1.34211900

H 2.27333800 -4.73725900 0.36476500

C 1.56660700 -2.51769300 -2.21240300

H 0.57124700 -2.89701100 -2.46289500

H 1.56964100 -1.43603300 -2.36555700

H 2.28200400 -2.95963500 -2.91643800

C 3.43646000 -2.41468200 -0.51988000

H 3.92550200 -3.05500200 0.21916100

H 4.01418000 -2.48035400 -1.44973800

H 3.48878400 -1.39273900 -0.14303700

C 0.92937400 -2.58386200 2.13390700

C 2.40455700 -2.68337500 2.55256600

H 2.96613900 -1.77803600 2.30006400

H 2.44371400 -2.82629500 3.63983000

H 2.88960400 -3.54981300 2.09343100

C 0.24132800 -1.53621700 3.03012900

H 0.80260500 -0.59205200 3.08207900

H -0.78988700 -1.32978800 2.71858500

H 0.19679900 -1.92273000 4.05612300

C 0.23997200 -3.92897400 2.39760300

H -0.84778000 -3.86485400 2.28659200

H 0.61363000 -4.73432700 1.76003300

H 0.44232400 -4.21402100 3.43742300

Rh 1.07211500 0.42457900 0.93276400

C -5.47565800 -0.20345500 -0.46962800

Fe -3.63189400 -1.00681800 -1.18536100

C -4.77012900 -0.78527400 0.61731400

C -5.73649900 -1.22400700 -1.42293900

C -5.18690500 -2.43604300 -0.92919900

C -4.59034600 -2.16298700 0.33223800

C -2.48461600 0.39347400 -2.31984100

C -2.72055000 -0.75912200 -3.09997200

C -2.13332400 -1.86459500 -2.43183900

C -1.52406800 -1.40810000 -1.22735600

C -1.72992000 0.01217200 -1.16667600

H -4.40084900 -0.26075400 1.48922300

H -6.22126900 -1.09141500 -2.38069300

H -5.18245300 -3.38770900 -1.44392300

H -4.06203600 -2.87489600 0.95364400

H -2.81065000 1.39903800 -2.55031500

H -3.29032500 -0.80202400 -4.01808500

H -2.20240600 -2.89177100 -2.76076400

H -5.73589900 0.84125800 -0.56776800

C 5.97587400 0.88599400 0.74181100

C 7.19727100 0.50847500 0.18667700

C 7.26238200 0.12010500 -1.14967400

C 6.10518300 0.10907400 -1.92151700

C 4.87753000 0.48646900 -1.36871600

H 5.91473500 1.18651100 1.78482800

H 8.09685000 0.51955600 0.79459500

H 8.21117600 -0.17303000 -1.58871300

H 6.14689700 -0.19448700 -2.96528800

C 4.81426900 0.88240400 -0.02334700

C 3.49002200 1.22598300 0.62912000

C 2.32555000 1.43828300 -0.34413300

H 3.61328900 2.13159200 1.23994900

C 3.65023900 0.45120900 -2.16656300

C 2.46412200 0.86809200 -1.69110700

H 3.72911600 0.07763800 -3.18651900

O 3.03516700 0.15307900 1.43489900

H 1.88388900 2.43743100 -0.34004300

H 1.58955100 0.85583000 -2.33666400

Cl 1.00141200 2.45625600 2.28396800

**M2-3A**

P 0.37176100 -1.09530300 0.21393000

C 0.38505400 -2.84129300 -0.31871200

C 1.29971100 -3.73049300 0.25386900

C -0.40247200 -3.24375300 -1.40289400

C 1.42405500 -5.02321000 -0.25011200

H 1.91639500 -3.41753500 1.09239500

C -0.26683900 -4.53608600 -1.90182500

H -1.10514000 -2.54526600 -1.84558500

C 0.64073000 -5.42520300 -1.32888700

H 2.13220800 -5.71256600 0.19940700

H -0.87800800 -4.84742400 -2.74290900

H 0.73834200 -6.43204500 -1.72390600

C 0.90571700 -1.18223300 1.95981100

C 0.08360000 -1.90290600 2.83661600

C 2.03655900 -0.53898300 2.45982400

C 0.38782300 -1.96633600 4.19111500

H -0.78958100 -2.42275700 2.44544400

C 2.33873100 -0.59945400 3.82107700

H 2.69185600 -0.00307900 1.78099000

C 1.51379700 -1.30789500 4.68802200

H -0.25149900 -2.53311800 4.86084200

H 3.22400400 -0.09569200 4.19876000

H 1.74830300 -1.35543100 5.74677100

C 1.65401600 2.10018500 0.01005800

H 1.80910000 1.74257900 1.03685200

C 2.59917700 3.29391200 -0.20409800

H 2.53807900 3.68670400 -1.21947000

H 2.41504700 4.11971500 0.47763500

H 3.62835900 2.94686600 -0.04907200

P -0.22853300 2.39737200 0.02807800

C -0.60698700 3.59303000 -1.40265900

C -0.23003800 5.05834400 -1.14698700

H 0.83405000 5.18538900 -0.93125600

H -0.44710000 5.62653100 -2.06033100

H -0.80618800 5.51479400 -0.33954900

C 0.14195600 3.13145900 -2.66642100

H 1.20359800 3.38512600 -2.62922700

H 0.03114200 2.06183700 -2.85276700

H -0.28776800 3.66792600 -3.52184900

C -2.11300000 3.50193300 -1.69899700

H -2.72447100 3.83964400 -0.85625100

H -2.34026000 4.15635500 -2.54964600

H -2.39482200 2.47921300 -1.96880100

C -0.45745400 3.30896500 1.69999000

C -1.82389100 4.02581500 1.73995500

H -2.61717800 3.50949500 1.19904000

H -2.14360100 4.13102000 2.78440800

H -1.75692500 5.03308300 1.32179500

C -0.39006400 2.22838500 2.79698700

H -1.12174100 1.43213300 2.66885800

H 0.59600500 1.75256500 2.83584700

H -0.56026800 2.70168800 3.77206500

C 0.59799100 4.35553200 2.08897600

H 1.56871900 3.89610400 2.29400900

H 0.72384200 5.14309700 1.34313100

H 0.26833100 4.83422100 3.02017600

Rh -1.55333400 0.10110400 -0.06063300

C 4.82895700 -1.25727000 0.75123100

Fe 3.81752500 -0.25592600 -0.84849800

C 5.11060900 0.13529400 0.80963400

C 5.29941500 -1.74774600 -0.49337500

C 5.87531800 -0.66072800 -1.20445700

C 5.76046700 0.50194200 -0.39900600

C 2.19258500 -1.07748100 -1.96720200

C 2.88668700 -0.14199400 -2.75997200

C 2.79935200 1.12270700 -2.12409500

C 2.03113400 0.98607400 -0.93679000

C 1.66992800 -0.39421400 -0.82599700

H 4.86640400 0.80559500 1.62430700

H 5.18927200 -2.75973900 -0.85830500

H 6.28054300 -0.70158700 -2.20625700

H 6.07298200 1.49978400 -0.67633000

H 2.07806800 -2.13304000 -2.17030600

H 3.43375900 -0.35992100 -3.66674500

H 3.27358900 2.02956600 -2.47309700

H 4.31048600 -1.83309100 1.50588200

C -5.94076800 -0.01390000 -1.05025300

C -6.82729400 -1.06984200 -1.23329500

C -6.61503900 -2.28242000 -0.57926900

C -5.49448900 -2.44830900 0.23332300

C -4.59663600 -1.40384300 0.41075700

H -6.11479500 0.94149600 -1.53880000

H -7.68995000 -0.94491500 -1.88054700

H -7.31257600 -3.10281800 -0.71726100

H -5.30109200 -3.40577800 0.71121300

C -4.83213500 -0.16605500 -0.21434600

C -3.97840500 0.96290400 0.14707000

C -3.23013900 0.90503500 1.31609700

H -4.20066800 1.92948500 -0.29745800

C -3.23553300 -1.59102500 1.03275500

C -2.70809500 -0.33574400 1.74492800

H -3.21923900 -2.47507200 1.68982300

O -2.35578200 -1.74567600 -0.04939600

H -2.93750400 1.80754400 1.83238100

H -2.21151800 -0.42208800 2.70725700

Cl -1.25311300 0.01432800 -2.53610400

**M2-3B**

P 0.04156200 -1.01457700 -0.09621900

C -0.22653100 -2.67593400 -0.80089500

C 0.00724500 -3.81999400 -0.03161100

C -0.56668300 -2.79905400 -2.15414800

C -0.09894100 -5.08264400 -0.60902800

H 0.27416000 -3.73256000 1.01735000

C -0.66551100 -4.06615400 -2.72147300

H -0.75119700 -1.90783700 -2.74604400

C -0.43607600 -5.20590200 -1.95396700

H 0.08386100 -5.96711800 -0.00659200

H -0.93089600 -4.15952200 -3.76986400

H -0.52118200 -6.19066800 -2.40389400

C 0.02540500 -1.30141700 1.70234600

C -1.20577500 -1.68253600 2.25115500

C 1.12823900 -1.12769000 2.53496600

C -1.32664500 -1.87659900 3.62177200

H -2.06782100 -1.82178900 1.60135200

C 1.00422400 -1.32264500 3.91115300

H 2.08163200 -0.83796500 2.10597100

C -0.22211900 -1.69085200 4.45544200

H -2.28567800 -2.17257600 4.03686700

H 1.86796300 -1.18370400 4.55539100

H -0.31862900 -1.83986200 5.52651500

C 1.92135300 1.75661200 0.47883400

H 1.74745400 1.25092200 1.43831700

C 3.08056700 2.74282900 0.68959600

H 3.38294700 3.22667000 -0.23942100

H 2.85136800 3.52728400 1.40640400

H 3.94669200 2.18193200 1.06193700

P 0.18470300 2.45124800 0.13394700

C 0.38853400 3.90251600 -1.08333400

C 0.89054200 5.21678000 -0.47250100

H 1.86528400 5.10389000 0.01009100

H 1.00975900 5.94356500 -1.28573800

H 0.19075400 5.64870400 0.24762500

C 1.36577400 3.49624700 -2.20054500

H 2.40424800 3.51695900 -1.86375600

H 1.14182100 2.51100300 -2.61405700

H 1.27320300 4.23217000 -3.00929800

C -0.98305900 4.14506100 -1.73449300

H -1.74552000 4.43542000 -1.00342300

H -0.88919900 4.96722200 -2.45452000

H -1.31735600 3.25453100 -2.27718900

C -0.29054700 3.11782900 1.86918000

C -1.59308900 3.92547100 1.75445300

H -2.39544900 3.36762000 1.27308500

H -1.93568700 4.18928800 2.76248400

H -1.45005900 4.85941500 1.20448900

C -0.51966100 1.90163500 2.78787200

H -1.09829500 1.10293700 2.32397100

H 0.42787900 1.46394300 3.11653300

H -1.04820300 2.23480000 3.69017600

C 0.73129800 3.99959900 2.60815200

H 1.60928800 3.42721800 2.91561200

H 1.05620500 4.87429700 2.04404900

H 0.25455200 4.35950200 3.52898900

Rh -1.42590700 0.59705100 -0.81411400

C 4.14914900 -2.23768000 1.43273700

Fe 3.76503100 -0.88979400 -0.18833300

C 4.62617800 -0.94091700 1.77071100

C 4.84255800 -2.67201300 0.27497000

C 5.75211600 -1.64830900 -0.10287300

C 5.61969800 -0.57982700 0.82127800

C 2.35337700 -1.25966300 -1.74770200

C 3.38262500 -0.42494100 -2.22832300

C 3.36713800 0.77505500 -1.47047700

C 2.31497500 0.69856500 -0.52109700

C 1.69164100 -0.57970500 -0.68078500

H 4.28837900 -0.32680000 2.59623600

H 4.67068200 -3.59899000 -0.25446700

H 6.39262100 -1.65729600 -0.97414400

H 6.15098200 0.36165600 0.78316800

H 2.10243800 -2.24645900 -2.11066600

H 4.09138100 -0.67417400 -3.00610000

H 4.07295300 1.58800100 -1.57368000

H 3.36281700 -2.78178300 1.93874700

C -5.48995300 -1.78033300 -0.35249700

C -5.92900800 -2.24624500 0.88815000

C -5.40397900 -1.70622400 2.06000100

C -4.45248000 -0.68886900 1.99708200

C -4.01547400 -0.21641600 0.75890900

H -5.87828200 -2.21385200 -1.27081400

H -6.67413700 -3.03418600 0.94023800

H -5.74708100 -2.06714300 3.02513000

H -4.05420500 -0.24974600 2.91000500

C -4.53584800 -0.77503700 -0.41959100

C -3.82242900 -0.34288200 -1.67991900

C -3.63744100 1.18214500 -1.70313500

H -4.34653100 -0.68921300 -2.58152500

C -3.07673100 0.91756600 0.61470400

C -3.35720500 1.79560200 -0.51344500

H -2.82396700 1.39744000 1.55418300

O -2.54312200 -0.89473300 -1.60739900

H -3.72875600 1.73889000 -2.63084100

H -3.20943900 2.86900500 -0.46495500

Cl -0.28720300 0.62681700 -3.05050600

**M2-4A**

P 0.06114100 -0.94718500 0.42067700

C 1.06070700 -1.33749800 1.91031200

C 2.29405400 -1.96148400 1.68314100

C 0.67268200 -1.04561300 3.22055300

C 3.11451500 -2.30291400 2.75283400

H 2.60765800 -2.17676500 0.66315100

C 1.50602700 -1.37550900 4.28888900

H -0.28829500 -0.58137600 3.41652500

C 2.72282900 -2.00998900 4.05818600

H 4.06719100 -2.78684600 2.55989600

H 1.19380000 -1.14597900 5.30313900

H 3.36362000 -2.27717900 4.89287300

C -0.02305900 -2.60392200 -0.36959800

C -0.02389900 -2.73897000 -1.75984200

C -0.12732900 -3.74618100 0.43620400

C -0.16414200 -4.00436300 -2.33063500

H 0.09598200 -1.86260800 -2.38686400

C -0.26847400 -5.00400600 -0.14007200

H -0.07448400 -3.65950400 1.51796000

C -0.29282200 -5.13370100 -1.52837200

H -0.16585200 -4.10082400 -3.41182200

H -0.34776200 -5.88178600 0.49404000

H -0.40096900 -6.11524200 -1.98022500

C -2.25585300 1.70721000 -0.03159400

H -2.14284500 1.32078900 -1.05265900

C -3.50156900 2.59827200 -0.01547000

H -3.65919300 3.09972500 0.94645300

H -3.45869100 3.35678900 -0.79609900

H -4.38053600 1.97721300 -0.22034600

P -0.56581200 2.53494400 0.16196800

C -0.34442900 3.08699300 1.99751200

C -0.54232600 4.58064900 2.28430900

H -1.57489100 4.90155000 2.13473700

H -0.29605700 4.75155500 3.33950300

H 0.11495000 5.22037100 1.69154400

C -1.29422800 2.33004600 2.93469200

H -2.34437500 2.55964800 2.73330100

H -1.16922500 1.24839800 2.87412300

H -1.07341000 2.63750800 3.96494700

C 1.09101000 2.72321600 2.39316500

H 1.82788100 3.22360100 1.75302200

H 1.27569100 3.03878700 3.42760300

H 1.25278900 1.64284400 2.33213300

C -0.68241000 4.11363700 -0.92561200

C 0.71839600 4.73452300 -1.06020700

H 1.35124400 4.12357200 -1.70707300

H 0.62130800 5.71902000 -1.53334900

H 1.22462900 4.87808600 -0.09858900

C -1.09713500 3.69952300 -2.34843700

H -0.41648000 2.96402300 -2.77761600

H -2.10262600 3.27400900 -2.40010800

H -1.08281200 4.60001800 -2.97541000

C -1.64483500 5.22850400 -0.43747000

H -2.47876900 4.87825300 0.16778700

H -1.11834700 5.99067700 0.13925200

H -2.06679000 5.72724700 -1.31762100

Rh 1.02808700 0.72924700 -0.85460500

C -3.44574400 -2.99632800 -1.05902000

Fe -3.44531200 -1.28414100 0.20274500

C -3.27895200 -1.82862800 -1.85048700

C -4.69432200 -2.89647500 -0.38762200

C -5.29811600 -1.66531200 -0.75962600

C -4.42022300 -1.00617600 -1.66421700

C -2.26705700 -1.53467100 1.96715600

C -3.50357900 -0.96260500 2.32681200

C -3.61826700 0.28056000 1.65335300

C -2.45164800 0.50126200 0.86879700

C -1.59890800 -0.64039200 1.06941400

H -2.40667600 -1.56341500 -2.43313500

H -5.09171500 -3.60606500 0.32588100

H -6.23591700 -1.27853200 -0.38365300

H -4.56799000 -0.02899800 -2.10518500

H -1.89231000 -2.49773500 2.28616800

H -4.25571600 -1.41457700 2.95900000

H -4.47870200 0.93202600 1.70235400

H -2.71778000 -3.78937200 -0.94394600

C 4.94861600 -0.34254800 0.90445000

C 6.01874000 -1.17045700 0.56591500

C 6.21732000 -1.55213500 -0.75861300

C 5.33880500 -1.11349500 -1.75141600

C 4.27202500 -0.29263100 -1.41647000

H 4.79773400 -0.03287600 1.93575500

H 6.70489800 -1.50819700 1.33715700

H 7.05346300 -2.19363700 -1.01959000

H 5.47614700 -1.42387000 -2.78418900

C 4.06997000 0.09834300 -0.08328900

C 3.00723700 1.09160700 0.18354000

C 2.83175400 2.07066300 -0.85367400

H 2.94161000 1.42045900 1.21356000

C 3.13625900 0.08541500 -2.33344800

C 2.76075500 1.57605500 -2.14176300

H 3.36169800 -0.12778300 -3.38693900

O 2.04477700 -0.66654900 -1.88706700

H 2.62360800 3.10539600 -0.61274300

H 2.54250200 2.20972000 -2.99601700

Cl -0.66690600 0.46450500 -2.71762900

**M2-4B**

P -0.29770700 -1.04774500 0.47696000

C 0.15887000 -1.89488800 2.04595300

C 0.99030900 -3.01967900 1.95234300

C -0.25882500 -1.46515700 3.30874800

C 1.40263400 -3.68765200 3.10006700

H 1.31997000 -3.35685900 0.97329600

C 0.15541200 -2.13872000 4.45668500

H -0.92683500 -0.61714400 3.40404400

C 0.98870500 -3.24782700 4.35591200

H 2.04704700 -4.55692800 3.01187400

H -0.18299000 -1.79531400 5.42929400

H 1.30855000 -3.77304100 5.25062000

C -0.63332400 -2.51084900 -0.58534100

C 0.03114100 -2.74218200 -1.79090200

C -1.58082000 -3.43739200 -0.13339800

C -0.29534700 -3.86370900 -2.55442800

H 0.78942300 -2.05103800 -2.13107000

C -1.89967700 -4.55454500 -0.89676500

H -2.07489400 -3.29718600 0.82237000

C -1.26426100 -4.76219000 -2.11979000

H 0.21638200 -4.02711900 -3.49741600

H -2.63936700 -5.26224100 -0.53421300

H -1.51610200 -5.62931700 -2.72278200

C -1.74544300 2.07528700 -0.43553700

H -1.49667900 1.56406700 -1.37375800

C -2.74592200 3.18547700 -0.78200100

H -3.03182000 3.80424100 0.07177800

H -2.35988500 3.84310000 -1.55964800

H -3.65452300 2.71641100 -1.17845500

P -0.01077700 2.51830700 0.14583200

C -0.18512200 3.11159200 1.95583200

C -1.43449300 3.96819300 2.20995000

H -2.34629200 3.38700800 2.04946600

H -1.43117000 4.28346900 3.26143800

H -1.47783000 4.86844400 1.59471700

C -0.30004800 1.87311200 2.85733900

H -1.25848200 1.36758600 2.71987800

H 0.50544300 1.15638700 2.67241800

H -0.24032600 2.19132600 3.90629100

C 1.05418700 3.88645800 2.42719100

H 1.20786800 4.82702800 1.89596800

H 0.91848800 4.13131600 3.48769400

H 1.96608600 3.28740000 2.35364200

C 0.53848200 4.00352000 -0.92080400

C 2.06067300 4.18637300 -0.79758900

H 2.58372500 3.30480500 -1.17389900

H 2.35490700 5.04184200 -1.41793000

H 2.39787800 4.38893000 0.22195600

C 0.28193200 3.68438700 -2.40444700

H 0.84387000 2.80706300 -2.72838400

H -0.76597600 3.49196800 -2.64127000

H 0.60556000 4.55126500 -2.99431200

C -0.13387900 5.33975300 -0.56649000

H 0.20027200 6.08957600 -1.29429700

H -1.22346900 5.30148200 -0.61416300

H 0.15181100 5.70477400 0.42294000

Rh 1.23310100 0.39007200 -0.29437000

C -3.48788400 -1.51535000 -2.10233900

Fe -3.72431100 -0.42120700 -0.27953000

C -3.87769900 -0.18136500 -2.38693300

C -4.50749900 -2.11234900 -1.31525300

C -5.53429500 -1.14818500 -1.11823600

C -5.14527000 0.04530700 -1.78298400

C -2.91200200 -0.87457500 1.66165200

C -3.97688200 0.03541500 1.81386400

C -3.64501300 1.21241900 1.09588100

C -2.37335000 1.04563000 0.48019400

C -1.90523700 -0.27268200 0.83834300

H -3.27838900 0.53923600 -2.92904900

H -4.48423000 -3.11302700 -0.90623900

H -6.43141300 -1.28344000 -0.52902600

H -5.69517400 0.97739800 -1.78240000

H -2.86075600 -1.85997400 2.10263300

H -4.90057000 -0.14873700 2.34510500

H -4.28637400 2.07561200 0.98349900

H -2.54967000 -1.96926700 -2.39406000

C 5.23322200 -2.25656700 -0.37980500

C 6.35513800 -2.23086300 -1.20475200

C 6.73117100 -1.04406800 -1.83428800

C 5.99833500 0.11589300 -1.61890800

C 4.88081600 0.09858800 -0.77577600

H 4.92015400 -3.18549800 0.09086000

H 6.93129500 -3.13695500 -1.36581700

H 7.59978100 -1.02440600 -2.48487300

H 6.29664000 1.04930300 -2.09016800

C 4.48648400 -1.10356300 -0.16618800

C 3.15807700 -1.15664000 0.54589900

C 2.69372300 0.16859200 1.16923300

H 3.16961600 -1.96577400 1.28974700

C 4.18619100 1.32263700 -0.41128100

C 3.30288800 1.36596800 0.62866300

H 4.51904300 2.24887500 -0.87499300

O 2.19266800 -1.40176200 -0.45949600

H 2.38607900 0.16447700 2.21378400

H 2.99616800 2.33291900 1.01079100

Cl 0.13112000 0.35812600 -2.61391000

**M3**

P 0.78488400 1.10208200 0.21925200

C 0.46767100 2.36963600 1.51947900

C 0.13314600 3.66168800 1.09336900

C 0.50738000 2.10679900 2.89048800

C -0.18079000 4.65566200 2.01284900

H 0.14204000 3.90227100 0.03420400

C 0.19819300 3.10548900 3.81168100

H 0.78536900 1.12371800 3.25065800

C -0.15443000 4.37863400 3.37792100

H -0.43718300 5.65038900 1.66160200

H 0.23498700 2.88124700 4.87324600

H -0.39634600 5.15439200 4.09776400

C 1.86364500 2.09114900 -0.90035300

C 1.47064200 2.39070100 -2.20554300

C 3.06941200 2.61542400 -0.41783500

C 2.28498800 3.17464700 -3.02403100

H 0.54877600 1.98007600 -2.59867400

C 3.88257200 3.39132600 -1.23541500

H 3.38295400 2.41481900 0.60098000

C 3.49333600 3.66829100 -2.54550100

H 1.97250400 3.38717300 -4.04152300

H 4.81816200 3.78408700 -0.84861500

H 4.12890100 4.27198900 -3.18589400

C 0.83204700 -2.45440900 0.09903400

H 1.00749800 -2.14666400 -0.93849800

C 1.24607300 -3.92721200 0.21267000

H 1.05750900 -4.35343200 1.20140600

H 0.73266600 -4.54366300 -0.52404000

H 2.32007300 -4.00809100 0.00656600

P -0.98814700 -1.99799000 0.26922200

C -1.44519100 -2.07021500 2.10744700

C -0.90358500 -3.30336800 2.84368900

H 0.19015100 -3.27770200 2.88703600

H -1.27053700 -3.27261300 3.87790600

H -1.21525800 -4.25467900 2.41109700

C -0.86683200 -0.83975800 2.82135000

H 0.22251400 -0.89325900 2.88665600

H -1.17775900 0.09132000 2.34121200

H -1.25370400 -0.83366900 3.84933800

C -2.97374800 -1.98515000 2.23415100

H -3.46997200 -2.91154100 1.93500900

H -3.22230400 -1.80950600 3.28787000

H -3.37374400 -1.14628500 1.65145600

C -1.96562000 -3.34771300 -0.66302900

C -3.41079000 -2.88147200 -0.90162500

H -3.42785500 -1.90130300 -1.37382600

H -3.89243300 -3.59880700 -1.57858800

H -4.01141900 -2.82948600 0.00669600

C -1.33921000 -3.50742800 -2.06009600

H -1.41901600 -2.58299500 -2.63603300

H -0.28242900 -3.78176400 -2.04531100

H -1.88373600 -4.29949600 -2.58873300

C -2.03621000 -4.71180100 0.04190000

H -1.06170000 -5.12399700 0.30785800

H -2.64836200 -4.66790000 0.94633300

H -2.52071200 -5.42094400 -0.64110800

Rh -1.12550700 0.23664400 -0.73892800

C 5.23781200 -0.01759100 -0.70025500

Fe 3.66706300 -0.99286100 0.35667200

C 4.18637300 -0.32687900 -1.60363100

C 5.71802100 -1.23725300 -0.14835600

C 4.95905500 -2.29869600 -0.70918200

C 4.00988000 -1.73426500 -1.60544400

C 2.86270100 0.16880000 1.97337200

C 3.37565000 -1.03548300 2.49412200

C 2.66617300 -2.10383100 1.88889000

C 1.70786200 -1.58043100 0.97738400

C 1.83213600 -0.14080600 1.02757000

H 3.58158600 0.38053800 -2.15617700

H 6.49133200 -1.33747400 0.60111500

H 5.05447000 -3.34713600 -0.45907900

H 3.24616200 -2.26620100 -2.15856700

H 3.18273000 1.16258200 2.25395800

H 4.18994700 -1.12961400 3.19936100

H 2.86651100 -3.15263300 2.05533500

H 5.58394500 0.97571800 -0.44939100

C -5.56990300 -0.04686500 -1.30182600

C -6.64006500 0.31286800 -0.48806300

C -6.49907900 1.34236800 0.44095700

C -5.27370400 1.99469200 0.57254000

C -4.19638900 1.63329200 -0.22628500

H -5.68731200 -0.83129100 -2.04617700

H -7.59074400 -0.20205100 -0.58830300

H -7.33771300 1.62725400 1.06869900

H -5.14407400 2.77371600 1.31988000

C -4.34493000 0.61737000 -1.18937900

C -3.23063600 0.36499900 -2.10896500

C -2.29579100 1.38159700 -2.33019700

H -3.36214600 -0.38652100 -2.88301500

C -2.78592900 2.09945700 0.03929500

C -1.95723000 2.22337900 -1.25706100

H -2.76905800 3.03466800 0.61706400

O -2.18009300 1.05516100 0.76322100

H -1.71456600 1.39747000 -3.24802700

H -1.30179500 3.07373700 -1.41411400

Cl 0.25392800 -0.57526900 -2.67233400

**TS_3-4_a1**

P 1.39723100 -1.18949400 0.03230500

C 0.70824000 -2.45566100 -1.11173900

C 0.42207900 -3.74385400 -0.64547100

C 0.29693700 -2.09982100 -2.40175700

C -0.25289600 -4.65878400 -1.45271200

H 0.72848700 -4.03817200 0.35501000

C -0.36835400 -3.01673600 -3.20897700

H 0.49197900 -1.09821700 -2.77055700

C -0.64989500 -4.29759900 -2.73704000

H -0.46029900 -5.65621500 -1.07616900

H -0.67444000 -2.72538900 -4.20926900

H -1.17192300 -5.01026300 -3.36843800

C 2.49450800 -2.23224100 1.07906900

C 2.47103200 -2.07144700 2.46525800

C 3.36398600 -3.17401600 0.51017300

C 3.32097900 -2.83425500 3.27179600

H 1.79340100 -1.34744700 2.91025200

C 4.20257700 -3.93491600 1.31511400

H 3.38277800 -3.31024500 -0.56858900

C 4.18512500 -3.76175700 2.70117800

H 3.29962400 -2.69729300 4.34862900

H 4.87351100 -4.66175400 0.86603700

H 4.84363200 -4.35404100 3.32952400

C 2.38572200 2.13991400 0.08193800

H 2.54248000 1.68430000 1.07104800

C 3.25317500 3.40485900 -0.00217200

H 3.09211600 3.95932300 -0.92844100

H 3.06271500 4.08669300 0.82288000

H 4.31139900 3.11981800 0.03782300

P 0.50144100 2.35134100 0.07936300

C 0.00761100 2.91456400 -1.67622400

C 0.82870600 4.06277200 -2.28181400

H 1.83411200 3.71735200 -2.54172900

H 0.34526400 4.36826700 -3.21926800

H 0.91400500 4.94682400 -1.65107200

C 0.13717300 1.73380800 -2.65914500

H 1.17352400 1.41513800 -2.79458500

H -0.47790400 0.89015800 -2.34372800

H -0.22484700 2.08279600 -3.63640700

C -1.48503100 3.28356600 -1.62758900

H -1.68240000 4.19941500 -1.06374300

H -1.83920200 3.44658300 -2.65357800

H -2.07541300 2.46603300 -1.19735500

C 0.17192900 3.78623400 1.30570100

C -1.27983800 3.62337500 1.79032600

H -1.36766400 2.72141200 2.40142300

H -1.54450100 4.49039800 2.40987000

H -1.99979000 3.55691000 0.97053600

C 1.06042500 3.64346100 2.55595500

H 1.04324100 2.62866300 2.95593300

H 2.09539300 3.94657100 2.38753500

H 0.65539400 4.31080700 3.32771000

C 0.34719200 5.20942500 0.75760000

H 1.35388700 5.39599500 0.37390300

H -0.37274900 5.44935200 -0.02913200

H 0.17481800 5.91281800 1.58239600

Rh -0.59868800 0.19935400 0.84372000

C 6.13883000 -1.34949000 -0.19921300

Fe 4.67693600 0.06023900 -0.86344500

C 5.55537300 -0.72006100 0.93163200

C 6.76309700 -0.34623200 -0.98867700

C 6.55935400 0.90446000 -0.34837200

C 5.81113100 0.67191400 0.83801200

C 3.21815600 -0.77941500 -2.19762600

C 3.89314300 0.28388400 -2.83732000

C 3.65651900 1.46281700 -2.08304700

C 2.82747600 1.14252600 -0.96923800

C 2.54586400 -0.26501900 -1.04692400

H 4.98194700 -1.21002800 1.70701200

H 7.25933700 -0.49965700 -1.93747900

H 6.87669400 1.86884300 -0.72273000

H 5.46164000 1.42643200 1.53077900

H 3.20496200 -1.81311700 -2.51791000

H 4.52182700 0.20681000 -3.71413600

H 4.08518800 2.43281900 -2.29367800

H 6.08376200 -2.40369500 -0.43270600

C -4.27879400 2.09276500 0.86947200

C -5.13172500 2.58508900 -0.11786600

C -5.26526300 1.91009600 -1.33030500

C -4.53313300 0.74115000 -1.55443200

C -3.66727600 0.25519500 -0.58138700

H -4.17447000 2.61838800 1.81588800

H -5.69278700 3.49776100 0.06105000

H -5.92007900 2.30117700 -2.10376100

H -4.59799000 0.23027800 -2.51345700

C -3.53100600 0.93130200 0.64713000

C -2.56603000 0.40428600 1.62501500

C -2.21222400 -0.99951100 1.59373000

H -2.54598800 0.89195500 2.59611300

C -2.63411000 -0.82213400 -0.84390500

C -2.45214500 -1.71435300 0.38104700

H -2.90949700 -1.43034300 -1.72070300

O -1.40486500 -0.20735500 -1.02161600

H -1.90775100 -1.51598200 2.49784900

H -1.90857500 -2.64090300 0.20600500

Cl 0.15372800 0.40876400 3.19976300

C -4.28640800 -3.27230800 1.62301900

H -4.18573000 -2.60108000 2.48168900

H -5.28879900 -3.70438500 1.59938300

H -3.53779900 -4.06389900 1.68409000

O -4.06587500 -2.56851700 0.40224200

H -4.82251200 -1.92426500 0.30120800

C -7.07552500 -0.35346200 0.77361000

O -6.45023700 -1.55034600 0.27178300

C -7.01393800 -1.90834900 -0.99310800

C -8.40749600 -1.29636500 -0.97451700

C -8.13538600 0.03177400 -0.26119800

H -7.51879000 -0.60164700 1.74386800

H -6.31109100 0.41525500 0.90802500

H -6.40387500 -1.48017900 -1.79986000

H -6.99748700 -2.99839600 -1.06968600

H -8.82892800 -1.17280100 -1.97429400

H -9.08344400 -1.92283100 -0.38344300

H -7.71886200 0.75848200 -0.96446700

H -9.02576900 0.46560900 0.19882200

**TS_3-4_a2**

P 1.02543900 -1.15960300 0.33987000

C -0.13163700 -2.41443600 -0.34760900

C -0.58112000 -3.45811700 0.47121200

C -0.76591300 -2.20530100 -1.58005200

C -1.64281300 -4.27125600 0.07417400

H -0.11029100 -3.63047000 1.43486400

C -1.82903900 -3.01406000 -1.97148900

H -0.44885200 -1.38495000 -2.21478700

C -2.27592300 -4.04478400 -1.14522000

H -1.96896900 -5.08165700 0.72026400

H -2.31819500 -2.83156500 -2.92383700

H -3.10352500 -4.67506200 -1.45890600

C 2.08896800 -2.21395900 1.40945300

C 2.43451800 -1.76147800 2.68411900

C 2.56557900 -3.45595600 0.96657500

C 3.25617200 -2.54258500 3.50262600

H 2.04955100 -0.80882800 3.04008600

C 3.37440900 -4.23360000 1.78607500

H 2.29798500 -3.81324200 -0.02550200

C 3.72382300 -3.77461800 3.05807900

H 3.52100200 -2.18308200 4.49224500

H 3.73801200 -5.19503900 1.43472700

H 4.35844300 -4.38104300 3.69737400

C 2.73431100 1.74290900 -0.31069700

H 2.92007000 1.35770300 0.70257300

C 3.87868100 2.70220400 -0.66987000

H 3.71801600 3.20353200 -1.62559200

H 4.01591700 3.47442700 0.08486100

H 4.81568000 2.13719200 -0.74396500

P 0.98760100 2.46253500 -0.11488800

C 0.41862500 3.00288100 -1.85631500

C 1.40887000 3.85075100 -2.67010100

H 2.24369500 3.23628900 -3.02070700

H 0.88702900 4.21586000 -3.56479000

H 1.80918000 4.71723300 -2.14445400

C 0.10638300 1.76500100 -2.72143300

H 1.00005700 1.18283700 -2.95444300

H -0.63579400 1.12468900 -2.24403800

H -0.30131600 2.13063700 -3.67457000

C -0.90496400 3.76440500 -1.67395800

H -0.76965000 4.74337400 -1.20586900

H -1.35305000 3.93127400 -2.66170000

H -1.61845400 3.18299200 -1.07918900

C 1.21266500 4.01286000 0.97728600

C -0.15145200 4.28848100 1.64021800

H -0.39602300 3.48649700 2.34164300

H -0.08369000 5.23053400 2.20015800

H -0.96918500 4.37876000 0.91955100

C 2.19629000 3.69592700 2.11927200

H 1.96428600 2.74578000 2.60436300

H 3.23968500 3.69319400 1.79613600

H 2.09673200 4.48362200 2.87701000

C 1.68740500 5.29417800 0.27890200

H 2.65028500 5.17075700 -0.22489800

H 0.96293000 5.67335400 -0.44654000

H 1.81649000 6.06999700 1.04470400

Rh -0.52756100 0.76638700 1.05132500

C 5.40012300 -2.64098300 -0.66796700

Fe 4.24092200 -0.96405800 -1.31709000

C 5.30098000 -1.70924100 0.39864400

C 6.05011000 -1.99715800 -1.75544800

C 6.34639900 -0.66559700 -1.36240300

C 5.88201300 -0.48942500 -0.03053900

C 2.37227900 -1.50922300 -2.22801800

C 3.14191800 -0.73665100 -3.12792900

C 3.36458100 0.53240200 -2.53027600

C 2.72205300 0.56263600 -1.25925200

C 2.09585000 -0.71731000 -1.07295800

H 4.83237000 -1.88458500 1.35697900

H 6.23688000 -2.42769700 -2.73009600

H 6.79786600 0.09493800 -1.98568300

H 5.92879100 0.42422200 0.54698900

H 2.04787800 -2.53134400 -2.37515700

H 3.54017200 -1.07150600 -4.07622000

H 3.96352000 1.32711500 -2.95384800

H 5.01493700 -3.65105800 -0.66177900

C -3.71893900 3.45183200 1.01809200

C -4.54126200 4.00313300 0.03604900

C -4.88396400 3.26085300 -1.09100400

C -4.39647600 1.95982300 -1.23379100

C -3.58198700 1.40424000 -0.25702600

H -3.44857100 4.03325600 1.89645200

H -4.91490500 5.01615400 0.15225700

H -5.51673500 3.69473800 -1.85929900

H -4.62675900 1.38435700 -2.12886600

C -3.22775000 2.15194900 0.88100900

C -2.33249900 1.52525500 1.86923300

C -2.31650600 0.08439300 1.99900900

H -2.12902300 2.09909000 2.76977300

C -2.85232900 0.09209800 -0.42378300

C -2.80805700 -0.68074300 0.89275400

H -3.32018400 -0.52427100 -1.21304900

O -1.52143000 0.34016100 -0.71588200

H -2.07888200 -0.39029600 2.94537900

H -2.48509900 -1.71901700 0.80876600

Cl 0.40309100 1.02017000 3.34286000

C -4.73997900 -1.88803500 2.27135800

H -4.42868800 -1.30453600 3.13794100

H -5.80018300 -2.13923300 2.36105800

H -4.13574400 -2.80079300 2.20446900

O -4.54867700 -1.06694800 1.12213600

H -4.78837100 -1.58049900 0.29363400

C -6.10709800 -1.34708300 -1.73550900

O -5.23821800 -2.29308300 -1.07407800

C -5.93815000 -3.52570000 -0.87790700

C -7.39747900 -3.11421900 -0.74566800

C -7.49008600 -2.01115400 -1.80687000

H -6.10396600 -0.43030500 -1.13620800

H -5.68859500 -1.12296500 -2.72059000

H -5.78114300 -4.17401400 -1.74972000

H -5.51134500 -4.01156000 0.00376400

H -8.09241700 -3.93849900 -0.91779400

H -7.58222100 -2.70493700 0.25376100

H -7.65148100 -2.45458400 -2.79379000

H -8.29693100 -1.30080000 -1.61819500

**TS_3-4_a3**

P 0.82804800 0.82697000 0.23760000

C 0.13688800 1.83461800 1.61964000

C -0.19051200 3.18391800 1.44197100

C -0.26318900 1.20207900 2.80525800

C -0.89929000 3.88437900 2.41883300

H 0.11297100 3.70043200 0.53571300

C -0.96214300 1.90264900 3.78234100

H -0.03426100 0.15300700 2.95634600

C -1.28887300 3.24549500 3.59227800

H -1.13603500 4.93429200 2.26556900

H -1.25753900 1.39420000 4.69519300

H -1.83257000 3.78990600 4.35892900

C 1.82211900 2.09577300 -0.65808800

C 1.76737800 2.12496800 -2.05404700

C 2.64297300 3.01164100 0.01510200

C 2.53309900 3.05273900 -2.76663100

H 1.12765200 1.41986300 -2.58162700

C 3.39535000 3.94022100 -0.69552600

H 2.69246500 2.99586700 1.10156900

C 3.34443400 3.95895400 -2.09175500

H 2.48860700 3.06098800 -3.85153700

H 4.02836100 4.64524800 -0.16419200

H 3.93847300 4.67923800 -2.64639900

C 1.97021800 -2.39308600 -0.42029900

H 2.05269500 -1.76182700 -1.31763200

C 2.90842700 -3.59635500 -0.59457800

H 2.82909500 -4.30741300 0.23020000

H 2.70820600 -4.14175500 -1.51433800

H 3.94682100 -3.24451400 -0.63454900

P 0.09891000 -2.71007000 -0.37918100

C -0.28977600 -3.59634800 1.26180400

C 0.61227400 -4.78489700 1.62438500

H 1.61176900 -4.43487900 1.90187500

H 0.18965700 -5.27912600 2.50916600

H 0.70992700 -5.53558100 0.84072100

C -0.18339900 -2.59735600 2.43093700

H 0.83162700 -2.21505600 2.56489500

H -0.88322300 -1.77016800 2.30044800

H -0.45109100 -3.13818300 3.34954700

C -1.76490700 -4.02897100 1.19798700

H -1.94551700 -4.83281700 0.47922000

H -2.06396700 -4.39833100 2.18724300

H -2.40945400 -3.17622900 0.95004100

C -0.23494700 -3.91474800 -1.82603200

C -1.71445900 -3.72729400 -2.21846700

H -1.85338400 -2.74426300 -2.67542400

H -1.98128500 -4.49457900 -2.95722200

H -2.40427300 -3.81073800 -1.37381400

C 0.59037800 -3.50221400 -3.05977700

H 0.53866200 -2.42861200 -3.24772900

H 1.63626300 -3.80906500 -2.99877900

H 0.16041100 -4.00908300 -3.93335500

C 0.02429000 -5.40383600 -1.55973100

H 1.05972900 -5.60863400 -1.27187900

H -0.63634400 -5.81909200 -0.79439400

H -0.17002000 -5.95319400 -2.49009200

Rh -1.15672700 -0.52405200 -0.73471000

C 5.55288700 1.22508300 0.39275500

Fe 4.19376100 -0.36874600 0.81188700

C 4.97074000 0.78332000 -0.82440500

C 6.25544900 0.13101100 0.96719200

C 6.10225500 -0.98767700 0.10675300

C 5.30566000 -0.58375800 -0.99936600

C 2.74249700 0.13412800 2.30828700

C 3.49296300 -0.98401800 2.73334000

C 3.29264200 -2.02407800 1.78807300

C 2.40824200 -1.56461600 0.77005900

C 2.06090500 -0.20801900 1.10006600

H 4.34928100 1.37350200 -1.48427900

H 6.77207900 0.13442900 1.91745300

H 6.48109400 -1.98488700 0.28749000

H 4.98117600 -1.21448800 -1.81646400

H 2.68630800 1.09419400 2.80502400

H 4.14663300 -1.02419100 3.59414500

H 3.77595700 -2.99092900 1.81491000

H 5.44532400 2.21111600 0.82383300

C -4.94363700 -2.36432500 -0.84482100

C -5.83755200 -2.91396400 0.07361800

C -5.91831100 -2.40549200 1.36730900

C -5.08626700 -1.35095100 1.74789300

C -4.18913100 -0.80709000 0.83996400

H -4.89012400 -2.75327000 -1.85909600

H -6.47695100 -3.73923600 -0.22538200

H -6.61725800 -2.83300300 2.07959100

H -5.11960600 -0.96573000 2.76530100

C -4.11402700 -1.30497300 -0.47351600

C -3.17447000 -0.65884600 -1.40645100

C -2.79460100 0.71486000 -1.18838900

H -3.21717600 -0.99160500 -2.44032800

C -3.11482700 0.18213700 1.21231100

C -2.93325000 1.25490000 0.13848300

H -3.31286700 0.63680100 2.19772600

O -1.88227900 -0.45859900 1.21115000

H -2.57505500 1.35730700 -2.03304700

H -2.24927000 2.04944300 0.43752900

Cl -0.53020500 -0.25039700 -3.13032400

C -4.63010800 2.89258300 1.40772300

H -3.69419500 3.18292000 1.89949000

H -5.27512000 3.76240300 1.26304300

H -5.15214700 2.13902000 1.99611200

O -4.35742700 2.30339000 0.12779000

H -3.94426600 3.00489000 -0.44172100

C -1.51915800 3.72802000 -1.74670000

O -2.61505500 4.08773400 -0.88011300

C -2.67044600 5.50845000 -0.79298300

C -1.20979500 5.94213500 -0.79697700

C -0.57438000 4.94532800 -1.78161500

H -1.05279400 2.82862700 -1.33269700

H -1.90949900 3.48555800 -2.74114700

H -3.21000000 5.91818200 -1.65880000

H -3.21336000 5.76464500 0.12095900

H -1.08133900 6.98301800 -1.10162800

H -0.78460800 5.82284900 0.20371600

H -0.53589400 5.36782300 -2.78901900

H 0.44770800 4.67464700 -1.50021400

**TS_3-4_a4**

P 1.36912200 -1.16219700 0.01632200

C 0.86379200 -2.44249900 -1.20597400

C 0.57869600 -3.74529900 -0.78118100

C 0.56117100 -2.08274900 -2.52545700

C 0.00391600 -4.66937400 -1.65350100

H 0.79916100 -4.04257300 0.24057900

C -0.00541800 -3.00765300 -3.39668300

H 0.75601800 -1.07085000 -2.86454600

C -0.29209200 -4.30199200 -2.96351700

H -0.20614200 -5.67668800 -1.30628000

H -0.22974500 -2.71359300 -4.41780900

H -0.73284100 -5.02174600 -3.64712400

C 2.38045800 -2.16899000 1.17734700

C 2.19079700 -2.01961300 2.55184500

C 3.34293500 -3.07460600 0.70826600

C 2.96893500 -2.75805300 3.44808800

H 1.43730900 -1.32744700 2.91916200

C 4.10932000 -3.81215200 1.60181000

H 3.49068000 -3.20018900 -0.36204500

C 3.92563100 -3.65045200 2.97733500

H 2.81806600 -2.63137900 4.51577500

H 4.85204700 -4.51204600 1.22973400

H 4.52832300 -4.22424100 3.67498800

C 2.20438500 2.19113200 0.24651000

H 2.26555400 1.70956800 1.23361800

C 3.02497000 3.48872700 0.30160000

H 2.96350700 4.05902800 -0.62646500

H 2.70449400 4.14344100 1.10867500

H 4.07957600 3.24054500 0.47249000

P 0.32757900 2.33287500 0.01994400

C 0.03175100 2.93551500 -1.76591500

C 0.86754300 4.14031800 -2.22613600

H 1.91016600 3.84683800 -2.38273000

H 0.48108200 4.46862300 -3.20014300

H 0.84260300 4.99887400 -1.55614600

C 0.33462900 1.80183600 -2.76644200

H 1.39315000 1.53310100 -2.78369800

H -0.27237500 0.91983000 -2.56002100

H 0.07831000 2.17617500 -3.76757800

C -1.46942100 3.24613500 -1.89172900

H -1.77221500 4.12783800 -1.32009200

H -1.69736700 3.44445600 -2.94669000

H -2.07533000 2.38950300 -1.57487300

C -0.21075700 3.70514000 1.23994400

C -1.70172600 3.46562500 1.54159000

H -1.81790300 2.54808100 2.12365500

H -2.07911200 4.30644900 2.13842700

H -2.31416700 3.38403800 0.63915200

C 0.53421200 3.54649900 2.57967400

H 0.54076800 2.51217000 2.92613800

H 1.55790500 3.92376900 2.54892300

H -0.00183800 4.14085700 3.33087000

C -0.03065300 5.15462800 0.76864500

H 1.00787100 5.39379700 0.52415000

H -0.65560700 5.40181700 -0.09322900

H -0.33339100 5.81607100 1.59068400

Rh -0.77132700 0.12120500 0.58180700

C 6.10291100 -1.15308600 0.28933400

Fe 4.67205400 0.22680100 -0.48855500

C 5.37747400 -0.58191300 1.36767100

C 6.77202800 -0.10314200 -0.39623600

C 6.45419500 1.11749300 0.25532400

C 5.59103300 0.81990900 1.34488800

C 3.39873800 -0.62616600 -1.99768000

C 4.09921000 0.47808100 -2.53219100

C 3.73874800 1.62611300 -1.77835900

C 2.80456300 1.24462200 -0.77262000

C 2.58671400 -0.16893400 -0.91557500

H 4.73742000 -1.11513700 2.05713800

H 7.37317800 -0.20683900 -1.28957400

H 6.77196600 2.10400300 -0.05519200

H 5.14717200 1.53754000 2.02236600

H 3.45776700 -1.65021100 -2.34304000

H 4.82095100 0.44692700 -3.33723400

H 4.14996300 2.61654100 -1.91587200

H 6.11173300 -2.19971200 0.01732200

C -4.54931500 1.81284700 0.05968600

C -5.32133900 2.22207300 -1.02838600

C -5.25264400 1.53294200 -2.23752600

C -4.38840800 0.44174800 -2.36031200

C -3.61054300 0.03589500 -1.28324400

H -4.60498200 2.35375800 1.00272800

H -5.97951700 3.08069300 -0.92973000

H -5.85342100 1.85124000 -3.08378300

H -4.29426600 -0.07743000 -3.31229800

C -3.69002300 0.71530700 -0.05135000

C -2.84001300 0.23578400 1.05346500

C -2.43413200 -1.15695200 1.07235800

H -2.98365500 0.71270600 2.01991300

C -2.47614200 -0.95746700 -1.39725600

C -2.41606900 -1.85694900 -0.16473600

H -2.56283200 -1.55511000 -2.31893200

O -1.28055700 -0.25454900 -1.38487300

H -2.25021800 -1.67517000 2.00712600

H -1.81617200 -2.75999200 -0.24968000

Cl -0.38912900 0.28276100 3.03044300

C -4.38265500 -3.35703200 -1.56107000

H -3.49792500 -3.81806500 -2.00716600

H -5.15953900 -4.11508600 -1.42573000

H -4.74939400 -2.55787500 -2.21470600

O -3.99727300 -2.84620900 -0.29081500

H -4.75845000 -2.33092400 0.10904300

C -5.96913400 -1.64068200 2.22697900

O -6.11755100 -1.82569100 0.81300500

C -6.90511000 -0.76290200 0.24477700

C -7.29141700 0.15138200 1.41103000

C -6.21925300 -0.15777300 2.46126100

H -6.70719000 -2.26525700 2.74614400

H -4.96244600 -1.96699000 2.50243600

H -6.29216200 -0.24601500 -0.49850600

H -7.77407100 -1.20797400 -0.24941900

H -7.30145200 1.20151700 1.11020900

H -8.28354600 -0.10936500 1.79341000

H -5.30577900 0.40619500 2.25590000

H -6.54259500 0.05540000 3.48255300

**TS_3-4_a5**

P 0.93108400 0.94124900 0.34714900

C 0.36422000 1.85969500 1.84118000

C 0.07762100 3.22843300 1.78502200

C 0.05337300 1.15745800 3.01343400

C -0.50247900 3.88145000 2.87360700

H 0.32276700 3.79887400 0.89327600

C -0.51814000 1.81131900 4.09982700

H 0.25347800 0.09294300 3.06788100

C -0.80487100 3.17441000 4.03272700

H -0.70458200 4.94759800 2.81509100

H -0.74751600 1.25082400 5.00098500

H -1.25456100 3.68114800 4.88109300

C 1.88091100 2.25425000 -0.52900800

C 1.69886900 2.42125700 -1.90323100

C 2.78733400 3.08072100 0.14880700

C 2.42188700 3.40001800 -2.59150300

H 0.99095300 1.78707300 -2.43288200

C 3.49802100 4.05931700 -0.53567900

H 2.93646100 2.95347000 1.21871300

C 3.31739600 4.21853400 -1.91163200

H 2.27777100 3.51753900 -3.66139100

H 4.19734200 4.69565700 -0.00071700

H 3.87664700 4.97992700 -2.44709700

C 2.02739600 -2.20577500 -0.64201100

H 2.05620300 -1.50081400 -1.48606600

C 2.95678900 -3.38168600 -0.97845600

H 2.92032500 -4.17074800 -0.22557600

H 2.71427000 -3.83406900 -1.93734900

H 3.99063700 -3.02005500 -1.03341200

P 0.16459400 -2.54007900 -0.50639600

C -0.10535900 -3.57633400 1.07029700

C 0.83117300 -4.77784200 1.26934500

H 1.84380700 -4.44095200 1.51217700

H 0.47158100 -5.34959000 2.13524100

H 0.88263900 -5.46171600 0.42211800

C 0.06657600 -2.68112000 2.31282500

H 1.08838700 -2.30995500 2.42292400

H -0.63361800 -1.84430700 2.29337800

H -0.15176400 -3.29952600 3.19462400

C -1.57238000 -4.03868800 1.06172200

H -1.77901700 -4.78910200 0.29341600

H -1.80153400 -4.49174500 2.03463100

H -2.25195100 -3.19004800 0.92115200

C -0.24455500 -3.61295300 -2.03691900

C -1.74862100 -3.43688000 -2.31783700

H -1.94010700 -2.42207400 -2.67466000

H -2.04360800 -4.14244900 -3.10566400

H -2.37820700 -3.61929300 -1.44237000

C 0.48829000 -3.06266800 -3.27467500

H 0.37547600 -1.98099600 -3.36741600

H 1.54953900 -3.31766700 -3.29768000

H 0.03175100 -3.51841400 -4.16293300

C 0.06601400 -5.11090600 -1.92008000

H 1.11782400 -5.30743000 -1.69627400

H -0.54655700 -5.60940400 -1.16457700

H -0.15859100 -5.58225300 -2.88564400

Rh -1.12144500 -0.34568100 -0.57027400

C 5.64732400 1.33197200 0.18827700

Fe 4.32721100 -0.29658200 0.60300700

C 4.99814300 0.94771900 -1.01425600

C 6.39169200 0.21847500 0.66288800

C 6.19729700 -0.85622300 -0.24349900

C 5.33392300 -0.40455800 -1.27901100

C 2.97659400 0.08715300 2.22938800

C 3.74974500 -1.06046800 2.51292000

C 3.48441800 -2.02189100 1.50278000

C 2.53745800 -1.48144200 0.58574600

C 2.21366300 -0.15872800 1.04636700

H 4.33047300 1.56303400 -1.60253300

H 6.96440800 0.18021100 1.57959200

H 6.59535400 -1.85664700 -0.13701800

H 4.96909700 -0.99543400 -2.10881300

H 2.95580500 1.00237500 2.80674400

H 4.45955300 -1.16840600 3.32188800

H 3.96568800 -2.98652600 1.41766700

H 5.55952900 2.29349800 0.67462700

C -4.80396100 -2.37557800 -0.52792600

C -5.58595600 -3.04098800 0.41557400

C -5.56758800 -2.64201800 1.74984000

C -4.75393200 -1.57717400 2.14283100

C -3.96940400 -0.91555700 1.20808900

H -4.82622200 -2.67991200 -1.57199700

H -6.21483000 -3.87041100 0.10616100

H -6.17724500 -3.16106000 2.48300400

H -4.71896800 -1.26803500 3.18521800

C -3.98756600 -1.31052100 -0.14237300

C -3.16227300 -0.54598700 -1.09577000

C -2.86399000 0.84094600 -0.80719000

H -3.27235200 -0.80837300 -2.14502300

C -2.94152300 0.13163100 1.56367100

C -2.94473500 1.27691800 0.55360300

H -3.10136000 0.51920900 2.58185800

O -1.67384100 -0.42268700 1.42733400

H -2.72062200 1.54757300 -1.61386900

H -2.34447000 2.13428000 0.85805300

Cl -0.72515600 0.15206000 -2.98731500

C -5.67255500 1.58318100 0.27538600

H -6.02765500 0.83943800 0.98651500

H -6.41990200 2.36800900 0.13822400

H -5.44664300 1.09901100 -0.68080400

O -4.48885900 2.17857600 0.82509100

H -4.25708300 2.97199900 0.26259000

C -3.63274100 3.91687100 -2.20905000

O -3.63087200 4.10053300 -0.78348300

C -2.36756100 4.66705800 -0.42030400

C -1.36762700 3.97007800 -1.33311800

C -2.15372200 3.84137700 -2.65017700

H -4.19776500 3.00472300 -2.41798300

H -4.15223400 4.76533200 -2.66779200

H -2.39523900 5.75053800 -0.60093500

H -2.21616400 4.48550400 0.64754900

H -0.43340000 4.52622200 -1.44824800

H -1.11823200 2.97633400 -0.94100300

H -1.92019200 4.66816500 -3.32602700

H -1.91448000 2.90418900 -3.16162900

**TS_3-4_a6**

P 1.02148200 -1.10593900 0.31228800

C 0.11557800 -2.48701000 -0.51626100

C -0.23175700 -3.63482900 0.20757500

C -0.31047500 -2.38307600 -1.84615600

C -0.93765000 -4.67534800 -0.39567500

H 0.07490400 -3.73379700 1.24514500

C -1.00907200 -3.42466700 -2.45027000

H -0.07199700 -1.49371800 -2.41934600

C -1.31239400 -4.58171400 -1.73284500

H -1.17666700 -5.56540100 0.17903300

H -1.30509900 -3.33562200 -3.49205300

H -1.83918300 -5.40233900 -2.21272400

C 2.04691900 -2.08064000 1.49546600

C 2.06520600 -1.72457300 2.84442900

C 2.80580000 -3.17970800 1.06676500

C 2.85262800 -2.44344200 3.74881400

H 1.48299500 -0.87227400 3.18263500

C 3.57998500 -3.89764000 1.96891400

H 2.79277600 -3.47241500 0.01992900

C 3.61018000 -3.52460200 3.31492800

H 2.86795100 -2.14870800 4.79362900

H 4.16462700 -4.74696000 1.62668100

H 4.22195800 -4.08184900 4.01810500

C 2.39611800 2.02009500 -0.45396400

H 2.58516400 1.79949500 0.60711600

C 3.38108900 3.11021500 -0.90129100

H 3.20431000 3.43269100 -1.92938900

H 3.32965600 3.99281200 -0.26933800

H 4.40411700 2.71917900 -0.84272400

P 0.55556100 2.46101600 -0.39859200

C -0.04449800 2.57826400 -2.20399500

C 0.83177400 3.39626300 -3.16452000

H 1.77491700 2.87608400 -3.35946400

H 0.30525200 3.47253700 -4.12526300

H 1.05405400 4.40875000 -2.82932000

C -0.13894600 1.16463900 -2.81019600

H 0.84164200 0.69858600 -2.93426500

H -0.79174000 0.52619300 -2.21319600

H -0.57831700 1.26736400 -3.81251700

C -1.47780600 3.13488100 -2.16621600

H -1.51660700 4.19336100 -1.89609600

H -1.91861500 3.03643800 -3.16648500

H -2.10122700 2.56282200 -1.46782800

C 0.47861400 4.21007000 0.38543500

C -0.93845500 4.37224000 0.96277500

H -1.08423500 3.66976500 1.78697700

H -1.04425600 5.39258600 1.35412200

H -1.73053000 4.20670500 0.22870800

C 1.43589300 4.31834800 1.58766900

H 1.31832400 3.48428100 2.28000200

H 2.48683000 4.39586300 1.30482300

H 1.18419000 5.24138700 2.12614900

C 0.76374900 5.38981100 -0.55456200

H 1.75212200 5.33357200 -1.01846100

H 0.01609000 5.49321600 -1.34462800

H 0.73419100 6.31073200 0.04192200

Rh -0.62845900 0.71520300 0.98277300

C 5.71531500 -1.86583300 -0.03329100

Fe 4.35753500 -0.49108500 -0.94765300

C 5.23381200 -0.96468400 0.95263800

C 6.39282700 -1.11052900 -1.02841500

C 6.32589200 0.25859200 -0.65911800

C 5.60722100 0.34775700 0.56435500

C 2.73967300 -1.43323200 -2.00770600

C 3.49001200 -0.63721600 -2.89983800

C 3.43024700 0.70254800 -2.43704300

C 2.64107400 0.75281000 -1.25131200

C 2.20034800 -0.59183900 -0.98610800

H 4.64730100 -1.22721500 1.82312200

H 6.83743200 -1.50306700 -1.93291600

H 6.71049600 1.09075600 -1.23387400

H 5.35400200 1.25826500 1.09188200

H 2.58841100 -2.50223300 -2.08084300

H 4.05320700 -0.98959100 -3.75341700

H 3.94963800 1.53717000 -2.88664000

H 5.55963700 -2.93558800 -0.04109700

C -4.09533700 3.06783500 1.19573400

C -5.10905800 3.43133300 0.31100100

C -5.49592300 2.56493400 -0.70860900

C -4.86282700 1.32645800 -0.83599700

C -3.86235100 0.95264700 0.05232300

H -3.79173700 3.74720700 1.98893900

H -5.59618900 4.39596000 0.41796200

H -6.27635200 2.85465100 -1.40546300

H -5.13005900 0.65809900 -1.65293000

C -3.45668800 1.83169300 1.07300600

C -2.38076500 1.38953400 1.97687100

C -2.21400300 -0.02693200 2.23143900

H -2.12817500 2.06097600 2.79390800

C -3.03612400 -0.30466200 -0.09450700

C -2.75496600 -0.93396500 1.27289300

H -3.56079400 -1.02361600 -0.74771200

O -1.78737100 0.00913900 -0.59814700

H -1.83242100 -0.38711600 3.18084800

H -2.35903700 -1.94810100 1.25093000

Cl 0.58100300 1.35357300 3.04180500

C -5.27776800 -0.57871900 2.45780500

H -5.81320000 0.02111300 1.71665200

H -5.97569600 -1.12735500 3.09514000

H -4.65776500 0.07732000 3.07188400

O -4.39843700 -1.51507000 1.83460500

H -4.84644500 -1.99127200 1.07810900

C -4.39364800 -3.68014000 -0.78517500

O -5.40876300 -2.85321100 -0.18501500

C -6.17925400 -2.18547600 -1.19727800

C -5.42885300 -2.41803600 -2.50557000

C -4.78173400 -3.78390300 -2.25576400

H -3.41551900 -3.19748400 -0.66727700

H -4.38184500 -4.63767900 -0.25843200

H -7.17918600 -2.63394900 -1.21933000

H -6.26655600 -1.13073400 -0.91982900

H -6.09182000 -2.40409400 -3.37319000

H -4.65477300 -1.65586100 -2.64756200

H -5.51219000 -4.58583500 -2.40469500

H -3.91694400 -3.97310000 -2.89443800

**M4-a1**

P 1.36808300 -1.17565400 0.06545400

C 0.66971700 -2.46238800 -1.05076200

C 0.40509700 -3.74855000 -0.56565700

C 0.20934500 -2.11735800 -2.32780400

C -0.29599100 -4.67221300 -1.34097600

H 0.74720200 -4.03415300 0.42554400

C -0.48335300 -3.04215200 -3.10267800

H 0.38296700 -1.11606600 -2.70787800

C -0.74327000 -4.32100900 -2.61166600

H -0.48184500 -5.66913700 -0.95122300

H -0.82765900 -2.75848800 -4.09282800

H -1.28459200 -5.04020600 -3.21927600

C 2.48989000 -2.20096200 1.10479600

C 2.49187800 -2.01622700 2.48828800

C 3.35034200 -3.15109200 0.53626300

C 3.35733000 -2.76490900 3.29171100

H 1.81626000 -1.28946400 2.93305100

C 4.20299200 -3.89930700 1.33828800

H 3.35098600 -3.30296800 -0.54071000

C 4.21048400 -3.70283500 2.72135400

H 3.35548700 -2.61022000 4.36638900

H 4.86648400 -4.63307900 0.88922100

H 4.88015400 -4.28487300 3.34760000

C 2.39309700 2.14871300 0.07669900

H 2.55478700 1.68326200 1.06031100

C 3.28501300 3.39607900 -0.00844400

H 3.11590800 3.96758000 -0.92295200

H 3.12253700 4.06934900 0.83088300

H 4.33887200 3.09232600 0.00649200

P 0.50906300 2.37794600 0.09573500

C 0.00913200 2.99084600 -1.64062800

C 0.84165300 4.13453400 -2.23674400

H 1.83331900 3.77338900 -2.52702500

H 0.34727600 4.47991400 -3.15481200

H 0.96214800 4.99643800 -1.58014800

C 0.09612300 1.81820200 -2.63708000

H 1.12247900 1.47425300 -2.78723800

H -0.53175500 0.98477900 -2.31563300

H -0.27021700 2.18194800 -3.60760000

C -1.47461600 3.38948500 -1.56415000

H -1.64187000 4.30430700 -0.98850000

H -1.84372200 3.56921700 -2.58234300

H -2.07572000 2.58108100 -1.13008700

C 0.21107300 3.78423700 1.35885500

C -1.24193100 3.63483400 1.84698000

H -1.36340500 2.68490900 2.37559200

H -1.46571000 4.45287800 2.54487700

H -1.97142800 3.67437800 1.03346300

C 1.10230500 3.56076000 2.59419100

H 1.02345500 2.53829400 2.96921500

H 2.15342100 3.79755900 2.41698000

H 0.75171700 4.23317000 3.38812800

C 0.41751900 5.22138000 0.86370300

H 1.42784500 5.39838300 0.48474000

H -0.29574300 5.50292800 0.08432200

H 0.25969700 5.90209000 1.71061100

Rh -0.65027100 0.17190200 0.86821400

C 6.10941700 -1.37418800 -0.28160800

Fe 4.64198900 0.04333700 -0.92158200

C 5.56797000 -0.72294100 0.85786600

C 6.71954300 -0.38851600 -1.10366800

C 6.54884200 0.87286900 -0.47466700

C 5.83555600 0.66445200 0.73720800

C 3.13834100 -0.78602600 -2.21189700

C 3.80769800 0.26842200 -2.87260100

C 3.60282500 1.45176100 -2.11586400

C 2.79935100 1.14356700 -0.98048900

C 2.50067700 -0.26191600 -1.04572300

H 5.01509600 -1.19558200 1.65868200

H 7.18512800 -0.55936300 -2.06498600

H 6.86356800 1.82890400 -0.87203500

H 5.51438800 1.43209800 1.42917100

H 3.10764500 -1.82086400 -2.52767200

H 4.41187000 0.18213700 -3.76573200

H 4.03418400 2.41789800 -2.33939400

H 6.03569700 -2.43078900 -0.49882400

C -4.28723800 2.08079900 0.92696300

C -5.11613600 2.64659400 -0.04086500

C -5.24531600 2.04828100 -1.29414000

C -4.52882100 0.88216200 -1.57631400

C -3.69117300 0.31820800 -0.62071600

H -4.18286600 2.55084700 1.90219000

H -5.66020800 3.55958700 0.18331700

H -5.88006500 2.49769800 -2.05247300

H -4.58315600 0.43366000 -2.56715800

C -3.56099200 0.91504900 0.65103500

C -2.63188900 0.31246900 1.61509700

C -2.20686200 -1.06038500 1.47897300

H -2.62252200 0.74359200 2.61312200

C -2.68285100 -0.77145800 -0.92936400

C -2.58955600 -1.76876700 0.23175000

H -2.95883500 -1.30300000 -1.85797700

O -1.42798800 -0.20542900 -1.02614900

H -1.95304000 -1.63819100 2.36371400

H -1.93816600 -2.60555800 -0.02951400

Cl 0.04384000 0.33522100 3.25224100

C -4.09357500 -3.40360100 1.42465300

H -3.99310300 -2.87684900 2.37511500

H -5.07737700 -3.86275400 1.33122200

H -3.30633400 -4.14936800 1.31258200

O -3.95947200 -2.47989300 0.32978500

H -4.79494000 -1.86310100 0.31113700

C -6.92865700 -0.40476700 0.78070300

O -6.26008100 -1.57394300 0.24624000

C -6.78064600 -1.89289800 -1.05506600

C -8.19410100 -1.33319700 -1.03168100

C -7.98609300 -0.02706500 -0.25831300

H -7.36940600 -0.70464400 1.73594700

H -6.18714400 0.37966500 0.94234800

H -6.16494500 -1.39998300 -1.81762100

H -6.72030500 -2.97684600 -1.17814600

H -8.60054900 -1.18066900 -2.03342200

H -8.85647600 -2.01078100 -0.48333200

H -7.58883000 0.74526000 -0.92209600

H -8.90118200 0.34802500 0.20435900

**M4-a2**

P 1.02681300 -1.15205500 0.33817600

C -0.11295500 -2.41979000 -0.36026600

C -0.55519300 -3.47132600 0.45283500

C -0.74653600 -2.21421600 -1.59429700

C -1.60631700 -4.29545600 0.04949400

H -0.08591400 -3.64156600 1.41764100

C -1.79943000 -3.03349300 -1.99261300

H -0.43732600 -1.38738200 -2.22419400

C -2.23816000 -4.07249200 -1.17163200

H -1.92321500 -5.11374300 0.69050000

H -2.28426500 -2.85448500 -2.94805400

H -3.05264000 -4.71633200 -1.49329300

C 2.10065200 -2.20604700 1.39893500

C 2.43987500 -1.75729500 2.67672300

C 2.58987400 -3.44105000 0.95037900

C 3.26836800 -2.53469900 3.49193900

H 2.04255600 -0.81143100 3.03808400

C 3.40474400 -4.21565500 1.76680800

H 2.32762600 -3.79488200 -0.04438400

C 3.74840300 -3.75989200 3.04156300

H 3.52830400 -2.17801400 4.48390500

H 3.77798000 -5.17176200 1.41085200

H 4.38806100 -4.36358000 3.67850400

C 2.73227700 1.76898000 -0.31647400

H 2.92458400 1.38018600 0.69413500

C 3.87551200 2.72892900 -0.67575600

H 3.71054400 3.23664200 -1.62744400

H 4.01346700 3.49686700 0.08408900

H 4.81377100 2.16656000 -0.75685800

P 0.98130000 2.47606600 -0.10479800

C 0.41093700 3.05466000 -1.83223900

C 1.39658700 3.91341400 -2.63869100

H 2.22089800 3.29963100 -3.01532800

H 0.86893100 4.30897400 -3.51720200

H 1.81341300 4.76015100 -2.09305000

C 0.08612300 1.82803700 -2.70732800

H 0.97545100 1.24118500 -2.94727400

H -0.65513300 1.18736200 -2.22714800

H -0.32396400 2.20169300 -3.65654600

C -0.91081300 3.81479700 -1.62877000

H -0.76866200 4.79202100 -1.15893500

H -1.37358000 3.98561600 -2.60931500

H -1.61597800 3.23110300 -1.02505800

C 1.20480800 4.00399100 1.01803100

C -0.16263000 4.26880400 1.67920100

H -0.44477800 3.42571000 2.31583100

H -0.07981200 5.16616300 2.30685700

H -0.96360500 4.43644700 0.95350400

C 2.17724700 3.64568800 2.15627900

H 1.91097700 2.69840300 2.63110900

H 3.21983000 3.60738100 1.83099600

H 2.10644400 4.42843200 2.92251900

C 1.68802500 5.29994600 0.35419200

H 2.65433700 5.18588300 -0.14553900

H 0.96991300 5.69303900 -0.37057900

H 1.81109600 6.06128900 1.13567700

Rh -0.55487200 0.72291100 1.07307800

C 5.41400300 -2.60088600 -0.68728700

Fe 4.24005600 -0.93085400 -1.32908500

C 5.30652400 -1.67479800 0.38348400

C 6.05860100 -1.94643800 -1.77173000

C 6.34323100 -0.61412700 -1.37247400

C 5.87699500 -0.44809100 -0.03996500

C 2.37254800 -1.48572000 -2.23246800

C 3.13358100 -0.70881100 -3.13586700

C 3.35032100 0.56150400 -2.53888600

C 2.71422900 0.58824600 -1.26471700

C 2.09513900 -0.69598000 -1.07560200

H 4.83810800 -1.85792100 1.34046700

H 6.24918600 -2.37089700 -2.74832500

H 6.78778000 0.15336400 -1.99220400

H 5.91461100 0.46350200 0.54146900

H 2.05456500 -2.51002700 -2.37873400

H 3.53002900 -1.04141100 -4.08574300

H 3.94227600 1.36061400 -2.96419400

H 5.03734400 -3.61421700 -0.68582300

C -3.75576500 3.36450400 1.07538500

C -4.54353700 3.96374000 0.09334200

C -4.85467500 3.27617400 -1.07710300

C -4.36760800 1.98040200 -1.26102900

C -3.58622500 1.37680700 -0.28520300

H -3.50867900 3.90689200 1.98501000

H -4.91353500 4.97393700 0.24254500

H -5.45753300 3.74923200 -1.84630300

H -4.56673900 1.44783700 -2.19005500

C -3.26425600 2.06836500 0.89833600

C -2.39535300 1.40110600 1.88195400

C -2.29774400 -0.03754900 1.91682200

H -2.21600100 1.93608700 2.81139900

C -2.86005300 0.06712000 -0.48957500

C -2.90140600 -0.77939600 0.78959700

H -3.30916000 -0.49799900 -1.32975300

O -1.52012100 0.30173600 -0.71846800

H -2.10280400 -0.55193800 2.85411600

H -2.47631700 -1.77304300 0.62608800

Cl 0.33962000 0.96015400 3.38463900

C -4.62731500 -1.91337800 2.22376800

H -4.35733800 -1.31404100 3.09101700

H -5.68815300 -2.16705600 2.25233300

H -4.00523600 -2.81278400 2.17039000

O -4.41015500 -1.09882500 1.05930000

H -4.75403200 -1.58473000 0.21217200

C -6.11078300 -1.26524900 -1.70901500

O -5.24262400 -2.20870600 -1.03139700

C -5.92318300 -3.46393100 -0.88330800

C -7.39054400 -3.07891000 -0.77381700

C -7.48264400 -1.94895600 -1.80771900

H -6.12174200 -0.35066200 -1.10792100

H -5.67089900 -1.03941700 -2.68329600

H -5.72837000 -4.07669300 -1.77124200

H -5.50203600 -3.96534800 -0.00812300

H -8.06559200 -3.91048000 -0.98479400

H -7.60636200 -2.70271600 0.23226600

H -7.62929700 -2.36778000 -2.80719400

H -8.30052500 -1.25457100 -1.60828100

**M4-a3**

P 0.82454400 0.83663200 0.23376200

C 0.12815400 1.82799100 1.62725100

C -0.18925000 3.18384300 1.48306800

C -0.28971500 1.16729900 2.79199500

C -0.91084000 3.86070200 2.46821900

H 0.13642200 3.72528300 0.59912100

C -1.00339700 1.84281200 3.77580800

H -0.06305400 0.11493600 2.91963900

C -1.32465800 3.19130800 3.61609400

H -1.13269300 4.91796700 2.34653200

H -1.31308600 1.31090900 4.67039600

H -1.87616500 3.71804200 4.38965500

C 1.79456400 2.12646200 -0.65933900

C 1.71469100 2.16885100 -2.05398200

C 2.61745700 3.04486600 0.00779200

C 2.45820000 3.11126500 -2.77103700

H 1.07396400 1.46036300 -2.57659000

C 3.34713400 3.98851900 -0.70679300

H 2.68730400 3.01857200 1.09297800

C 3.27167600 4.01976600 -2.10174500

H 2.39570800 3.12824100 -3.85500700

H 3.98239000 4.69496700 -0.17996400

H 3.84894800 4.75122000 -2.65954200

C 2.01778600 -2.38095400 -0.40606800

H 2.08906200 -1.75443500 -1.30762300

C 2.97836900 -3.56720900 -0.57507900

H 2.91485700 -4.27538700 0.25355900

H 2.78434500 -4.12129300 -1.49131000

H 4.01037700 -3.19722400 -0.62092200

P 0.14905700 -2.72036900 -0.36476300

C -0.22502800 -3.62010200 1.27142100

C 0.70261000 -4.78538600 1.64110000

H 1.69233700 -4.41080500 1.92164700

H 0.28925300 -5.29000900 2.52458400

H 0.82119800 -5.53315700 0.85692200

C -0.15202500 -2.61138100 2.43420500

H 0.85109400 -2.19691500 2.56690600

H -0.87573500 -1.80600700 2.29219300

H -0.40609800 -3.15093000 3.35759400

C -1.68883600 -4.08824100 1.20275000

H -1.84631200 -4.90157600 0.48941900

H -1.98552800 -4.45640400 2.19328200

H -2.35168300 -3.25309500 0.94308400

C -0.16504400 -3.92519900 -1.81410400

C -1.64895000 -3.76299300 -2.20363900

H -1.81459900 -2.77188700 -2.63485000

H -1.89685200 -4.51760100 -2.96203400

H -2.33682700 -3.88610100 -1.36222200

C 0.65031900 -3.48266800 -3.04337800

H 0.55772800 -2.41108500 -3.22982200

H 1.70755600 -3.74710800 -2.97485800

H 0.24640400 -4.00498300 -3.92041300

C 0.12372700 -5.41055600 -1.55900300

H 1.16350400 -5.59700900 -1.27429800

H -0.52570000 -5.84242500 -0.79316000

H -0.06287900 -5.95963700 -2.49124600

Rh -1.15655400 -0.52910600 -0.74218400

C 5.54554700 1.29644900 0.35520100

Fe 4.20847100 -0.31018300 0.79687300

C 4.95400100 0.84315200 -0.85323200

C 6.26675100 0.21264000 0.92586800

C 6.11571400 -0.91127000 0.07190000

C 5.30198200 -0.52086100 -1.02646000

C 2.75921400 0.18479100 2.29731800

C 3.52859100 -0.91754600 2.72909700

C 3.33817900 -1.96951200 1.79504900

C 2.44237400 -1.53370800 0.77659100

C 2.07699600 -0.17840000 1.09525400

H 4.31850400 1.42386300 -1.50841400

H 6.79483000 0.22601500 1.86970900

H 6.50771100 -1.90350700 0.25211200

H 4.97437200 -1.15882200 -1.83665100

H 2.69054500 1.14850300 2.78545300

H 4.18666100 -0.94025300 3.58720000

H 3.83615100 -2.92868800 1.82830600

H 5.43287000 2.28307900 0.78350500

C -4.93516400 -2.37530300 -0.88930400

C -5.83293300 -2.95121100 0.00884800

C -5.92714000 -2.47523100 1.31417900

C -5.10220600 -1.42682500 1.72553400

C -4.20135400 -0.85666600 0.83746000

H -4.87127600 -2.74113900 -1.91156500

H -6.46524600 -3.77279300 -0.31473700

H -6.62847300 -2.92388400 2.01096600

H -5.14207700 -1.06800800 2.75262500

C -4.11321900 -1.31991800 -0.48821900

C -3.17304600 -0.64522700 -1.39882100

C -2.75894400 0.70853900 -1.13446800

H -3.21592600 -0.94618000 -2.44277700

C -3.14058700 0.13533600 1.23965800

C -2.99747900 1.26451600 0.20956100

H -3.34288900 0.54362300 2.24617900

O -1.89517500 -0.47159000 1.20710900

H -2.58989700 1.38079100 -1.96877700

H -2.24110900 1.97705500 0.54872400

Cl -0.54297900 -0.23961100 -3.14397200

C -4.65695300 2.76783100 1.40248000

H -3.76704900 3.09506600 1.95013400

H -5.33101000 3.60091300 1.20048000

H -5.17903700 1.97780700 1.93888000

O -4.26400000 2.21134300 0.12744200

H -3.86434800 2.95628900 -0.43025800

C -1.56140400 3.69175800 -1.70127800

O -2.72400500 4.03085700 -0.90464700

C -2.81259800 5.45155800 -0.79344300

C -1.36229500 5.91146900 -0.74385500

C -0.68314900 4.95417200 -1.73555300

H -1.07501000 2.83723500 -1.22292500

H -1.88802600 3.38705600 -2.70012000

H -3.33470000 5.86171400 -1.66856600

H -3.38760700 5.68081600 0.10791900

H -1.24623800 6.96227400 -1.01756900

H -0.96396800 5.77125300 0.26484000

H -0.67655300 5.38484900 -2.74041200

H 0.35337700 4.73155000 -1.46444700

**M4-a4**

P 1.34367900 -1.15196200 0.02497500

C 0.82402500 -2.43410900 -1.19027900

C 0.54148200 -3.73614300 -0.76058200

C 0.48656400 -2.07017700 -2.50090200

C -0.06399400 -4.65534400 -1.61760500

H 0.78654500 -4.03557300 0.25467400

C -0.11571000 -2.98831500 -3.35564500

H 0.67863800 -1.05895700 -2.84330700

C -0.39897700 -4.28233900 -2.91717500

H -0.26754200 -5.66300400 -1.26694700

H -0.36715500 -2.69021900 -4.36940400

H -0.86124300 -4.99876200 -3.59063200

C 2.36833200 -2.16177800 1.17294600

C 2.19874300 -2.00424300 2.54938400

C 3.31853400 -3.07625600 0.69601400

C 2.98410100 -2.74371200 3.43854700

H 1.45053400 -1.30944800 2.92342100

C 4.09128100 -3.81581600 1.58255400

H 3.45192500 -3.20622200 -0.37578500

C 3.92754300 -3.64596100 2.95957400

H 2.84835900 -2.61104800 4.50761000

H 4.82443300 -4.52238500 1.20395800

H 4.53538400 -4.22085800 3.65192800

C 2.23364800 2.20068400 0.25457600

H 2.30073100 1.70696500 1.23520600

C 3.07850300 3.48144500 0.31716100

H 3.00743200 4.07203200 -0.59737300

H 2.78128200 4.12411900 1.14396600

H 4.13312900 3.21680900 0.46287400

P 0.35441900 2.36075200 0.05332200

C 0.04547200 3.03400700 -1.70274500

C 0.87776900 4.24755000 -2.14407500

H 1.91622700 3.95523100 -2.32959700

H 0.47569100 4.61076500 -3.09955700

H 0.86994000 5.08322700 -1.44378000

C 0.31678900 1.92137000 -2.73510300

H 1.37139800 1.63867900 -2.77610100

H -0.29658200 1.04191700 -2.53155400

H 0.04746600 2.31799600 -3.72451600

C -1.45540300 3.35899800 -1.79330600

H -1.73497900 4.23899000 -1.20725200

H -1.70670900 3.56851100 -2.84106500

H -2.06239800 2.50563400 -1.46749400

C -0.15748000 3.68690600 1.32995000

C -1.65156700 3.45925100 1.63176100

H -1.79819600 2.48257200 2.10222100

H -1.99007300 4.23601000 2.33070600

H -2.27938700 3.51104600 0.73753000

C 0.59604100 3.43079400 2.64826900

H 0.53725700 2.38317700 2.95122400

H 1.64437400 3.73465000 2.60761000

H 0.11762500 4.02898400 3.43474200

C 0.04270800 5.15505600 0.93259000

H 1.08184400 5.38947000 0.68565900

H -0.58619800 5.45067600 0.08842300

H -0.24146800 5.78391200 1.78653800

Rh -0.81416400 0.08016000 0.58874000

C 6.08593700 -1.19308000 0.22617400

Fe 4.65958500 0.20525400 -0.53039000

C 5.38685600 -0.60966700 1.31545500

C 6.75713800 -0.15387000 -0.47343500

C 6.46721200 1.07247200 0.18046400

C 5.61916700 0.78892200 1.28561300

C 3.34994800 -0.62665500 -2.01845700

C 4.05614800 0.47017900 -2.56133000

C 3.72212500 1.62005000 -1.79792800

C 2.79989200 1.24764400 -0.77820700

C 2.56002100 -0.16348200 -0.92214300

H 4.75035500 -1.13217400 2.01623500

H 7.34059600 -0.26811200 -1.37718700

H 6.79223600 2.05401200 -0.13823700

H 5.19505100 1.51411000 1.96769000

H 3.39258000 -1.65076000 -2.36637100

H 4.76500500 0.43227300 -3.37747900

H 4.14243600 2.60646900 -1.93807200

H 6.07708800 -2.24046000 -0.04274800

C -4.55697000 1.79670500 0.19585100

C -5.33216200 2.31536500 -0.84141400

C -5.28235600 1.74517400 -2.11240600

C -4.42143900 0.66944600 -2.34660000

C -3.63916100 0.15453300 -1.32020000

H -4.59418200 2.25326300 1.18336000

H -5.97658700 3.16999100 -0.65473400

H -5.88623300 2.14985200 -2.91856300

H -4.33193800 0.24745300 -3.34630600

C -3.71271100 0.70107600 -0.02156700

C -2.88007300 0.10295300 1.03252100

C -2.37030000 -1.24623500 0.89686300

H -3.02564000 0.48341900 2.04145300

C -2.52418100 -0.84683200 -1.53098200

C -2.55145800 -1.91073500 -0.42356800

H -2.61437500 -1.31687600 -2.52717900

O -1.30354100 -0.21484800 -1.40819000

H -2.24528200 -1.87690300 1.77427100

H -1.82386600 -2.70244200 -0.61892200

Cl -0.49372600 0.16049900 3.05810000

C -4.29908800 -3.23285800 -1.66496000

H -3.43374700 -3.72357800 -2.11462700

H -5.08068000 -3.95969300 -1.44171100

H -4.67372700 -2.43986000 -2.31711500

O -3.86758500 -2.67839300 -0.41113400

H -4.67059000 -2.18446900 0.08413100

C -5.74299600 -1.70915300 2.17898600

O -5.85507900 -1.84126800 0.74513500

C -6.75716000 -0.84606600 0.20018500

C -7.26128100 -0.04485200 1.40218600

C -6.18981400 -0.28638000 2.47278600

H -6.39998900 -2.45501500 2.63944900

H -4.70366600 -1.91048900 2.44771900

H -6.19362800 -0.23251900 -0.50534600

H -7.55632500 -1.37634100 -0.32419700

H -7.37048700 1.01270400 1.15158200

H -8.23021000 -0.42524100 1.73952900

H -5.34927500 0.39714000 2.33964700

H -6.57372800 -0.17785500 3.48921400

**M4-a5**

P 0.92445800 0.94212900 0.32384900

C 0.35993700 1.86895700 1.81551600

C 0.09534700 3.24235900 1.76807800

C 0.02310400 1.16341500 2.97947700

C -0.48963900 3.89642900 2.85422600

H 0.36265700 3.81666200 0.88538200

C -0.55512000 1.81704900 4.06229100

H 0.20783300 0.09608200 3.02914400

C -0.82129000 3.18485100 4.00240600

H -0.67042400 4.96700700 2.80304300

H -0.80469200 1.25274900 4.95569600

H -1.27435000 3.69178100 4.84897800

C 1.87522800 2.25285600 -0.55657600

C 1.68667700 2.41342800 -1.93092400

C 2.78587700 3.08209700 0.11213900

C 2.40732200 3.38818500 -2.62767700

H 0.97648800 1.77545500 -2.45378700

C 3.49349900 4.05732900 -0.58039300

H 2.94168700 2.95882600 1.18154500

C 3.30645000 4.20974500 -1.95628400

H 2.25959400 3.49918600 -3.69782100

H 4.19664500 4.69526400 -0.05228100

H 3.86445600 4.96742400 -2.49838300

C 2.05197900 -2.22288500 -0.61999400

H 2.08005700 -1.52746300 -1.47187200

C 2.99369200 -3.39322000 -0.94018500

H 2.95431900 -4.17977300 -0.18449200

H 2.76211000 -3.85110300 -1.89994600

H 4.02648100 -3.02684700 -0.98778900

P 0.18803700 -2.55775400 -0.48680200

C -0.08357900 -3.58981800 1.09080700

C 0.86195700 -4.77907100 1.31126800

H 1.86965700 -4.42992200 1.55800400

H 0.50240100 -5.34974700 2.17819400

H 0.92600200 -5.46647500 0.46687600

C 0.05791800 -2.67311800 2.32099200

H 1.07179800 -2.28097300 2.43594800

H -0.65652200 -1.84817300 2.27467300

H -0.16068700 -3.27799000 3.21228100

C -1.54552700 -4.06779700 1.07076300

H -1.73079900 -4.83961400 0.31834900

H -1.78712900 -4.49879100 2.05086900

H -2.23119000 -3.22955100 0.89678400

C -0.20884900 -3.63673100 -2.01332500

C -1.71612600 -3.48297800 -2.29110700

H -1.93657500 -2.45478100 -2.59029000

H -1.99072600 -4.15121300 -3.11817600

H -2.34127000 -3.73322000 -1.42971400

C 0.51160800 -3.05554800 -3.24354300

H 0.35307500 -1.97835200 -3.33263300

H 1.58388100 -3.26223900 -3.25432100

H 0.08514600 -3.52656100 -4.13884200

C 0.12457600 -5.13023200 -1.90742600

H 1.17862800 -5.31359600 -1.68263800

H -0.48024900 -5.64048900 -1.15303500

H -0.09337300 -5.60246700 -2.87437100

Rh -1.14592100 -0.32604300 -0.58913800

C 5.64743600 1.34516400 0.17283400

Fe 4.32858900 -0.27986600 0.60686100

C 4.99721500 0.94700500 -1.02472400

C 6.39315900 0.23758000 0.65898200

C 6.19870000 -0.84720800 -0.23530900

C 5.33404700 -0.40774200 -1.27493400

C 2.96882600 0.12073500 2.22125700

C 3.74777600 -1.01728800 2.52638800

C 3.49235100 -1.99570400 1.53013200

C 2.54727600 -1.47552800 0.59969600

C 2.21263300 -0.14687900 1.03825000

H 4.32848600 1.55525800 -1.61930300

H 6.96702700 0.20976500 1.57534300

H 6.59717600 -1.84628600 -0.11810300

H 4.96787800 -1.00851700 -2.09694100

H 2.93981700 1.04397700 2.78535200

H 4.45385300 -1.10847100 3.34073600

H 3.97950200 -2.95875100 1.46087300

H 5.55939300 2.31183500 0.64886500

C -4.78581700 -2.37938900 -0.58975700

C -5.54717300 -3.09815900 0.33091200

C -5.53782600 -2.74612500 1.67864500

C -4.75333600 -1.67289200 2.10606400

C -3.98805200 -0.95829200 1.19393100

H -4.79829600 -2.65105000 -1.64300000

H -6.15263800 -3.93435600 -0.00616200

H -6.13006400 -3.30839800 2.39398100

H -4.72236400 -1.40014600 3.15896700

C -3.99618600 -1.30594200 -0.17011900

C -3.18591600 -0.49788100 -1.09724500

C -2.82488900 0.85872400 -0.75624700

H -3.29782000 -0.72433200 -2.15532300

C -2.99190500 0.10704100 1.58665100

C -3.03677400 1.31278600 0.63565700

H -3.16003500 0.43842900 2.62546500

O -1.70823200 -0.39091200 1.41873100

H -2.73251500 1.59304600 -1.54743300

H -2.31170500 2.05615800 0.97959800

Cl -0.77415800 0.17988600 -3.01561000

C -5.58418300 1.57269100 0.32763000

H -5.90314100 0.86473600 1.08926300

H -6.29550700 2.39378800 0.23285900

H -5.43347000 1.06148800 -0.62581100

O -4.33863000 2.16308800 0.77154700

H -4.12141100 2.98702700 0.17357800

C -3.67574900 3.90144500 -2.13941300

O -3.66819900 4.08917900 -0.70547600

C -2.39345900 4.65391800 -0.34064300

C -1.40242300 3.97911900 -1.27707600

C -2.20008600 3.85771600 -2.58816900

H -4.22125500 2.97609500 -2.34025300

H -4.22076600 4.73998300 -2.58297300

H -2.44130900 5.73807300 -0.50101900

H -2.23413200 4.44992600 0.72141500

H -0.47860200 4.55110400 -1.39707700

H -1.13304000 2.98461100 -0.90181200

H -1.98654400 4.69721700 -3.25459100

H -1.95069300 2.92942400 -3.11135400

**M4-a6**

P 1.00117800 -1.09380200 0.31661800

C 0.11043800 -2.47216600 -0.53843600

C -0.20755300 -3.65094500 0.14759200

C -0.32642700 -2.33624700 -1.86310300

C -0.88557600 -4.69133900 -0.48936300

H 0.10433400 -3.77682800 1.18053700

C -0.99634400 -3.37646900 -2.50112400

H -0.11641900 -1.42033500 -2.40485300

C -1.26380100 -4.56635100 -1.82273200

H -1.09551500 -5.60713800 0.05596800

H -1.29614900 -3.26093200 -3.53930900

H -1.76392300 -5.38749100 -2.32984100

C 2.02500400 -2.07783000 1.49412600

C 2.03761100 -1.72233100 2.84345800

C 2.78868900 -3.17455800 1.06785800

C 2.82291000 -2.44027100 3.75054300

H 1.45126000 -0.87117900 3.17903000

C 3.55949800 -3.89266500 1.97288800

H 2.78260200 -3.46506300 0.02029500

C 3.58328400 -3.52061200 3.31934700

H 2.83424000 -2.14548900 4.79545200

H 4.14766500 -4.74032000 1.63238500

H 4.19311300 -4.07734800 4.02471700

C 2.40863800 2.03644700 -0.45112200

H 2.59011400 1.81114000 0.61032900

C 3.40886400 3.11608100 -0.88883400

H 3.24190000 3.44483600 -1.91662500

H 3.35995400 3.99719000 -0.25365100

H 4.42860500 2.71659800 -0.82515200

P 0.56877100 2.48326900 -0.39906200

C -0.01689600 2.63949200 -2.20554000

C 0.87198300 3.45497400 -3.15478300

H 1.80859900 2.92429300 -3.35343000

H 0.35129000 3.55494700 -4.11681800

H 1.10828100 4.45837400 -2.80034300

C -0.13749000 1.23082600 -2.81779900

H 0.83379900 0.74238500 -2.93241300

H -0.80794800 0.60636200 -2.22313300

H -0.56574500 1.34233000 -3.82422400

C -1.44255700 3.21521600 -2.16707300

H -1.46892700 4.27139600 -1.88683900

H -1.88355400 3.13111400 -3.16886000

H -2.07397700 2.64521300 -1.47376700

C 0.49989900 4.21701700 0.41355200

C -0.92046200 4.37221300 0.98799600

H -1.08639000 3.63010400 1.77372100

H -1.01577600 5.37346000 1.42883700

H -1.70746700 4.25626900 0.23872300

C 1.45006700 4.28146600 1.62408600

H 1.30980900 3.43268100 2.29536300

H 2.50438300 4.34346900 1.34855000

H 1.21337500 5.19587000 2.18381200

C 0.79875500 5.41686200 -0.49558700

H 1.79192500 5.36600300 -0.95045700

H 0.06110000 5.53689300 -1.29292000

H 0.76504100 6.32708700 0.11742500

Rh -0.66693500 0.69260700 1.00592700

C 5.70143500 -1.87262200 -0.02476400

Fe 4.34879800 -0.49144200 -0.93879100

C 5.22069300 -0.97238500 0.96240200

C 6.38302700 -1.11690800 -1.01682200

C 6.31940500 0.25148900 -0.64426800

C 5.59866400 0.33981200 0.57799300

C 2.72565900 -1.42244900 -1.99894700

C 3.48312800 -0.63327900 -2.89108300

C 3.43186500 0.70718000 -2.42941900

C 2.64172800 0.76477900 -1.24474700

C 2.18975200 -0.57695300 -0.97832900

H 4.63171200 -1.23517300 1.83115300

H 6.82813000 -1.50852400 -1.92149300

H 6.70707300 1.08406400 -1.21637600

H 5.34607600 1.24987700 1.10655600

H 2.56735200 -2.49046600 -2.07176200

H 4.04500300 -0.99083300 -3.74342300

H 3.95752800 1.53846800 -2.87800800

H 5.54281000 -2.94191800 -0.03532600

C -4.11419900 3.04515800 1.21539600

C -5.10291600 3.45681600 0.32343900

C -5.48206600 2.63640300 -0.73656600

C -4.86078600 1.39581000 -0.89867500

C -3.88127300 0.97573800 -0.00700700

H -3.81922600 3.68985100 2.04006100

H -5.57770700 4.42443300 0.45655000

H -6.24327100 2.96431900 -1.43763500

H -5.11930100 0.76240400 -1.74668700

C -3.48705300 1.80549700 1.06065400

C -2.44427700 1.31441100 1.97507800

C -2.22603600 -0.10517300 2.13744400

H -2.20694600 1.95061300 2.82463900

C -3.07728200 -0.29390600 -0.17674500

C -2.90020400 -1.00449800 1.17729300

H -3.59553900 -0.96225500 -0.89138100

O -1.79954800 -0.01047800 -0.60475900

H -1.91307800 -0.50852300 3.09698700

H -2.38677700 -1.96143900 1.05103900

Cl 0.49084400 1.32959400 3.10177500

C -5.18646100 -0.60479200 2.37485200

H -5.71773400 0.00095900 1.63858900

H -5.86719800 -1.22209600 2.96136800

H -4.58944200 0.03362100 3.02401500

O -4.27207100 -1.51363300 1.72239900

H -4.73607300 -2.07183100 0.97758800

C -4.26277400 -3.67747900 -0.77607200

O -5.27577900 -2.88080100 -0.11444500

C -6.09472900 -2.19299700 -1.08780700

C -5.45842800 -2.49091900 -2.44329300

C -4.77842100 -3.84203200 -2.19751300

H -3.30891500 -3.13764500 -0.76021800

H -4.16121100 -4.61026400 -0.21790300

H -7.10791400 -2.59815600 -1.00757400

H -6.10651900 -1.12810100 -0.83505200

H -6.19862500 -2.52015100 -3.24546800

H -4.70788100 -1.73293900 -2.69085400

H -5.50699000 -4.65724000 -2.25016400

H -3.96788500 -4.04565800 -2.89962200

**TS_3-4_b1**

P -1.81051900 1.06739200 0.21638600

C -1.60732100 2.64393700 -0.70969800

C -1.75198700 3.86510900 -0.04133300

C -1.11123700 2.64667100 -2.01850300

C -1.43104700 5.06470800 -0.67371600

H -2.11756800 3.88252500 0.98164500

C -0.78809800 3.84471000 -2.64772200

H -0.96287500 1.70676500 -2.53971000

C -0.94664900 5.05773100 -1.97842300

H -1.55719300 6.00321300 -0.14225300

H -0.40431900 3.82902400 -3.66355000

H -0.69502300 5.99132700 -2.47306200

C -3.18785500 1.50102300 1.35742500

C -3.09397600 1.12538400 2.69834700

C -4.32640700 2.18673500 0.91178800

C -4.13836700 1.41851500 3.58008400

H -2.20287600 0.61088400 3.05129100

C -5.35843700 2.48483400 1.79302100

H -4.40132100 2.48835700 -0.13063800

C -5.26724700 2.09456600 3.13122700

H -4.05824200 1.11923700 4.62075200

H -6.23677200 3.01849100 1.44056200

H -6.07621300 2.32377500 3.81869600

C -1.68959900 -2.39598000 -0.22759500

H -2.00019400 -2.15472000 0.79977100

C -2.10288800 -3.84606900 -0.51668700

H -1.76145300 -4.18980100 -1.49482600

H -1.71073500 -4.53871200 0.22616500

H -3.19689600 -3.92505100 -0.49781700

P 0.15449800 -1.96392400 -0.14998700

C 0.82798800 -2.10240900 -1.92886600

C 0.43276200 -3.34786600 -2.73350100

H -0.63021400 -3.31422700 -2.99089200

H 0.99000700 -3.34652100 -3.68018000

H 0.63879000 -4.29204700 -2.22659500

C 0.35467400 -0.87326400 -2.73101700

H -0.72702400 -0.87121000 -2.88455200

H 0.66129900 0.06049900 -2.25189300

H 0.82390000 -0.92301700 -3.72404700

C 2.35915800 -1.98596300 -1.84761200

H 2.83168700 -2.85609600 -1.38073100

H 2.76149400 -1.89573800 -2.86505900

H 2.63607000 -1.07613100 -1.29945500

C 0.92915800 -3.34647100 0.92078600

C 2.23896100 -2.76757500 1.49081000

H 2.01742900 -1.88070300 2.09565200

H 2.71818400 -3.52331400 2.12890400

H 2.94881400 -2.49270300 0.70295300

C 0.02194500 -3.60532100 2.13749100

H -0.24690200 -2.67389300 2.64219500

H -0.88784400 -4.15231300 1.88305200

H 0.57932100 -4.22393900 2.85343300

C 1.23348000 -4.68383400 0.23530900

H 0.34252000 -5.14384000 -0.20070400

H 1.98572000 -4.58903400 -0.55319100

H 1.63001800 -5.38109100 0.98544100

Rh 0.50918100 0.41200400 0.91103800

C -6.34955800 -0.25754900 -0.23963700

Fe -4.51476800 -1.03034400 -1.01715000

C -5.61072200 -0.84886700 0.81877900

C -6.62301400 -1.26541400 -1.20336200

C -6.04793500 -2.47911500 -0.74276800

C -5.42147500 -2.21920300 0.50679700

C -3.38422500 0.41006800 -2.14023700

C -3.67785100 -0.70726400 -2.95365500

C -3.08543800 -1.84762200 -2.35066000

C -2.41542700 -1.44627100 -1.15863700

C -2.59616800 -0.02435000 -1.03093100

H -5.22172400 -0.33659500 1.68847800

H -7.13143700 -1.12286600 -2.14738700

H -6.04251800 -3.42132200 -1.27470500

H -4.86415100 -2.93072100 1.10195000

H -3.69751300 1.43067000 -2.31814800

H -4.28983500 -0.70556900 -3.84539900

H -3.18092400 -2.86280100 -2.71086500

H -6.62021400 0.78659800 -0.31382400

C 5.29975200 1.67705500 -0.69711700

C 5.67881600 2.62443500 -1.64373700

C 4.76005200 3.57848900 -2.07106800

C 3.46833700 3.57199200 -1.55269300

C 3.08469300 2.63379000 -0.59856200

H 6.03459000 0.94601700 -0.37321000

H 6.68876300 2.61613400 -2.04282600

H 5.04842300 4.31908500 -2.81115900

H 2.73106100 4.29387400 -1.89554100

C 4.01047200 1.67570200 -0.16127300

C 3.58349000 0.67817200 0.88568200

C 2.38815000 1.02498000 1.68665700

C 1.65327500 2.49578500 -0.13597200

C 1.55553400 2.13473900 1.34439700

H 1.08297000 3.40880900 -0.37464900

O 1.14148400 1.36248600 -0.78738300

H 2.37856700 0.67415000 2.71289700

H 1.13316600 2.80799300 2.08386200

Cl 0.02605800 -0.32171800 3.28315400

O 4.87173200 0.60190800 1.88051100

H 3.60468500 -0.36374100 0.54680100

H 5.54378600 0.04263300 1.36681800

C 4.65344000 -0.11922300 3.11920600

H 4.19035400 0.56283300 3.82660600

H 4.01198800 -0.98647700 2.94013300

H 5.63733100 -0.42939600 3.47195700

C 5.82770900 -1.85503500 -0.36131100

O 6.42070000 -1.09521200 0.71867300

C 7.80985300 -0.82505200 0.43929000

C 7.99612300 -1.17414100 -1.03267800

C 7.00520900 -2.32912600 -1.20429600

H 5.23836900 -2.66128700 0.08141500

H 5.16851500 -1.19211400 -0.93587300

H 8.00827800 0.22424800 0.67572000

H 8.41845200 -1.46160400 1.09008400

H 7.70458800 -0.32830400 -1.66498200

H 9.02697600 -1.44566700 -1.26783800

H 6.72006200 -2.50386100 -2.24334200

H 7.42200900 -3.25511200 -0.79612500

**TS_3-4_b2**

P -1.22869200 -1.16164800 -0.05232300

C -0.53320800 -2.42091300 1.09539200

C -0.09075200 -3.64893200 0.59069600

C -0.25111500 -2.09842000 2.42788400

C 0.59842300 -4.54608700 1.40482200

H -0.28765000 -3.90953900 -0.44573200

C 0.43902800 -2.99340600 3.23905700

H -0.55991000 -1.13722400 2.82518400

C 0.86643500 -4.21980600 2.73126700

H 0.92580500 -5.49879200 0.99902200

H 0.64935200 -2.72858100 4.27110900

H 1.40324000 -4.91702500 3.36807900

C -2.17708000 -2.23521000 -1.20928900

C -2.07975400 -2.01323300 -2.58347600

C -3.00090300 -3.26523400 -0.73396100

C -2.81106700 -2.80679000 -3.47215100

H -1.43059600 -1.22417300 -2.95542500

C -3.72005500 -4.05690000 -1.62052700

H -3.07843300 -3.44514000 0.33577800

C -3.62882300 -3.82481000 -2.99503200

H -2.73180800 -2.62410200 -4.53950700

H -4.35597100 -4.85318400 -1.24392600

H -4.19555800 -4.44108200 -3.68674800

C -2.51529900 2.08815600 -0.08980800

H -2.57852900 1.64362600 -1.09402300

C -3.49293600 3.27093500 -0.03486300

H -3.42804500 3.82173000 0.90521800

H -3.31915900 3.98124200 -0.84137800

H -4.52049100 2.90031900 -0.13527400

P -0.65493000 2.42908600 0.03072400

C -0.31154400 2.98823800 1.81857200

C -1.25483900 4.04565700 2.40648000

H -2.24329500 3.61477900 2.59364600

H -0.85794200 4.36918900 3.37814600

H -1.37296400 4.93259100 1.78230700

C -0.37389800 1.75837300 2.74721600

H -1.38355300 1.34808600 2.82986800

H 0.31727700 0.97667200 2.42281000

H -0.07404100 2.08757200 3.75219300

C 1.14267700 3.48443400 1.86667000

H 1.29011800 4.42942000 1.33637800

H 1.42498300 3.64812300 2.91437200

H 1.81971900 2.72483900 1.45535900

C -0.35106500 3.89244400 -1.16089200

C 1.14160700 3.85118600 -1.54393800

H 1.38094800 2.89525800 -2.02287100

H 1.34601100 4.66190200 -2.25614600

H 1.79827700 3.99335500 -0.68002300

C -1.12867900 3.64282000 -2.46617000

H -0.93279500 2.64522000 -2.86651900

H -2.20657600 3.77815200 -2.35671500

H -0.78733200 4.37409500 -3.21063400

C -0.68793900 5.29698800 -0.64452500

H -1.73417000 5.39397600 -0.34113000

H -0.05991400 5.59531800 0.19981800

H -0.51109600 6.01815300 -1.45352600

Rh 0.67975600 0.30560700 -0.65577500

C -5.94944600 -1.71127600 -0.13350300

Fe -4.65691600 -0.20739400 0.66220100

C -5.35195700 -0.99924200 -1.20657800

C -6.70382100 -0.78899900 0.64126900

C -6.56750600 0.49445900 0.04956400

C -5.72952100 0.36303500 -1.09165000

C -3.21296300 -0.96051400 2.06247600

C -4.01612800 0.02003500 2.68616600

C -3.83935400 1.23753900 1.97798300

C -2.92197900 1.02400400 0.90883100

C -2.51972200 -0.35685400 0.96849300

H -4.68718100 -1.41268400 -1.95265500

H -7.24365500 -1.01384500 1.55122500

H -6.98144300 1.41774600 0.43297600

H -5.40243500 1.16614900 -1.73919400

H -3.12710000 -1.99847400 2.35699200

H -4.68832700 -0.13987000 3.51827600

H -4.36593900 2.15959800 2.18293600

H -5.81788900 -2.76389500 0.07508500

C 5.22409800 1.42335100 1.46918700

C 5.79330600 0.88332700 2.61986800

C 5.33204100 -0.33833900 3.10967900

C 4.28486700 -0.99654500 2.46727200

C 3.70310500 -0.45953900 1.32014300

H 5.59390800 2.36341700 1.06739400

H 6.59341000 1.41078900 3.12933500

H 5.77516700 -0.76589400 4.00437800

H 3.88613800 -1.92573700 2.86841700

C 4.19377700 0.75011900 0.80890200

C 3.66620600 1.27122800 -0.49463500

C 2.74114100 0.42146600 -1.25142600

C 2.42650100 -1.01868600 0.73007100

C 2.35175500 -0.88142300 -0.79205700

H 2.27808300 -2.06365500 1.05642100

O 1.41029000 -0.17205700 1.19080800

H 2.66879400 0.64285200 -2.31352500

H 2.28131900 -1.74669000 -1.44712400

Cl 0.22657500 0.50825100 -3.13354000

O 5.07902800 1.39190100 -1.34687700

H 3.44156300 2.34117900 -0.50019900

H 5.51772200 0.44895000 -1.35542800

C 4.95411800 1.88349600 -2.69020100

H 4.52554600 1.12541700 -3.34855100

H 4.30162700 2.75794100 -2.66063400

H 5.95154100 2.16805600 -3.02698300

C 6.85468900 -1.32016200 -0.26298600

O 6.17164200 -0.86981500 -1.45834700

C 5.38391900 -1.94108900 -2.01998300

C 5.18840100 -2.92159500 -0.87428700

C 6.52164400 -2.80575300 -0.12976000

H 7.92170400 -1.12420400 -0.39360300

H 6.47718100 -0.74054400 0.58358000

H 4.45392800 -1.50954500 -2.39708200

H 5.95105300 -2.38416300 -2.84663400

H 4.36356500 -2.59014500 -0.23638400

H 4.97623000 -3.93371200 -1.22543900

H 6.45031200 -3.10625900 0.91785200

H 7.28663500 -3.41888400 -0.61657300

**TS_3-4_b3**

P -1.77534600 1.04669100 0.27647900

C -1.68103500 2.66245300 -0.59921300

C -1.83505700 3.85167000 0.12223000

C -1.25385800 2.73280000 -1.93022800

C -1.58842600 5.08497300 -0.47817800

H -2.14877300 3.81802300 1.16192300

C -1.00364800 3.96418400 -2.52737800

H -1.10049600 1.81924000 -2.49507200

C -1.16974700 5.14475300 -1.80407100

H -1.72103600 5.99783500 0.09489100

H -0.67112200 3.99993100 -3.56062700

H -0.97650200 6.10501900 -2.27334900

C -3.11232500 1.38661600 1.49513700

C -2.94575800 0.97522400 2.81813400

C -4.29418800 2.04116300 1.12061000

C -3.95997800 1.20294800 3.75277400

H -2.02326300 0.48203500 3.11562500

C -5.29625400 2.27408800 2.05449100

H -4.42694400 2.37124000 0.09278800

C -5.13168100 1.84894800 3.37487900

H -3.82270700 0.87571700 4.77891600

H -6.20750500 2.78499300 1.75620100

H -5.91674700 2.02679300 4.10387100

C -1.52853500 -2.40345900 -0.32131900

H -1.79981700 -2.23147500 0.73073300

C -1.88748800 -3.85574400 -0.66692900

H -1.57751300 -4.13413400 -1.67563000

H -1.43008200 -4.56410800 0.02192700

H -2.97504000 -3.98616900 -0.60360600

P 0.29235400 -1.88234700 -0.31132600

C 0.88403100 -1.90600300 -2.12254000

C 0.52456000 -3.13994700 -2.96163600

H -0.54944700 -3.15993600 -3.16925500

H 1.03465400 -3.06572100 -3.93170500

H 0.80931200 -4.09030400 -2.50707600

C 0.29483500 -0.67866700 -2.84627500

H -0.78792000 -0.75465600 -2.97136800

H 0.54316100 0.25024300 -2.32628800

H 0.73689300 -0.64023900 -3.85213000

C 2.40798400 -1.69789600 -2.10844300

H 2.95291900 -2.54940700 -1.68651900

H 2.75560600 -1.56215100 -3.14064900

H 2.65847900 -0.78509900 -1.55228400

C 1.18688500 -3.26386400 0.66234200

C 2.48841800 -2.64338300 1.20403700

H 2.26613200 -1.79154300 1.85577100

H 3.02634000 -3.40337600 1.78971700

H 3.15188900 -2.30827200 0.39760300

C 0.34612200 -3.63128700 1.89889600

H 0.04722200 -2.74244800 2.45950600

H -0.54152500 -4.21624000 1.65161800

H 0.96516200 -4.25087200 2.56099000

C 1.53333400 -4.54931200 -0.09918000

H 0.65102300 -5.03689800 -0.52372000

H 2.24970900 -4.37873500 -0.90926600

H 1.99446100 -5.25813100 0.60217000

Rh 0.57032200 0.43742400 0.82117400

C -6.26514200 -0.46317800 0.00172200

Fe -4.44322500 -1.12481700 -0.89681900

C -5.44459800 -1.05126300 0.99971300

C -6.54449900 -1.45300200 -0.97899600

C -5.89168700 -2.65207600 -0.58928500

C -5.21084900 -2.40159900 0.63352200

C -3.43815500 0.40526500 -2.01919600

C -3.72921400 -0.69070900 -2.86171500

C -3.05819200 -1.82663500 -2.33867400

C -2.34156800 -1.44428100 -1.16765900

C -2.57254800 -0.03714900 -0.97193500

H -5.03070600 -0.54714900 1.86261800

H -7.10877900 -1.30507500 -1.88990200

H -5.87277800 -3.57639200 -1.15146900

H -4.59168400 -3.10398000 1.17596800

H -3.80385900 1.41673700 -2.13818000

H -4.38737600 -0.67987800 -3.71998700

H -3.12989400 -2.83008800 -2.73546100

H -6.58656300 0.56880400 -0.02406400

C 5.18732200 1.85084900 -1.04865900

C 5.48193700 2.83338200 -1.98878700

C 4.52388400 3.79646500 -2.30078100

C 3.27979600 3.76625500 -1.67808600

C 2.98177700 2.79217500 -0.72798700

H 5.94707600 1.11469600 -0.79183800

H 6.45237000 2.84717400 -2.47500400

H 4.74622800 4.56445500 -3.03570600

H 2.51436700 4.49528000 -1.93316800

C 3.94536500 1.82724000 -0.40979500

C 3.64226300 0.82046300 0.66061400

C 2.49039900 1.07016500 1.53448900

C 1.59133300 2.60836000 -0.16578300

C 1.59445000 2.16269700 1.29758100

H 0.99117200 3.52136300 -0.31667800

O 1.06076900 1.50469500 -0.85155300

H 2.58102200 0.64826500 2.53268500

H 1.19176300 2.78028200 2.09493000

Cl 0.21496200 -0.41194600 3.16523100

O 4.96565500 0.85720600 1.67674300

H 3.80785100 -0.22282600 0.37593300

C 5.38593500 2.14214400 2.16221700

H 5.91578500 2.69538100 1.38526100

H 4.47214500 2.66656300 2.44605300

H 6.01478500 1.97438200 3.03667300

C 6.07108600 -2.14505800 1.54268500

O 6.37582100 -1.04765400 0.65093600

C 6.30859400 -1.50679700 -0.70831200

C 6.72226200 -2.96713500 -0.62186000

C 6.02628800 -3.40746700 0.67085100

H 6.86689600 -2.17621900 2.29160800

H 5.11573700 -1.94535000 2.04146700

H 5.28309200 -1.39749800 -1.09118700

H 6.98116300 -0.88410900 -1.30160700

H 6.40848700 -3.54390800 -1.49416900

H 7.80991200 -3.03891800 -0.52484300

H 4.99035200 -3.68978900 0.46356700

H 6.51861400 -4.25382800 1.15343200

H 5.68164500 0.28939100 1.25246500

**TS_3-4_b4**

P -1.45395700 0.97700100 0.59449200

C -1.33646200 2.79188700 0.31320900

C -1.19770600 3.64864000 1.41091600

C -1.18263400 3.31541500 -0.97537200

C -0.93574800 5.00480900 1.22665500

H -1.29633700 3.25617700 2.41936700

C -0.91839300 4.66932100 -1.15815100

H -1.25259100 2.65751100 -1.83546600

C -0.79456700 5.51857600 -0.05924600

H -0.84090800 5.65634500 2.09024000

H -0.80155100 5.06114600 -2.16419700

H -0.59014700 6.57499200 -0.20653500

C -2.50497000 0.94221800 2.10648200

C -2.16623600 0.10235700 3.16813400

C -3.64819900 1.74800500 2.20408000

C -2.97345500 0.06125000 4.30962500

H -1.27194300 -0.51286500 3.10552200

C -4.44316200 1.71089300 3.34264200

H -3.91453500 2.40825300 1.38227400

C -4.10705400 0.86114900 4.39910400

H -2.70411800 -0.59669000 5.13026500

H -5.32587700 2.34066500 3.40812800

H -4.72932100 0.82979100 5.28852100

C -1.69552300 -2.04760100 -1.13494900

H -1.72979600 -2.22294900 -0.04942600

C -2.27946400 -3.28651900 -1.82863400

H -2.20353500 -3.22969000 -2.91615100

H -1.78128000 -4.20260600 -1.51481300

H -3.34160500 -3.38031000 -1.57044800

P 0.13646900 -1.62956200 -1.37503000

C 0.34023100 -1.03751200 -3.17364000

C -0.34729000 -1.87625100 -4.25887500

H -1.43439500 -1.76891500 -4.19521600

H -0.04492900 -1.49156800 -5.24219700

H -0.09804400 -2.93839900 -4.22699600

C -0.21148700 0.39783700 -3.28746600

H -1.29740500 0.43538800 -3.17010100

H 0.26118000 1.06750000 -2.56428400

H 0.01929600 0.76249900 -4.29829700

C 1.84820300 -0.94071300 -3.45469900

H 2.33538000 -1.91843100 -3.51174700

H 1.99392900 -0.44497700 -4.42254300

H 2.34457600 -0.32842100 -2.69128000

C 1.04444100 -3.29667900 -1.15460000

C 2.49961900 -2.95743600 -0.78309500

H 2.52149400 -2.33511900 0.11971200

H 3.04009900 -3.89471000 -0.58396400

H 3.02823300 -2.43110100 -1.58378000

C 0.45550000 -4.01755700 0.07144600

H 0.39941200 -3.35546500 0.93948200

H -0.53282000 -4.44260300 -0.11360200

H 1.11935900 -4.85472700 0.32917000

C 1.04901900 -4.26289100 -2.34552900

H 0.03987000 -4.53193900 -2.66919300

H 1.58800700 -3.86181000 -3.20859300

H 1.55674500 -5.18940600 -2.04469500

Rh 0.87078500 0.13794200 0.40506300

C -6.09155500 -0.16767900 0.75605800

Fe -4.51393500 -0.54949500 -0.63219600

C -5.15738000 -1.05493000 1.35223000

C -6.61456300 -0.79531800 -0.40708200

C -5.99803600 -2.06825400 -0.53211400

C -5.09617700 -2.22641500 0.55537600

C -3.57943800 1.23781800 -1.37443100

C -4.13684800 0.50950900 -2.44903400

C -3.50476700 -0.76082400 -2.48388100

C -2.54663100 -0.83057300 -1.43165300

C -2.58610300 0.43081900 -0.73974100

H -4.56345700 -0.85494400 2.23340800

H -7.31847800 -0.36106100 -1.10418000

H -6.15057700 -2.77191700 -1.33982300

H -4.44808400 -3.07559700 0.72918700

H -3.85145800 2.24184000 -1.07582000

H -4.93933600 0.83844200 -3.09523900

H -3.75303100 -1.56312900 -3.16512200

H -6.32877800 0.82734400 1.10674200

C 5.24870800 1.90536300 -1.72159700

C 5.44516800 3.10928100 -2.38966100

C 4.52336600 4.14446300 -2.23113800

C 3.40715900 3.96715400 -1.41960400

C 3.20573300 2.76857600 -0.73811400

H 5.96892200 1.09695300 -1.82593800

H 6.31023300 3.24011800 -3.03216200

H 4.67294100 5.08657500 -2.75032600

H 2.66710600 4.75680700 -1.31399800

C 4.13882800 1.73519200 -0.89120900

C 3.97773400 0.47916300 -0.09781400

C 2.92940600 0.43463000 0.92505700

C 1.91607900 2.47649200 -0.00361000

C 2.10215000 1.56945700 1.21389600

H 1.40471500 3.41744800 0.25891700

O 1.15137800 1.69022400 -0.88226300

H 3.10754200 -0.29803200 1.70748500

H 1.88001600 1.90224800 2.22452700

Cl 0.87730900 -1.45131900 2.39635800

O 5.42576100 0.36532900 0.72660000

H 4.13248300 -0.44152800 -0.66753000

C 5.70163300 1.46677900 1.61189900

H 5.98423800 2.31792800 0.99442800

H 4.81292600 1.70040200 2.20409900

H 6.53108400 1.16361900 2.25186200

C 4.42538000 -2.21689900 3.13030200

O 5.27116700 -1.81661400 2.02262900

C 5.50131400 -2.92542700 1.13057300

C 4.93099900 -4.14882800 1.84233900

C 3.81678300 -3.54218300 2.69926800

H 5.06608000 -2.31716000 4.01353400

H 3.67045400 -1.44590200 3.29810800

H 4.97758500 -2.72650700 0.18842800

H 6.57562300 -2.98956200 0.93845700

H 4.56936200 -4.89941400 1.13555400

H 5.69730500 -4.61018000 2.47389300

H 2.91167200 -3.35100000 2.11450400

H 3.54524700 -4.16346000 3.55516800

H 5.32824900 -0.48849100 1.29009100

**M4-b1**

P -1.72366400 1.08641000 0.17180600

C -1.35896600 2.60447800 -0.80258100

C -1.39577000 3.85774400 -0.17921700

C -0.84508700 2.51431400 -2.10141700

C -0.94793600 4.99669300 -0.84455500

H -1.77609300 3.94606900 0.83477900

C -0.39500400 3.65221400 -2.76379700

H -0.78418200 1.54822900 -2.59180700

C -0.44442700 4.89736200 -2.13915300

H -0.99100600 5.96098300 -0.34688200

H 0.00300900 3.56286600 -3.77024600

H -0.09457100 5.78441800 -2.65924400

C -3.07631000 1.68675200 1.26686100

C -3.04774000 1.33934400 2.61878800

C -4.13458300 2.46381400 0.77693500

C -4.07896000 1.75354300 3.46703300

H -2.21725100 0.75310700 3.00719200

C -5.15402000 2.88032300 1.62436500

H -4.15481000 2.74456500 -0.27383300

C -5.12899200 2.51996400 2.97388800

H -4.05044600 1.47708500 4.51668500

H -5.96985500 3.48436600 1.23706700

H -5.92740700 2.84289600 3.63550600

C -1.88915600 -2.36363000 -0.17011700

H -2.19585600 -2.07031300 0.84481100

C -2.41016400 -3.78435100 -0.43009800

H -2.08139300 -4.17833300 -1.39387500

H -2.08635700 -4.48662300 0.33581100

H -3.50719100 -3.77359600 -0.43024900

P -0.01619000 -2.07988200 -0.06829800

C 0.65583400 -2.29463500 -1.84029700

C 0.16920600 -3.51142200 -2.63596800

H -0.88754800 -3.40065000 -2.89651700

H 0.72719000 -3.56068800 -3.58105000

H 0.30460700 -4.46316200 -2.11907300

C 0.29619900 -1.03534000 -2.65409300

H -0.77735400 -0.95900800 -2.84327600

H 0.65240300 -0.12498700 -2.16175100

H 0.79358000 -1.11533800 -3.63159200

C 2.18779000 -2.30479200 -1.73767600

H 2.57456700 -3.19735200 -1.23567000

H 2.61431700 -2.27939500 -2.74990900

H 2.52536400 -1.40375400 -1.20999300

C 0.61855000 -3.50915000 1.02913900

C 1.95865700 -3.02893500 1.61582000

H 1.77880800 -2.12465900 2.20828500

H 2.36812500 -3.81101900 2.27056700

H 2.70827600 -2.80770200 0.84649900

C -0.32486200 -3.65153500 2.23873200

H -0.50234600 -2.68562100 2.72028900

H -1.28414100 -4.10679500 1.98663900

H 0.15855400 -4.30701800 2.97535800

C 0.81419900 -4.88115400 0.37408000

H -0.10621500 -5.26801600 -0.07148900

H 1.58720400 -4.86969000 -0.40057200

H 1.13414400 -5.59665000 1.14363100

Rh 0.58346000 0.30133100 0.98434900

C -6.36085000 0.14212300 -0.32536100

Fe -4.58615500 -0.80436500 -1.04812400

C -5.68829200 -0.47297000 0.76314500

C -6.70479300 -0.87089400 -1.26068100

C -6.23824600 -2.11210800 -0.75279300

C -5.60901500 -1.86412600 0.49794800

C -3.32746600 0.50417600 -2.19387700

C -3.69791100 -0.61120900 -2.97888600

C -3.20758900 -1.77630600 -2.33282100

C -2.52535800 -1.39162300 -1.14226100

C -2.59269300 0.04218700 -1.05954300

H -5.26942300 0.03584800 1.62116400

H -7.18781500 -0.71825600 -2.21644800

H -6.30532900 -3.06920300 -1.25317000

H -5.12010200 -2.60039000 1.12265800

H -3.55643500 1.54028000 -2.40714700

H -4.29628900 -0.58877600 -3.87957700

H -3.37894500 -2.79135500 -2.66461500

H -6.54104000 1.20233500 -0.43700400

C 5.58198800 1.80973600 -0.14486400

C 6.02257400 2.85633200 -0.95511200

C 5.10119600 3.73262300 -1.51854400

C 3.74288300 3.56922400 -1.25402700

C 3.29583700 2.53346900 -0.44088100

H 6.32510700 1.16335800 0.31245400

H 7.08586000 2.98889200 -1.13286400

H 5.44030500 4.54700000 -2.15158900

H 3.00936900 4.25197500 -1.67611200

C 4.22168400 1.63396600 0.10644200

C 3.65636100 0.50694700 0.94665100

C 2.44827300 0.88287700 1.77274700

C 1.83696300 2.29429000 -0.12345900

C 1.65881300 2.00132600 1.36672300

H 1.23838600 3.15971100 -0.44670500

O 1.42908600 1.09522800 -0.72675500

H 2.49802400 0.65512700 2.83245200

H 1.25092200 2.72346400 2.06749600

Cl -0.02171500 -0.32334000 3.34516800

O 4.80533800 0.05774800 1.85233000

H 3.47202000 -0.39083500 0.34430400

H 5.43808100 -0.48424100 1.22570500

C 4.47108400 -0.81515800 2.96645000

H 4.17947900 -0.19157100 3.80698300

H 3.66156900 -1.48656200 2.68122600

H 5.38341100 -1.36670900 3.19445000

C 5.59705000 -1.60947600 -0.96542800

O 6.19153600 -1.36804700 0.34044800

C 7.61872500 -1.17592700 0.21373100

C 7.85572100 -0.96774200 -1.27560600

C 6.78031200 -1.86997700 -1.88706800

H 4.90616000 -2.44861900 -0.86359900

H 5.04811900 -0.70981700 -1.26496900

H 7.90670700 -0.32513700 0.83725300

H 8.11278100 -2.07901300 0.58631200

H 7.66760300 0.07684400 -1.54617300

H 8.87116100 -1.23408200 -1.57488900

H 6.54812800 -1.62161500 -2.92411900

H 7.08540500 -2.92001600 -1.83852400

**M4-b2**

P -1.25525000 -1.16622500 -0.05074500

C -0.57717900 -2.43358400 1.09816600

C -0.15043200 -3.66812600 0.59573800

C -0.28923800 -2.11198200 2.42965200

C 0.52806800 -4.57213700 1.41108000

H -0.35135400 -3.92800200 -0.44011800

C 0.39022200 -3.01396000 3.24215500

H -0.58538800 -1.14611200 2.82528800

C 0.80131900 -4.24673800 2.73679700

H 0.84290300 -5.52979200 1.00688700

H 0.60497700 -2.74944300 4.27338100

H 1.32937900 -4.94966600 3.37466300

C -2.21375400 -2.22512200 -1.21243300

C -2.10645600 -1.99473900 -2.58467500

C -3.05282000 -3.24772800 -0.74834400

C -2.84417400 -2.77167000 -3.48260600

H -1.44344300 -1.21275400 -2.94771400

C -3.77854600 -4.02309400 -1.64410600

H -3.13703600 -3.43364600 0.31996800

C -3.67799900 -3.78184200 -3.01643200

H -2.75729900 -2.58271500 -4.54830200

H -4.42694900 -4.81351300 -1.27652500

H -4.24981100 -4.38526500 -3.71529000

C -2.49495600 2.09560300 -0.09263200

H -2.55997400 1.64902100 -1.09576800

C -3.45688400 3.29173100 -0.04539400

H -3.38445900 3.84764900 0.89113900

H -3.27348500 3.99441000 -0.85657100

H -4.48939600 2.93461000 -0.14346800

P -0.62985200 2.41198600 0.03567800

C -0.29088500 2.96788700 1.82563600

C -1.22408700 4.03666700 2.40868200

H -2.21950200 3.61874700 2.58784700

H -0.82997200 4.35291700 3.38392400

H -1.32547800 4.92648300 1.78546900

C -0.37221700 1.73860300 2.75372600

H -1.38712500 1.34082200 2.83238300

H 0.31095600 0.94871100 2.43170800

H -0.07207500 2.06364000 3.76001600

C 1.16879900 3.44601400 1.88227800

H 1.33193800 4.38794900 1.35108200

H 1.44674400 3.60809400 2.93140000

H 1.83706900 2.67538200 1.47741300

C -0.30464600 3.87181600 -1.15465500

C 1.19028500 3.81961300 -1.52762700

H 1.42918200 2.85541000 -1.99026100

H 1.40173200 4.61923500 -2.25051200

H 1.84120000 3.97440000 -0.66124600

C -1.07431100 3.62097700 -2.46448900

H -0.87934600 2.62066800 -2.85925700

H -2.15240600 3.76011300 -2.36229900

H -0.72570200 4.34798700 -3.20976800

C -0.63303800 5.28074000 -0.64502100

H -1.67954600 5.38770200 -0.34642300

H -0.00633600 5.57583600 0.20160800

H -0.44614600 5.99823000 -1.45513400

Rh 0.70041000 0.27872100 -0.64761200

C -5.97875600 -1.65468200 -0.14898800

Fe -4.67285300 -0.16681400 0.65446100

C -5.37094300 -0.94586900 -1.21844100

C -6.72568600 -0.72686900 0.62631300

C -6.57447900 0.55680900 0.03865200

C -5.73478000 0.41987200 -1.10062600

C -3.24491000 -0.93986100 2.06010200

C -4.03629900 0.05275400 2.68024200

C -3.83934400 1.26713700 1.97179900

C -2.92105600 1.03922400 0.90633900

C -2.53911800 -0.34719600 0.96835200

H -4.70798500 -1.36404000 -1.96350400

H -7.27012100 -0.94874400 1.53425500

H -6.98058600 1.48318300 0.42303300

H -5.39842100 1.22112500 -1.74570100

H -3.17495800 -1.97889300 2.35523900

H -4.71420000 -0.09665800 3.50966100

H -4.35314400 2.19702500 2.17406900

H -5.85828800 -2.70913800 0.05697700

C 5.26248800 1.33513500 1.51779700

C 5.84018800 0.76557000 2.65013800

C 5.37176000 -0.46082400 3.11983100

C 4.30728300 -1.09244600 2.47855100

C 3.71545500 -0.52474500 1.35130100

H 5.63800600 2.27963400 1.13186500

H 6.65205300 1.27417400 3.16022800

H 5.82168100 -0.91240600 3.99920200

H 3.90319800 -2.02486000 2.86647100

C 4.21717300 0.68713300 0.85714100

C 3.68176200 1.24771900 -0.43270500

C 2.74880600 0.39776400 -1.20764300

C 2.42847500 -1.05758300 0.75627600

C 2.36861800 -0.91258900 -0.76523700

H 2.26200400 -2.10189500 1.07647400

O 1.42184600 -0.19608800 1.20894400

H 2.71474400 0.61249600 -2.27344700

H 2.30690100 -1.77255700 -1.42799600

Cl 0.27833600 0.47515100 -3.13306600

O 5.02228800 1.42336300 -1.27028400

H 3.39695800 2.30233000 -0.36837500

H 5.48884000 0.47723900 -1.34411100

C 4.87880500 2.02625800 -2.56968300

H 4.49022300 1.30814900 -3.29345500

H 4.18364700 2.86119900 -2.46749500

H 5.86474900 2.38359000 -2.86650600

C 6.86152500 -1.27606300 -0.34061200

O 6.14655300 -0.78240500 -1.50356200

C 5.37316800 -1.84673600 -2.10487800

C 5.21888600 -2.87925200 -1.00002300

C 6.56777200 -2.77410800 -0.28296800

H 7.91986200 -1.04271000 -0.47775800

H 6.47939800 -0.75328300 0.53961600

H 4.42943800 -1.41944100 -2.44995900

H 5.94176400 -2.23690400 -2.95634600

H 4.40569900 -2.58610600 -0.32874000

H 5.01279400 -3.87783800 -1.39101600

H 6.52972700 -3.13227600 0.74794100

H 7.33403900 -3.34059000 -0.82138000

**M4-b3**

P -1.77325300 1.07906200 0.16374700

C -1.58594300 2.61107500 -0.83946200

C -1.70073700 3.85808600 -0.21178100

C -1.12960900 2.56367800 -2.16078700

C -1.38486400 5.03026800 -0.89430200

H -2.03914200 3.91434100 0.81931800

C -0.80799400 3.73524500 -2.84004500

H -1.01919600 1.60640700 -2.65911300

C -0.93346800 4.97206800 -2.21048300

H -1.48856700 5.98839500 -0.39369300

H -0.45323900 3.67926500 -3.86490500

H -0.68404200 5.88450300 -2.74408100

C -3.14646500 1.57594700 1.28586000

C -3.03343200 1.30705700 2.65087500

C -4.30037300 2.20770300 0.80201800

C -4.07518200 1.64884900 3.51842600

H -2.13205500 0.83139300 3.03136100

C -5.33168700 2.55055500 1.66755500

H -4.38863400 2.43343000 -0.25823100

C -5.22198000 2.26512000 3.03064100

H -3.98002600 1.43172600 4.57807000

H -6.22283700 3.03914700 1.28339700

H -6.03015200 2.52991900 3.70614000

C -1.55364600 -2.39551400 -0.15048000

H -1.84024600 -2.13921800 0.88022500

C -1.91077600 -3.86892300 -0.39002900

H -1.59501200 -4.21835700 -1.37493700

H -1.45552900 -4.52538800 0.34922200

H -2.99838100 -3.99674400 -0.32150000

P 0.27061700 -1.88362600 -0.14928000

C 0.86216000 -2.00823400 -1.95924900

C 0.49617100 -3.27974100 -2.73571600

H -0.58018300 -3.31189900 -2.92902700

H 0.99455900 -3.24956300 -3.71438100

H 0.78894900 -4.20721100 -2.24035700

C 0.28838000 -0.81188500 -2.74511100

H -0.79017600 -0.89608000 -2.89526700

H 0.52640500 0.13625400 -2.25371700

H 0.75631400 -0.80912100 -3.74015500

C 2.38468400 -1.79904800 -1.95404300

H 2.93032700 -2.60605900 -1.45275000

H 2.74188600 -1.75221400 -2.99162400

H 2.61215200 -0.83535200 -1.48004100

C 1.16747900 -3.21258000 0.89501200

C 2.46435300 -2.55869900 1.41360200

H 2.23020400 -1.69269200 2.04216100

H 3.01541500 -3.29670500 2.01569100

H 3.12286100 -2.22904300 0.60085900

C 0.32996700 -3.51853600 2.15076900

H 0.01723300 -2.60203600 2.65801800

H -0.54925100 -4.12946500 1.93928700

H 0.95664400 -4.09029500 2.84822200

C 1.51742900 -4.53315100 0.19932200

H 0.63443000 -5.04239300 -0.19765700

H 2.22786800 -4.40045100 -0.62349400

H 1.98383300 -5.20609800 0.93204700

Rh 0.57591700 0.48350500 0.87214800

C -6.29648400 -0.42480600 -0.06715500

Fe -4.45004400 -1.14879700 -0.86322300

C -5.48286100 -0.88785000 1.00000600

C -6.54489800 -1.51897600 -0.93932000

C -5.87939500 -2.65745800 -0.41312700

C -5.22043500 -2.26503300 0.78386000

C -3.42034000 0.27874600 -2.09910200

C -3.71424600 -0.88091900 -2.85085000

C -3.05906300 -1.97554000 -2.22838100

C -2.35030100 -1.50354400 -1.08490800

C -2.57128200 -0.08245500 -1.00930100

H -5.09448100 -0.28622600 1.81108800

H -7.09990200 -1.48098800 -1.86694100

H -5.83652100 -3.63686500 -0.87143000

H -4.59626200 -2.89440400 1.40469300

H -3.77421900 1.28039700 -2.30617500

H -4.36404300 -0.93521300 -3.71391900

H -3.13916800 -3.00737600 -2.54164600

H -6.63324000 0.59316500 -0.20652800

C 5.43834500 2.02590500 -0.55099000

C 5.82770100 3.07345900 -1.38487100

C 4.88079700 3.99458000 -1.82286200

C 3.54998500 3.86379100 -1.43029700

C 3.15484400 2.82691100 -0.59114300

H 6.19171300 1.31687100 -0.20902600

H 6.86569500 3.16652500 -1.68891700

H 5.17887000 4.81085700 -2.47392500

H 2.79813400 4.56559700 -1.78298600

C 4.10984000 1.90369300 -0.14516600

C 3.62788900 0.78253900 0.74877600

C 2.43711500 1.10009000 1.61413100

C 1.71636700 2.56430800 -0.19833100

C 1.60624600 2.21660600 1.28865600

H 1.08396400 3.42594400 -0.46834400

O 1.31197800 1.37910500 -0.83352700

H 2.53104200 0.77308500 2.64797200

H 1.19825800 2.89932300 2.02753600

Cl 0.04281600 -0.21786400 3.22895300

O 4.75660200 0.36704600 1.68159800

H 3.50674600 -0.15171200 0.19441400

C 5.32681000 1.36898700 2.55227200

H 5.99021000 2.02830400 1.99283800

H 4.48944000 1.92962400 2.96629800

H 5.85580700 0.83303700 3.33908000

C 6.12826700 -2.53035800 1.04891400

O 6.17081200 -1.22583800 0.41601700

C 5.96284100 -1.34663000 -1.01506000

C 6.25497000 -2.80796800 -1.31948600

C 5.75131900 -3.51278100 -0.05606600

H 7.12348700 -2.72440500 1.45881000

H 5.39729300 -2.48990600 1.86193500

H 4.92622900 -1.07900700 -1.24845100

H 6.63617600 -0.64156200 -1.50485400

H 5.74469200 -3.14228100 -2.22526500

H 7.33104200 -2.96206000 -1.44620700

H 4.66566200 -3.64345300 -0.09529800

H 6.20810400 -4.49234200 0.09749200

H 5.43328400 -0.24053800 1.15712800

**M4-b4**

P -1.46950500 0.98021200 0.58823300

C -1.34328200 2.79249700 0.29395800

C -1.20444500 3.65744800 1.38551300

C -1.18217200 3.30582900 -0.99777400

C -0.93537500 5.01077100 1.19181700

H -1.30874500 3.27312000 2.39655600

C -0.91034800 4.65702200 -1.19004600

H -1.25283500 2.64205400 -1.85327300

C -0.78680300 5.51422600 -0.09747100

H -0.84073700 5.66854000 2.05072700

H -0.78795400 5.04037200 -2.19871100

H -0.57690300 6.56852900 -0.25207600

C -2.52427200 0.95879200 2.09775100

C -2.18487500 0.12198900 3.16187500

C -3.66680500 1.76541800 2.19365600

C -2.99135700 0.08419900 4.30394900

H -1.29013000 -0.49319900 3.10043900

C -4.46143100 1.73147400 3.33268800

H -3.93277800 2.42322300 1.36967900

C -4.12498500 0.88447300 4.39129700

H -2.72180100 -0.57117500 5.12659000

H -5.34416600 2.36137300 3.39701000

H -4.74701800 0.85555000 5.28097900

C -1.70076000 -2.04596000 -1.12163300

H -1.73614300 -2.21271900 -0.03478300

C -2.28120200 -3.29165900 -1.80615300

H -2.20229900 -3.24376100 -2.89394000

H -1.78332000 -4.20477200 -1.48323800

H -3.34401500 -3.38420400 -1.55029500

P 0.13202900 -1.62597900 -1.36349900

C 0.33010500 -1.03887100 -3.16457900

C -0.35963900 -1.88086700 -4.24573000

H -1.44676400 -1.77667400 -4.17776100

H -0.06238500 -1.49678000 -5.23088600

H -0.10713100 -2.94230500 -4.21337600

C -0.22229600 0.39604900 -3.28040500

H -1.30831700 0.43303900 -3.16427800

H 0.25014400 1.06646500 -2.55756900

H 0.00932100 0.75988500 -4.29138200

C 1.83708700 -0.94133100 -3.44998800

H 2.32480000 -1.91890200 -3.50595400

H 1.97971000 -0.44826600 -4.41968600

H 2.33349000 -0.32566900 -2.68935600

C 1.04006600 -3.29286900 -1.14360800

C 2.49793800 -2.95265200 -0.78451800

H 2.52624900 -2.31748700 0.10944900

H 3.03581100 -3.88963100 -0.57668900

H 3.02378800 -2.43910100 -1.59525600

C 0.46007800 -4.00457600 0.09199500

H 0.41508100 -3.33701000 0.95682900

H -0.53216600 -4.42562100 -0.08046600

H 1.12216700 -4.84392300 0.34790200

C 1.03765900 -4.26607400 -2.32874100

H 0.02704400 -4.53548500 -2.64709000

H 1.57363100 -3.86993500 -3.19604500

H 1.54561800 -5.19177300 -2.02541200

Rh 0.88793500 0.14382500 0.41577000

C -6.10571500 -0.16116800 0.74763700

Fe -4.52646600 -0.55198000 -0.63627900

C -5.17275900 -1.04510500 1.35052900

C -6.62746900 -0.79571400 -0.41232900

C -6.01127300 -2.06963400 -0.52876300

C -5.11100600 -2.22148300 0.56096400

C -3.59369600 1.23201500 -1.38775800

C -4.14686400 0.49533600 -2.45905300

C -3.51197000 -0.77399200 -2.48399400

C -2.55610500 -0.83422300 -1.42903800

C -2.60093200 0.43116400 -0.74508400

H -4.57996000 -0.83979300 2.23122700

H -7.33021400 -0.36535100 -1.11301400

H -6.16281400 -2.77819100 -1.33237600

H -4.46376900 -3.07000000 0.74104300

H -3.86805900 2.23748800 -1.09608800

H -4.94852000 0.81810300 -3.10942600

H -3.75696700 -1.58142400 -3.16034700

H -6.34279200 0.83616500 1.09178800

C 5.31264600 1.95214700 -1.64900400

C 5.51949400 3.16431900 -2.29973700

C 4.58337000 4.18905700 -2.16225000

C 3.44420600 3.99308100 -1.38776600

C 3.23214300 2.78643800 -0.72336100

H 6.04238000 1.15059700 -1.74204500

H 6.40360900 3.30857800 -2.91287900

H 4.73965100 5.13709200 -2.66847900

H 2.69446600 4.77543300 -1.29666500

C 4.17920300 1.76379800 -0.85587200

C 4.00149000 0.48614900 -0.08678100

C 2.92455800 0.44684200 0.93094300

C 1.92947500 2.48214800 -0.01593100

C 2.10957400 1.58973400 1.21259700

H 1.40359300 3.41950700 0.23110400

O 1.18732200 1.67705200 -0.89536300

H 3.12015500 -0.25905600 1.73443400

H 1.88440600 1.93150400 2.21949900

Cl 0.88941600 -1.43353700 2.42108200

O 5.37423700 0.29982900 0.70260200

H 4.07931100 -0.41087800 -0.70990900

C 5.70972900 1.36918700 1.61455000

H 6.05546900 2.21120100 1.01778500

H 4.82862700 1.64404500 2.19877500

H 6.50724200 0.99097700 2.25450300

C 4.41505800 -2.24908500 3.08030400

O 5.26067400 -1.85569700 1.96588800

C 5.49734400 -2.97390800 1.08295200

C 4.91288100 -4.18851500 1.79866300

C 3.80132400 -3.57202000 2.65273800

H 5.06149400 -2.34878300 3.95891200

H 3.66350300 -1.47505400 3.24696400

H 4.98644100 -2.77607400 0.13439200

H 6.57376300 -3.04328600 0.90668500

H 4.54612900 -4.93743000 1.09282600

H 5.67364800 -4.65547700 2.43258300

H 2.89635100 -3.37768300 2.06904000

H 3.52767400 -4.18875700 3.51115300

H 5.27364300 -0.58202800 1.25502200

**M4-b5**

P -1.43454500 1.03300400 0.50485100

C -1.25275300 2.82088700 0.10701700

C -1.08524200 3.74798800 1.14359700

C -1.06925000 3.24738300 -1.21256300

C -0.76032200 5.07366200 0.86628000

H -1.20654300 3.43300400 2.17640500

C -0.73854500 4.57074800 -1.48805800

H -1.17079700 2.53873500 -2.02745600

C -0.58307800 5.48871100 -0.45105500

H -0.64079600 5.78005900 1.68232200

H -0.59476700 4.88281000 -2.51813600

H -0.32691600 6.52132900 -0.66931800

C -2.45755600 1.12280900 2.03392300

C -2.11520900 0.32187200 3.12459200

C -3.57300400 1.96699200 2.12293500

C -2.89252800 0.35495700 4.28641100

H -1.23952400 -0.32067900 3.06739300

C -4.33957500 2.00285700 3.28124900

H -3.83912900 2.59886100 1.27876400

C -4.00105400 1.19055600 4.36628400

H -2.62069100 -0.27287900 5.12950500

H -5.20219400 2.66051100 3.34081400

H -4.60215500 1.21539600 5.27036800

C -1.77484500 -2.05958500 -1.03779600

H -1.77935800 -2.16655100 0.05686400

C -2.39846700 -3.32757500 -1.63871500

H -2.33510500 -3.34651600 -2.72883700

H -1.92202400 -4.23363400 -1.26677300

H -3.45926100 -3.37428200 -1.36320300

P 0.05807900 -1.68424100 -1.35788700

C 0.19082600 -1.16080200 -3.18511700

C -0.58023400 -2.00819200 -4.20411500

H -1.65879700 -1.87483900 -4.08072300

H -0.32729100 -1.65906100 -5.21439400

H -0.35057100 -3.07473100 -4.15404200

C -0.30391700 0.29390700 -3.31122100

H -1.38221000 0.38050900 -3.15675100

H 0.22561800 0.95493600 -2.61735500

H -0.09411800 0.63133400 -4.33610300

C 1.68096100 -1.12827600 -3.55509500

H 2.13374100 -2.12279300 -3.59939500

H 1.78610100 -0.67614300 -4.54921200

H 2.23248800 -0.49720400 -2.84800500

C 0.94744700 -3.35876800 -1.12310200

C 2.43464200 -3.04062200 -0.87846400

H 2.54389100 -2.33999600 -0.03987300

H 2.95962600 -3.97718700 -0.63896100

H 2.91049600 -2.60575600 -1.76267500

C 0.42635300 -3.99182700 0.17913500

H 0.44056000 -3.27928200 1.00921800

H -0.58413500 -4.39320400 0.08132600

H 1.08090100 -4.83427200 0.44316300

C 0.84979400 -4.38498700 -2.25855400

H -0.18431900 -4.63516400 -2.50789900

H 1.34544800 -4.04408500 -3.17229100

H 1.34709600 -5.31234500 -1.94277600

Rh 0.93392800 0.10592000 0.35033000

C -6.09173800 0.03509700 0.83326900

Fe -4.55979700 -0.46990100 -0.56922200

C -5.17157800 -0.84914000 1.45491300

C -6.66148100 -0.63558300 -0.28301000

C -6.08747100 -1.93204500 -0.35427800

C -5.16485700 -2.06171100 0.71973400

C -3.59866900 1.24391300 -1.43805200

C -4.19653700 0.46414300 -2.45401400

C -3.59522300 -0.82162300 -2.42270700

C -2.61591200 -0.84836800 -1.38763400

C -2.61399300 0.45275500 -0.77280800

H -4.55223600 -0.62330800 2.31188000

H -7.36814000 -0.21570000 -0.98613200

H -6.27685300 -2.66984400 -1.12291200

H -4.54075000 -2.92153500 0.92545100

H -3.83976700 2.27082800 -1.19579200

H -5.00522800 0.77191200 -3.10301300

H -3.88159900 -1.66038100 -3.04263600

H -6.29277800 1.05306900 1.13738900

C 5.66528000 2.15128400 -1.13426200

C 5.98272500 3.46795400 -1.46797500

C 5.00770400 4.45735100 -1.40020800

C 3.71985400 4.13201600 -0.97862300

C 3.39592300 2.82431100 -0.63496500

H 6.44972000 1.40019700 -1.16057600

H 6.99415400 3.71614500 -1.77454300

H 5.25336800 5.48207500 -1.66241200

H 2.95066800 4.89694600 -0.90179000

C 4.37324800 1.82320000 -0.73358400

C 3.93191900 0.42018700 -0.37595700

C 2.95882200 0.36058100 0.77969700

C 2.03246600 2.41230300 -0.12529400

C 2.19113800 1.52090800 1.10949600

H 1.42558300 3.30613900 0.08879700

O 1.40936600 1.54864500 -1.04257300

H 3.23523000 -0.32827800 1.56843400

H 2.02857900 1.87235700 2.12409000

Cl 0.92307800 -1.35176600 2.45607100

O 5.15928000 -0.36395400 0.04364900

H 3.57458400 -0.12450400 -1.25617300

C 5.72616500 -1.21837700 -0.96752900

H 5.98981200 -0.59660800 -1.82192600

H 6.61847900 -1.67024100 -0.53249000

H 4.99996300 -1.97978800 -1.26359100

C 4.62498600 -1.51179900 3.38319200

O 5.12974400 -1.81151300 2.04760000

C 4.89269800 -3.20240400 1.74057000

C 3.68914400 -3.57922100 2.58280600

C 3.94803700 -2.79445400 3.87404900

H 5.47809300 -1.20484000 3.99189200

H 3.91246100 -0.68957100 3.29729000

H 4.72691200 -3.28204600 0.66363000

H 5.79055700 -3.76950900 2.01359400

H 2.76677200 -3.21559300 2.11824200

H 3.61696600 -4.65707000 2.74503500

H 3.01575600 -2.56678800 4.39429100

H 4.61151000 -3.35070800 4.54335100

H 5.04585100 -0.90986000 0.96012100

**M5**

P 1.14617000 1.09701500 0.13836800

C 0.42765100 2.22426900 1.41876500

C -0.16775400 3.40607300 0.95470900

C 0.36863700 1.94792500 2.78753100

C -0.81737500 4.27366800 1.82467100

H -0.09823700 3.65740100 -0.10068600

C -0.26720600 2.82755400 3.66546700

H 0.83076600 1.05012000 3.18247600

C -0.86922100 3.98717100 3.18922700

H -1.26697000 5.18437200 1.43968700

H -0.28858400 2.59830700 4.72711000

H -1.36373400 4.66975400 3.87378800

C 2.18075400 2.32557400 -0.76853300

C 1.97670300 2.49892800 -2.13874200

C 3.14235600 3.10873500 -0.11821700

C 2.75017700 3.41536500 -2.85445500

H 1.23880800 1.88946600 -2.64958400

C 3.91386400 4.01790600 -0.83184200

H 3.29044200 3.01124000 0.95267200

C 3.72370300 4.16677600 -2.20653900

H 2.59138700 3.53021100 -3.92223100

H 4.66153400 4.61424800 -0.31683800

H 4.33014600 4.87290400 -2.76567600

C 1.87931300 -2.36052800 0.04808100

H 2.05195300 -1.95275300 -0.95711000

C 2.63384100 -3.69354100 0.14252700

H 2.46889200 -4.21033300 1.09209800

H 2.35179800 -4.37424900 -0.65884200

H 3.70974800 -3.50307900 0.04369500

P -0.01588500 -2.37793600 0.06430800

C -0.54470700 -2.64971000 1.87205200

C 0.25747100 -3.69581200 2.65526100

H 1.30043900 -3.38292700 2.76374400

H -0.16369600 -3.78921100 3.66555800

H 0.23698000 -4.68455600 2.19270600

C -0.38370700 -1.30661600 2.60457700

H 0.66823400 -1.09034400 2.80579600

H -0.80769000 -0.46959000 2.04019400

H -0.90825200 -1.35460600 3.56877600

C -2.03706400 -3.01133500 1.89676300

H -2.22023600 -4.03888900 1.57310200

H -2.40748500 -2.91727200 2.92577300

H -2.63073700 -2.33769100 1.26741100

C -0.53330500 -3.91254500 -0.95027500

C -2.01980800 -3.75506300 -1.31634000

H -2.19801000 -2.77798800 -1.77089100

H -2.28386100 -4.52852800 -2.04976400

H -2.69562900 -3.86559000 -0.46621100

C 0.21467400 -3.87953900 -2.29618700

H -0.05417900 -2.99265700 -2.87332100

H 1.30264300 -3.88039500 -2.20740100

H -0.07492000 -4.76995000 -2.86891300

C -0.34115700 -5.28057100 -0.28071600

H 0.68882200 -5.47145700 0.02814700

H -0.98663600 -5.40382400 0.59325600

H -0.62143500 -6.06154800 -0.99964400

Rh -0.63316500 -0.08828200 -1.17378600

C 5.81516700 0.88648700 -0.15392900

Fe 4.33517400 -0.40495100 0.67080400

C 4.96658500 0.42412500 -1.19390100

C 6.44156300 -0.24376500 0.43943200

C 5.97610000 -1.40455500 -0.23377800

C 5.06202600 -0.99116400 -1.24158700

C 3.14033100 0.54269300 2.16961000

C 3.80273000 -0.55850500 2.74947100

C 3.38420700 -1.72423300 2.05851100

C 2.45771000 -1.36358100 1.03865900

C 2.30320300 0.07150400 1.10752100

H 4.31871000 1.03369000 -1.80958300

H 7.11433100 -0.22782800 1.28620000

H 6.23380300 -2.42616800 0.01278100

H 4.49439300 -1.63469400 -1.90159700

H 3.24393300 1.57334500 2.47925300

H 4.53462500 -0.51705300 3.54479200

H 3.76122200 -2.72075100 2.24085200

H 5.92665600 1.91608200 0.15749300

C -4.38645800 -1.48511400 -2.53588200

C -5.66308000 -1.67333400 -2.01226100

C -6.15686500 -0.82506000 -1.02254800

C -5.35150900 0.21203200 -0.55421400

C -4.07308100 0.39306300 -1.06594600

H -4.02077100 -2.14054500 -3.32239500

H -6.28227800 -2.48239500 -2.38831700

H -7.15690000 -0.96645000 -0.62441200

H -5.71696500 0.89031000 0.21658900

C -3.56502400 -0.45169300 -2.07174700

C -2.21888800 -0.18995200 -2.61552200

C -1.65109600 1.12316100 -2.49751100

H -1.91277500 -0.80757300 -3.45683600

C -3.13131500 1.43536600 -0.54843800

C -2.42987700 2.15251000 -1.71491000

H -3.63294600 2.14192600 0.12514500

O -2.07222300 0.79400900 0.19227100

H -1.04134000 1.51010700 -3.31027300

H -1.75081400 2.91158100 -1.29535900

Cl 0.98146800 -0.64304900 -2.90267100

C -4.07428500 3.82888500 -1.98706700

H -3.40467200 4.50972800 -1.43860600

H -4.56031700 4.38112600 -2.79337700

H -4.84912200 3.46538100 -1.29665100

O -3.35787400 2.77431600 -2.57804300

H -2.50441800 0.20956500 0.85667300

C -3.32194300 1.12474800 3.05990500

O -3.43137400 -0.07568700 2.30164600

C -4.65672800 -0.71905100 2.64717000

C -5.59585900 0.38918800 3.17525500

C -4.74619700 1.67166500 3.08710900

H -2.95654200 0.89496800 4.07185400

H -2.59176000 1.76735400 2.55944000

H -5.02906200 -1.21337200 1.74671400

H -4.45973000 -1.47693900 3.41529500

H -6.51176100 0.46225700 2.58355500

H -5.88603200 0.18471100 4.20906400

H -4.95093700 2.20845200 2.15504900

H -4.92194100 2.35794000 3.91836300

**Product**

C 2.37345400 1.14112400 -0.33083300

C 3.27156600 0.10560900 -0.57705400

C 2.85255200 -1.21740500 -0.47768800

C 1.53292300 -1.49734800 -0.12232800

C 0.63328300 -0.46830700 0.13246600

H 2.69629800 2.17536500 -0.42094100

H 4.29706300 0.33325200 -0.85154400

H 3.54696600 -2.02779000 -0.67619700

H 1.19637900 -2.52961700 -0.04595300

C 1.05148200 0.86793300 0.02260700

C 0.07428400 1.94270500 0.23417100

C -1.23279800 1.69774100 0.34360400

H 0.45178700 2.96253100 0.25733100

C -0.75785600 -0.76353400 0.64256400

C -1.78815100 0.30090100 0.25035600

H -1.08824200 -1.73479700 0.24263200

O -0.76901200 -0.78827400 2.06529700

H -1.94705100 2.51006200 0.43841700

H -2.62773800 0.19157100 0.95472900

C -3.29903300 -0.80563600 -1.19053200

H -4.18093400 -0.47230200 -0.62496900

H -3.55621800 -0.86954900 -2.24945400

H -3.01868200 -1.80689800 -0.83489600

O -2.24319100 0.11848200 -1.07924000

H 0.05775100 -1.19619800 2.35839500

**M3-syn**

P -0.56027800 -1.14028500 0.02205600

C 0.02302700 -2.39783100 1.23616500

C 0.27575900 -3.71834600 0.85402500

C 0.33723600 -1.99495400 2.53992200

C 0.83227800 -4.62155900 1.75909800

H 0.04529800 -4.05354200 -0.15245100

C 0.88852700 -2.89760600 3.44063700

H 0.16130200 -0.96999500 2.84623200

C 1.14251300 -4.21253800 3.05105100

H 1.02507700 -5.64298900 1.44659300

H 1.13033600 -2.57081700 4.44708900

H 1.57944900 -4.91431800 3.75475900

C -1.38890300 -2.20453600 -1.22272500

C -1.05764200 -2.14613600 -2.57543200

C -2.35054200 -3.12834200 -0.78686300

C -1.70598900 -2.98331900 -3.48723300

H -0.31473000 -1.43422800 -2.92055300

C -2.98229000 -3.96736800 -1.69456800

H -2.60460400 -3.19032100 0.26791900

C -2.66568700 -3.88939900 -3.05250000

H -1.45055000 -2.92027600 -4.54023700

H -3.72276100 -4.68132700 -1.34598900

H -3.16470300 -4.54029400 -3.76398400

C -1.92305300 2.17564400 0.03314800

H -2.00162000 1.79405500 -0.99578400

C -2.90696400 3.34591300 0.18130100

H -2.83141900 3.83091000 1.15646300

H -2.75073000 4.11028600 -0.57578700

H -3.93303400 2.97501000 0.06740400

P -0.07019600 2.51846300 0.13402800

C 0.33483300 2.98660300 1.93136500

C -0.62361300 3.95255100 2.63868600

H -1.59443800 3.47697500 2.81159600

H -0.20749000 4.19906500 3.62470700

H -0.78332200 4.88988000 2.10291300

C 0.36321800 1.68539000 2.75539500

H -0.62926500 1.23100900 2.83805900

H 1.06602500 0.95720800 2.33896100

H 0.69270700 1.93477600 3.77333600

C 1.76785300 3.54417800 1.95042600

H 1.84607300 4.53663400 1.49968500

H 2.09961300 3.62725300 2.99296300

H 2.45731100 2.87029300 1.42929800

C 0.16551100 4.04476900 -0.98855200

C 1.63539500 4.04697200 -1.44154000

H 1.82379700 3.17697400 -2.07594300

H 1.82068900 4.95336300 -2.03274200

H 2.34514800 4.02864200 -0.61105300

C -0.66218800 3.88882900 -2.27947700

H -0.49294500 2.91934500 -2.75156400

H -1.73436300 4.03090100 -2.13309000

H -0.33084500 4.66465400 -2.98155200

C -0.16839000 5.40222100 -0.35521400

H -1.20311400 5.46727400 -0.00849400

H 0.48781200 5.64907200 0.48282000

H -0.02792400 6.17934500 -1.11760500

Rh 1.18104300 0.19835200 -0.59929600

C -5.27073900 -1.63349600 -0.33472200

Fe -4.00779800 -0.18354800 0.59729400

C -4.61030200 -0.87590400 -1.33865400

C -6.05616100 -0.74202900 0.44521100

C -5.87806600 0.56673600 -0.07425900

C -4.98265100 0.48296100 -1.17554200

C -2.60143600 -0.99050300 2.01627800

C -3.43691900 -0.05758400 2.66218000

C -3.25549400 1.19700600 2.02588800

C -2.31104800 1.05517600 0.97015300

C -1.88638100 -0.32005800 0.97090600

H -3.90996000 -1.25895200 -2.06924900

H -6.64569500 -1.00628300 1.31254900

H -6.30374200 1.47481100 0.33197200

H -4.61828300 1.31334000 -1.76631400

H -2.50066800 -2.03657300 2.27211500

H -4.12549000 -0.26982800 3.46850500

H -3.80036700 2.09949700 2.26376200

H -5.16910500 -2.69751900 -0.17279800

C 6.51464600 -0.96066400 -0.56295200

C 7.12891800 -0.78377300 0.67122900

C 6.36498700 -0.81966500 1.83718100

C 4.99273200 -1.04054600 1.75441500

C 4.36813900 -1.22582700 0.52204400

H 7.10521800 -0.92807000 -1.47581600

H 8.20037300 -0.61642800 0.72449400

H 6.83723400 -0.67942000 2.80463300

H 4.38246700 -1.06742900 2.65401100

C 5.13350700 -1.17970500 -0.65624000

C 4.47876300 -1.31427000 -1.96064800

C 3.14824500 -1.44389500 -2.07860900

H 5.11061400 -1.26647800 -2.84502100

C 2.86456600 -1.44253600 0.49559900

C 2.25376800 -1.53802200 -0.91212400

H 2.64714200 -2.34687700 1.08603200

O 2.19482100 -0.32595200 1.07108200

H 2.68826700 -1.49971100 -3.06218900

H 1.57617600 -2.38769100 -1.02911800

Cl 0.61075400 0.72837700 -2.95192200

C 3.96839900 1.79167900 -1.76046800

H 3.48035700 1.52902400 -2.70225100

H 4.15713200 2.86949200 -1.72764900

H 4.91438000 1.25347800 -1.67373100

O 3.08157300 1.43572900 -0.70449900

H 3.53990800 1.03262600 0.05563800

**TS_3-4_syn**

P 0.54482700 -1.13347000 -0.00705400

C -0.16988000 -2.30641100 -1.23526900

C -0.55099700 -3.60068700 -0.86560500

C -0.48063200 -1.84843400 -2.52107500

C -1.23573100 -4.41793600 -1.76330900

H -0.32296400 -3.97811200 0.12636100

C -1.16091000 -2.66673300 -3.41565800

H -0.20241700 -0.84318500 -2.81907500

C -1.54594100 -3.95229400 -3.03685600

H -1.52954700 -5.41779700 -1.46007400

H -1.39932400 -2.29491600 -4.40717400

H -2.08543700 -4.58723100 -3.73278500

C 1.30917300 -2.29902100 1.18681600

C 1.00525200 -2.25942800 2.54712200

C 2.18873600 -3.28053400 0.70556700

C 1.60023800 -3.17376900 3.42050600

H 0.32196900 -1.50729400 2.92618900

C 2.76903800 -4.19363200 1.57543000

H 2.42062800 -3.32995700 -0.35477500

C 2.48043600 -4.13588700 2.94052600

H 1.36542200 -3.12743300 4.47912200

H 3.44759500 -4.94986400 1.19193000

H 2.93800400 -4.84590700 3.62258800

C 2.09746800 2.08494200 -0.00328500

H 2.15377200 1.68450500 1.01982200

C 3.15135400 3.19474800 -0.13279000

H 3.11090100 3.69634000 -1.10151500

H 3.03641500 3.95690200 0.63430300

H 4.15219100 2.76031500 -0.01904000

P 0.26716600 2.53831300 -0.09300900

C -0.11780200 3.08016200 -1.87253800

C 0.91189700 3.98736300 -2.55622600

H 1.83519100 3.43701300 -2.76591200

H 0.50527600 4.31541600 -3.52203900

H 1.16020000 4.88044600 -1.98018400

C -0.24245600 1.80478300 -2.72518300

H 0.70754300 1.26559800 -2.80143600

H -1.01141300 1.13217100 -2.33334500

H -0.53609100 2.09711700 -3.74216700

C -1.50654200 3.74284300 -1.87170000

H -1.49972400 4.73777800 -1.41958100

H -1.83863800 3.85713000 -2.91149000

H -2.23914900 3.12572800 -1.33885700

C 0.10819200 4.03765900 1.07715500

C -1.36301900 4.11570300 1.52092100

H -1.63314200 3.22902700 2.10034500

H -1.48666200 5.00157200 2.15769500

H -2.05918500 4.19226300 0.68357200

C 0.92948400 3.80113500 2.35899500

H 0.70480600 2.83462200 2.81161500

H 2.00800200 3.87961200 2.20765900

H 0.65046500 4.58017900 3.07965800

C 0.52496100 5.39063000 0.48253600

H 1.56793200 5.40955700 0.15484000

H -0.10201000 5.69363700 -0.35879900

H 0.41290200 6.15448000 1.26272200

Rh -1.16456300 0.34468200 0.58346200

C 5.20482900 -1.94785500 0.27924100

Fe 4.03225600 -0.39893200 -0.61163100

C 4.59864000 -1.17359600 1.30429700

C 6.04278700 -1.09012100 -0.48378700

C 5.95131400 0.21447900 0.06766600

C 5.05664600 0.16247300 1.17127700

C 2.56788000 -1.09231400 -2.02972800

C 3.45499900 -0.20489300 -2.67092300

C 3.35893800 1.04978200 -2.01592700

C 2.41492200 0.95434700 -0.95500400

C 1.90830100 -0.39333000 -0.96699500

H 3.88209600 -1.53014800 2.03277600

H 6.61191900 -1.37026900 -1.35976700

H 6.43461000 1.10213900 -0.31838300

H 4.74957400 1.00117400 1.78261700

H 2.39912800 -2.12615100 -2.29927800

H 4.12030600 -0.44869500 -3.48776600

H 3.95360900 1.92145100 -2.24986700

H 5.03283500 -2.99889400 0.09312900

C -6.68165500 -0.93291800 0.68765700

C -7.34281300 -0.89388000 -0.53428900

C -6.61568500 -0.96514200 -1.72124000

C -5.22914300 -1.08089900 -1.67143200

C -4.56047600 -1.11932100 -0.45066800

H -7.24564500 -0.87317000 1.61532400

H -8.42470500 -0.80631100 -0.56182400

H -7.12649400 -0.93243800 -2.67855000

H -4.64715000 -1.13560800 -2.58869300

C -5.28718800 -1.04202200 0.74793700

C -4.58547800 -1.04750500 2.03398500

C -3.25059500 -1.13951700 2.11996200

H -5.19074600 -0.95100700 2.93255800

C -3.05171100 -1.22101900 -0.44567300

C -2.38586500 -1.29360800 0.93473400

H -2.75744000 -2.07263900 -1.06616100

O -2.47043900 -0.04604900 -1.07637600

H -2.75903700 -1.11738100 3.08899800

H -1.75238800 -2.17852500 1.04718100

Cl -0.52310000 0.70498600 2.88036800

C -3.85839300 2.00879000 1.36919900

H -3.57462900 1.70897000 2.38663300

H -4.00671000 3.09841400 1.36403900

H -4.81832200 1.53427500 1.12755500

O -2.86230600 1.66585500 0.44508700

H -2.96834100 0.83801500 -0.53728500

**M4-syn**

P 0.57700500 -1.13262000 0.05242800

C -0.14917300 -2.36124600 -1.11350200

C -0.54325200 -3.63144300 -0.67935000

C -0.46797800 -1.96221900 -2.41739400

C -1.24602700 -4.48260800 -1.53063400

H -0.30883600 -3.96270400 0.32784600

C -1.16593200 -2.81410200 -3.26624100

H -0.18284900 -0.97446100 -2.76552100

C -1.56191100 -4.07535200 -2.82333300

H -1.54999900 -5.46270000 -1.17698000

H -1.40934800 -2.48755800 -4.27240100

H -2.11517600 -4.73637600 -3.48319100

C 1.41911900 -2.22608500 1.26309400

C 1.27511400 -2.02214700 2.63533100

C 2.23314400 -3.27096000 0.80148000

C 1.95302100 -2.84738300 3.53668900

H 0.64240100 -1.21806300 2.99737600

C 2.89562300 -4.09523500 1.70107400

H 2.35064700 -3.43689000 -0.26638900

C 2.76041000 -3.87972200 3.07438700

H 1.84005200 -2.67701000 4.60269600

H 3.52188800 -4.90359500 1.33518200

H 3.28388800 -4.52053200 3.77735900

C 2.09398500 2.07170200 -0.05611700

H 2.17347900 1.67655900 0.96734900

C 3.14366700 3.18258600 -0.21058000

H 3.08011100 3.68391400 -1.17822800

H 3.04663000 3.94469500 0.55928400

H 4.14699300 2.74839100 -0.12111300

P 0.25968100 2.52347800 -0.11571900

C -0.14984200 3.04962400 -1.89350500

C 0.85982800 3.96298200 -2.59894800

H 1.78375600 3.41993900 -2.82424400

H 0.43281200 4.28469000 -3.55816700

H 1.11097100 4.85997100 -2.03033900

C -0.27298100 1.77020100 -2.74121900

H 0.68083200 1.24016100 -2.82964300

H -1.02595900 1.08614200 -2.34012800

H -0.58557100 2.05667700 -3.75413300

C -1.54230800 3.70399400 -1.86743300

H -1.52317100 4.70790000 -1.43482900

H -1.90587400 3.79757300 -2.89874600

H -2.25661100 3.09754100 -1.29731500

C 0.12779400 4.02777300 1.04667600

C -1.33579200 4.12818400 1.50981900

H -1.60587900 3.25509500 2.10786100

H -1.43990200 5.02570900 2.13379700

H -2.04486500 4.19841500 0.68298000

C 0.95892000 3.76519000 2.31719800

H 0.72534900 2.79310200 2.75575100

H 2.03690400 3.83391000 2.15690900

H 0.69422500 4.53362600 3.05433500

C 0.55247000 5.37639500 0.44896200

H 1.58557500 5.38375800 0.09220300

H -0.09495900 5.69039800 -0.37343800

H 0.47098900 6.13856000 1.23467300

Rh -1.23986000 0.36365300 0.59903600

C 5.24749300 -1.92925200 0.18335400

Fe 4.03963900 -0.39806900 -0.68781900

C 4.67735900 -1.14094300 1.21800200

C 6.05465700 -1.08093900 -0.62261000

C 5.97929900 0.23182800 -0.08829300

C 5.12597100 0.19407500 1.04831800

C 2.54145600 -1.12938000 -2.04290000

C 3.40028100 -0.24470700 -2.72715400

C 3.31182400 1.02139800 -2.09229200

C 2.39801500 0.93511400 -1.00420000

C 1.90327900 -0.41514100 -0.97806800

H 3.98807100 -1.48811900 1.97663500

H 6.59252400 -1.37244800 -1.51456400

H 6.44452000 1.11482400 -0.50609200

H 4.83866900 1.04114800 1.65771900

H 2.38103500 -2.17187000 -2.28269900

H 4.04525800 -0.49807800 -3.55731000

H 3.89407900 1.89319800 -2.35575300

H 5.07110100 -2.98335500 0.01960500

C -6.73960600 -0.91751400 0.57380200

C -7.36193100 -0.90408800 -0.66871200

C -6.60095300 -1.02297900 -1.83035000

C -5.21880500 -1.15557900 -1.73393200

C -4.58911200 -1.16277400 -0.49148300

H -7.33103400 -0.82507700 1.48136700

H -8.44101500 -0.80160500 -0.73225200

H -7.08165900 -1.01438400 -2.80355300

H -4.61056900 -1.24719100 -2.63123400

C -5.34972400 -1.04425100 0.68223300

C -4.68944900 -1.04072200 1.98916900

C -3.35767900 -1.12217400 2.11382500

H -5.32229100 -0.94020400 2.86777600

C -3.08539900 -1.28124900 -0.43582100

C -2.45254900 -1.27098900 0.95891000

H -2.76936800 -2.15717400 -1.00765900

O -2.48070200 -0.14676900 -1.15005600

H -2.89376700 -1.08774400 3.09551300

H -1.79158000 -2.12821900 1.11641700

Cl -0.70645700 0.73966800 2.90746700

C -3.76975600 1.99128100 1.50314500

H -3.40729800 1.69762800 2.49910000

H -3.90854900 3.08435300 1.50774100

H -4.75819400 1.53149900 1.35679200

O -2.87527900 1.63189200 0.49483300

H -3.00345700 0.65633800 -0.82587100

**TS_4-5_syn**

P -1.13574700 -1.11424100 0.45857000

C -0.50601700 -1.70960400 2.08425000

C 0.10628800 -2.96922500 2.11770300

C -0.54880000 -0.94885200 3.25107700

C 0.67449400 -3.45070800 3.29102400

H 0.12437400 -3.58344800 1.21839000

C 0.02725300 -1.42886200 4.42821400

H -1.03135700 0.02276300 3.24661200

C 0.64214600 -2.67497900 4.45101100

H 1.13922600 -4.43230700 3.30444200

H -0.00858400 -0.82214300 5.32814300

H 1.08796000 -3.04787800 5.36796300

C -2.41233400 -2.39368800 0.11519600

C -2.59874400 -2.84977700 -1.19274900

C -3.19684400 -2.92949600 1.14507800

C -3.59112800 -3.78972700 -1.47130300

H -1.97004800 -2.46239600 -1.99033800

C -4.18850400 -3.86314900 0.86291400

H -3.01594100 -2.63533000 2.17572500

C -4.39337800 -4.28856200 -0.44946900

H -3.73244800 -4.13056300 -2.49230700

H -4.79302200 -4.26863300 1.66862900

H -5.16667300 -5.01884000 -0.66838000

C -0.83223300 2.33021700 -0.46667700

H -0.71213500 1.61327200 -1.29294100

C -1.34022200 3.65108200 -1.06600200

H -1.35808700 4.46624300 -0.34086100

H -0.72250200 3.97262100 -1.90281700

H -2.35995000 3.51469200 -1.44215900

P 0.91239600 2.32534400 0.29168200

C 0.86969900 3.40489000 1.85592700

C 0.09563000 4.72622700 1.79986800

H -0.97845000 4.54471300 1.70269400

H 0.24726700 5.27307500 2.74048800

H 0.41699900 5.37473100 0.98068700

C 0.24513700 2.52727700 2.95544500

H -0.80373200 2.29558900 2.74676900

H 0.79111700 1.58383700 3.06636500

H 0.28615000 3.06481400 3.91237300

C 2.31881000 3.68127800 2.28454200

H 2.83123800 4.38198900 1.62065900

H 2.31436800 4.12100000 3.29011100

H 2.90709200 2.75694800 2.32858700

C 1.99278800 3.15812600 -1.03711800

C 3.43202700 2.66571000 -0.78239900

H 3.49035100 1.57685000 -0.90650000

H 4.10371000 3.11914900 -1.52325300

H 3.81014800 2.93069200 0.20939900

C 1.58886600 2.57601600 -2.40321600

H 1.47665400 1.48983600 -2.35390700

H 0.66238700 2.99576200 -2.80011500

H 2.38470600 2.79838900 -3.12610700

C 1.98424800 4.68915000 -1.11154000

H 0.98365600 5.09777400 -1.27566300

H 2.39577700 5.15403000 -0.21102900

H 2.61166100 5.00533900 -1.95551500

Rh 0.69362600 -0.82744000 -0.89585600

C -5.00077900 -0.39204800 -1.15457200

Fe -3.79005100 1.04673900 -0.16519400

C -3.99141400 0.06291700 -2.04614600

C -5.79590200 0.72746900 -0.78567800

C -5.27666400 1.87425900 -1.44440700

C -4.16236600 1.45982300 -2.22404900

C -3.21470300 0.36789700 1.76487200

C -3.70965000 1.68250100 1.88278500

C -2.88691200 2.52672000 1.09257200

C -1.86254300 1.75005700 0.48021300

C -2.07470700 0.38313300 0.89671900

H -3.18543200 -0.52052300 -2.47400900

H -6.61860500 0.71863700 -0.08315800

H -5.63953200 2.88770800 -1.33668100

H -3.52739400 2.09357600 -2.82842800

H -3.65746400 -0.51513800 2.20375100

H -4.59361700 1.98309800 2.42875300

H -3.04809200 3.58434200 0.93489400

H -5.10753100 -1.40258200 -0.77985100

C 6.57664500 -1.26304300 -0.71048600

C 7.03120600 -0.23772600 0.11264300

C 6.17521900 0.32288700 1.05615600

C 4.86722100 -0.14596400 1.17770000

C 4.40437700 -1.15839300 0.34629900

H 7.23964500 -1.70319100 -1.45086300

H 8.05059900 0.12204600 0.01845300

H 6.52418000 1.12077400 1.70398500

H 4.20349900 0.28026800 1.92632700

C 5.26247600 -1.72827400 -0.61105300

C 4.75893300 -2.76532400 -1.50882000

C 3.44491600 -3.04512500 -1.61399300

H 5.48350200 -3.28948900 -2.12756400

C 3.00742600 -1.70387600 0.49397100

C 2.48007800 -2.37375300 -0.75567000

H 2.99800100 -2.44923100 1.30506800

O 2.05514200 -0.70702800 0.88393800

H 3.08437500 -3.78064500 -2.32528500

H 1.59733300 -2.98816800 -0.56381600

Cl -0.46166700 -0.79221500 -2.99900500

C 2.81646400 -0.69886300 -3.11840600

H 3.89978700 -0.67536300 -3.30287900

H 2.40706700 -1.63135800 -3.53086600

H 2.34291600 0.12168200 -3.67239700

O 2.57038800 -0.55983400 -1.74869000

H 2.34637400 0.19483700 0.62971500

**M5-syn**

P -1.29028200 -1.11505500 0.41519800

C -0.59258800 -1.82695300 1.96420500

C -0.13653300 -3.15083300 1.90817000

C -0.40996200 -1.09693600 3.13696000

C 0.48788500 -3.73021700 3.00488100

H -0.26591900 -3.72069100 0.98978100

C 0.22660700 -1.67639300 4.23688400

H -0.76262500 -0.07256900 3.19873100

C 0.67631200 -2.98997200 4.17334900

H 0.83146600 -4.75905900 2.95111700

H 0.36517000 -1.09545200 5.14419600

H 1.16877300 -3.44070700 5.02965800

C -2.76432100 -2.19192100 0.17270900

C -3.12989600 -2.57601700 -1.12039900

C -3.51746900 -2.64912800 1.26148700

C -4.26309200 -3.36286000 -1.32207800

H -2.52645500 -2.25294200 -1.96485400

C -4.64861400 -3.43293600 1.05682100

H -3.20468600 -2.41343300 2.27557800

C -5.02886500 -3.78368100 -0.23827600

H -4.54265800 -3.64826300 -2.33154400

H -5.22631500 -3.77953100 1.90845600

H -5.91090800 -4.39659000 -0.39873200

C -0.56419000 2.24697100 -0.45868100

H -0.60342200 1.51688100 -1.27924800

C -0.92893000 3.62309400 -1.03801000

H -0.77288900 4.44195900 -0.33362900

H -0.35107300 3.84619000 -1.93399300

H -1.98669100 3.62423800 -1.32168900

P 1.21014100 1.99177200 0.17737400

C 1.43687700 3.07877800 1.72207900

C 0.82403200 4.48247300 1.70652300

H -0.26798700 4.43195800 1.68772300

H 1.10914200 5.00953000 2.62714600

H 1.16240600 5.08527200 0.85989600

C 0.80382500 2.29446000 2.88575900

H -0.27815700 2.18205900 2.76552100

H 1.24468200 1.29548500 2.97845700

H 0.98289600 2.83565100 3.82447000

C 2.93826900 3.18433400 2.02399600

H 3.47009600 3.81904900 1.31060500

H 3.07120300 3.62409200 3.02098900

H 3.41577200 2.19885200 2.02461900

C 2.29310400 2.66215800 -1.24231900

C 3.68541700 2.02081600 -1.09198900

H 3.61501200 0.93382100 -1.20613300

H 4.33922900 2.39709300 -1.88980900

H 4.17090500 2.23447500 -0.13540600

C 1.72220700 2.11034900 -2.56188200

H 1.51769100 1.03819100 -2.48830200

H 0.79949900 2.59841000 -2.88053500

H 2.46739300 2.25987800 -3.35439800

C 2.44743500 4.18395900 -1.34798100

H 1.48848100 4.70086000 -1.42773900

H 2.98837900 4.60490700 -0.49578200

H 3.02795000 4.42057900 -2.24958100

Rh 0.32451600 -1.10026500 -1.08679800

C -5.18220600 0.21249900 -0.76993100

Fe -3.66056500 1.42034900 0.09122200

C -4.21099100 0.51490100 -1.76160500

C -5.74334000 1.43902400 -0.31900800

C -5.11516600 2.49858700 -1.02786300

C -4.16881300 1.92329200 -1.91948100

C -3.01248700 0.60641000 1.94296400

C -3.29468800 1.97597000 2.12784300

C -2.43197400 2.71440900 1.27627700

C -1.59205800 1.81311400 0.56274700

C -1.96684800 0.48178800 0.97171100

H -3.55548000 -0.18063100 -2.26900500

H -6.48016000 1.55221000 0.46505200

H -5.29493100 3.55530700 -0.88102200

H -3.50284000 2.45470100 -2.58567000

H -3.53718400 -0.21596900 2.40904600

H -4.07191700 2.38640800 2.75823800

H -2.44948300 3.78834800 1.14848700

H -5.40673400 -0.77486100 -0.38507800

C 6.62471800 -0.50849700 0.05262000

C 6.60649900 0.27104600 1.20504200

C 5.45133100 0.33139800 1.97905000

C 4.31069700 -0.37006600 1.58833200

C 4.31666300 -1.12654400 0.42029100

H 7.52701000 -0.57185300 -0.54996500

H 7.49399900 0.81954900 1.50356900

H 5.43484800 0.92199700 2.88994700

H 3.41279000 -0.33525700 2.20097600

C 5.48852200 -1.21561100 -0.34509600

C 5.49589800 -2.05949400 -1.54420300

C 4.38199100 -2.58726600 -2.05922100

H 6.45960000 -2.24770100 -2.01240900

C 3.09357900 -1.92198200 0.01344500

C 3.01999100 -2.28817200 -1.48356100

H 3.12184600 -2.87950400 0.55221200

O 1.90140300 -1.27461000 0.44742300

H 4.42751600 -3.20647500 -2.95009100

H 2.36735900 -3.16809300 -1.58238100

Cl -1.00632500 -0.56617200 -3.03776100

C 2.28278300 -1.41773900 -3.59199600

H 3.26701800 -1.29213200 -4.05348000

H 1.88341500 -2.41783500 -3.80457200

H 1.57770400 -0.67757700 -3.96903800

O 2.37170600 -1.21567100 -2.18280400

H 2.01585400 -0.29119700 0.47675900

**PhO-TS-a**

P -1.50841600 1.09837300 0.34278300

C -0.71757800 2.54292500 -0.48106600

C -0.34048500 3.66371400 0.26894100

C -0.31009000 2.45978600 -1.81908200

C 0.41211700 4.68635100 -0.30822700

H -0.63622400 3.74350900 1.31118100

C 0.44356800 3.47972300 -2.39203100

H -0.57081100 1.58590500 -2.40646500

C 0.80765200 4.59575500 -1.63989800

H 0.68241700 5.55502500 0.28592800

H 0.75188600 3.39964100 -3.43011300

H 1.39426500 5.38986100 -2.09297400

C -2.49763900 1.94374100 1.64515600

C -2.48863900 1.44104900 2.94706300

C -3.26823900 3.07782800 1.35040100

C -3.25266400 2.06179100 3.93995900

H -1.88329000 0.56968600 3.18412600

C -4.01788900 3.69821200 2.34171000

H -3.27889100 3.47488800 0.33787600

C -4.01387300 3.18637100 3.64149800

H -3.24242700 1.66155200 4.94916100

H -4.60944800 4.57757700 2.10341400

H -4.60281900 3.66849300 4.41610200

C -2.86017800 -2.02609600 -0.30544600

H -2.94690300 -1.77053500 0.76108000

C -3.87525800 -3.13775400 -0.61064000

H -3.78496600 -3.51662700 -1.62997000

H -3.76866300 -3.98559300 0.06297700

H -4.89117400 -2.74266200 -0.49011900

P -1.01279100 -2.43165900 -0.43790200

C -0.63294000 -2.68091600 -2.29185200

C -1.58018800 -3.60426600 -3.07306800

H -2.55352800 -3.12449300 -3.21501200

H -1.15421700 -3.76158800 -4.07290100

H -1.73539800 -4.58405800 -2.62236000

C -0.66169000 -1.32256400 -3.02193400

H -1.66257400 -0.88699700 -3.05573100

H 0.03901900 -0.61696600 -2.57401400

H -0.35319800 -1.50599400 -4.06108900

C 0.81245400 -3.19780000 -2.38155800

H 0.92002700 -4.22439700 -2.01999100

H 1.12540400 -3.18483800 -3.43335600

H 1.49519500 -2.54836000 -1.82095400

C -0.81437200 -4.10293600 0.47199400

C 0.65959100 -4.20154400 0.90667200

H 0.88370200 -3.42928900 1.64728000

H 0.82445900 -5.18431200 1.36818000

H 1.35784100 -4.09568000 0.07205000

C -1.64785700 -4.09033300 1.76728900

H -1.47933000 -3.18369600 2.35108300

H -2.71827500 -4.21058100 1.59033300

H -1.32733900 -4.94271600 2.38033500

C -1.17503300 -5.36634300 -0.32093800

H -2.20513600 -5.35711100 -0.68745000

H -0.50694100 -5.53190200 -1.17049400

H -1.07169000 -6.23017100 0.34857600

Rh 0.38076600 -0.59629600 0.70081500

C -6.21420000 1.81996500 0.26290700

Fe -4.91074400 0.44329800 -0.72705000

C -5.71781100 0.89007600 1.21412700

C -6.93641700 1.09710300 -0.72458400

C -6.88087000 -0.28047200 -0.38644400

C -6.12549200 -0.40701700 0.81122300

C -3.36168700 1.38765300 -1.88381400

C -4.15435600 0.56924400 -2.71955300

C -4.05408100 -0.76350100 -2.24119800

C -3.19216700 -0.78483000 -1.10696700

C -2.75109000 0.56544000 -0.88820500

H -5.10566000 1.12557100 2.07402500

H -7.40144500 1.51552300 -1.60693500

H -7.29763100 -1.09372600 -0.96576600

H -5.87498400 -1.33229300 1.31338400

H -3.23046700 2.45834200 -1.97440100

H -4.76988400 0.90367800 -3.54367500

H -4.58632500 -1.61343600 -2.64625600

H -6.03944600 2.88676100 0.27208300

C 3.84459400 -2.77825200 0.04961500

C 4.62081300 -3.11775500 -1.05885800

C 4.78095800 -2.21450100 -2.10878200

C 4.13524600 -0.97664300 -2.05582500

C 3.34904800 -0.63915700 -0.96039400

H 3.71796100 -3.48887400 0.86429100

H 5.10252700 -4.09061900 -1.10174900

H 5.38519300 -2.47978700 -2.97073900

H 4.22088000 -0.27521800 -2.88348800

C 3.20478600 -1.53529100 0.11708100

C 2.35674400 -1.12315700 1.24853800

C 2.12943800 0.28502100 1.50624100

H 2.33614300 -1.78737800 2.10962100

C 2.41937400 0.55398900 -0.93821300

C 2.41513300 1.21578200 0.43761300

H 2.69435200 1.28121700 -1.71925900

O 1.11794800 0.11014700 -1.10650800

H 1.96870900 0.65148400 2.51506400

H 1.91727500 2.18453900 0.47376100

Cl -0.22500400 -1.21825100 3.02880300

O 4.02590700 1.83282500 0.67163700

H 4.66858700 1.08347000 0.96127600

C 5.47659600 -0.17766800 2.95466900

O 5.72857300 0.22461700 1.59953100

C 6.50247900 -0.77614200 0.90874700

C 6.65646700 -1.94464200 1.89012500

C 5.54873000 -1.69581400 2.92107400

H 6.24942700 0.25685300 3.60063500

H 4.49797700 0.21629700 3.23967900

H 5.95335100 -1.05928100 0.00749400

H 7.46306200 -0.33154100 0.62969400

H 6.54782000 -2.90572500 1.38261000

H 7.63882200 -1.91538400 2.37133900

H 4.59672100 -2.09762800 2.56776100

H 5.77173500 -2.12487700 3.90029100

C 4.56337500 2.62530500 -0.33558200

C 4.03616600 3.90001300 -0.49766400

C 5.57272800 2.13892000 -1.15967500

C 4.53406800 4.70679200 -1.51561100

H 3.24488800 4.24106900 0.16181000

C 6.07047400 2.96520300 -2.16344700

H 5.94354500 1.12668100 -1.02830400

C 5.55289600 4.24577900 -2.34631000

H 4.12639400 5.70340200 -1.65283600

H 6.86069600 2.59949600 -2.81120500

H 5.94113200 4.88139400 -3.13510300

**PhO-TS-b**

P 1.82922900 -1.06251500 0.03869700

C 1.72982300 -2.52436200 -1.07249900

C 1.59047100 -3.80284800 -0.51832300

C 1.58235600 -2.36901700 -2.45515000

C 1.33289300 -4.90526600 -1.32989400

H 1.68424100 -3.94108100 0.55541900

C 1.32053900 -3.47064300 -3.26421100

H 1.65764500 -1.38099200 -2.89732700

C 1.19497400 -4.74176600 -2.70550600

H 1.23571400 -5.89053200 -0.88374300

H 1.20648600 -3.33247000 -4.33526300

H 0.99069100 -5.59922200 -3.34003200

C 2.84767600 -1.73890100 1.41573700

C 2.48116700 -1.46862100 2.73486400

C 3.99217400 -2.50784100 1.16283200

C 3.26075100 -1.95332000 3.78913900

H 1.58637400 -0.88494000 2.93684700

C 4.76095100 -2.99375200 2.21313300

H 4.27874900 -2.72673400 0.13675800

C 4.39685100 -2.71263300 3.53208200

H 2.97037800 -1.73454200 4.81229000

H 5.64572300 -3.58923800 2.00636600

H 5.00011800 -3.08881600 4.35304200

C 2.07471200 2.41807200 -0.02228100

H 2.10318200 2.05531900 1.01560800

C 2.64565500 3.84249800 -0.04020600

H 2.57199300 4.30760700 -1.02508200

H 2.13556600 4.49512300 0.66660000

H 3.70606200 3.81405000 0.23987600

P 0.24488700 2.15669600 -0.44281000

C 0.05718600 2.48971900 -2.30949900

C 0.71552200 3.76219100 -2.85991100

H 1.80597700 3.67361600 -2.83059800

H 0.43294900 3.87344400 -3.91540000

H 0.42485600 4.67708100 -2.34042000

C 0.65354600 1.29822300 -3.08627300

H 1.74251100 1.25676000 -3.00820700

H 0.22176300 0.34884400 -2.75893900

H 0.40787300 1.43702000 -4.14863700

C -1.44765300 2.49144700 -2.62291900

H -1.98010900 3.33606800 -2.17577500

H -1.58052300 2.55445300 -3.71039200

H -1.90039500 1.55009600 -2.28807200

C -0.68656400 3.49774900 0.54816100

C -2.13946000 3.00995400 0.71435700

H -2.16317000 2.07336600 1.28231100

H -2.70981100 3.76611800 1.27115000

H -2.64799800 2.85909900 -0.24274100

C -0.09548800 3.56011900 1.96837800

H -0.02129100 2.56595900 2.41621400

H 0.88251500 4.04387000 2.00592800

H -0.77290800 4.15642000 2.59356800

C -0.70530500 4.90909300 -0.05020800

H 0.29831900 5.30671900 -0.22418200

H -1.25733600 4.95126000 -0.99369400

H -1.21103900 5.58219900 0.65490700

Rh -0.49426800 -0.22927200 0.23153100

C 6.43472100 -0.16329000 0.82682400

Fe 4.90114800 0.85479400 -0.25937000

C 5.50193400 0.38294000 1.74690500

C 7.00125100 0.90829500 0.08495900

C 6.41442500 2.11678700 0.54426000

C 5.48603400 1.79000700 1.57006800

C 3.98213600 -0.35277800 -1.78117300

C 4.55248400 0.80078600 -2.36443200

C 3.91609800 1.93373100 -1.79266600

C 2.94032500 1.49228100 -0.85289800

C 2.97607000 0.05378800 -0.85186800

H 4.88359100 -0.17834100 2.43408300

H 7.71492600 0.81587300 -0.72258200

H 6.60121000 3.10536400 0.14594400

H 4.85176700 2.48642400 2.10268600

H 4.25274700 -1.37834800 -1.99654200

H 5.36685200 0.82088900 -3.07601700

H 4.17245300 2.96369000 -1.99975100

H 6.64396000 -1.21522000 0.69034800

C -4.98696100 -1.10326300 -2.39455600

C -5.23300500 -2.01563400 -3.41682400

C -4.28440700 -2.98847200 -3.72395200

C -3.09312000 -3.04831700 -3.00583500

C -2.84238100 -2.14946700 -1.97358800

H -5.74405100 -0.36839300 -2.13640600

H -6.16621800 -1.96831700 -3.96909900

H -4.47450400 -3.69922000 -4.52272500

H -2.33783800 -3.79418400 -3.24196300

C -3.79388000 -1.16375000 -1.67246400

C -3.48070300 -0.21106200 -0.56046400

C -2.54990500 -0.67522400 0.47667600

C -1.53269200 -2.11193500 -1.22120800

C -1.74832900 -1.85085900 0.27017800

H -0.96849700 -3.04300300 -1.39415900

O -0.82523300 -0.97582600 -1.64750600

H -2.75623100 -0.31371200 1.47954900

H -1.57667500 -2.61924300 1.01930200

Cl -0.58090800 0.18200900 2.75088400

O -4.99065000 -0.00756400 0.23691200

H -3.40351700 0.83785400 -0.84646300

C -5.34652100 1.31516200 0.49451500

C -5.49383600 1.76229800 1.80191700

C -5.52861400 2.16472900 -0.59195600

C -5.83350100 3.09510400 2.01929100

H -5.35464700 1.08101600 2.63429100

C -5.85548900 3.49608000 -0.35663400

H -5.40302300 1.78789400 -1.60232400

C -6.00828400 3.96450500 0.94636400

H -5.95090500 3.45320400 3.03672900

H -5.99164500 4.16775700 -1.19783200

H -6.26183300 5.00389900 1.12514800

H -4.96476400 -0.58176900 1.11473800

C -4.19815200 -1.24148300 3.46440400

O -4.98348300 -1.48577100 2.27115500

C -4.79716400 -2.84604900 1.80801000

C -3.97515900 -3.54131000 2.89124900

C -3.20190500 -2.38756600 3.53890000

H -3.70584700 -0.26854300 3.37321600

H -4.89046100 -1.22880600 4.31325300

H -5.78686500 -3.28361100 1.65971100

H -4.26549100 -2.80753600 0.85081200

H -4.63619000 -4.01780600 3.62216200

H -3.32020400 -4.30720600 2.46980200

H -2.90740600 -2.59918700 4.56897600

H -2.30043300 -2.12504900 2.97884200

**PhO-b2**

P 1.79978400 -1.14461600 0.35362800

C 1.44255800 -2.77930800 -0.41460100

C 1.16388400 -3.88504200 0.39806000

C 1.24072100 -2.89193000 -1.79494300

C 0.71458200 -5.08151800 -0.15697200

H 1.29874300 -3.81338300 1.47384700

C 0.78658000 -4.08549700 -2.34773100

H 1.42505600 -2.03795200 -2.43832600

C 0.52181400 -5.18467900 -1.53188500

H 0.51242700 -5.93104100 0.48886900

H 0.63334100 -4.15359400 -3.42071200

H 0.17011500 -6.11611300 -1.96621800

C 2.77328400 -1.66406800 1.82811700

C 2.49084100 -1.08645700 3.06687800

C 3.79471300 -2.61915700 1.72983200

C 3.23597900 -1.45191600 4.19241000

H 1.68840900 -0.35659300 3.15042000

C 4.52736900 -2.98629400 2.85178100

H 4.01416100 -3.07630200 0.76762800

C 4.25057500 -2.39699400 4.08770600

H 3.01351300 -0.99428000 5.15163200

H 5.31596900 -3.72855400 2.76540200

H 4.82618200 -2.67994300 4.96408900

C 2.53621200 2.14860300 -0.49879500

H 2.55383600 2.02216700 0.59380000

C 3.31591600 3.42908000 -0.83343100

H 3.27897400 3.67441500 -1.89656200

H 2.93927900 4.29219400 -0.28669800

H 4.36936300 3.29471400 -0.55757200

P 0.66976100 2.08913700 -0.83635600

C 0.46903000 2.03241000 -2.73059700

C 1.31333100 3.01602700 -3.55111900

H 2.37148700 2.73973300 -3.51005500

H 1.00633300 2.94870700 -4.60368800

H 1.21144300 4.05780900 -3.24152600

C 0.81999600 0.61230200 -3.21742000

H 1.88666200 0.39409100 -3.12280800

H 0.23893700 -0.14750200 -2.68780800

H 0.56942600 0.55346100 -4.28628300

C -1.02307600 2.22923100 -3.03774100

H -1.37914000 3.23734200 -2.80772200

H -1.18969500 2.05457900 -4.10822800

H -1.61909800 1.49306300 -2.48485400

C 0.00410800 3.75260900 -0.17545000

C -1.50002800 3.55811700 0.09424400

H -1.66047200 2.77213800 0.84134100

H -1.91536800 4.49627900 0.48652400

H -2.06136600 3.30425500 -0.81071400

C 0.64644400 4.02933800 1.19619100

H 0.55757200 3.16719100 1.86207100

H 1.69624600 4.32132300 1.12866200

H 0.10892500 4.86628000 1.66142500

C 0.19124900 4.98259900 -1.07178200

H 1.24000800 5.16776800 -1.31942400

H -0.37055800 4.90783700 -2.00768500

H -0.18212900 5.86594400 -0.53635400

Rh -0.41769600 0.06311000 0.37081000

C 6.52262100 -0.77089000 0.87692300

Fe 5.09437400 0.17915000 -0.39840200

C 5.69680800 0.07717500 1.66067200

C 7.19347500 0.03309800 -0.08404900

C 6.77701800 1.37761000 0.10257300

C 5.85066900 1.40270700 1.18074700

C 3.95833400 -1.19323300 -1.60052500

C 4.66161400 -0.29565500 -2.43509700

C 4.21307800 1.01711200 -2.13307500

C 3.22456900 0.94428900 -1.10913100

C 3.05818100 -0.44634200 -0.78074800

H 5.03391000 -0.23709200 2.45508600

H 7.86263800 -0.32302200 -0.85570900

H 7.06914200 2.22389300 -0.50526400

H 5.32336700 2.27317000 1.54870200

H 4.07268000 -2.26927400 -1.57925700

H 5.44052600 -0.55467400 -3.13968700

H 4.60627700 1.92643100 -2.56668100

H 6.59483300 -1.84544400 0.97328600

C -5.04097300 -0.80487900 -2.02941600

C -5.42855800 -1.89210900 -2.81235200

C -4.62862900 -3.03267800 -2.85718800

C -3.44459000 -3.07953800 -2.12353900

C -3.05752300 -2.00438700 -1.32663900

H -5.65343800 0.09366800 -2.00952600

H -6.34196100 -1.84044400 -3.39718100

H -4.92149900 -3.87774700 -3.47326200

H -2.79659000 -3.95157600 -2.17027100

C -3.86619600 -0.86033300 -1.27719900

C -3.43844900 0.29400800 -0.40037700

C -2.49775100 -0.03425600 0.71519900

C -1.73087500 -1.95078100 -0.60441700

C -1.86720700 -1.32209400 0.78270500

H -1.28428700 -2.95813700 -0.55694300

O -0.92238000 -1.03676700 -1.29470200

H -2.67764200 0.51393700 1.63688100

H -1.75922300 -1.89146400 1.70255000

Cl -0.28709800 1.02107400 2.69882800

O -4.79811100 0.77301000 0.23217100

H -3.18281900 1.19270000 -0.96816600

C -4.78597300 2.01841100 0.88342100

C -4.66906200 2.08132600 2.26452000

C -4.89816000 3.15156500 0.09088500

C -4.65597400 3.33652600 2.86758200

H -4.57996000 1.17228600 2.85032300

C -4.88178500 4.39739100 0.70923400

H -4.99099700 3.04903600 -0.98597000

C -4.75932200 4.48983300 2.09444600

H -4.54964600 3.40729200 3.94459700

H -4.95879300 5.29608500 0.10615000

H -4.73817400 5.46383500 2.57186100

H -5.23340900 -0.02579900 0.83480200

C -7.17813200 -1.23150700 0.78837200

O -5.92814300 -0.98076900 1.48460100

C -5.22207000 -2.23287500 1.71859900

C -5.85235600 -3.21130000 0.74255000

C -7.31251500 -2.74976500 0.72749900

H -7.97388500 -0.73142400 1.34304300

H -7.09025900 -0.79482700 -0.21124900

H -4.15936500 -2.04416200 1.55370500

H -5.39684600 -2.51090800 2.76232900

H -5.40104000 -3.09565100 -0.24722200

H -5.73376400 -4.24749600 1.06582200

H -7.85196100 -3.06975500 -0.16636700

H -7.84534900 -3.12249900 1.60784800

**PhO-b3**

P 2.16579000 -1.08791000 0.08683200

C 2.01083400 -2.50626800 -1.07618400

C 1.97668300 -3.81190800 -0.57050500

C 1.71637900 -2.30267000 -2.42883100

C 1.67471000 -4.88924800 -1.39995600

H 2.18574000 -3.99085600 0.48041500

C 1.40987500 -3.37933800 -3.25586400

H 1.71424500 -1.29627900 -2.83379000

C 1.38755900 -4.67623700 -2.74564600

H 1.65996000 -5.89488600 -0.99009200

H 1.18143500 -3.20114000 -4.30246800

H 1.14899800 -5.51481900 -3.39336000

C 3.32198800 -1.79679500 1.33314300

C 3.02949800 -1.63404600 2.68801600

C 4.48297900 -2.48717700 0.95745500

C 3.90124600 -2.14028800 3.65675800

H 2.12099000 -1.11299000 2.98210900

C 5.34345400 -2.99558700 1.92278700

H 4.71059300 -2.62730600 -0.09697700

C 5.05524300 -2.81694900 3.27815100

H 3.66780200 -2.00365500 4.70835400

H 6.24023500 -3.53085800 1.62317900

H 5.73097200 -3.21079000 4.03166700

C 2.26571600 2.39431800 0.12003500

H 2.37928000 2.00712500 1.14336100

C 2.77880300 3.84152500 0.10271500

H 2.63073500 4.32503800 -0.86493500

H 2.28865300 4.45902200 0.85363200

H 3.85436100 3.84832400 0.31899200

P 0.42168500 2.06685000 -0.18039100

C 0.10651100 2.42970500 -2.02650500

C 0.70283900 3.72372400 -2.59615300

H 1.79353100 3.65260600 -2.64645000

H 0.34329400 3.85296400 -3.62614100

H 0.43764900 4.62247300 -2.03598400

C 0.66601300 1.25936700 -2.85987600

H 1.75794000 1.22741600 -2.84982600

H 0.26043700 0.30165100 -2.52021500

H 0.35479400 1.41296700 -3.90316600

C -1.41374500 2.39891100 -2.24722700

H -1.93924200 3.21911600 -1.74836300

H -1.61717600 2.48105500 -3.32259400

H -1.81253200 1.43542900 -1.90824600

C -0.49389300 3.36634800 0.88032300

C -1.90518600 2.79997400 1.13531300

H -1.84053100 1.85912600 1.69248000

H -2.47703900 3.52676600 1.73055100

H -2.45440800 2.61587400 0.20525700

C 0.17721500 3.44768200 2.26334200

H 0.32714700 2.45470900 2.69473300

H 1.13043600 3.97890000 2.24472800

H -0.48862900 4.00678200 2.93445800

C -0.61655400 4.78149000 0.30345500

H 0.35745900 5.22977000 0.08771800

H -1.21260400 4.81140300 -0.61383200

H -1.11712800 5.42177400 1.04272400

Rh -0.22041300 -0.35204800 0.52512500

C 6.77534100 -0.02335100 0.57605600

Fe 5.12880500 0.96063500 -0.36545100

C 5.87906900 0.43579100 1.57661500

C 7.24117900 1.10549100 -0.15064600

C 6.62792700 2.26246900 0.39791400

C 5.78405600 1.84659500 1.46393300

C 4.15519900 -0.23513900 -1.86536800

C 4.63518600 0.96097100 -2.44457400

C 3.99423600 2.04535700 -1.78966000

C 3.10832300 1.53067800 -0.79878600

C 3.20514000 0.09544500 -0.85188100

H 5.32975500 -0.18393100 2.27228600

H 7.90362200 1.08391400 -1.00549700

H 6.74168300 3.27456200 0.03253900

H 5.15059700 2.48719000 2.06379400

H 4.44817500 -1.24093100 -2.13746900

H 5.39342800 1.04201200 -3.21169100

H 4.19490700 3.09131000 -1.97704700

H 7.02325800 -1.05696700 0.37836500

C -4.91771600 -1.46900800 -1.69988600

C -5.24085900 -2.44050000 -2.64545700

C -4.28761500 -3.38055600 -3.02554100

C -3.01407100 -3.34577800 -2.46102100

C -2.68625900 -2.38535900 -1.50951100

H -5.67423400 -0.74448500 -1.40526400

H -6.23445800 -2.46072800 -3.08252500

H -4.53526400 -4.13645000 -3.76497500

H -2.25413100 -4.06321700 -2.76166100

C -3.64940100 -1.44297200 -1.12611400

C -3.23081800 -0.41868700 -0.09276400

C -2.20581000 -0.87583500 0.91593100

C -1.30914500 -2.23901400 -0.89783600

C -1.40699700 -2.02544100 0.61509100

H -0.68574300 -3.11264200 -1.15061500

O -0.75506500 -1.03133300 -1.35026600

H -2.46289600 -0.65168000 1.94931100

H -1.16591100 -2.79618100 1.34138900

Cl -0.00770300 0.05285500 2.99870300

O -4.46370800 0.04542100 0.68729700

H -2.97604100 0.53566500 -0.56124800

C -5.27956300 -0.79369200 1.46851100

C -5.01371500 -2.14605700 1.62239300

C -6.35479000 -0.16940400 2.08881600

C -5.87786100 -2.88728600 2.42587900

H -4.15887800 -2.60497800 1.14108000

C -7.21089900 -0.93178700 2.87719800

H -6.49889500 0.90230500 1.97462000

C -6.97363100 -2.29321600 3.04604200

H -5.68369900 -3.94568000 2.56306600

H -8.05159800 -0.45437500 3.36942200

H -7.63459400 -2.88786200 3.66718100

H -5.01350100 0.77610500 0.14033900

C -7.06286200 1.62967400 -0.87862500

O -5.64803000 1.75670400 -0.59942800

C -5.31113300 3.12137100 -0.23678200

C -6.57474100 3.92296700 -0.52797500

C -7.68177700 2.89064500 -0.29269900

H -7.18472200 1.57185400 -1.96434600

H -7.41324200 0.70049900 -0.42123500

H -5.04181300 3.13215300 0.82493600

H -4.44463600 3.41777100 -0.83004700

H -6.65875200 4.80074800 0.11513500

H -6.58141000 4.25564500 -1.57018700

H -7.86504800 2.76178200 0.77963600

H -8.62542300 3.15115800 -0.77549500

**PhO-b4**

P -1.86058400 1.06038200 0.49390600

C -1.70024900 2.80358200 -0.07416600

C -1.44252200 3.81838300 0.85530800

C -1.62875800 3.10747000 -1.43848800

C -1.14179300 5.11140600 0.43248300

H -1.47492200 3.59912800 1.91900100

C -1.32259200 4.39780900 -1.85990400

H -1.79481000 2.32733900 -2.17405200

C -1.07853300 5.40480600 -0.92696000

H -0.95355400 5.88689400 1.16904500

H -1.26665100 4.61411200 -2.92262200

H -0.84095700 6.41136600 -1.25862700

C -2.75575200 1.29166700 2.08631700

C -2.34388100 0.55662300 3.19946100

C -3.83954300 2.17254100 2.20563300

C -3.02182600 0.69121600 4.41459300

H -1.48969400 -0.11236800 3.11837300

C -4.50656300 2.31016500 3.41675000

H -4.15660300 2.75494300 1.34352400

C -4.09938900 1.56321600 4.52492600

H -2.69706900 0.11397400 5.27499500

H -5.34420100 2.99694700 3.50026400

H -4.62189400 1.66818000 5.47119800

C -2.39644000 -2.14370700 -0.71969700

H -2.33881400 -2.14405400 0.37896900

C -3.09684300 -3.43909500 -1.15514400

H -3.11426600 -3.56060100 -2.24001200

H -2.62124100 -4.32376000 -0.73535200

H -4.13625600 -3.42368400 -0.80482900

P -0.57163700 -1.88034600 -1.17769700

C -0.52652200 -1.57338800 -3.05629600

C -1.35841600 -2.51588000 -3.93505800

H -2.42759500 -2.34103400 -3.78189800

H -1.14528100 -2.29267300 -4.98917800

H -1.14988100 -3.57480700 -3.77042000

C -1.01483900 -0.13627000 -3.32736200

H -2.08385500 -0.01942300 -3.13357800

H -0.44985800 0.59630300 -2.74336400

H -0.85286000 0.07618800 -4.39369200

C 0.94707400 -1.61160500 -3.48679600

H 1.38459500 -2.61219800 -3.42562200

H 1.01900100 -1.28418700 -4.53157100

H 1.53668700 -0.91375800 -2.88004100

C 0.25596900 -3.55813200 -0.79167300

C 1.75478500 -3.26674400 -0.61070100

H 1.89401100 -2.50211800 0.15876300

H 2.25661600 -4.19215900 -0.28912700

H 2.24232400 -2.92494900 -1.53013500

C -0.23883500 -4.03244700 0.58788600

H -0.14336500 -3.24256800 1.33835500

H -1.27035000 -4.38874700 0.57826600

H 0.38782000 -4.87745600 0.90594100

C 0.08595500 -4.69126800 -1.80962400

H -0.96283700 -4.93513000 -1.99768300

H 0.55865100 -4.46257400 -2.76937300

H 0.56899400 -5.59591900 -1.41504400

Rh 0.51008000 0.10403900 0.24925700

C -6.49320800 0.25200400 1.24376700

Fe -5.09918000 -0.43229600 -0.22313000

C -5.56801500 -0.62879800 1.86297900

C -7.17865700 -0.47040800 0.22980700

C -6.67149200 -1.79616900 0.21825100

C -5.67431100 -1.89214400 1.22716900

C -4.15464800 1.16540600 -1.30228500

C -4.83919700 0.31976600 -2.20397700

C -4.27030000 -0.97683700 -2.09690000

C -3.22324800 -0.94287900 -1.13129400

C -3.14457300 0.40455200 -0.63820400

H -4.87457900 -0.37160000 2.65206500

H -7.91662900 -0.06817000 -0.45105600

H -6.95962800 -2.58013400 -0.46963800

H -5.07711100 -2.76572900 1.45359600

H -4.35398500 2.21599900 -1.13510100

H -5.68227200 0.59538500 -2.82300300

H -4.61275400 -1.85554900 -2.62647000

H -6.61918500 1.30011800 1.47825800

C 4.87015400 1.81424700 -2.24595900

C 5.08323700 3.02460500 -2.90216400

C 4.14140700 4.04526500 -2.79902500

C 2.98654400 3.85176400 -2.04452100

C 2.76697800 2.64930200 -1.37872400

H 5.60954800 1.02063700 -2.31828600

H 5.98383700 3.16841300 -3.49111800

H 4.30544900 4.98912700 -3.31046900

H 2.23486600 4.63406300 -1.96806700

C 3.71964600 1.62829400 -1.48274200

C 3.46363000 0.34214600 -0.73177000

C 2.57232300 0.44708500 0.48412000

C 1.49892500 2.35187600 -0.60822500

C 1.79935400 1.62874500 0.70494000

H 0.93519900 3.28381600 -0.44231800

O 0.76039600 1.39699700 -1.32317800

H 2.95371300 -0.07819000 1.35819300

H 1.70051900 2.10369300 1.67735600

Cl 0.65486000 -1.17812200 2.44126700

O 4.81638200 -0.21946500 -0.29845000

H 3.16535400 -0.46392700 -1.41005700

C 5.66465100 0.44225900 0.61152100

C 5.38149000 1.68466500 1.16206900

C 6.82497100 -0.25634000 0.92905400

C 6.30010700 2.22366200 2.06208300

H 4.47426600 2.21673600 0.90701500

C 7.72445600 0.29612700 1.83430800

H 7.02411300 -1.21348600 0.45548500

C 7.46281100 1.53922700 2.40373600

H 6.09226600 3.19382800 2.50102100

H 8.63126200 -0.24427100 2.08502500

H 8.16347400 1.97336100 3.10882200

H 4.63294000 -1.26363600 0.09393900

C 5.18281100 -3.56499500 0.20768100

O 4.41102400 -2.40679100 0.60005200

C 4.00214300 -2.47925700 2.00346800

C 4.23428200 -3.93854600 2.35567200

C 5.46804500 -4.29074800 1.51704900

H 4.56225600 -4.15604000 -0.47092300

H 6.07529000 -3.22096300 -0.32187800

H 4.65485600 -1.80450300 2.56946300

H 2.95989900 -2.15245800 2.07241000

H 4.38846800 -4.07750300 3.42708800

H 3.37020900 -4.53596200 2.04781100

H 6.37209300 -3.88629800 1.98459000

H 5.60290100 -5.36381600 1.36827900

**PhO-b5**

P -2.06637400 0.97611200 0.44933500

C -2.04790100 2.75332200 -0.02345500

C -2.08536700 3.73877000 0.97080900

C -1.78433400 3.13952400 -1.34217300

C -1.88337600 5.07921100 0.65116300

H -2.27166600 3.45938500 2.00424300

C -1.57730600 4.47844500 -1.65957800

H -1.72619600 2.38787000 -2.12207200

C -1.62597400 5.45354800 -0.66514200

H -1.92311800 5.82967900 1.43520300

H -1.36743600 4.75773000 -2.68773400

H -1.46291600 6.49787700 -0.91452300

C -3.24012300 0.99495800 1.86826300

C -2.91955100 0.24925600 3.00426300

C -4.44297600 1.71315400 1.83435000

C -3.80166200 0.20942400 4.08735700

H -1.97768100 -0.29304400 3.04249500

C -5.31542900 1.67728500 2.91562700

H -4.69373200 2.30380700 0.95613500

C -4.99689000 0.91900000 4.04480800

H -3.54395600 -0.37504900 4.96536000

H -6.24516300 2.23829900 2.88152300

H -5.68043300 0.88913700 4.88813900

C -1.90660600 -2.18743600 -0.95208700

H -2.02346800 -2.24502000 0.14013000

C -2.31999600 -3.54283600 -1.54454800

H -2.13682200 -3.60326500 -2.61933600

H -1.79299500 -4.37328000 -1.07700200

H -3.39324000 -3.69783900 -1.37949800

P -0.09632700 -1.62321000 -1.09778000

C 0.17402800 -1.17146100 -2.92988500

C -0.37156700 -2.15327900 -3.97420100

H -1.46530200 -2.14812600 -3.97239400

H -0.05105500 -1.82012900 -4.97053100

H -0.02577800 -3.18007600 -3.83964800

C -0.47512700 0.20164900 -3.19678100

H -1.56648400 0.15454800 -3.17879000

H -0.12082900 0.95392500 -2.48500600

H -0.18098500 0.51973400 -4.20703800

C 1.68278100 -0.96541000 -3.13541900

H 2.26134900 -1.89085000 -3.05285500

H 1.84932900 -0.56275700 -4.14251800

H 2.05385700 -0.22262000 -2.41848100

C 0.92838900 -3.18236800 -0.68797000

C 2.33534700 -2.69986700 -0.28896600

H 2.27265600 -1.96631400 0.52631200

H 2.90931600 -3.57645300 0.04814300

H 2.88492700 -2.24221800 -1.11751700

C 0.33258600 -3.82028900 0.57999200

H 0.19527600 -3.08031500 1.37465300

H -0.62200400 -4.31795500 0.40184500

H 1.02991700 -4.59012600 0.94210300

C 1.07345500 -4.25464100 -1.77391000

H 0.10906800 -4.63700600 -2.11795300

H 1.62460800 -3.88952000 -2.64528400

H 1.63682600 -5.10382900 -1.36268900

Rh 0.43385200 0.35117600 0.60331400

C -6.57349000 -0.48110500 0.37489400

Fe -4.89177300 -0.89750500 -0.87433300

C -5.65860900 -1.29911300 1.08805300

C -6.99762800 -1.19505900 -0.77804000

C -6.33977300 -2.45304400 -0.78095500

C -5.51154000 -2.51557400 0.37313000

C -4.01739200 0.86979300 -1.71937600

C -4.40916800 -0.01081700 -2.75291400

C -3.68497600 -1.22071500 -2.58732300

C -2.83152000 -1.09662900 -1.45306000

C -3.03651000 0.21870600 -0.91091800

H -5.13234600 -1.02461600 1.99240800

H -7.66339300 -0.82555800 -1.54628300

H -6.41960600 -3.20979600 -1.55022700

H -4.85772800 -3.33360900 0.64531400

H -4.39263100 1.87176100 -1.55617000

H -5.16604300 0.17801900 -3.50225500

H -3.80345700 -2.10702100 -3.19620900

H -6.86138100 0.52569000 0.64380600

C 5.00481600 2.89841100 -0.57911200

C 5.19211800 4.22550500 -0.97059900

C 4.10351100 5.08243200 -1.08161000

C 2.82407300 4.61499800 -0.78400200

C 2.62701800 3.29836200 -0.38576400

H 5.87089500 2.25047200 -0.49077400

H 6.19401200 4.58379600 -1.18730400

H 4.25038700 6.11346100 -1.38974200

H 1.96250600 5.27573500 -0.85016800

C 3.72596200 2.42818400 -0.29637900

C 3.40127200 1.00479600 0.11858000

C 2.35011300 0.94685200 1.21730200

C 1.27137300 2.74935300 0.00824400

C 1.39958900 2.00086200 1.33514600

H 0.53745200 3.56865700 0.06373000

O 0.86849400 1.72744600 -0.86799700

H 2.70245900 0.49504100 2.14042100

H 1.08463100 2.41862800 2.28664100

Cl 0.34358800 -1.04201400 2.74737200

H 3.06281600 0.43144500 -0.74476400

H 4.42476300 -0.61943200 1.75006000

C 3.58868400 -1.65926900 3.51773700

O 4.59908700 -1.39555300 2.45743800

C 5.04204200 -2.65047500 1.82038200

C 4.41229000 -3.74390000 2.67178900

C 3.15452600 -3.08478500 3.24869500

H 4.12924300 -1.52914900 4.45642300

H 2.77236800 -0.94493800 3.40899200

H 4.67363000 -2.63170000 0.79409800

H 6.13135800 -2.63121300 1.82675000

H 4.18335300 -4.62434000 2.06665100

H 5.09786100 -4.04419400 3.46991900

H 2.32737900 -3.07559800 2.53414500

H 2.80581300 -3.56691400 4.16391700

C 5.56820800 -0.16043800 -0.19157300

C 5.34144400 -0.51771400 -1.51836900

C 6.82765900 -0.31856200 0.38759300

C 6.39470000 -1.05855700 -2.25440600

H 4.37444600 -0.36925500 -1.98418600

C 7.86462000 -0.86632300 -0.35959200

H 6.97557500 0.00584200 1.41386600

C 7.65099800 -1.24275800 -1.68380100

H 6.22192700 -1.33415500 -3.28983100

H 8.84436500 -0.98557700 0.09220000

H 8.46052900 -1.66406100 -2.27013900

O 4.58421300 0.32048400 0.65812900

**PhO-b6**

P -1.92336700 1.08386500 -0.03597500

C -1.81807400 2.47149200 -1.23940600

C -1.78338400 3.79414800 -0.78134200

C -1.56269700 2.22084600 -2.59237400

C -1.51987400 4.84369900 -1.65874500

H -1.96372900 4.00811500 0.26843100

C -1.29543600 3.26962100 -3.46675200

H -1.55862500 1.20009900 -2.95978800

C -1.27313700 4.58433300 -3.00433500

H -1.50467000 5.86388600 -1.28668200

H -1.09538800 3.05656400 -4.51253400

H -1.06514400 5.40095500 -3.68948200

C -3.02718800 1.80868600 1.24712700

C -2.68658600 1.64358900 2.59057200

C -4.19880200 2.50203100 0.91406000

C -3.51928300 2.15330200 3.59075200

H -1.76998700 1.11955000 2.85232700

C -5.02025800 3.01547200 1.91032600

H -4.46492900 2.63853100 -0.13187700

C -4.68277100 2.83657600 3.25408200

H -3.24951200 2.01453300 4.63339500

H -5.92575300 3.55331000 1.64383300

H -5.32853700 3.23337000 4.03189800

C -2.01953700 -2.38357000 0.11024600

H -2.09516900 -1.94932800 1.11799600

C -2.54374000 -3.82523000 0.17987100

H -2.41959900 -4.36087100 -0.76328600

H -2.04174300 -4.40508500 0.95269000

H -3.61438400 -3.81151000 0.41849000

P -0.18211400 -2.08538100 -0.26978300

C 0.05816400 -2.52892900 -2.10954900

C -0.60043900 -3.82304900 -2.60221600

H -1.69010800 -3.72657800 -2.60149600

H -0.29553200 -3.99692100 -3.64317800

H -0.32499600 -4.70709500 -2.02353100

C -0.49452700 -1.37007800 -2.96303200

H -1.58271400 -1.29054800 -2.90010000

H -0.03600800 -0.41658900 -2.68269000

H -0.24376300 -1.57692900 -4.01320100

C 1.56770300 -2.56909500 -2.38996000

H 2.08637100 -3.39103600 -1.88937800

H 1.72176000 -2.68777300 -3.47035200

H 2.02297200 -1.61752400 -2.09526100

C 0.75389300 -3.35131700 0.80861800

C 2.19474400 -2.82452100 0.95653200

H 2.18489700 -1.82606300 1.41106900

H 2.76062200 -3.50454800 1.60859200

H 2.72968500 -2.76873800 0.00359300

C 0.14503600 -3.32839200 2.22213500

H 0.05610800 -2.30811700 2.60443500

H -0.83153300 -3.81319900 2.27682700

H 0.81572500 -3.88098100 2.89370700

C 0.80770600 -4.79944200 0.30758500

H -0.18589400 -5.23175000 0.16235100

H 1.36063900 -4.89449000 -0.63123800

H 1.32823800 -5.41170900 1.05636400

Rh 0.47863200 0.39417600 0.34250900

C -6.51654300 0.04425400 0.61739900

Fe -4.90148300 -0.97399000 -0.34226200

C -5.60270100 -0.40496600 1.60665400

C -7.00987700 -1.09349600 -0.07643000

C -6.39593200 -2.24597300 0.48058200

C -5.52451800 -1.81848600 1.51930500

C -3.97196000 0.17682600 -1.90138700

C -4.46425300 -1.03761000 -2.43096700

C -3.80295500 -2.10081400 -1.76125400

C -2.89066200 -1.55443600 -0.81265000

C -2.99309700 -0.12223400 -0.90550900

H -5.03228100 0.22249400 2.27801500

H -7.69048800 -1.08101700 -0.91703700

H -6.52803900 -3.26383000 0.13807600

H -4.88530700 -2.45429200 2.11800900

H -4.27606100 1.17351400 -2.19403600

H -5.24323900 -1.14280900 -3.17392100

H -4.00536200 -3.15272600 -1.91000400

H -6.76035100 1.07613700 0.40568800

C 5.20052400 1.64693400 -1.83171600

C 5.52204000 2.67028400 -2.72607300

C 4.53682500 3.55259000 -3.15583300

C 3.23008600 3.42157800 -2.68388600

C 2.89992100 2.41212600 -1.78775700

H 5.97441300 0.96338300 -1.49073500

H 6.54175900 2.77295100 -3.08456100

H 4.78536400 4.34650700 -3.85403500

H 2.45330800 4.11050600 -3.00786300

C 3.89408500 1.51182700 -1.37152000

C 3.45324300 0.42810500 -0.41327600

C 2.46190400 0.93054400 0.62173100

C 1.51860100 2.23584100 -1.18783500

C 1.64891700 2.07126700 0.32920500

H 0.87572700 3.08947700 -1.45949300

O 0.96306000 1.00452500 -1.57238500

H 2.73469300 0.73537100 1.64990000

H 1.43563800 2.86878600 1.03673400

Cl 0.47697100 0.10274400 2.89506400

O 4.62909700 -0.10439800 0.31629100

H 3.04789200 -0.42641000 -0.95535000

C 5.12122300 -1.35528500 -0.02846200

C 5.60988100 -2.16233300 0.99335900

C 5.12226600 -1.78215500 -1.35297000

C 6.09413800 -3.42861900 0.68299000

H 5.59620600 -1.80999400 2.02073900

C 5.59616800 -3.05912700 -1.64338300

H 4.75624900 -1.13113300 -2.14059700

C 6.07952200 -3.88534400 -0.63264100

H 6.46889100 -4.06498100 1.47808500

H 5.58712700 -3.40313900 -2.67253600

H 6.44468700 -4.87908900 -0.86841200

H 4.77946500 0.30285100 1.56781000

C 4.07648900 0.35044800 3.65619100

O 4.98745200 0.75112000 2.55637500

C 5.04331400 2.21858300 2.43279800

C 4.50694800 2.70527700 3.76774200

C 3.44530900 1.65501300 4.11453600

H 3.35014300 -0.36435300 3.25976600

H 4.72120700 -0.11369400 4.40415700

H 6.08355700 2.46892300 2.22731400

H 4.40163400 2.49676900 1.59108400

H 5.30660000 2.72159600 4.51495900

H 4.08996600 3.71028200 3.68124400

H 3.21179000 1.62392400 5.18033700

H 2.50998400 1.81962800 3.56985300

**PhN-TS-a**

P -1.60442900 1.07264000 0.34894500

C -0.82399100 2.53273700 -0.45380800

C -0.44276800 3.63707400 0.31824500

C -0.43863000 2.48381300 -1.79930400

C 0.29381300 4.67773600 -0.24548800

H -0.72326500 3.68948600 1.36658400

C 0.29612000 3.52439700 -2.35963800

H -0.70042500 1.62101400 -2.40250000

C 0.66427200 4.62367800 -1.58610200

H 0.57003200 5.53304200 0.36499300

H 0.58950400 3.47230200 -3.40367400

H 1.23745200 5.43254500 -2.02915700

C -2.58678700 1.88987900 1.67398900

C -2.56680400 1.37390300 2.97031000

C -3.36549500 3.02276400 1.39585300

C -3.32676000 1.98065800 3.97492700

H -1.95858300 0.50155500 3.19407700

C -4.11190800 3.62841200 2.39859100

H -3.38472500 3.43109000 0.38794500

C -4.09594400 3.10394900 3.69325400

H -3.30729400 1.56974300 4.97970100

H -4.70961400 4.50694100 2.17308200

H -4.68193700 3.57523600 4.47667300

C -2.91084500 -2.05162300 -0.30087500

H -2.99349300 -1.80899100 0.76903500

C -3.90761100 -3.17866400 -0.61255100

H -3.82184000 -3.53792700 -1.63926600

H -3.78172800 -4.03524200 0.04545300

H -4.92792300 -2.80123300 -0.47524900

P -1.06196200 -2.43897800 -0.45648700

C -0.69044200 -2.63678000 -2.32034700

C -1.63288900 -3.56350100 -3.10570300

H -2.61375700 -3.09511700 -3.23246300

H -1.21149300 -3.69485100 -4.11084400

H -1.76994300 -4.55388800 -2.67495300

C -0.75891700 -1.27178700 -3.03623100

H -1.77037600 -0.86184300 -3.05891800

H -0.07027300 -0.55368300 -2.59217700

H -0.45622700 -1.44256100 -4.07896400

C 0.76309000 -3.12561600 -2.43476400

H 0.90016300 -4.14934700 -2.07486500

H 1.05536200 -3.10676100 -3.49220100

H 1.44190300 -2.46072900 -1.88910500

C -0.83545700 -4.13009800 0.41431300

C 0.63913800 -4.21280500 0.84751100

H 0.82193600 -3.51549900 1.66804000

H 0.84447200 -5.23058300 1.20437100

H 1.33722900 -3.98485500 0.03756100

C -1.67398500 -4.19040500 1.70507700

H -1.56674900 -3.28878300 2.30911700

H -2.73253700 -4.37866700 1.51970000

H -1.30109000 -5.03273300 2.30215600

C -1.17086400 -5.37179800 -0.42408300

H -2.20214200 -5.37085900 -0.78728800

H -0.50010500 -5.49696200 -1.27771500

H -1.05007900 -6.25346700 0.21832800

Rh 0.26201800 -0.64088100 0.66180900

C -6.31125300 1.74539700 0.32561100

Fe -5.00490900 0.38897200 -0.68700100

C -5.79873600 0.81229300 1.26497900

C -7.03536500 1.02424800 -0.66168200

C -6.96477900 -0.35547100 -0.33550200

C -6.19828500 -0.48509700 0.85475800

C -3.48109200 1.36077000 -1.85714900

C -4.27178500 0.53539700 -2.68788700

C -4.14908700 -0.79836900 -2.21761800

C -3.27403900 -0.81317200 -1.09320300

C -2.84996900 0.54120300 -0.87316100

H -5.18197800 1.04640500 2.12195500

H -7.51129900 1.44588300 -1.53663500

H -7.37962700 -1.16755100 -0.91779300

H -5.93741000 -1.41200800 1.34856300

H -3.36389700 2.43338900 -1.94337300

H -4.90028100 0.86561400 -3.50383700

H -4.67423200 -1.65266600 -2.62253000

H -6.14579500 2.81360800 0.34211100

C 3.79556900 -2.76101400 -0.08264900

C 4.56276200 -3.01833800 -1.21899700

C 4.67658500 -2.05563400 -2.22048900

C 4.00992200 -0.83461000 -2.08570700

C 3.23423900 -0.57790700 -0.96143100

H 3.70345900 -3.51357900 0.69853500

H 5.07313000 -3.97205200 -1.32093000

H 5.26936400 -2.25890100 -3.10718700

H 4.07168200 -0.08402000 -2.87100300

C 3.11627500 -1.54719400 0.05251300

C 2.24647300 -1.23626700 1.19947200

C 2.08666100 0.15398600 1.58211800

H 2.22645800 -1.96455700 2.00620800

C 2.27040100 0.58479900 -0.85587700

C 2.20560700 1.11933200 0.57220100

H 2.53111900 1.38704600 -1.56202900

O 0.99373500 0.09119700 -1.11579400

H 1.87513500 0.42475100 2.61120200

H 1.80153300 2.12023400 0.71394100

Cl -0.35993700 -1.30795500 2.97431400

H 4.72494000 1.12737200 0.77620200

C 5.46187500 -1.06989800 2.74735700

O 5.75172300 -0.20594600 1.65193300

C 6.65877800 -0.85557200 0.75225100

C 7.06135600 -2.17857800 1.41784200

C 6.70700800 -1.93608000 2.88861200

H 5.24684100 -0.44563600 3.61884500

H 4.57878800 -1.68181900 2.52126100

H 6.16354600 -1.01394800 -0.21237600

H 7.51702400 -0.18794200 0.61112300

H 6.45570500 -2.99620300 1.01572700

H 8.11473300 -2.42048700 1.26011200

H 6.52321900 -2.85735500 3.44593800

H 7.50085700 -1.37362000 3.39066000

C 4.52285200 2.75785700 -0.42896600

C 3.97385800 4.02768600 -0.63056600

C 5.38922500 2.22377600 -1.39005700

C 4.29080200 4.74563100 -1.77989900

H 3.28151500 4.44568000 0.09355600

C 5.69296900 2.94642500 -2.53714100

H 5.81388900 1.23587300 -1.23085500

C 5.14767500 4.21337700 -2.73889800

H 3.86010500 5.73218000 -1.92409500

H 6.36831900 2.52026300 -3.27283300

H 5.39121600 4.77934900 -3.63177500

N 4.16959600 1.98350600 0.69667200

C 4.13764800 2.65203200 1.99390500

H 3.98984000 1.88838400 2.76027700

H 5.06854000 3.19354000 2.19568200

H 3.29896300 3.35291200 2.04497800

**PhN-TS-b**

P -2.00864700 0.84419800 0.72049400

C -2.01094400 2.68366600 0.74386500

C -1.84492800 3.35207600 1.96169900

C -1.97624300 3.42398200 -0.44316500

C -1.67072400 4.73449100 1.99645800

H -1.85196900 2.79165200 2.89256400

C -1.80049300 4.80362800 -0.40727700

H -2.06875800 2.91709800 -1.39805600

C -1.64731200 5.46378100 0.81150800

H -1.55078300 5.23672600 2.95164300

H -1.77620900 5.36436700 -1.33694500

H -1.51075400 6.54088400 0.83535000

C -2.95326200 0.48926100 2.26290900

C -2.49583300 -0.48051900 3.15493000

C -4.13852000 1.18153000 2.54975900

C -3.22462600 -0.76596000 4.31297600

H -1.57396800 -1.01326300 2.94201300

C -4.85673600 0.90059700 3.70544900

H -4.49943400 1.94395400 1.86384700

C -4.40191500 -0.08063100 4.58924000

H -2.86142200 -1.52642600 4.99751700

H -5.77296100 1.44366800 3.91925300

H -4.96636000 -0.30362800 5.48970600

C -2.20788000 -1.87652100 -1.49827200

H -2.16051700 -2.24621500 -0.46323500

C -2.76398000 -3.00122600 -2.38202900

H -2.76430400 -2.73554100 -3.44090400

H -2.19135700 -3.92133400 -2.27796800

H -3.79928200 -3.21770000 -2.08993600

P -0.41950100 -1.31196900 -1.75335100

C -0.35087800 -0.39911500 -3.42263300

C -1.03015700 -1.08647200 -4.61585000

H -2.11808800 -1.07329400 -4.49725200

H -0.80123400 -0.51368000 -5.52454900

H -0.70923900 -2.11598300 -4.78313900

C -1.01119100 0.98648800 -3.26602200

H -2.08786900 0.91767000 -3.09222200

H -0.54351500 1.56506700 -2.46575600

H -0.86855600 1.52823500 -4.21164100

C 1.13137200 -0.13329400 -3.73435000

H 1.68533100 -1.04276300 -3.98363700

H 1.19276800 0.53567800 -4.60196700

H 1.62164100 0.37108600 -2.89207300

C 0.60656900 -2.92298700 -1.87333900

C 2.05014100 -2.55130600 -1.48230400

H 2.06972500 -2.14225000 -0.46603300

H 2.67320000 -3.45593600 -1.50776000

H 2.49345200 -1.81653900 -2.16150500

C 0.11577100 -3.91716800 -0.80486300

H 0.01138100 -3.44442200 0.17392800

H -0.82841400 -4.39617400 -1.07142600

H 0.86444100 -4.71555000 -0.71230900

C 0.62374400 -3.63785600 -3.22926100

H -0.37884800 -3.92102400 -3.56260900

H 1.08914000 -3.03880500 -4.01657300

H 1.21018500 -4.56117900 -3.13064600

Rh 0.27376800 0.16532700 0.26177300

C -6.51333300 -0.64673100 0.99766000

Fe -5.05124600 -0.66288700 -0.56275200

C -5.49401500 -1.57793900 1.32785700

C -7.11461400 -1.06455000 -0.21997900

C -6.46381000 -2.25257700 -0.64458500

C -5.46067500 -2.56769100 0.31223000

C -4.26928100 1.28130600 -1.04397000

C -4.86523100 0.71047400 -2.19030300

C -4.17203500 -0.49278200 -2.48318000

C -3.13762000 -0.67955300 -1.52137400

C -3.19088000 0.44368600 -0.62219400

H -4.83041800 -1.51925800 2.18011200

H -7.89509600 -0.54218500 -0.75659100

H -6.66197900 -2.79150400 -1.56158100

H -4.76798200 -3.39742700 0.26034700

H -4.57051400 2.20136600 -0.56027400

H -5.72784200 1.09287500 -2.71912400

H -4.42867500 -1.18426700 -3.27399600

H -6.75979800 0.24462400 1.55789200

C 4.38297600 2.31767300 -1.85438500

C 4.45730000 3.58263000 -2.42610100

C 3.52800500 4.55810700 -2.06908200

C 2.51966700 4.25208900 -1.16129700

C 2.44087200 2.98800800 -0.57986500

H 5.12921900 1.57651600 -2.12218200

H 5.23937300 3.80575300 -3.14496100

H 3.58484200 5.54956400 -2.50858200

H 1.76326000 4.99021800 -0.90461900

C 3.38888500 2.00938800 -0.91899500

C 3.35783300 0.69908700 -0.21766500

C 2.38439700 0.48458500 0.82342300

C 1.21791900 2.58067900 0.21021200

C 1.48421200 1.50801800 1.26803700

H 0.72107700 3.46784700 0.63681900

O 0.40250300 1.92712400 -0.72954700

H 2.57212400 -0.36886600 1.47011500

H 1.26210500 1.67508900 2.31889100

Cl 0.39637700 -1.77928400 1.89328500

H 3.64891200 -0.17311500 -0.80031900

N 5.00180500 0.68134400 0.80444800

H 4.62602700 0.13969100 1.59856100

C 3.60732300 -1.87360900 3.61524000

O 4.50021800 -1.51635700 2.54751700

C 4.48913200 -2.53686600 1.53903000

C 3.49512200 -3.60329200 2.00191200

C 3.45190900 -3.38645000 3.51754300

H 4.06290500 -1.53430800 4.54968000

H 2.64036800 -1.37855100 3.46964600

H 4.19596700 -2.09009300 0.57936500

H 5.51221900 -2.92033200 1.43716300

H 2.50533000 -3.40596700 1.58040200

H 3.81151900 -4.61012300 1.71841500

H 2.51508400 -3.73002500 3.96155300

H 4.28941900 -3.89147900 4.01104000

C 5.30948000 2.04507500 1.26760800

H 4.37546300 2.51038300 1.59443300

H 6.01924800 2.02294700 2.09970900

H 5.73143200 2.62076700 0.44224800

C 6.17331400 -0.06101000 0.36030100

C 6.31289700 -0.51485300 -0.94813400

C 7.14469300 -0.38335900 1.31158400

C 7.43492400 -1.26154700 -1.30755500

H 5.54725000 -0.31973100 -1.68855500

C 8.26142400 -1.12593200 0.94762200

H 7.00471700 -0.07502800 2.34325600

C 8.41282700 -1.56286900 -0.36714000

H 7.53365500 -1.61317800 -2.32940000

H 9.00912800 -1.37097500 1.69527500

H 9.28295500 -2.14524300 -0.65184700

**PhN-b1**

P -2.30492300 0.98199700 0.50103600

C -2.24080100 2.75826200 0.02605700

C -2.21949300 3.74330000 1.02026300

C -2.00122600 3.13630900 -1.29975600

C -1.98809700 5.07813000 0.69477600

H -2.38618900 3.46820800 2.05823100

C -1.76668000 4.46902000 -1.62359600

H -1.98384200 2.38177300 -2.07924200

C -1.75955100 5.44512600 -0.62848900

H -1.98392600 5.82927500 1.47932500

H -1.58019900 4.74282300 -2.65790100

H -1.57678600 6.48498700 -0.88348300

C -3.43841000 1.04293900 1.95064700

C -3.10850800 0.30455900 3.08848900

C -4.62264400 1.79243900 1.93532000

C -3.96506300 0.30334100 4.19295500

H -2.18045900 -0.26266400 3.10937400

C -5.46853700 1.79504900 3.03793600

H -4.88050900 2.37540000 1.05413500

C -5.14191200 1.04381500 4.16966100

H -3.70159700 -0.27690400 5.07205500

H -6.38402400 2.37970700 3.01832300

H -5.80496700 1.04311500 5.02976800

C -2.30616500 -2.20862700 -0.90243900

H -2.41449300 -2.27646100 0.18992500

C -2.77945700 -3.54044400 -1.50290200

H -2.62881700 -3.58939700 -2.58308300

H -2.26233700 -4.39199200 -1.06318400

H -3.85198000 -3.66696500 -1.30937000

P -0.47469000 -1.72743300 -1.05295200

C -0.17510500 -1.31286200 -2.88845500

C -0.72709700 -2.29956900 -3.92584000

H -1.82040900 -2.26079600 -3.94747100

H -0.37726200 -1.99399000 -4.92136100

H -0.41719000 -3.33408300 -3.76741800

C -0.80032000 0.06339000 -3.19271000

H -1.89239400 0.03449800 -3.17807900

H -0.43889800 0.82947700 -2.50172500

H -0.49746300 0.34950800 -4.21019300

C 1.34123800 -1.14132400 -3.07262800

H 1.89280100 -2.08092000 -2.97306900

H 1.53171300 -0.75283300 -4.08134600

H 1.72799400 -0.40639200 -2.35576100

C 0.48381800 -3.31695900 -0.59772200

C 1.89694400 -2.87970000 -0.16428300

H 1.84277800 -2.20404500 0.69741700

H 2.46954600 -3.77081600 0.12729300

H 2.44785200 -2.38264200 -0.96954500

C -0.16280300 -3.94199700 0.65237400

H -0.29334000 -3.20338900 1.44746400

H -1.12298500 -4.41950700 0.44722400

H 0.51093300 -4.72301300 1.02962100

C 0.61622400 -4.39358300 -1.68191100

H -0.35157700 -4.74556200 -2.04977300

H 1.20231900 -4.05098000 -2.53994000

H 1.14033500 -5.25870900 -1.25336600

Rh 0.10362500 0.23957600 0.53930700

C -6.86216600 -0.33503600 0.54827500

Fe -5.22295100 -0.79778300 -0.74233600

C -5.94774100 -1.16954900 1.24287900

C -7.33148300 -1.04604100 -0.58911900

C -6.70217800 -2.31860500 -0.60043200

C -5.84616300 -2.39310000 0.53234300

C -4.30833600 0.93786400 -1.61264000

C -4.76248500 0.07423000 -2.63453900

C -4.07964100 -1.16200100 -2.48966400

C -3.19124400 -1.07383700 -1.37911600

C -3.33001400 0.24931100 -0.83198700

H -5.39210900 -0.90254400 2.13166700

H -8.00804200 -0.66601300 -1.34267100

H -6.81478100 -3.07648100 -1.36450200

H -5.20244500 -3.22313800 0.79236400

H -4.63837800 1.95398700 -1.43918500

H -5.53179100 0.29310300 -3.36286500

H -4.25102200 -2.04264100 -3.09353200

H -7.12038200 0.67963000 0.81769500

C 4.57022700 2.03267200 -1.49455900

C 4.78871800 3.21092900 -2.20331500

C 3.83981900 4.22868500 -2.15049900

C 2.67069900 4.04709000 -1.41657200

C 2.44753400 2.87261700 -0.70068600

H 5.30246100 1.22904500 -1.57422200

H 5.68811200 3.32683100 -2.80072600

H 4.00155400 5.15150600 -2.69963100

H 1.89972100 4.81417700 -1.40697600

C 3.42044600 1.86541000 -0.72085300

C 3.21665200 0.60374100 0.09668100

C 2.08661600 0.62631400 1.08567400

C 1.11312200 2.56267500 -0.05496000

C 1.25952100 1.78370600 1.24869600

H 0.53383200 3.49161100 0.08381800

O 0.46617700 1.65386600 -0.90633400

H 2.25584400 0.00542600 1.96064000

H 0.99537900 2.20380700 2.21561100

Cl 0.05611300 -1.11667600 2.69306500

H 3.20479500 -0.28524700 -0.54461900

C 4.67380900 -1.00817200 1.37464900

C 4.23317400 -1.42456100 2.62444500

C 5.22021200 -1.91091400 0.46422200

C 4.34423000 -2.77286000 2.96069700

H 3.78777400 -0.73093100 3.32810800

C 5.33809000 -3.24974900 0.81576600

H 5.54920700 -1.56126800 -0.51188400

C 4.89835500 -3.68204600 2.06613800

H 3.97887900 -3.10687400 3.92539500

H 5.76006300 -3.95609300 0.10824500

H 4.97735200 -4.72968900 2.33688300

N 4.55037600 0.38210100 0.92325400

C 4.73058800 1.41000400 1.98440800

H 3.87963700 1.39051100 2.66164400

H 5.66267300 1.20169900 2.51173200

H 4.77719700 2.38068100 1.48937500

H 5.35252200 0.52637000 0.28798900

C 8.02435800 -0.06917400 1.06165100

O 7.20628600 0.52286200 0.04624800

C 7.85716800 0.42450100 -1.22895000

C 9.21447900 -0.23243300 -0.96515300

C 8.94079000 -1.02725600 0.31463400

H 8.59500100 0.71783200 1.57195900

H 7.36675900 -0.56258400 1.78356500

H 7.23502000 -0.19532900 -1.88753400

H 7.93493600 1.42549200 -1.66337600

H 9.54496600 -0.85103000 -1.80182600

H 9.97781200 0.52938800 -0.77902700

H 8.39947100 -1.95257100 0.08759500

H 9.84381700 -1.27760900 0.87474700

**PhN-b2**

P -1.69423500 1.23660600 -0.12512200

C -1.34574500 2.56953400 -1.35050000

C -0.31708500 3.45415900 -1.00080200

C -1.97791700 2.73270800 -2.58222700

C 0.06655400 4.47795600 -1.85513900

H 0.18596100 3.33152700 -0.04273700

C -1.58894200 3.75899800 -3.44629600

H -2.77448800 2.05792800 -2.87982800

C -0.56996700 4.63263500 -3.08755900

H 0.86384400 5.15577500 -1.56277000

H -2.08999800 3.87108600 -4.40333200

H -0.27186500 5.43137400 -3.76018800

C -2.21584500 2.30245100 1.29559200

C -1.86750900 1.92689700 2.59603700

C -2.94229000 3.48530000 1.10310400

C -2.27717300 2.69858100 3.68464200

H -1.28729000 1.02125400 2.75412400

C -3.35108200 4.25205300 2.18976800

H -3.16833000 3.82489000 0.09591400

C -3.02461300 3.85516700 3.48665200

H -2.00777800 2.38831300 4.68987000

H -3.91505000 5.16531600 2.02355600

H -3.34256500 4.45328300 4.33565900

C -2.79981300 -2.14602000 -0.69825700

H -2.68604400 -2.19119100 0.39546300

C -3.63029900 -3.36397400 -1.13106300

H -3.90349800 -3.32034300 -2.18745200

H -3.10140400 -4.30019400 -0.96971300

H -4.55753900 -3.40582000 -0.54649400

P -0.97501200 -2.11155700 -1.17351700

C -0.85645700 -1.88861000 -3.05186100

C -1.76030600 -2.78022500 -3.91211300

H -2.81305400 -2.52674000 -3.75265200

H -1.53890200 -2.58751200 -4.97058400

H -1.63039300 -3.84880100 -3.73563100

C -1.23995100 -0.43050800 -3.35942400

H -2.29049700 -0.23539400 -3.12495800

H -0.59972600 0.27957700 -2.82819000

H -1.10739400 -0.26024600 -4.43712300

C 0.62030900 -2.05036300 -3.45104300

H 0.97991400 -3.07879600 -3.35052600

H 0.73247200 -1.76465900 -4.50530700

H 1.25229200 -1.38248400 -2.85270400

C -0.33626600 -3.86172900 -0.71060000

C 1.16948800 -3.69182300 -0.43478700

H 1.29902600 -3.07273500 0.45883000

H 1.61693300 -4.68005700 -0.25732000

H 1.70357900 -3.21202400 -1.26251000

C -0.94431500 -4.32423800 0.62711100

H -0.85333300 -3.55069600 1.39385800

H -1.99260900 -4.62099900 0.55258300

H -0.38466500 -5.20774200 0.96327900

C -0.51882800 -4.97567800 -1.75048100

H -1.56666900 -5.15461800 -2.00397600

H 0.02411200 -4.77499500 -2.67773800

H -0.11927900 -5.90950800 -1.33272800

Rh 0.23596700 -0.23871300 0.09863800

C -5.10882500 0.76968000 2.40821100

Fe -4.91877100 -0.09744200 0.47371300

C -4.35039300 -0.43002600 2.49610700

C -6.39803500 0.43970800 1.91083800

C -6.43898000 -0.96287600 1.68595200

C -5.17301600 -1.49800700 2.05112200

C -4.41623000 1.29924300 -1.05041500

C -5.41943100 0.44081300 -1.54259400

C -4.90332800 -0.87984800 -1.51746300

C -3.57373200 -0.86405000 -0.99998900

C -3.26526600 0.51903100 -0.71359700

H -3.30555000 -0.51408500 2.77386000

H -7.19245400 1.14037300 1.69077900

H -7.26918900 -1.51674600 1.26847100

H -4.86709600 -2.53266400 1.96066200

H -4.50332800 2.36827600 -0.91351900

H -6.41883500 0.72692500 -1.84110900

H -5.46622600 -1.76124900 -1.78648500

H -4.74412900 1.76561800 2.62517900

C 4.50391800 -1.48928800 -1.98863500

C 4.91946800 -1.00351300 -3.22355300

C 4.54161800 0.28145700 -3.61445600

C 3.71943900 1.04023200 -2.78897300

C 3.30179600 0.56409600 -1.54210700

H 4.77011300 -2.50264400 -1.69280200

H 5.51791000 -1.62609200 -3.88090400

H 4.85560200 0.67078000 -4.57845900

H 3.34602600 2.00448600 -3.12741500

C 3.73403500 -0.70188400 -1.12418200

C 3.36670100 -1.20141000 0.24784100

C 2.21939100 -0.50999100 0.90223500

C 2.14204400 1.25838900 -0.84578300

C 1.92535500 0.86042500 0.61617200

H 2.22977700 2.35402100 -0.97422200

O 1.02856500 0.74339400 -1.51434100

H 2.03264100 -0.84846400 1.91744200

H 1.78295200 1.60960700 1.39297200

Cl -0.57816200 -1.24416500 2.28053300

H 3.28518800 -2.29308800 0.26043000

C 4.37979400 -1.14999800 2.60615200

C 4.59234700 -0.09293600 3.47951400

C 3.93322300 -2.38835200 3.05300600

C 4.34283900 -0.27883200 4.83711000

H 4.94632100 0.86076900 3.09737600

C 3.68099200 -2.56000500 4.40947600

H 3.74466200 -3.19702300 2.35251100

C 3.88600700 -1.50857900 5.30101600

H 4.49769700 0.54251700 5.52869800

H 3.30900300 -3.51371700 4.76739900

H 3.67929900 -1.64740100 6.35681700

N 4.64669200 -0.94082200 1.18057500

C 5.88367200 -1.66939200 0.78810500

H 6.66887700 -1.41958300 1.50368100

H 5.68785500 -2.74348300 0.81494800

H 6.17636300 -1.36562700 -0.21526800

H 4.85389800 0.07066000 1.03846000

C 6.52132800 1.53684200 -0.52340900

O 5.69621000 1.57447100 0.65235400

C 4.99135000 2.82566600 0.73278400

C 5.22002300 3.52123600 -0.60611500

C 6.58877200 2.97674800 -1.02361700

H 7.49631000 1.12967100 -0.23810500

H 6.05456500 0.88075800 -1.26895200

H 3.93755600 2.61496500 0.93920100

H 5.41386300 3.40219600 1.56503800

H 4.45631700 3.21201400 -1.32457500

H 5.19663100 4.60942500 -0.51633900

H 6.75986800 3.02884600 -2.10118700

H 7.39076700 3.52155500 -0.51513600

**PhN-b3**

P -2.13087800 -1.11929900 0.08864900

C -1.76248700 -2.32502200 1.42869100

C -1.62005200 -3.68428700 1.12405100

C -1.41591700 -1.88363400 2.71090300

C -1.16514400 -4.58516100 2.08461100

H -1.86460300 -4.04352400 0.12808500

C -0.95835200 -2.78357100 3.66851400

H -1.48681400 -0.82850900 2.95417300

C -0.83173600 -4.13738900 3.36004300

H -1.06835600 -5.63693700 1.83194200

H -0.69082500 -2.42291000 4.65735900

H -0.47474500 -4.83783100 4.10948900

C -3.28273100 -2.09640400 -0.96305000

C -3.11271700 -2.05375000 -2.34818000

C -4.32526400 -2.85900000 -0.41929400

C -3.98964600 -2.75513300 -3.18054100

H -2.29127100 -1.47978300 -2.77312100

C -5.19084300 -3.56091900 -1.24931000

H -4.45606700 -2.90025200 0.65997000

C -5.02572300 -3.50547600 -2.63541000

H -3.85158200 -2.71470600 -4.25674600

H -5.99657600 -4.14991100 -0.81982500

H -5.70410200 -4.05237400 -3.28373900

C -2.62595700 2.26275300 -0.44382100

H -2.78903500 1.69623800 -1.37255200

C -3.30118900 3.63282600 -0.60648500

H -3.11782400 4.29277900 0.24381500

H -2.96269800 4.15210700 -1.50131800

H -4.38586100 3.49386200 -0.69273400

P -0.73360000 2.18170600 -0.30432400

C -0.29386800 2.89629200 1.40670900

C -0.98298900 4.20218200 1.82043300

H -2.04735800 4.03184100 2.00776700

H -0.54488100 4.54834000 2.76672800

H -0.88048900 5.00828700 1.09144900

C -0.63089000 1.83698700 2.47497200

H -1.70831700 1.68940200 2.58383400

H -0.14545000 0.88040000 2.25858200

H -0.25380800 2.20348700 3.44059600

C 1.23246900 3.06893700 1.44757600

H 1.59106900 3.86899100 0.79241100

H 1.53403500 3.31895600 2.47362700

H 1.71874600 2.12321100 1.17722100

C -0.11720400 3.36647800 -1.66676700

C 1.33047500 2.94381600 -1.96987400

H 1.34457200 1.90253900 -2.30894600

H 1.73512900 3.58323400 -2.76735700

H 1.98335500 3.04270100 -1.09502700

C -0.90893800 3.07800700 -2.95618100

H -0.91334900 2.00878800 -3.18788200

H -1.93798000 3.44041400 -2.92208600

H -0.41182800 3.59601900 -3.78695200

C -0.14362300 4.87058000 -1.37198300

H -1.14200100 5.23528100 -1.11729700

H 0.53500000 5.14496800 -0.55838200

H 0.18728400 5.41089300 -2.26919700

Rh 0.20552500 -0.31299700 -0.66024700

C -6.83938600 -0.61868800 -0.09438600

Fe -5.26281400 0.67318600 0.54334500

C -6.10428400 -0.19094100 -1.23127000

C -7.38667100 0.53189100 0.53516300

C -6.98396600 1.67175000 -0.20926900

C -6.18990500 1.22296500 -1.30020300

C -4.04080300 -0.18061000 2.08735800

C -4.59251600 1.04097700 2.53607100

C -4.13093400 2.06769900 1.67028700

C -3.28388100 1.49029100 0.68035400

C -3.22031600 0.07959300 0.94730800

H -5.53581200 -0.82590000 -1.89706700

H -7.96478100 0.54317200 1.44927900

H -7.20052300 2.70235800 0.03924900

H -5.70458800 1.85037400 -2.03633200

H -4.20359800 -1.15622800 2.52705300

H -5.28807300 1.16540300 3.35507700

H -4.42450200 3.10738400 1.72333800

H -6.92830300 -1.63911000 0.25131400

C 5.09723800 -0.86401500 1.41525700

C 5.47735900 -1.56275700 2.56022700

C 4.55816400 -2.37754200 3.21320900

C 3.26241800 -2.48545800 2.71578500

C 2.87471800 -1.79216200 1.57244000

H 5.84458000 -0.24593000 0.92243600

H 6.49403300 -1.47321600 2.93166400

H 4.84985900 -2.92484800 4.10449500

H 2.52524000 -3.10837600 3.21720600

C 3.79907200 -0.97260500 0.91045100

C 3.30500600 -0.20730700 -0.31242300

C 2.18209900 -0.88811900 -1.08456600

C 1.45495200 -1.82772700 1.05011700

C 1.43467800 -1.93831200 -0.47088300

H 0.90947200 -2.65630400 1.52860700

O 0.85755700 -0.58095700 1.28212300

H 2.32054000 -0.95245100 -2.15972000

H 1.15070000 -2.84878400 -0.99255600

Cl -0.11883600 -0.35968500 -3.15868100

H 2.98834900 0.79644600 -0.00084400

C 5.35580400 -0.99469700 -1.67032200

C 4.89898500 -2.30262300 -1.76174800

C 6.66924700 -0.66198500 -1.99135800

C 5.78464600 -3.28613200 -2.19894400

H 3.88279800 -2.55602300 -1.48355900

C 7.54219100 -1.65290200 -2.42532700

H 7.00717400 0.36876800 -1.90179600

C 7.09733800 -2.96812400 -2.53338000

H 5.43695000 -4.31109500 -2.27150600

H 8.56629800 -1.39663600 -2.67551300

H 7.77533800 -3.74435800 -2.87247600

N 4.49575200 0.11950400 -1.23030200

C 4.09021500 0.99086300 -2.37983500

H 3.64289100 1.89369600 -1.96568400

H 4.98664700 1.23910500 -2.94805600

H 3.37076800 0.47050600 -3.01030200

H 5.08697200 0.74771400 -0.64909900

C 7.09070300 2.29633300 0.50528500

O 5.68558700 2.14478400 0.25588700

C 4.92972200 2.48840800 1.43781000

C 5.95187300 2.54476100 2.56524300

C 7.19761000 3.04460400 1.83080600

H 7.54969400 1.30020000 0.57680800

H 7.53414700 2.83068900 -0.33970900

H 4.44720500 3.45792100 1.26739100

H 4.16289500 1.72574400 1.59648200

H 5.63578200 3.19627400 3.38216700

H 6.11588300 1.53775700 2.96480100

H 7.13397200 4.12451400 1.66332600

H 8.13084600 2.82942400 2.35531400

**PhN-b4**

P -1.95077900 1.15641300 0.35087700

C -2.16035900 2.91483400 -0.18012400

C -1.64558700 3.89691500 0.67810200

C -2.71661100 3.32254600 -1.39516200

C -1.68956200 5.24346600 0.33493300

H -1.21113400 3.60050500 1.63070100

C -2.75944200 4.67261900 -1.74044500

H -3.12922500 2.59048100 -2.08000800

C -2.24773400 5.63731600 -0.87964900

H -1.28993900 5.98558800 1.01989100

H -3.19868500 4.96720900 -2.68906600

H -2.28503000 6.68827900 -1.14999000

C -2.56706700 1.33164100 2.08504800

C -1.75808500 0.90836100 3.14061200

C -3.81358700 1.90296900 2.37192900

C -2.20791300 1.01478500 4.45913700

H -0.79289100 0.46059600 2.92735900

C -4.26062700 2.00848200 3.68351200

H -4.43936300 2.27260200 1.56491800

C -3.45952200 1.55374100 4.73258100

H -1.57469800 0.66571200 5.26928700

H -5.23200300 2.44924500 3.88978000

H -3.81147700 1.62978300 5.75716000

C -2.06294300 -2.01837300 -1.17939700

H -1.79764600 -2.20168800 -0.12793300

C -2.55454300 -3.34173400 -1.78088500

H -2.78574600 -3.25731600 -2.84642500

H -1.81965700 -4.13607600 -1.66601200

H -3.46525000 -3.66151600 -1.25940900

P -0.42178300 -1.29834600 -1.80009200

C -0.77084300 -0.50154700 -3.49345800

C -1.67999000 -1.30181700 -4.43357900

H -2.68482300 -1.39808300 -4.01004900

H -1.77742600 -0.75637700 -5.38209900

H -1.30249400 -2.30048100 -4.66264400

C -1.44828200 0.85545100 -3.23514900

H -2.46293300 0.72359500 -2.85486500

H -0.86340700 1.47718200 -2.54651500

H -1.52604800 1.38930100 -4.19260100

C 0.56405400 -0.17655500 -4.18061200

H 1.09309500 -1.06516600 -4.53536300

H 0.35988100 0.45022900 -5.05783300

H 1.20983100 0.40006300 -3.50595500

C 0.72461200 -2.80407000 -2.08677900

C 2.16026600 -2.25330100 -2.15917300

H 2.38131600 -1.75121500 -1.21289200

H 2.85817300 -3.09273400 -2.29239600

H 2.32066200 -1.55442800 -2.98271300

C 0.71954000 -3.70125400 -0.83439500

H 1.02995400 -3.13830100 0.04990200

H -0.24865700 -4.15301400 -0.61128900

H 1.43225000 -4.52152600 -1.00627100

C 0.46603800 -3.65801400 -3.33534900

H -0.54697800 -4.06529200 -3.37101800

H 0.63928900 -3.10139900 -4.26022800

H 1.16265700 -4.50784200 -3.33340400

Rh 0.37636300 0.31997900 0.01729700

C -5.88122100 -0.85061300 2.04973900

Fe -4.79452200 -1.04118300 0.22331600

C -4.59775300 -1.39184200 2.32548200

C -6.55993700 -1.74658100 1.17917200

C -5.69241700 -2.83934100 0.91375000

C -4.47850100 -2.61731000 1.62020400

C -4.59221200 0.81212100 -0.82049000

C -5.28440800 -0.10250200 -1.64152900

C -4.42531400 -1.20680900 -1.87277300

C -3.18921500 -0.99383800 -1.19609100

C -3.29483600 0.28403300 -0.53085800

H -3.82323900 -0.92721500 2.92168700

H -7.54347000 -1.59965600 0.75352100

H -5.90049600 -3.66747100 0.24904800

H -3.59534500 -3.24255000 1.59333900

H -4.97617900 1.75915600 -0.46685800

H -6.30299900 -0.00485400 -1.99195500

H -4.69644600 -2.09382800 -2.42770000

H -6.25560600 0.09782200 2.40969600

C 5.15396400 2.48509000 -1.19416500

C 5.47454500 3.82539800 -1.41038200

C 4.50185600 4.80580700 -1.25073100

C 3.21215300 4.44309600 -0.86624400

C 2.88134600 3.11057600 -0.64348900

H 5.93776700 1.74044500 -1.29844900

H 6.48518100 4.09642200 -1.69952200

H 4.74689000 5.84963200 -1.42203100

H 2.44386700 5.20128200 -0.73712600

C 3.86463000 2.12067100 -0.81446800

C 3.37981000 0.70027000 -0.59175700

C 2.39502100 0.54515900 0.56420700

C 1.49595800 2.66285800 -0.21169100

C 1.61386400 1.67545400 0.95297200

H 0.89413300 3.54513600 0.05772500

O 0.88995600 1.88975600 -1.21370900

H 2.70595400 -0.15447300 1.33638100

H 1.43514600 1.95708000 1.98753100

Cl 0.11490400 -1.51744000 1.76194500

H 2.89989800 0.37558000 -1.51906300

C 5.41384800 -0.15563600 0.75220600

C 5.28892100 0.89809800 1.65040400

C 6.37287500 -1.14898600 0.94334300

C 6.16317600 0.96239300 2.73429800

H 4.52427300 1.65390300 1.52370800

C 7.23805600 -1.07183400 2.02885300

H 6.44056200 -1.98891000 0.25910000

C 7.13650700 -0.01212100 2.92628100

H 6.07043200 1.78608600 3.43397100

H 7.98523100 -1.84505400 2.17317000

H 7.80899200 0.04865500 3.77550700

N 4.48029200 -0.34113400 -0.38526400

C 5.17599000 -0.73855400 -1.65576500

H 6.17864700 -0.31580800 -1.67966500

H 5.23210100 -1.82803300 -1.69838900

H 4.59199600 -0.37410100 -2.50018300

H 3.94778000 -1.19160600 -0.08403300

C 3.41409100 -2.68899400 2.20265000

O 3.64860900 -2.69582500 0.78838300

C 4.08074500 -4.00967600 0.45228300

C 3.33356300 -4.96454300 1.40262400

C 2.72587000 -4.02428100 2.46647000

H 4.38107500 -2.60025300 2.72182000

H 2.76946400 -1.84015200 2.43253700

H 3.85281600 -4.17196700 -0.60423500

H 5.16954700 -4.08355500 0.60013300

H 2.55300400 -5.51822000 0.87656700

H 4.02407400 -5.69067700 1.83869000

H 1.65217100 -3.89352000 2.30917300

H 2.89020600 -4.37765300 3.48669100

**PhN-b5**

P -2.16180800 0.93908500 0.49990400

C -2.19870300 2.74234200 0.13697700

C -2.28179700 3.65783400 1.19385100

C -1.93163900 3.22377900 -1.14929600

C -2.12126400 5.02265900 0.96724900

H -2.47086300 3.30283400 2.20341700

C -1.76660400 4.58743000 -1.37370800

H -1.83701000 2.52800300 -1.97614000

C -1.86087800 5.49192600 -0.31770800

H -2.19588500 5.71766000 1.79835600

H -1.55576700 4.94250100 -2.37809900

H -1.73269100 6.55559300 -0.49604000

C -3.36560300 0.83198300 1.88923700

C -3.04335800 0.04686800 2.99807100

C -4.59594100 1.50158600 1.85419400

C -3.95195800 -0.07805200 4.05289000

H -2.08130300 -0.45928100 3.03810400

C -5.49432700 1.38072800 2.90741800

H -4.84782700 2.12149500 0.99670500

C -5.17398400 0.58415400 4.00933700

H -3.69351400 -0.69155000 4.91063700

H -6.44535100 1.90489200 2.87218200

H -5.87809800 0.48725000 4.83043300

C -1.85167200 -2.13495100 -1.08785900

H -1.98448800 -2.27078800 -0.00450100

C -2.19688600 -3.46148600 -1.78037600

H -1.99486800 -3.43615100 -2.85308000

H -1.64000400 -4.29944200 -1.36389000

H -3.26483300 -3.67325800 -1.64525500

P -0.06722000 -1.49085800 -1.15435900

C 0.23144000 -0.92757900 -2.95214900

C -0.22027700 -1.88002500 -4.06609200

H -1.31206200 -1.93117900 -4.11014200

H 0.11910700 -1.47770500 -5.03025900

H 0.17493600 -2.89284900 -3.97040700

C -0.48544400 0.42007000 -3.17499500

H -1.57275000 0.31747000 -3.18911100

H -0.18831700 1.15530400 -2.42084400

H -0.18145900 0.79947500 -4.16099500

C 1.73044600 -0.61869000 -3.09345300

H 2.37385400 -1.49773000 -2.99030200

H 1.90884000 -0.19353500 -4.08979000

H 2.01212300 0.14243200 -2.35591900

C 1.02162500 -3.01611800 -0.78463800

C 2.37687500 -2.48560000 -0.27527200

H 2.22858700 -1.88548400 0.63057400

H 3.01437200 -3.34611000 -0.02901700

H 2.91044000 -1.88216800 -1.01902400

C 0.40060500 -3.77223300 0.40362400

H 0.17031200 -3.09632800 1.23274100

H -0.50254800 -4.32335500 0.13634100

H 1.13123900 -4.51195100 0.76152800

C 1.27071900 -4.00282600 -1.93039900

H 0.34149100 -4.40953300 -2.33918700

H 1.83589200 -3.55350900 -2.75205400

H 1.86111900 -4.84724000 -1.54892300

Rh 0.33249700 0.39613500 0.63006700

C -6.60606700 -0.71601600 0.25225600

Fe -4.88053700 -0.96919100 -0.98550500

C -5.66034900 -1.52432600 0.93522900

C -6.97050000 -1.38213200 -0.94861300

C -6.24548300 -2.60138900 -1.01065000

C -5.43414900 -2.68691400 0.15403600

C -4.06952700 0.88206600 -1.70669500

C -4.40902800 0.05031900 -2.79800200

C -3.63800200 -1.13696800 -2.69238100

C -2.80856600 -1.04896200 -1.53655300

C -3.07442800 0.22414000 -0.92149200

H -5.16456400 -1.27475800 1.86385900

H -7.64276000 -1.00439500 -1.70717300

H -6.26809400 -3.31308400 -1.82527900

H -4.73639200 -3.48044300 0.38782200

H -4.48816600 1.85667000 -1.49090900

H -5.16296200 0.25278000 -3.54675900

H -3.71458100 -1.99001900 -3.35328200

H -6.95608000 0.25450500 0.57493000

C 4.80514000 3.03169500 -0.67214100

C 4.95467100 4.37043600 -1.03572000

C 3.86344400 5.22973700 -0.99032200

C 2.62356900 4.74417200 -0.58025300

C 2.46663300 3.41380100 -0.20767200

H 5.67489300 2.38378000 -0.73101900

H 5.92568200 4.73215100 -1.35937700

H 3.97580600 6.27074400 -1.27809800

H 1.75454700 5.39719800 -0.55133900

C 3.56841900 2.54214800 -0.24834400

C 3.29265300 1.09949500 0.15436900

C 2.24788000 0.95790200 1.26196200

C 1.13169000 2.83643900 0.21052200

C 1.29530500 1.99677200 1.47656900

H 0.39529500 3.64511900 0.34440300

O 0.72018000 1.88837500 -0.73986500

H 2.56735600 0.36983800 2.12210600

H 0.98342100 2.33482200 2.45996800

Cl 0.27537900 -1.09405000 2.67915400

H 2.97310800 0.52062300 -0.71156400

N 4.53717400 0.36713800 0.66082800

H 4.16298200 -0.48678000 1.12918200

C 3.65653400 -2.03860400 3.49615300

O 4.44306000 -1.86795200 2.30681500

C 4.97470200 -3.15027800 1.95843600

C 4.01783800 -4.20735200 2.54664900

C 2.95975900 -3.37537700 3.29334400

H 4.32738500 -2.04893700 4.36758000

H 2.95253900 -1.20697800 3.56790300

H 5.05477200 -3.18744000 0.86940400

H 5.98278800 -3.24646800 2.38537300

H 3.56799000 -4.82635200 1.76595600

H 4.55887300 -4.87148900 3.22596700

H 2.06826700 -3.21031000 2.68347800

H 2.64863800 -3.82814400 4.23716100

C 5.22640200 1.11128800 1.77000500

H 4.47726600 1.72478500 2.27127700

H 5.63357800 0.37917000 2.46593000

H 6.01163100 1.73978700 1.35624800

C 5.47038800 -0.17197700 -0.35376900

C 5.25015900 -0.00186300 -1.71717100

C 6.55113300 -0.92077500 0.10685600

C 6.12774300 -0.59477400 -2.62284500

H 4.42760600 0.60130000 -2.08334100

C 7.41925900 -1.50706400 -0.80839400

H 6.70403900 -1.06310400 1.17163800

C 7.20806500 -1.34764500 -2.17527400

H 5.95748500 -0.45828500 -3.68550000

H 8.25880800 -2.09256700 -0.44827600

H 7.88565300 -1.80526700 -2.88839200

**PhN-b6**

P -2.02294500 -1.20564300 -0.06369300

C -1.64641200 -2.57728500 1.10499000

C -1.46891800 -3.87836100 0.61761700

C -1.33022600 -2.31246800 2.44193800

C -1.00266400 -4.89307500 1.45050200

H -1.69658300 -4.10219500 -0.42095800

C -0.85955900 -3.32551200 3.27186100

H -1.44263800 -1.30692600 2.83271400

C -0.69344900 -4.61923400 2.78043000

H -0.87970100 -5.89720500 1.05546000

H -0.61729400 -3.10014100 4.30623800

H -0.32915500 -5.40865800 3.43143600

C -3.13446000 -2.06598100 -1.25403400

C -2.92847800 -1.88194400 -2.62243200

C -4.18512900 -2.88633200 -0.82060200

C -3.77665100 -2.50085900 -3.54560500

H -2.10636500 -1.25627100 -2.96336000

C -5.02232500 -3.50519300 -1.74059200

H -4.34539200 -3.04143500 0.24381400

C -4.82073200 -3.30856300 -3.10892500

H -3.61150400 -2.34803300 -4.60785200

H -5.83403500 -4.13944900 -1.39538500

H -5.47842800 -3.78794500 -3.82812000

C -2.52538100 2.23653200 -0.08448200

H -2.61810700 1.82485700 -1.10025800

C -3.21370200 3.60831300 -0.06268800

H -3.08366500 4.12288100 0.89180100

H -2.83615400 4.26701700 -0.84287300

H -4.29023900 3.47811500 -0.22940000

P -0.64561400 2.14945100 0.15739900

C -0.31696600 2.54114800 1.99446200

C -1.06697700 3.73456900 2.59896500

H -2.13820600 3.52284100 2.66679100

H -0.70677200 3.89159700 3.62495500

H -0.92787700 4.66942400 2.05212600

C -0.68081000 1.29479600 2.82462100

H -1.75738700 1.10843000 2.84109200

H -0.15481400 0.40788300 2.45797700

H -0.36698300 1.47755600 3.86234700

C 1.19853400 2.72905800 2.16478000

H 1.58245000 3.63526200 1.68791200

H 1.42759700 2.79753000 3.23662700

H 1.72875800 1.85382300 1.76994900

C 0.06052800 3.56330900 -0.91503300

C 1.54031400 3.21671800 -1.16806700

H 1.61367600 2.21827600 -1.61696600

H 1.96968200 3.95204800 -1.86319200

H 2.14172700 3.24048200 -0.25435700

C -0.62474900 3.51887100 -2.29274600

H -0.58173100 2.51643700 -2.72765400

H -1.66582200 3.84685300 -2.26810600

H -0.08840500 4.20273300 -2.96433100

C -0.02196000 4.98793400 -0.35395200

H -1.04762700 5.29461200 -0.13117100

H 0.57545700 5.11273900 0.55392800

H 0.37506500 5.68515900 -1.10405300

Rh 0.25036900 -0.21367000 -0.61416700

C -6.76310100 -0.65257400 -0.39035500

Fe -5.18255800 0.49040700 0.48686000

C -5.93786600 -0.13773500 -1.42366300

C -7.30546100 0.44671500 0.32942900

C -6.80902700 1.64095500 -0.25669000

C -5.96110900 1.27810500 -1.33846600

C -4.03609500 -0.58952300 1.95178000

C -4.63008500 0.54361100 2.55158100

C -4.13703500 1.69460400 1.88274100

C -3.23011300 1.28477700 0.86265800

C -3.16191800 -0.15182200 0.91098400

H -5.35797300 -0.72254800 -2.12473100

H -7.94157400 0.38656000 1.20227700

H -6.99972500 2.64701200 0.09343900

H -5.39816200 1.95792900 -1.96481600

H -4.20700000 -1.62210700 2.22689400

H -5.36734400 0.53544700 3.34322100

H -4.44872500 2.71135100 2.07965100

H -6.91922000 -1.69989700 -0.17109800

C 5.18746400 -0.85221300 1.31360600

C 5.65182200 -1.78025500 2.24740500

C 4.81147400 -2.79461700 2.69132000

C 3.50928400 -2.86878500 2.19926500

C 3.03942200 -1.94537800 1.26942800

H 5.87661500 -0.06712200 1.00596600

H 6.66567700 -1.69651100 2.62792800

H 5.16438100 -3.51621200 3.42190400

H 2.82917700 -3.64209800 2.54847700

C 3.88704600 -0.92275100 0.80899900

C 3.28476200 0.07275400 -0.18679800

C 2.25392200 -0.54878400 -1.12451000

C 1.61295400 -1.94761300 0.76552700

C 1.59239800 -1.75897200 -0.74868400

H 1.10677200 -2.87653400 1.07563700

O 0.96378400 -0.79732000 1.23594500

H 2.35327900 -0.35603800 -2.18348400

H 1.38193300 -2.56474100 -1.44636300

Cl -0.10104400 0.14939500 -3.08387700

H 2.81211500 0.88458600 0.36519300

N 4.45693100 0.79987600 -0.94964300

C 4.72505900 2.14633200 -0.37348100

C 4.65230100 2.31682000 1.01024700

C 4.99785500 3.23801900 -1.19380900

C 4.85446500 3.57657500 1.56357600

H 4.41293300 1.48690200 1.66452400

C 5.19766300 4.49513100 -0.62674100

H 5.03211100 3.14988800 -2.27084800

C 5.12586400 4.67169000 0.74911800

H 4.78054100 3.69555900 2.63946900

H 5.39807600 5.33918500 -1.27819400

H 5.27129400 5.65497500 1.18362100

H 5.28228900 0.18444100 -0.81111900

C 4.34657900 0.82682700 -2.44009200

H 4.22451400 -0.19151500 -2.79349500

H 3.49574800 1.43887500 -2.74015000

H 5.28439300 1.21642400 -2.83266600

C 5.35649500 -2.40325300 -1.84176700

O 6.22072500 -1.30262200 -1.51607200

C 7.44403300 -1.79766500 -0.95462500

C 7.31532600 -3.32490100 -0.89040800

C 5.79974500 -3.54187900 -0.93741900

H 4.31941000 -2.09913700 -1.66542200

H 5.48486300 -2.65308100 -2.90399800

H 8.27900400 -1.46811100 -1.58098100

H 7.55980000 -1.36339100 0.04411400

H 7.79250300 -3.78568100 -1.76101000

H 7.77752900 -3.73614400 0.00981600

H 5.51963200 -4.52026400 -1.33382600

H 5.35337000 -3.42201000 0.05396500

**S-TS-a**

P 1.51726000 -1.15494200 0.10053400

C 0.97734900 -2.51152500 -1.01830400

C 0.71131800 -3.78219200 -0.49386800

C 0.61418200 -2.23956200 -2.34345700

C 0.09506800 -4.76005600 -1.27405600

H 0.97678500 -4.01025100 0.53488200

C 0.00707100 -3.21934400 -3.12330700

H 0.78805500 -1.25130400 -2.75621000

C -0.26258400 -4.47969200 -2.59026900

H -0.09953900 -5.74077600 -0.85009700

H -0.26273600 -2.99342700 -4.15086800

H -0.73712300 -5.24180100 -3.20160400

C 2.59259000 -2.07762400 1.27460200

C 2.48269100 -1.82393300 2.64266400

C 3.52752100 -3.01915300 0.82096100

C 3.30917900 -2.49791700 3.54613800

H 1.74841500 -1.10657800 3.00024800

C 4.34237100 -3.69232100 1.72254900

H 3.61413700 -3.22428200 -0.24379900

C 4.23627600 -3.42857200 3.09029900

H 3.21793100 -2.29279600 4.60836300

H 5.06326900 -4.42055400 1.36201200

H 4.87586000 -3.95260400 3.79427800

C 2.34567800 2.20167500 0.14934000

H 2.44448300 1.75038000 1.14756600

C 3.17376400 3.49575400 0.13236200

H 3.06789200 4.04615700 -0.80370200

H 2.89718600 4.17009200 0.94029200

H 4.23476000 3.24649000 0.25453000

P 0.45991100 2.33746500 -0.00830000

C 0.09949400 2.90936000 -1.79351500

C 0.92717500 4.09468900 -2.31230600

H 1.95835700 3.78563300 -2.50875200

H 0.50270100 4.41201700 -3.27421400

H 0.94156500 4.96356700 -1.65498900

C 0.34909700 1.74797300 -2.77665000

H 1.40333000 1.46669500 -2.83021300

H -0.25466500 0.87655200 -2.52040800

H 0.05520300 2.09832100 -3.77621000

C -1.40135000 3.23636700 -1.86855700

H -1.66886200 4.14120200 -1.31604500

H -1.67115000 3.40123000 -2.91958000

H -2.00672700 2.40198000 -1.49647200

C -0.02792500 3.73321300 1.20366000

C -1.51097200 3.51772100 1.55903300

H -1.63001700 2.58916700 2.12360700

H -1.84505000 4.35216700 2.18976400

H -2.15887500 3.47611500 0.67905100

C 0.76134500 3.57625200 2.51685200

H 0.72714200 2.55231000 2.89264900

H 1.80276000 3.89260900 2.43199700

H 0.29069800 4.22094200 3.27036600

C 0.15083700 5.17427900 0.70665100

H 1.18191900 5.40087100 0.42224600

H -0.50063500 5.41327800 -0.13784500

H -0.11837700 5.85167800 1.52738900

Rh -0.63400700 0.11628400 0.65639700

C 6.25798200 -1.12643100 0.14003400

Fe 4.78781700 0.21753600 -0.63180100

C 5.60736400 -0.48240400 1.22509700

C 6.88748100 -0.12813500 -0.65126600

C 6.62047300 1.13363300 -0.05821700

C 5.82772200 0.91296800 1.10095600

C 3.44288000 -0.70050400 -2.03482700

C 4.10993700 0.38348900 -2.64879000

C 3.78161500 1.56030000 -1.92515900

C 2.89965900 1.21766700 -0.86028600

C 2.68190000 -0.20084800 -0.93442900

H 5.01531800 -0.96593600 1.98990300

H 7.42830000 -0.29429500 -1.57319600

H 6.92588900 2.09598900 -0.44740600

H 5.43406700 1.67483700 1.76075500

H 3.49297400 -1.73849400 -2.33771100

H 4.79341500 0.32189600 -3.48482000

H 4.18137400 2.54572100 -2.12172000

H 6.24379700 -2.18773500 -0.06517400

C -4.38893600 1.81861900 0.20930300

C -5.13791400 2.27805700 -0.87385300

C -5.06722900 1.62495300 -2.10342700

C -4.23449800 0.51191600 -2.24352000

C -3.48802200 0.04759800 -1.16621200

H -4.43061600 2.33902600 1.16437600

H -5.77385200 3.15149900 -0.75782000

H -5.64182300 1.98716800 -2.95024800

H -4.13924300 0.01870200 -3.20963800

C -3.55143100 0.70567400 0.07760900

C -2.69753000 0.21496500 1.17043200

C -2.25741000 -1.16349500 1.17759700

H -2.82522100 0.69412400 2.13803400

C -2.39235100 -0.98617400 -1.29632100

C -2.32014100 -1.89072200 -0.06157100

H -2.52037600 -1.58774800 -2.21319600

O -1.17435100 -0.33057500 -1.30145300

H -2.06339000 -1.67921800 2.11232900

H -1.66080700 -2.75037700 -0.17153300

Cl -0.22286100 0.33478900 3.09068500

C -4.89479400 -3.10654300 -1.71376700

H -4.24480300 -3.68736400 -2.36991500

H -5.87678700 -3.58043200 -1.66239000

H -4.98082500 -2.08305600 -2.08155800

H -5.00652500 -2.24693100 0.53639100

C -6.31731200 -0.48621600 2.16117600

O -6.55232600 -1.41736400 1.10030600

C -7.01115100 -0.67706200 -0.02864700

C -7.92760600 0.38927700 0.56105200

C -7.24131400 0.72282400 1.89964900

H -6.53546500 -1.00678200 3.09618700

H -5.26135400 -0.18717600 2.15966000

H -6.16172200 -0.22219700 -0.55394400

H -7.51967700 -1.37881000 -0.69614800

H -8.01975100 1.25808000 -0.09501400

H -8.92401600 -0.02777800 0.73116500

H -6.65459500 1.64011700 1.81837000

H -7.96912800 0.85947000 2.70213700

S -4.14309700 -3.12154000 -0.05898500

**S-b1**

P -1.88507200 1.07271900 0.17048000

C -1.64932100 2.61953700 -0.79810500

C -1.77296600 3.86073800 -0.16270900

C -1.15336800 2.57908800 -2.10639900

C -1.43211100 5.03731400 -0.82684100

H -2.14001700 3.91118800 0.85877900

C -0.81037200 3.75442300 -2.76746000

H -1.02288400 1.62365100 -2.60388000

C -0.94857400 4.98743000 -2.13127700

H -1.54311200 5.99175700 -0.32095800

H -0.42748400 3.70491200 -3.78256800

H -0.68211000 5.90303100 -2.65105500

C -3.26341500 1.55810500 1.28819500

C -3.18097600 1.21616000 2.63910700

C -4.39059200 2.24605000 0.81852900

C -4.22677200 1.54404500 3.50686000

H -2.29771700 0.70094600 3.01118100

C -5.42440600 2.57804400 1.68547700

H -4.45473300 2.52257700 -0.23157800

C -5.34535800 2.22075900 3.03367900

H -4.15618000 1.27120800 4.55545100

H -6.29451600 3.11246600 1.31426200

H -6.15576600 2.47677500 3.70984200

C -1.79058600 -2.39238900 -0.18763700

H -2.10665100 -2.12606900 0.83183000

C -2.20834700 -3.84662200 -0.44795900

H -1.86111000 -4.21166400 -1.41654400

H -1.82544200 -4.52596500 0.31166200

H -3.30293700 -3.91938700 -0.43564200

P 0.05592000 -1.96880700 -0.10698300

C 0.73618500 -2.13490100 -1.88089900

C 0.35190000 -3.39660500 -2.66469000

H -0.71082700 -3.37464500 -2.92475400

H 0.91129400 -3.40713900 -3.61002000

H 0.56368800 -4.33058200 -2.14167400

C 0.26082800 -0.92342700 -2.70836300

H -0.81818100 -0.93653400 -2.87887100

H 0.55192100 0.02043300 -2.23924200

H 0.74611200 -0.98272100 -3.69314100

C 2.26556300 -2.00535200 -1.78837700

H 2.74047000 -2.86551700 -1.30489800

H 2.67690300 -1.92674500 -2.80353800

H 2.52726900 -1.08405300 -1.25107400

C 0.81537600 -3.34026700 0.98738900

C 2.12722900 -2.76251600 1.54978800

H 1.91378200 -1.85143200 2.11937900

H 2.58652200 -3.50261300 2.22105200

H 2.85210200 -2.52711300 0.76155100

C -0.09791000 -3.55900200 2.20770800

H -0.35072500 -2.61193200 2.69210100

H -1.01755200 -4.09411400 1.96412400

H 0.44700200 -4.17230600 2.93773900

C 1.10977400 -4.69318800 0.33012800

H 0.21810500 -5.15173400 -0.10543100

H 1.86996500 -4.61896300 -0.45319200

H 1.49353100 -5.38088200 1.09566100

Rh 0.44498200 0.43310800 0.92812000

C -6.43832800 -0.22233800 -0.27578400

Fe -4.60418200 -1.02779400 -1.02168100

C -5.70858800 -0.78751500 0.80308300

C -6.71278000 -1.25556500 -1.21186700

C -6.14699400 -2.45882700 -0.71396500

C -5.52533300 -2.16732200 0.53090900

C -3.45935800 0.37505900 -2.17650900

C -3.75831500 -0.76026200 -2.96277600

C -3.17626700 -1.88957300 -2.32955700

C -2.50772800 -1.46284700 -1.14549800

C -2.67868300 -0.03736900 -1.05383400

H -5.32283900 -0.25246300 1.66072800

H -7.21554000 -1.13673800 -2.16222200

H -6.14451500 -3.41597600 -1.21866800

H -4.97397800 -2.86521000 1.14765200

H -3.76451100 1.39312200 -2.38121600

H -4.36743000 -0.77611400 -3.85637000

H -3.27738000 -2.91259200 -2.66510800

H -6.70226100 0.82079800 -0.38143700

C 5.28264400 1.68219200 -0.62986400

C 5.67273500 2.59549700 -1.60652500

C 4.75558100 3.52527500 -2.08632400

C 3.45208200 3.52294300 -1.59658500

C 3.05320500 2.61602400 -0.61863200

H 6.01063900 0.95546500 -0.27756100

H 6.69058600 2.57906000 -1.98513700

H 5.05255800 4.24095100 -2.84709400

H 2.71539700 4.22214500 -1.98473000

C 3.98207400 1.68953900 -0.11973100

C 3.52713100 0.71623800 0.95057000

C 2.28622200 1.08890300 1.70985400

C 1.60788200 2.47249800 -0.19577500

C 1.47094600 2.18389500 1.29527200

H 1.03392400 3.36471900 -0.49566900

O 1.12977500 1.30055600 -0.79996000

H 2.28009000 0.84722500 2.76957700

H 1.03853400 2.89461500 1.99282800

Cl -0.05147800 -0.24552700 3.31479000

H 3.52721800 -0.32474700 0.60985100

H 5.86829600 -0.01366100 1.53021000

C 4.42675200 -0.80768800 3.22586900

H 3.44618500 -0.61317600 3.66601800

H 4.37465400 -1.67325000 2.56350700

H 5.16617000 -0.96464800 4.01171900

C 5.67352500 -1.88240400 -0.51734900

O 6.59352500 -1.26127300 0.39973200

C 7.83524000 -0.98009100 -0.26647700

C 7.54920900 -1.15475200 -1.75452100

C 6.50547400 -2.27384800 -1.73436600

H 5.20034800 -2.72786400 -0.00900100

H 4.90301600 -1.15299100 -0.79976500

H 8.15126200 0.03288900 0.00342200

H 8.59056500 -1.69122600 0.08800400

H 7.10980700 -0.23976300 -2.16715900

H 8.44535500 -1.40229600 -2.32698200

H 5.90297800 -2.32356500 -2.64352200

H 6.98619100 -3.24476400 -1.57783400

S 4.93211800 0.65165600 2.27006800

**S-b2**

P -1.37721600 -1.15200400 -0.03430000

C -0.77956200 -2.45006400 1.12410600

C -0.31952100 -3.67231300 0.61811200

C -0.57891100 -2.16499400 2.47931300

C 0.30820900 -4.59613200 1.45060500

H -0.45437000 -3.90543600 -0.43468700

C 0.05214400 -3.08677800 3.30923100

H -0.90653000 -1.21272900 2.88297400

C 0.49773600 -4.30517500 2.79925000

H 0.65024100 -5.54268600 1.04242500

H 0.20114000 -2.84845000 4.35817700

H 0.98654800 -5.02389100 3.45056300

C -2.25768200 -2.17480400 -1.28670400

C -2.05815500 -1.89985900 -2.64067200

C -3.12820400 -3.20778500 -0.91399500

C -2.73755000 -2.64244600 -3.61081700

H -1.36941300 -1.11049400 -2.93435400

C -3.79648600 -3.94895300 -1.88106800

H -3.28208900 -3.42900000 0.13987500

C -3.60478000 -3.66225000 -3.23476600

H -2.57900900 -2.41967000 -4.66162600

H -4.46914500 -4.74854000 -1.58355400

H -4.13051100 -4.23805700 -3.99075200

C -2.56896400 2.10557800 -0.08329400

H -2.57437200 1.68481100 -1.09960200

C -3.51101800 3.31872300 -0.06997100

H -3.50683200 3.83869500 0.89001100

H -3.25216300 4.04766500 -0.83631100

H -4.53769300 2.98338700 -0.26318500

P -0.70969800 2.38209900 0.18508900

C -0.49613500 2.85719000 2.01625300

C -1.44942500 3.92200300 2.57301300

H -2.46520800 3.52271800 2.65150800

H -1.12901500 4.18224200 3.59086500

H -1.47991400 4.84211900 1.98717900

C -0.66947400 1.59119700 2.87960500

H -1.70515800 1.24484900 2.90419100

H -0.01353800 0.78409900 2.54176700

H -0.38860400 1.85025600 3.91033800

C 0.96473900 3.29588900 2.20167500

H 1.20145300 4.23287800 1.68919000

H 1.15295500 3.45020000 3.27165300

H 1.63846900 2.50073200 1.85867400

C -0.27940100 3.88111000 -0.91863400

C 1.23473200 3.80218800 -1.18989500

H 1.46876100 2.85632400 -1.69029700

H 1.52043600 4.63379300 -1.84994800

H 1.83079800 3.87943200 -0.27392500

C -0.95708700 3.68709600 -2.28790700

H -0.76061500 2.69067400 -2.69357600

H -2.03497100 3.85513200 -2.25986500

H -0.53529500 4.42088400 -2.98735600

C -0.61558300 5.27608800 -0.37936100

H -1.67706300 5.39104800 -0.14403100

H -0.04056000 5.52501400 0.51734000

H -0.36445600 6.02091000 -1.14666100

Rh 0.68781100 0.25737100 -0.48862200

C -6.08295400 -1.60995600 -0.46433600

Fe -4.83001800 -0.14600400 0.45996400

C -5.40873300 -0.87552800 -1.47501500

C -6.87612600 -0.70094200 0.28701200

C -6.68740300 0.59703800 -0.25642900

C -5.77840700 0.48759900 -1.34438800

C -3.50967500 -0.96159400 1.94499600

C -4.33157300 0.02839000 2.52900300

C -4.07496500 1.25387200 1.85927600

C -3.08591400 1.03507700 0.85674800

C -2.72522300 -0.35587700 0.91619500

H -4.70314500 -1.27742300 -2.18939900

H -7.47458700 -0.94544800 1.15414700

H -7.11411100 1.51450600 0.12679100

H -5.40647700 1.30390900 -1.94962600

H -3.47137400 -2.00726600 2.22233000

H -5.06597200 -0.12803100 3.30755800

H -4.59109500 2.18595200 2.04520600

H -5.97626700 -2.66942600 -0.27749700

C 5.47151900 0.74099000 1.74621500

C 6.03594100 -0.00917800 2.77788300

C 5.40796600 -1.17170200 3.21295300

C 4.20537200 -1.56509400 2.62826400

C 3.63321900 -0.82440200 1.59754500

H 5.96763800 1.65654600 1.43328000

H 6.96057500 0.32252000 3.23988900

H 5.84398900 -1.75957400 4.01515400

H 3.68253900 -2.45188300 2.97951500

C 4.28415700 0.33130500 1.13488500

C 3.63839300 1.09881900 -0.01193500

C 2.73786100 0.27563500 -0.90726000

C 2.27636900 -1.15999000 1.01452800

C 2.28966700 -1.02062800 -0.50305900

H 1.97149700 -2.17098100 1.33353600

O 1.36542500 -0.17345400 1.41306900

H 2.85135800 0.43532300 -1.97436900

H 2.24446500 -1.87446600 -1.17442700

Cl 0.45046200 0.53889600 -2.99635300

H 3.17268100 2.02642600 0.33846500

C 4.25979200 2.62217800 -2.43245100

H 3.41619000 2.05187400 -2.82713500

H 3.91446600 3.58465100 -2.05294300

H 5.01540300 2.77888500 -3.20362600

S 5.07272700 1.76436500 -1.05757700

H 5.41008400 0.51903300 -1.65368700

C 5.11471300 -1.97341800 -2.35331800

O 5.98210600 -0.84089800 -2.16322700

C 7.06946500 -1.27457600 -1.33876900

C 6.44547700 -2.23100900 -0.32493700

C 5.23956200 -2.83014400 -1.08190200

H 4.11017900 -1.58509500 -2.52896500

H 5.45864100 -2.51088400 -3.24404600

H 7.81251400 -1.76965700 -1.97657700

H 7.52085000 -0.38263800 -0.89299000

H 7.15783400 -2.99060500 0.00409600

H 6.10580700 -1.67971300 0.55481400

H 5.39888800 -3.87921000 -1.34120800

H 4.33540400 -2.76377600 -0.47244300

**S-b3**

P -1.91186200 1.05867000 0.12555700

C -1.71801400 2.55641500 -0.92724600

C -1.82063500 3.82337300 -0.33849600

C -1.27047600 2.46458800 -2.24923600

C -1.50260700 4.97147700 -1.05975800

H -2.15217300 3.91425400 0.69246400

C -0.94717300 3.61230700 -2.96748900

H -1.16689500 1.49180800 -2.71810200

C -1.06130600 4.86917000 -2.37681800

H -1.59765100 5.94538800 -0.58874700

H -0.59915900 3.52175100 -3.99218100

H -0.81038400 5.76276200 -2.94072300

C -3.28366800 1.59864400 1.22930000

C -3.17534600 1.37391400 2.60261600

C -4.43313300 2.21961400 0.72180100

C -4.21734600 1.74975000 3.45568700

H -2.27737400 0.90674200 3.00106000

C -5.46473000 2.59635700 1.57283300

H -4.51782300 2.40930400 -0.34572000

C -5.35963700 2.35583700 2.94489500

H -4.12600200 1.56701700 4.52212400

H -6.35249900 3.07607700 1.17021000

H -6.16794800 2.64712200 3.60918800

C -1.70063800 -2.42012800 -0.06720300

H -1.98318300 -2.12338800 0.95378300

C -2.06398800 -3.89991600 -0.25125500

H -1.74853300 -4.28696400 -1.22226100

H -1.61139800 -4.52960000 0.51246200

H -3.15225100 -4.01993900 -0.17894200

P 0.12629600 -1.91670800 -0.08855300

C 0.71106300 -2.10209400 -1.89704600

C 0.33686100 -3.40044600 -2.62296400

H -0.74077000 -3.43678300 -2.80888200

H 0.82991800 -3.40887200 -3.60465200

H 0.63063800 -4.30869100 -2.09386500

C 0.13708400 -0.93382200 -2.72352100

H -0.94135200 -1.02243400 -2.87186100

H 0.37589100 0.03052100 -2.26531500

H 0.60588700 -0.96547400 -3.71762600

C 2.23479700 -1.89965900 -1.90795200

H 2.77887500 -2.68293500 -1.37133700

H 2.58656800 -1.90502900 -2.94831400

H 2.46898200 -0.91409000 -1.48561700

C 1.01944600 -3.21364500 0.99695000

C 2.32664500 -2.55640800 1.48493600

H 2.10144600 -1.66392900 2.07930100

H 2.87318400 -3.27145300 2.11534900

H 2.98508400 -2.27050100 0.65556700

C 0.18474700 -3.46315700 2.26730500

H -0.11607500 -2.52543600 2.74211900

H -0.70259000 -4.07133500 2.08278800

H 0.80854800 -4.01559300 2.98254500

C 1.35411100 -4.56209900 0.34915600

H 0.46700600 -5.07270500 -0.03632100

H 2.07385900 -4.46536700 -0.46994200

H 1.80638500 -5.21476100 1.10811300

Rh 0.43464300 0.48447200 0.85654700

C -6.44082900 -0.43170100 -0.06251600

Fe -4.59619000 -1.19220400 -0.82736300

C -5.63282900 -0.86193500 1.02255000

C -6.69246700 -1.55399100 -0.89732700

C -6.03445000 -2.67712600 -0.33022300

C -5.37704100 -2.24705200 0.85484800

C -3.55972100 0.18665000 -2.11109500

C -3.85579600 -0.99830900 -2.82147100

C -3.20470900 -2.07201200 -2.15932200

C -2.49586900 -1.56137700 -1.03287400

C -2.71291100 -0.13815400 -1.00809000

H -5.24287900 -0.23367700 1.81216500

H -7.24472400 -1.54486000 -1.82731100

H -5.99528200 -3.67194000 -0.75429600

H -4.75703200 -2.85843800 1.49761900

H -3.91036900 1.18132200 -2.35442300

H -4.50543900 -1.08165300 -3.68237300

H -3.28670800 -3.11433800 -2.43508800

H -6.77073100 0.58286900 -0.23837100

C 5.30683600 2.02468800 -0.65362200

C 5.66709900 3.06228300 -1.51356000

C 4.70490100 3.96306200 -1.95678900

C 3.38216100 3.81384000 -1.54753700

C 3.01224400 2.78589000 -0.68621700

H 6.08366500 1.33212400 -0.33487400

H 6.69962500 3.15828300 -1.83457000

H 4.98244400 4.77184200 -2.62598100

H 2.61164100 4.49187600 -1.90661600

C 3.98606100 1.88602800 -0.22241900

C 3.48732900 0.77361800 0.68711900

C 2.29279500 1.12595300 1.56626900

C 1.57483800 2.52166000 -0.29380900

C 1.46510000 2.23257500 1.20194400

H 0.93809700 3.36835300 -0.59818300

O 1.17732100 1.31134700 -0.88246700

H 2.37925300 0.86955700 2.62233700

H 1.07235500 2.94782000 1.91809800

Cl -0.08961200 -0.11925900 3.23994400

H 3.31666200 -0.14777100 0.12274300

C 5.36321100 1.73294100 2.63706500

H 5.78507300 2.38197800 1.87053000

H 4.47534900 2.19531300 3.07432700

H 6.08803800 1.49744600 3.41620100

S 4.78691200 0.18172400 1.90440000

H 5.82450600 -0.14358900 1.05727800

C 7.46039500 -2.10747300 0.38770700

O 6.77269600 -0.97350200 -0.16809600

C 5.91286500 -1.39984300 -1.24330700

C 5.73586300 -2.89803300 -1.03886700

C 7.11862000 -3.29270200 -0.51449000

H 8.52809900 -1.87879900 0.42793200

H 7.09397000 -2.26825500 1.41085300

H 4.98262200 -0.82488700 -1.19290800

H 6.40567300 -1.17376000 -2.19578200

H 4.96903600 -3.09170400 -0.27957600

H 5.44941600 -3.41533500 -1.95678200

H 7.12655400 -4.24125000 0.02591600

H 7.83252300 -3.35964000 -1.34149300

**S-b4**

P -1.42371000 0.90519300 0.75985800

C -1.17582000 2.72500600 0.85937800

C -0.90918100 3.31325400 2.10176500

C -1.04343600 3.50369400 -0.29510800

C -0.54102500 4.65358800 2.19090300

H -0.99020900 2.72091400 3.00942600

C -0.67039200 4.84142600 -0.20538000

H -1.21831300 3.05870800 -1.26892000

C -0.41771600 5.42172200 1.03631800

H -0.34790400 5.09444200 3.16445100

H -0.57048700 5.42990700 -1.11265900

H -0.12739500 6.46615500 1.10221400

C -2.39639200 0.60719500 2.29751000

C -2.05238600 -0.47995200 3.10172300

C -3.47126400 1.42541500 2.67273400

C -2.78341100 -0.75481300 4.26069800

H -1.20996500 -1.10689100 2.82198500

C -4.19362300 1.15360600 3.82874500

H -3.73918700 2.28040600 2.05651200

C -3.85157400 0.05730600 4.62468500

H -2.50932000 -1.60573800 4.87702300

H -5.02546300 1.79243900 4.11153800

H -4.41872100 -0.15672900 5.52572400

C -1.93181400 -1.62953900 -1.56306000

H -1.89477000 -2.02271900 -0.53639900

C -2.63448500 -2.67300200 -2.44341300

H -2.60306200 -2.41282200 -3.50394100

H -2.18938000 -3.66156400 -2.33498700

H -3.68777300 -2.75023900 -2.14719200

P -0.09831800 -1.24144600 -1.87282200

C -0.01924000 -0.23477900 -3.48571200

C -0.85940400 -0.75222800 -4.65996500

H -1.92759900 -0.63434300 -4.45511800

H -0.63312500 -0.14503300 -5.54679700

H -0.66452300 -1.79609800 -4.91435700

C -0.47008700 1.20846000 -3.18740800

H -1.53665500 1.27127600 -2.95928700

H 0.11174700 1.64471700 -2.37023500

H -0.29518700 1.80608900 -4.09311500

C 1.45864800 -0.14401900 -3.89645000

H 1.84851700 -1.08981500 -4.28329700

H 1.55577000 0.59996500 -4.69677500

H 2.07405000 0.19407700 -3.05338200

C 0.72453300 -2.94206000 -2.16914400

C 2.24044400 -2.74899000 -1.97749500

H 2.43916900 -2.19905900 -1.05166200

H 2.72329000 -3.73518800 -1.92355800

H 2.70437700 -2.20186000 -2.80135400

C 0.26391600 -3.87628300 -1.03580300

H 0.39419400 -3.41228000 -0.05328600

H -0.77995400 -4.18319000 -1.13127400

H 0.87104900 -4.79196800 -1.06972100

C 0.49721900 -3.62942900 -3.52069300

H -0.56138200 -3.77623000 -3.74852400

H 0.95027400 -3.07153500 -4.34520600

H 0.97226700 -4.61989000 -3.49817400

Rh 0.91109600 -0.01653600 0.24557000

C -6.12085400 -0.05337800 0.97866700

Fe -4.63384100 -0.17399600 -0.55117900

C -5.19203100 -1.07065800 1.31892400

C -6.72812300 -0.40685700 -0.25713300

C -6.16788500 -1.64014900 -0.68269900

C -5.21627000 -2.04812400 0.29152300

C -3.65110400 1.69739300 -0.94517800

C -4.31119500 1.24791100 -2.11116200

C -3.75121700 -0.00695900 -2.46839600

C -2.73585300 -0.34466000 -1.52793400

C -2.66706500 0.73098100 -0.57467900

H -4.54768500 -1.07914800 2.18669800

H -7.44979800 0.18401300 -0.80490100

H -6.39010100 -2.15134700 -1.61009500

H -4.59013400 -2.92966500 0.24330100

H -3.84926300 2.61946200 -0.41426000

H -5.13146600 1.74363500 -2.61252600

H -4.08514700 -0.62936900 -3.28733900

H -6.30040800 0.85103900 1.54413200

C 5.42971600 2.01690000 -1.55089500

C 5.69362900 3.34498900 -1.87300400

C 4.82498000 4.34697100 -1.44758700

C 3.69056100 4.00683200 -0.71765900

C 3.41790500 2.67925000 -0.39000000

H 6.11556000 1.23986900 -1.88043400

H 6.57559600 3.59444500 -2.45492500

H 5.02685000 5.38540400 -1.69312900

H 2.98601100 4.77282700 -0.40119800

C 4.30195600 1.67247400 -0.80096500

C 4.02187500 0.22615400 -0.42389200

C 2.97586500 0.03236100 0.65590300

C 2.11388400 2.28578400 0.26888600

C 2.28676900 1.13057700 1.25116400

H 1.65242700 3.16579800 0.74772900

O 1.28555900 1.73319700 -0.72315400

H 3.17663000 -0.82021600 1.29490600

H 2.14148500 1.24556500 2.32271100

Cl 0.86840100 -2.00222200 1.92866200

H 3.81305100 -0.36961000 -1.32037900

C 6.00311500 0.57626600 1.54920700

H 6.79600900 0.13361800 2.15503700

H 6.34183500 1.52667900 1.13329900

H 5.10464000 0.74066500 2.15024700

S 5.62481000 -0.56301800 0.18584300

H 4.97523000 -1.97244800 1.25225400

C 4.14317800 -2.46085800 3.18538700

O 4.68345200 -2.81055000 1.85771200

C 3.69028600 -3.66463900 1.17628600

C 3.19246600 -4.54956600 2.30347100

C 3.32011700 -3.68730600 3.58323600

H 5.01344500 -2.25990700 3.80961900

H 3.51333100 -1.57632200 3.07047000

H 2.89717800 -3.01415100 0.80268700

H 4.23092900 -4.17152600 0.37731900

H 2.15276500 -4.82385500 2.11566100

H 3.79933000 -5.45436700 2.38158300

H 2.33715300 -3.35686400 3.91959400

H 3.80999600 -4.24701800 4.38176300

**S-b5**

P -1.60367200 0.96106800 0.59325400

C -1.52903500 2.78048300 0.32797300

C -1.46323300 3.63810600 1.43291000

C -1.33750500 3.31473200 -0.95105800

C -1.23260900 5.00120400 1.26325500

H -1.59059900 3.23889700 2.43539800

C -1.10333800 4.67620900 -1.11900900

H -1.35428700 2.66160300 -1.81711000

C -1.05102100 5.52461600 -0.01414300

H -1.19146200 5.65116600 2.13217500

H -0.95312200 5.07441200 -2.11804500

H -0.86868200 6.58666200 -0.14939900

C -2.70905700 0.88344200 2.06665800

C -2.37033600 0.03711600 3.12394000

C -3.88330500 1.64467000 2.14578700

C -3.20750200 -0.05598700 4.24027300

H -1.45128300 -0.54269800 3.07602500

C -4.71024200 1.55504000 3.25847800

H -4.14804600 2.31294200 1.32993000

C -4.37382000 0.69803200 4.30896300

H -2.93731100 -0.71761000 5.05779200

H -5.61754400 2.15038100 3.30946500

H -5.02066900 0.62637000 5.17839700

C -1.68378100 -2.00667500 -1.19651900

H -1.73439100 -2.19002900 -0.11283000

C -2.20977900 -3.25933700 -1.91251500

H -2.11401300 -3.19011100 -2.99803800

H -1.69130200 -4.16342900 -1.59679300

H -3.27356200 -3.39022900 -1.67910300

P 0.14261500 -1.52061600 -1.39379900

C 0.33960700 -0.88068400 -3.17736200

C -0.33265300 -1.69753000 -4.28734300

H -1.42159100 -1.62960000 -4.20994500

H -0.05326700 -1.26609700 -5.25813700

H -0.04675200 -2.75155300 -4.29832500

C -0.23463300 0.54847000 -3.23606700

H -1.32314000 0.55813600 -3.13877500

H 0.21277800 1.18981800 -2.47001200

H 0.00792400 0.96655900 -4.22318000

C 1.84379500 -0.74792800 -3.45937200

H 2.35122100 -1.71406200 -3.53955700

H 1.97807900 -0.22708500 -4.41562900

H 2.32328700 -0.14001800 -2.68283700

C 1.08714400 -3.17163500 -1.21788900

C 2.53990600 -2.80713500 -0.85625400

H 2.56847400 -2.17119400 0.03937100

H 3.08912500 -3.74285200 -0.66741500

H 3.04865900 -2.27869500 -1.66768300

C 0.52184200 -3.91656400 0.00373900

H 0.45767700 -3.26126600 0.87848000

H -0.46278100 -4.35139200 -0.17568500

H 1.19638600 -4.75161500 0.24537400

C 1.11322800 -4.12036800 -2.42272700

H 0.11157100 -4.40904200 -2.75055800

H 1.63831600 -3.68832700 -3.27948100

H 1.64604100 -5.03961300 -2.14148900

Rh 0.88024000 0.23844100 0.50713900

C -6.21053700 -0.35864300 0.60029600

Fe -4.57685100 -0.64130100 -0.74878000

C -5.25396300 -1.21336600 1.20772000

C -6.67125300 -0.98709400 -0.58857100

C -5.99354600 -2.22796400 -0.71819100

C -5.11595400 -2.36512600 0.39189700

C -3.69648400 1.19898900 -1.42580100

C -4.19596100 0.47303100 -2.53037100

C -3.51189600 -0.77015000 -2.57518300

C -2.57922600 -0.82265000 -1.49891000

C -2.68929200 0.41846700 -0.78142800

H -4.69497800 -1.00211000 2.10890100

H -7.37480500 -0.57185800 -1.29746400

H -6.09234400 -2.92235700 -1.54203900

H -4.43295300 -3.18602000 0.56775000

H -4.01462300 2.18506300 -1.11352100

H -4.99436400 0.78312900 -3.19089000

H -3.71065700 -1.56652200 -3.27941500

H -6.50330100 0.61759700 0.96186000

C 5.36989700 2.78419700 -0.94117400

C 5.52865800 4.11989700 -1.30939200

C 4.45820100 5.00129400 -1.20868200

C 3.23172400 4.53970600 -0.73594600

C 3.06669300 3.20851800 -0.36826000

H 6.22876100 2.12102200 -1.00035800

H 6.49239700 4.46758600 -1.66891000

H 4.57878300 6.04228500 -1.49359100

H 2.38145600 5.21273500 -0.64958300

C 4.14409600 2.31634300 -0.47236200

C 3.87214800 0.86356100 -0.09391600

C 2.86973600 0.71008100 1.05242400

C 1.74704200 2.66160900 0.12516300

C 1.96324400 1.76758700 1.34363200

H 1.05808000 3.49259200 0.34223700

O 1.22187500 1.76280700 -0.81839600

H 3.21959800 0.11635800 1.89433400

H 1.71471500 2.07775900 2.35441400

Cl 0.81503900 -1.39576100 2.47166500

H 3.52878400 0.32241800 -0.98179400

C 5.92879400 -0.77022500 -1.17961500

H 6.14585100 0.01264500 -1.90908300

H 6.83446200 -1.35612900 -1.00691500

H 5.13497100 -1.41379600 -1.56535700

C 3.90861900 -2.50347600 3.08958500

O 5.04281000 -2.30864400 2.14184900

C 5.27812900 -3.51595800 1.33797900

C 4.45137300 -4.58742900 2.03291200

C 3.27742300 -3.80017800 2.62566100

H 4.37659700 -2.55969700 4.07295300

H 3.23415300 -1.64985900 3.00215700

H 4.92446600 -3.29680000 0.32793000

H 6.35440600 -3.68862500 1.34151500

H 4.12771900 -5.35284900 1.32353700

H 5.04099900 -5.06980600 2.81815800

H 2.51580100 -3.57497100 1.87594600

H 2.79032100 -4.31917800 3.45297100

H 5.00865700 -1.44283700 1.51380300

S 5.43333300 -0.00763500 0.39313800

**S-b6**

P -1.51581900 -1.17189400 -0.09093700

C -0.94924500 -2.49971600 1.05216100

C -0.58122800 -3.74994500 0.53986500

C -0.67874400 -2.21911500 2.39609400

C 0.02407500 -4.70362600 1.35527300

H -0.76797300 -3.98091800 -0.50528500

C -0.06937800 -3.17011300 3.20874500

H -0.93194500 -1.24617100 2.80349900

C 0.28412200 -4.41563600 2.69284700

H 0.29345700 -5.67121700 0.94167300

H 0.13584200 -2.93300800 4.24846600

H 0.75550700 -5.15757900 3.33107700

C -2.48743100 -2.16142500 -1.30457300

C -2.31579600 -1.91505500 -2.66769000

C -3.39653000 -3.14501700 -0.89159000

C -3.06054500 -2.63580200 -3.60605700

H -1.59885400 -1.16356600 -2.99188000

C -4.12961900 -3.86486700 -1.82703000

H -3.52896000 -3.34448800 0.16945500

C -3.96517200 -3.60626100 -3.18995700

H -2.92369800 -2.43447600 -4.66426600

H -4.83198600 -4.62542300 -1.49717700

H -4.54153100 -4.16560800 -3.92111300

C -2.54156100 2.14694600 -0.08790000

H -2.57526200 1.73400700 -1.10686900

C -3.43028300 3.39931500 -0.05808200

H -3.39008900 3.91735400 0.90202200

H -3.14881000 4.11695200 -0.82699600

H -4.47299500 3.10891700 -0.23836400

P -0.66807800 2.33394600 0.16137900

C -0.42257700 2.77728300 2.00016200

C -1.35454500 3.84594900 2.58497800

H -2.37583500 3.46202800 2.66409900

H -1.02215300 4.08234400 3.60508300

H -1.37344300 4.77747300 2.01615300

C -0.59986000 1.49745400 2.84125200

H -1.63708100 1.15501700 2.86131900

H 0.04973500 0.69291600 2.48337300

H -0.31546500 1.73380600 3.87673500

C 1.04358500 3.19456800 2.18876200

H 1.30268200 4.12218900 1.67121500

H 1.23069200 3.34831400 3.25924500

H 1.70241600 2.38615100 1.85221100

C -0.16735600 3.82921300 -0.91570100

C 1.34085100 3.67926300 -1.20029100

H 1.52944600 2.76670100 -1.77527500

H 1.68057900 4.54131800 -1.79020500

H 1.94152300 3.64093700 -0.28472100

C -0.86366500 3.70951600 -2.28391900

H -0.73692100 2.71273900 -2.71404700

H -1.92800400 3.94940500 -2.24536700

H -0.39656600 4.43009600 -2.96831800

C -0.44043600 5.22523500 -0.34308000

H -1.49872100 5.38205900 -0.11602400

H 0.13526900 5.43111200 0.56395200

H -0.14955800 5.97461300 -1.09162300

Rh 0.58106100 0.17769900 -0.58475800

C -6.25242000 -1.40324300 -0.37441000

Fe -4.90352500 0.00007300 0.50492800

C -5.56977300 -0.70879400 -1.40737500

C -6.97975300 -0.45185300 0.39066700

C -6.74186500 0.83211600 -0.16665200

C -5.86858900 0.67177500 -1.27712300

C -3.58098900 -0.88410600 1.94846300

C -4.33782000 0.14176200 2.55844200

C -4.04088300 1.35577000 1.88449000

C -3.09084300 1.09423000 0.85472000

C -2.79718400 -0.31332700 0.89966500

H -4.90029200 -1.14827800 -2.13417800

H -7.56627500 -0.66176200 1.27494600

H -7.11607100 1.77140800 0.21885700

H -5.47154600 1.46463900 -1.89724700

H -3.58509400 -1.93130200 2.22252900

H -5.05763600 0.01711800 3.35605200

H -4.50526500 2.31135900 2.08664400

H -6.19288600 -2.46556600 -0.18248900

C 5.56630900 0.11493300 1.40441700

C 6.15792700 -0.82129700 2.25805700

C 5.45069800 -1.95784900 2.63519200

C 4.15249100 -2.14378800 2.15956500

C 3.56088700 -1.21519700 1.30734600

H 6.13750000 0.99792900 1.13210100

H 7.16142200 -0.64718200 2.63771600

H 5.89910300 -2.68545900 3.30518400

H 3.57489000 -3.01734500 2.45319000

C 4.27453400 -0.06786900 0.91254800

C 3.55284100 0.90365400 -0.01973800

C 2.62739600 0.20139000 -1.02470800

C 2.15648700 -1.37539000 0.77052700

C 2.15443600 -1.12090300 -0.73671700

H 1.77424100 -2.38004600 1.01882000

O 1.34484200 -0.35038700 1.26907300

H 2.76873300 0.48805800 -2.06687700

H 2.09323600 -1.91794900 -1.47388200

Cl 0.20517500 0.47025100 -3.05199500

H 2.99319400 1.62417700 0.58349200

C 5.19889300 3.27980800 0.07095500

H 5.72307300 2.92919000 0.95957500

H 5.84176800 3.95190200 -0.50030800

H 4.29836000 3.82147100 0.36954100

C 5.57611200 -1.39901800 -2.02277500

O 6.47590400 -0.23768600 -1.80553500

C 7.68216200 -0.63582800 -1.06021300

C 7.59159600 -2.15533500 -0.98410600

C 6.08515500 -2.43677500 -1.04243400

H 4.55468100 -1.05404300 -1.84286300

H 5.71550500 -1.67498100 -3.06891400

H 8.53187300 -0.25520100 -1.62593300

H 7.61870000 -0.16491700 -0.08006000

H 8.10817500 -2.61041300 -1.83404400

H 8.04417100 -2.52496100 -0.06165000

H 5.86480700 -3.44773000 -1.39080300

H 5.62122800 -2.29274800 -0.06393500

H 5.95115200 0.61081100 -1.40864600

S 4.68484800 1.94317800 -1.04577300

**cat-MeO-1**

P 0.55276800 1.08726700 0.13148400

C 1.15479600 2.39960500 1.28534000

C 1.71873100 3.58877200 0.81666300

C 0.93055500 2.25586800 2.65875100

C 2.08518600 4.59423700 1.70845300

H 1.83903900 3.73841000 -0.25130100

C 1.29340100 3.26150100 3.54915500

H 0.46831600 1.34613700 3.03513900

C 1.87968900 4.43313000 3.07551700

H 2.51828500 5.51465100 1.32743700

H 1.11229100 3.13120300 4.61247100

H 2.16252000 5.22132700 3.76793400

C 1.71898600 1.24482200 -1.29073300

C 1.18444100 1.17222200 -2.57909900

C 3.10045900 1.38668600 -1.13348000

C 2.02397000 1.20564400 -3.69235700

H 0.10447100 1.11530900 -2.68889600

C 3.93866200 1.43621400 -2.24440200

H 3.52405800 1.45880000 -0.13382300

C 3.40080500 1.33499700 -3.52717700

H 1.59793200 1.14625000 -4.68963700

H 5.01142600 1.55075400 -2.11072600

H 4.05422000 1.36678800 -4.39460100

C -0.64208800 -2.16709400 0.27522300

H -0.30913800 -2.16131500 -0.77326600

C -0.89487700 -3.62953800 0.67440400

H -1.05390600 -3.72229500 1.75199000

H -1.75294600 -4.06970900 0.17783400

H -0.01176400 -4.23174500 0.42197300

P -2.12933000 -0.97982400 0.12957400

C -3.15280400 -1.28581900 1.69725500

C -3.85597500 -2.64381400 1.81661800

H -3.16662900 -3.49080100 1.81960500

H -4.41001000 -2.66890000 2.76559000

H -4.58332100 -2.79425200 1.01434300

C -2.17200500 -1.11141000 2.86837800

H -1.42297200 -1.90817400 2.91848900

H -1.64602800 -0.15241900 2.78927700

H -2.73488400 -1.11691600 3.81112000

C -4.21167700 -0.17722100 1.81734600

H -5.00275700 -0.28134600 1.07263600

H -4.66494100 -0.24578100 2.81695600

H -3.77778000 0.81486800 1.67010600

C -3.04303800 -1.68183700 -1.40681800

C -4.50390000 -1.20590700 -1.38996200

H -4.54400100 -0.14379400 -1.11810200

H -4.92917400 -1.35393500 -2.39256000

H -5.11140800 -1.79137300 -0.69087000

C -2.35372400 -1.03385100 -2.62131800

H -2.46470100 0.05400200 -2.59205500

H -1.28172900 -1.26697900 -2.66176900

H -2.81773300 -1.42005100 -3.53974100

C -3.03847800 -3.19848300 -1.63291000

H -2.03391400 -3.59726600 -1.81183000

H -3.50239300 -3.75271100 -0.81059200

H -3.63089300 -3.40392900 -2.53475800

Rh -1.61523700 1.18169600 -0.29224700

C 4.26687100 -1.75475100 -0.79956900

Fe 2.54211000 -1.97539000 0.44778100

C 3.11690900 -1.91491800 -1.61745300

C 4.40372600 -2.91826800 0.00454600

C 3.33569600 -3.79821800 -0.31334800

C 2.54134100 -3.17665800 -1.31572600

C 2.31630000 -0.42365500 1.89797400

C 2.34401200 -1.67997900 2.54316100

C 1.24683200 -2.43659400 2.05554300

C 0.52519600 -1.65130300 1.10344800

C 1.19218500 -0.38605200 1.01751100

H 2.72097700 -1.17768700 -2.30478600

H 5.15560900 -3.08108800 0.76512600

H 3.13023700 -4.74757800 0.16349800

H 1.62741400 -3.57419400 -1.73817300

H 3.01463800 0.38874300 2.04880300

H 3.09242500 -2.02654700 3.24351900

H 1.03975700 -3.46184100 2.32611400

H 4.89816800 -0.87792100 -0.76784300

Cl -0.96299500 3.47218300 -1.19070700

O -3.67525800 1.51985200 -0.55193900

C -4.09603600 2.24374000 -1.65185000

H -5.10780400 2.66257200 -1.47930900

H -3.43582000 3.08591500 -1.90528500

H -4.18777800 1.61865500 -2.57404600

**cat-MeO-2**

P 0.56938000 1.07990600 0.16263800

C 1.17741100 2.37691300 1.32325800

C 1.68969400 3.58899800 0.85097900

C 1.02294600 2.20360300 2.70191500

C 2.07894600 4.58429900 1.74329200

H 1.75241800 3.75446500 -0.21870300

C 1.40267400 3.20282000 3.59396400

H 0.59922300 1.27784200 3.08359200

C 1.94054400 4.39487200 3.11603700

H 2.47631000 5.52037100 1.36094900

H 1.27240300 3.04996100 4.66174600

H 2.23929600 5.17664600 3.80919900

C 1.71506100 1.21550700 -1.27002500

C 1.18017900 1.05863900 -2.55119200

C 3.09312600 1.40812100 -1.13029300

C 2.00987600 1.07933700 -3.67236900

H 0.10520700 0.92664400 -2.65042800

C 3.92250400 1.43283500 -2.24863500

H 3.52077200 1.54089900 -0.13863500

C 3.38208200 1.26321200 -3.52355700

H 1.57908400 0.95891500 -4.66223200

H 4.99210300 1.58397500 -2.12674200

H 4.02931700 1.27995400 -4.39587000

C -0.71022900 -2.15074400 0.31694500

H -0.37122500 -2.19244900 -0.72865600

C -1.01330100 -3.59091000 0.76373900

H -1.15783900 -3.64280500 1.84605700

H -1.89601800 -4.01383700 0.29709800

H -0.15907500 -4.23566500 0.51659100

P -2.17169600 -0.93086000 0.11391100

C -3.22052600 -1.17404400 1.67524000

C -3.95106700 -2.51249600 1.83913700

H -3.27183400 -3.36449700 1.90645400

H -4.53415000 -2.48372800 2.77026800

H -4.65415700 -2.69848400 1.02285200

C -2.25462100 -0.97216800 2.85442000

H -1.51866600 -1.77814700 2.94266000

H -1.71251600 -0.02475900 2.74924000

H -2.83029000 -0.93614700 3.78899000

C -4.25235800 -0.03553400 1.73171700

H -5.00384000 -0.10890000 0.94210600

H -4.76786900 -0.08005600 2.70185700

H -3.77355900 0.94124000 1.61969800

C -3.06974600 -1.67179700 -1.41563600

C -4.53989500 -1.22469700 -1.44336000

H -4.63450900 -0.14215500 -1.32227100

H -4.96033800 -1.49792800 -2.42158400

H -5.13793000 -1.73364500 -0.68065900

C -2.36993100 -1.04059700 -2.63416700

H -2.50668100 0.04527100 -2.63661300

H -1.29316000 -1.25432400 -2.64600800

H -2.80759300 -1.45950900 -3.55123600

C -3.05126600 -3.19313700 -1.60957400

H -2.03956000 -3.59397500 -1.73304200

H -3.55255400 -3.72942500 -0.79712100

H -3.60143300 -3.42027700 -2.53252000

Rh -1.53562700 1.22163400 -0.31880100

C 4.19767400 -1.83447200 -0.80147100

Fe 2.48191200 -2.03492100 0.45832000

C 3.04011000 -1.98232600 -1.61025900

C 4.32755900 -2.99935900 0.00202700

C 3.24714500 -3.86750900 -0.30743100

C 2.45181800 -3.23741700 -1.30373100

C 2.31661500 -0.48287300 1.90636900

C 2.31329600 -1.73804700 2.55415400

C 1.18823600 -2.46119100 2.07974100

C 0.47792300 -1.65574300 1.13427700

C 1.18400300 -0.41147700 1.03877400

H 2.64926800 -1.24118200 -2.29562700

H 5.08215600 -3.16945300 0.75832200

H 3.03521200 -4.81461700 0.17098700

H 1.53075700 -3.62533700 -1.71961700

H 3.04202500 0.30790600 2.04500700

H 3.05886000 -2.10730400 3.24599200

H 0.95686300 -3.48013700 2.35354300

H 4.83502400 -0.96174400 -0.77306400

Cl -3.80489300 2.08959300 -1.04813200

O -0.61025600 3.08190500 -0.73662300

C -0.86324200 3.66089200 -1.96560800

H -0.46546000 4.69529400 -1.98803000

H -0.37421900 3.13443900 -2.81611600

H -1.93780400 3.71599900 -2.19777000

**cat-2MeO**

P 0.50201300 1.07031200 0.18214700

C 0.94183600 2.42194500 1.35760800

C 1.24327400 3.69878700 0.87178900

C 0.84796600 2.23212800 2.74024800

C 1.49181800 4.74625300 1.75501500

H 1.23780400 3.86792300 -0.19896600

C 1.09247300 3.28154000 3.62205100

H 0.57908600 1.25452100 3.13292000

C 1.42513900 4.54143800 3.13075100

H 1.72532400 5.73235600 1.36302200

H 1.01809300 3.11389000 4.69311600

H 1.61802100 5.36169400 3.81707100

C 1.69534900 1.36415900 -1.19211000

C 1.21075900 1.24982600 -2.49671000

C 3.04956100 1.64992500 -0.99302900

C 2.07042900 1.39351900 -3.58579100

H 0.14982800 1.05572700 -2.63554000

C 3.90963800 1.79382300 -2.07833400

H 3.43377100 1.76089800 0.01895700

C 3.42163300 1.65919000 -3.37880000

H 1.67939600 1.30476700 -4.59537300

H 4.96138900 2.01284700 -1.91202400

H 4.09375200 1.77005600 -4.22529600

C -0.54336100 -2.24962600 0.28623400

H -0.19532100 -2.27187100 -0.75683700

C -0.76873400 -3.70354900 0.73080800

H -0.93237500 -3.76448700 1.81041400

H -1.61750800 -4.17288300 0.24437100

H 0.12432700 -4.29986100 0.49985800

P -2.06593000 -1.11680000 0.06808500

C -3.11135200 -1.38232600 1.62508500

C -3.81223800 -2.73626100 1.78596200

H -3.11101900 -3.57343900 1.82914600

H -4.37869000 -2.73426700 2.72811900

H -4.52435200 -2.92817200 0.97919500

C -2.16155800 -1.15838000 2.81316000

H -1.42310300 -1.95948800 2.92392300

H -1.62302800 -0.20964600 2.70034400

H -2.74896700 -1.11143500 3.74022600

C -4.15715000 -0.25457800 1.66542100

H -4.86099800 -0.29534600 0.83119800

H -4.71951900 -0.32999500 2.60735100

H -3.66691000 0.72216500 1.61183600

C -2.93508800 -1.89605700 -1.45434100

C -4.41074700 -1.46370100 -1.48630800

H -4.48266200 -0.37456400 -1.36600700

H -4.82647000 -1.74955000 -2.46370900

H -5.00567700 -1.97403800 -0.72151300

C -2.25070600 -1.25057500 -2.67448300

H -2.42341800 -0.16966000 -2.67461200

H -1.16783100 -1.43266700 -2.68572500

H -2.67539900 -1.68692600 -3.59002900

C -2.87746100 -3.41726300 -1.63446900

H -1.85583200 -3.78906800 -1.77038500

H -3.34832800 -3.96078700 -0.80828500

H -3.43573400 -3.66966400 -2.54622400

Rh -1.59359800 1.04139000 -0.38324700

C 4.37618300 -1.51720600 -0.74370700

Fe 2.64497600 -1.87493700 0.46163100

C 3.25009600 -1.70017100 -1.58866700

C 4.55873300 -2.70582900 0.01408300

C 3.54177700 -3.62359300 -0.35975000

C 2.73309400 -3.00024200 -1.34950900

C 2.33192900 -0.37560500 1.93826900

C 2.42069900 -1.64084300 2.56018600

C 1.36406200 -2.44130300 2.05304800

C 0.60329600 -1.67622200 1.11455600

C 1.21062300 -0.37642200 1.05364800

H 2.82978800 -0.95539800 -2.25237800

H 5.30605000 -2.86391700 0.78019000

H 3.37646400 -4.60131300 0.07343300

H 1.84716500 -3.42450400 -1.80469800

H 2.98996800 0.46726300 2.10363800

H 3.18410600 -1.96393800 3.25568300

H 1.20713400 -3.47967900 2.30620000

H 4.95889000 -0.60981300 -0.66540600

O -1.00255400 3.02449400 -0.90031800

O -3.58356000 1.34420000 -1.07792400

C -1.36716700 3.42869700 -2.16692600

H -0.63451200 3.13717500 -2.95693300

H -2.34608300 3.02528400 -2.48533000

H -1.43992200 4.53388500 -2.23147300

C -4.17488300 2.49599400 -0.60321800

H -4.62930900 2.37206100 0.40809900

H -3.47208100 3.34525600 -0.52414400

H -5.00463500 2.82734700 -1.26190800

**B3LYP/6-31G(d), Lanl2DZ, SMD(THF)-computed Coordinates and Energies of Stationary Points**

**MeOH-b3lyp**

Zero-point correction= 0.051377 (Hartree/Particle)

Thermal correction to Energy= 0.054669

Thermal correction to Enthalpy= 0.055614

Thermal correction to Gibbs Free Energy= 0.028650

Sum of electronic and zero-point Energies= -115.667177

Sum of electronic and thermal Energies= -115.663885

Sum of electronic and thermal Enthalpies= -115.662941

Sum of electronic and thermal Free Energies= -115.689904

C -0.66411000 -0.01884400 -0.00003200

H -1.03445900 -0.54639700 -0.89255600

H -1.03457800 -0.54589100 0.89274400

H -1.08728900 0.99037200 -0.00035600

O 0.75172500 0.12337700 0.00002700

H 1.12718600 -0.77203300 0.00013900

**THF-b3lyp**

Zero-point correction= 0.117144 (Hartree/Particle)

Thermal correction to Energy= 0.121234

Thermal correction to Enthalpy= 0.122178

Thermal correction to Gibbs Free Energy= 0.090165

Sum of electronic and zero-point Energies= -232.338430

Sum of electronic and thermal Energies= -232.334339

Sum of electronic and thermal Enthalpies= -232.333395

Sum of electronic and thermal Free Energies= -232.365408

C 1.16717700 -0.42776800 0.13216800

O -0.00000500 -1.25471500 -0.00001900

C -1.16718700 -0.42775600 -0.13214400

C -0.73293700 0.99583300 0.22856700

C 0.73295400 0.99581800 -0.22857800

H 1.53509100 -0.47946800 1.16879600

H 1.95301100 -0.82076700 -0.52440500

H -1.53515600 -0.47946600 -1.16875200

H -1.95299200 -0.82073800 0.52447500

H -1.34481200 1.75937300 -0.26174300

H -0.79410800 1.15135400 1.31248500

H 0.79412900 1.15130900 -1.31250000

H 1.34483900 1.75936000 0.26171500

**cod-b3lyp**

Zero-point correction= 0.180907 (Hartree/Particle)

Thermal correction to Energy= 0.188319

Thermal correction to Enthalpy= 0.189263

Thermal correction to Gibbs Free Energy= 0.149425

Sum of electronic and zero-point Energies= -311.858138

Sum of electronic and thermal Energies= -311.850726

Sum of electronic and thermal Enthalpies= -311.849782

Sum of electronic and thermal Free Energies= -311.889620

C -1.08684200 -1.10584000 0.66485000

H -1.77917700 -1.90683300 0.95267800

H -0.66317100 -0.73140000 1.60026000

C -1.92456700 -0.00039500 -0.01372400

H -2.72903500 0.30339000 0.67556200

H -2.44543700 -0.43898700 -0.87769600

C 1.92456700 0.00039500 -0.01371000

H 2.44544300 0.43899400 -0.87767500

H 2.72903000 -0.30339500 0.67557900

C 1.08683800 1.10583500 0.66486600

H 0.66316100 0.73138800 1.60027100

H 1.77917100 1.90682600 0.95270400

C 0.01784800 1.70146600 -0.22688800

H 0.30819500 2.62454700 -0.73067900

C -1.21011800 1.23802100 -0.49788300

H -1.81497400 1.84085300 -1.17832800

C 1.21012100 -1.23801700 -0.49788400

H 1.81498200 -1.84084400 -1.17833000

C -0.01784600 -1.70146400 -0.22690100

H -0.30819000 -2.62454100 -0.73070200

**OA-b3lyp**

Zero-point correction= 0.152233 (Hartree/Particle)

Thermal correction to Energy= 0.159405

Thermal correction to Enthalpy= 0.160350

Thermal correction to Gibbs Free Energy= 0.120403

Sum of electronic and zero-point Energies= -460.892108

Sum of electronic and thermal Energies= -460.884936

Sum of electronic and thermal Enthalpies= -460.883992

Sum of electronic and thermal Free Energies= -460.923939

C -0.00650000 1.38678300 1.42045900

C -0.16267300 2.58735700 0.69653300

C -0.16267300 2.58735700 -0.69653300

C -0.00650000 1.38678300 -1.42045900

C 0.15327400 0.21694800 -0.70361300

H -0.00663500 1.39514100 2.50787100

H -0.28043900 3.52435900 1.23487000

H -0.28043900 3.52435900 -1.23487000

H -0.00663500 1.39514100 -2.50787100

C 0.15327400 0.21694800 0.70361300

C 0.34504300 -1.26300100 1.07621000

C -0.94624400 -1.99792500 0.66750100

H 0.77431900 -1.48849000 2.05190000

C 0.34504300 -1.26300100 -1.07621000

C -0.94624400 -1.99792500 -0.66750100

H 0.77431900 -1.48849000 -2.05190000

O 1.23103700 -1.66658400 0.00000000

H -1.70878800 -2.34564300 1.35451200

H -1.70878800 -2.34564300 -1.35451200

**Josiphos-b3lyp**

Zero-point correction= 0.637176 (Hartree/Particle)

Thermal correction to Energy= 0.673280

Thermal correction to Enthalpy= 0.674224

Thermal correction to Gibbs Free Energy= 0.570278

Sum of electronic and zero-point Energies= -2048.989154

Sum of electronic and thermal Energies= -2048.953050

Sum of electronic and thermal Enthalpies= -2048.952106

Sum of electronic and thermal Free Energies= -2049.056052

P -0.94661300 1.12180200 0.64500800

C -0.33353200 2.61893100 -0.26792100

C -0.28729100 3.82812700 0.44925000

C 0.11479800 2.61367100 -1.59688200

C 0.17706700 4.99995200 -0.14968600

H -0.61800600 3.85476300 1.48566300

C 0.58778300 3.78559200 -2.19540500

H 0.09812500 1.69264800 -2.17078300

C 0.61786700 4.98188500 -1.47623400

H 0.20064600 5.92509900 0.42087200

H 0.93101600 3.76092700 -3.22684600

H 0.98609800 5.89199000 -1.94274700

C -2.77628900 1.45860300 0.63881600

C -3.50046000 1.11494300 1.79225700

C -3.46210600 2.07975200 -0.41940900

C -4.87560700 1.35178600 1.87608300

H -2.98228800 0.66613000 2.63630200

C -4.83553300 2.31848400 -0.33784800

H -2.92129200 2.39000200 -1.30884900

C -5.54706900 1.95046300 0.80812200

H -5.41742800 1.07827000 2.77795600

H -5.34913800 2.79820200 -1.16737800

H -6.61519500 2.14156100 0.87184700

C 1.45633900 -1.35741600 0.41772500

H 1.03863700 -1.13494300 1.40719200

C 2.00069000 -2.79698200 0.45968500

H 2.41591600 -3.11662100 -0.49915100

H 2.78374800 -2.91188700 1.20991200

H 1.19235100 -3.49410600 0.71082400

P 2.70624900 0.10185400 0.22640600

C 4.08448000 -0.43148500 -1.01469100

C 5.10324700 -1.48811300 -0.55063200

H 4.62987000 -2.43389500 -0.27302500

H 5.80667200 -1.70244800 -1.36903500

H 5.69757000 -1.14258800 0.30061400

C 3.37689600 -0.93711700 -2.29037000

H 2.89976900 -1.91000500 -2.15148400

H 2.61542500 -0.23241000 -2.64459100

H 4.11879700 -1.04956400 -3.09350800

C 4.84309200 0.85636400 -1.41477700

H 5.41944500 1.28555800 -0.59155100

H 5.55052300 0.62297900 -2.22319900

H 4.15809600 1.62808400 -1.78438600

C 3.44119600 0.26723000 2.00965100

C 4.67254900 1.19500500 1.96493700

H 4.46834900 2.12448900 1.42119300

H 4.95311000 1.46953800 2.99157300

H 5.54360600 0.71331300 1.51064300

C 2.35369900 0.99246000 2.83392400

H 2.09445100 1.96306700 2.39737800

H 1.43129000 0.40873500 2.92081600

H 2.72553100 1.16937200 3.85324900

C 3.81915900 -1.02937000 2.74939800

H 2.94519300 -1.66117000 2.93830700

H 4.56453300 -1.62300600 2.21210400

H 4.24792200 -0.77548700 3.73002200

C -3.54284200 -2.52191300 0.08334800

Fe -1.57437600 -2.14938700 -0.49623100

C -2.71493200 -2.39895900 1.24053500

C -3.04552500 -3.60945000 -0.69816700

C -1.90836600 -4.15352600 -0.02654000

C -1.70544300 -3.40519400 1.17325100

C -1.57496500 -0.48899900 -1.73391900

C -1.04544700 -1.61009200 -2.42937400

C 0.10716100 -2.05813000 -1.71992700

C 0.31592500 -1.21860800 -0.57838700

C -0.74892800 -0.22757000 -0.58580800

H -2.82052000 -1.65720500 2.02023200

H -3.43820100 -3.94010400 -1.65164200

H -1.29258800 -4.97117000 -0.37990100

H -0.91612500 -3.56235600 1.89742500

H -2.47084800 0.05421900 -2.00092300

H -1.46672900 -2.07039500 -3.31463400

H 0.70450100 -2.91912000 -1.98584100

H -4.37928200 -1.88319900 -0.16988600

**RhCl-cod-b3lyp**

Zero-point correction= 0.370342 (Hartree/Particle)

Thermal correction to Energy= 0.392006

Thermal correction to Enthalpy= 0.392950

Thermal correction to Gibbs Free Energy= 0.318855

Sum of electronic and zero-point Energies= -1763.342373

Sum of electronic and thermal Energies= -1763.320709

Sum of electronic and thermal Enthalpies= -1763.319765

Sum of electronic and thermal Free Energies= -1763.393861

Rh -1.76370900 -0.11850300 -0.19227400

Rh 1.76370500 -0.11850300 -0.19225800

Cl -0.00000600 -1.88264100 0.15825800

Cl 0.00000100 1.04063600 -1.55758200

C -3.03357100 -0.76312100 1.43593900

C -3.34312900 -1.49023400 0.26887300

C -4.51772500 -1.24012100 -0.65732900

C -4.72530300 0.25568000 -0.98049800

C -3.40689200 1.02245200 -1.01211500

C -2.85326900 1.70314900 0.09101700

C -3.42660200 1.74979000 1.49428100

C -3.88958600 0.36658100 2.00040100

C 3.40692400 1.02229000 -1.01222900

C 2.85332300 1.70314600 0.09081700

C 3.42666300 1.74994300 1.49407200

C 3.88958600 0.36677700 2.00036800

C 3.03353500 -0.76296500 1.43603500

C 3.34308000 -1.49023200 0.26906100

C 4.51769000 -1.24027100 -0.65716400

C 4.72531200 0.25548500 -0.98051300

H -2.36521500 -1.24652900 2.14718200

H -2.90689800 -2.48520600 0.18183500

H -4.31906000 -1.78263400 -1.58876100

H -5.43707100 -1.67775300 -0.23740900

H -5.21459200 0.34739000 -1.95637100

H -5.40328600 0.71719900 -0.25496000

H -3.07250700 1.33884100 -1.99915800

H -2.14907500 2.50381200 -0.13422000

H -2.64018200 2.12937900 2.15696200

H -4.25015200 2.47923400 1.54803100

H -3.83298900 0.34536700 3.09429300

H -4.94088200 0.19552900 1.74585500

H 3.07255000 1.33856500 -1.99931100

H 2.14915900 2.50380700 -0.13451900

H 4.25024600 2.47935600 1.54772600

H 2.64026200 2.12965500 2.15670800

H 4.94087800 0.19565500 1.74585500

H 3.83297400 0.34569900 3.09426200

H 2.36515900 -1.24626400 2.14733300

H 2.90682100 -2.48520200 0.18214500

H 5.43702000 -1.67787700 -0.23718200

H 4.31901500 -1.78289400 -1.58852900

H 5.40330200 0.71706900 -0.25502400

H 5.21461000 0.34706500 -1.95639400

**M1-a-b3lyp**

Zero-point correction= 0.796399 (Hartree/Particle)

Thermal correction to Energy= 0.843809

Thermal correction to Enthalpy= 0.844753

Thermal correction to Gibbs Free Energy= 0.718421

Sum of electronic and zero-point Energies= -3079.691074

Sum of electronic and thermal Energies= -3079.643664

Sum of electronic and thermal Enthalpies= -3079.642720

Sum of electronic and thermal Free Energies= -3079.769052

P 0.44097200 -1.13648700 -0.07733900

C 0.12524700 -2.67643600 -1.06158200

C -0.35394100 -3.84223000 -0.43806300

C 0.21768300 -2.65784000 -2.46421400

C -0.69120600 -4.96910800 -1.19230800

H -0.46885900 -3.88403900 0.63925000

C -0.10720000 -3.78994600 -3.21324900

H 0.51058900 -1.75040400 -2.97656200

C -0.55857600 -4.95118500 -2.58152100

H -1.05643400 -5.85979600 -0.68776800

H -0.01802300 -3.75597800 -4.29588500

H -0.81453400 -5.82987500 -3.16775400

C 0.83825600 -1.82529600 1.59840000

C 0.26333900 -1.29956600 2.76059000

C 1.72849000 -2.90859800 1.71710400

C 0.56795600 -1.83601000 4.01645400

H -0.42621900 -0.47065000 2.68516100

C 2.02167200 -3.45318700 2.96691000

H 2.19090000 -3.33345700 0.83248900

C 1.44308600 -2.91668900 4.12221400

H 0.11144200 -1.41140500 4.90661600

H 2.70491600 -4.29526300 3.03892700

H 1.67314600 -3.34230500 5.09529400

C 1.93401600 2.01263700 0.36206600

H 1.87711900 1.55356800 1.35667000

C 2.93042400 3.18524700 0.44854900

H 3.06428700 3.68895000 -0.51104000

H 2.61875800 3.93421700 1.17359400

H 3.91135900 2.81033400 0.76133200

P 0.08497200 2.43631700 0.06674700

C -0.00429300 3.75721200 -1.34797500

C 0.42003700 5.18995700 -0.96792500

H 1.43704500 5.24815600 -0.57173300

H 0.39003400 5.80946600 -1.87548900

H -0.25907200 5.65168400 -0.24610200

C 0.89239300 3.27099100 -2.50660600

H 1.95693000 3.34480100 -2.27198500

H 0.65717900 2.24430100 -2.79836500

H 0.70814300 3.91263200 -3.37978600

C -1.45634600 3.80278900 -1.87442500

H -2.16899000 4.16323600 -1.12829500

H -1.49677900 4.49842000 -2.72477300

H -1.77970400 2.82140600 -2.23046000

C -0.49340100 3.27784600 1.72521400

C -1.89310300 3.89027700 1.50178700

H -2.57298200 3.20501800 0.98701700

H -2.33545000 4.12708200 2.47908400

H -1.85456400 4.82239100 0.93213500

C -0.63095500 2.17228800 2.78890500

H -1.36760300 1.42160100 2.49533800

H 0.31988700 1.66718100 2.98802300

H -0.96206800 2.62561600 3.73342800

C 0.42177200 4.36270400 2.33246600

H 1.34172100 3.93745700 2.74468100

H 0.68604100 5.15808600 1.63435500

H -0.11166100 4.83134000 3.17150600

Rh -1.27441100 0.29837600 -0.39462200

C 4.98556800 -1.95984300 0.78085300

Fe 4.05694000 -0.36914000 -0.21247000

C 4.50747200 -1.01209900 1.73788800

C 5.91878500 -1.29366000 -0.06880800

C 6.01216800 0.06592000 0.35758900

C 5.14204800 0.23660800 1.47774300

C 2.82430400 -1.05017500 -1.73982700

C 3.73557500 -0.08304000 -2.23955500

C 3.50816100 1.13340600 -1.53637600

C 2.44333300 0.94578200 -0.59675200

C 1.99568000 -0.42666000 -0.74133300

H 3.77335100 -1.20056300 2.50826400

H 6.43414600 -1.73099800 -0.91483800

H 6.61259200 0.83809800 -0.10676400

H 4.97300500 1.15848500 2.01906800

H 2.75663100 -2.07959300 -2.05992200

H 4.50043800 -0.25436700 -2.98643000

H 4.07917200 2.04145800 -1.66779600

H 4.68353200 -2.99584200 0.70185000

C -6.09124400 -0.32025800 1.97892800

C -7.25177600 -1.02139300 1.60924200

C -7.39535400 -1.54074500 0.31956800

C -6.38443000 -1.37589500 -0.64276900

C -5.24511500 -0.67511200 -0.27601100

H -5.98449600 0.08103400 2.98383700

H -8.05026800 -1.15591500 2.33421700

H -8.30404600 -2.07514300 0.05493000

H -6.50156800 -1.78304200 -1.64392400

C -5.10066600 -0.15365300 1.02272100

C -3.72815800 0.49206000 1.03941600

C -2.64563300 -0.59576700 0.95575100

H -3.57108300 1.31458700 1.73407900

C -3.95016300 -0.33267800 -0.98687400

C -2.80180500 -1.14353100 -0.36747900

H -3.95677100 -0.22951100 -2.06917400

O -3.63357300 0.97261300 -0.35675100

H -2.35718900 -1.16423700 1.83323900

H -2.63278700 -2.17785200 -0.64514000

Cl -1.20225500 0.39125000 -3.13115100

**M1-b-b3lyp**

Zero-point correction= 0.796542 (Hartree/Particle)

Thermal correction to Energy= 0.843914

Thermal correction to Enthalpy= 0.844859

Thermal correction to Gibbs Free Energy= 0.719074

Sum of electronic and zero-point Energies= -3079.695087

Sum of electronic and thermal Energies= -3079.647715

Sum of electronic and thermal Enthalpies= -3079.646770

Sum of electronic and thermal Free Energies= -3079.772555

P -0.48228600 -1.11723600 0.36951000

C 0.02946800 -1.96668600 1.94767100

C 0.88137200 -3.08393400 1.85622600

C -0.35046000 -1.52839200 3.22556700

C 1.34402900 -3.73071500 3.00298500

H 1.17726600 -3.46834400 0.88593800

C 0.10826000 -2.18058400 4.37355500

H -1.01961600 -0.68586600 3.33841100

C 0.96027400 -3.28046100 4.26831100

H 1.99891800 -4.59228900 2.90347400

H -0.20918900 -1.82586900 5.35068400

H 1.31620500 -3.78665200 5.16163500

C -0.92683800 -2.62184100 -0.63387600

C -0.39129400 -2.82851800 -1.91161100

C -1.76560900 -3.61047600 -0.08581300

C -0.70165100 -3.98625400 -2.63387200

H 0.24637500 -2.07157000 -2.35068700

C -2.07762400 -4.76139300 -0.80986500

H -2.16591200 -3.50354600 0.91640900

C -1.54692000 -4.95268500 -2.08935600

H -0.27852400 -4.12565800 -3.62546000

H -2.72689400 -5.51327000 -0.36862600

H -1.78630700 -5.85183800 -2.65123400

C -1.78402500 2.20138200 -0.28983200

H -1.64319900 1.77449500 -1.28936100

C -2.70849200 3.42492700 -0.43521600

H -2.87655000 3.95145000 0.50658900

H -2.31086700 4.14418200 -1.15045400

H -3.68432900 3.10337700 -0.81790300

P 0.05134300 2.48427100 0.15698200

C 0.16011200 3.04968900 2.00905700

C -0.89638400 4.08520400 2.44426500

H -1.90688700 3.66826000 2.40211900

H -0.70626200 4.36402800 3.49057000

H -0.87773300 5.00344300 1.85483100

C -0.03112400 1.80217900 2.88917200

H -1.02948600 1.37581600 2.77697400

H 0.70656300 1.02758100 2.66257000

H 0.09006700 2.08492700 3.94438900

C 1.57178500 3.59081600 2.31488200

H 1.77459400 4.55908600 1.85194100

H 1.66521000 3.72673900 3.40096200

H 2.35374900 2.88832600 2.00544200

C 0.66084500 3.96839000 -0.95253600

C 2.20436000 3.90277300 -1.00606000

H 2.55340500 2.94945600 -1.41031800

H 2.56676200 4.70084700 -1.66901300

H 2.67499000 4.05540000 -0.03192700

C 0.16662300 3.77840600 -2.40239300

H 0.42555300 2.79527400 -2.79936900

H -0.91338900 3.90943000 -2.51251600

H 0.64769100 4.54210100 -3.02930900

C 0.24978100 5.37846200 -0.48151600

H -0.83312700 5.51177400 -0.41667200

H 0.68726900 5.64806800 0.48254100

H 0.62166800 6.10659600 -1.21634700

Rh 1.11973200 0.22120100 -0.51012300

C -4.91067400 -1.78418700 -0.99660600

Fe -3.95190400 -0.18251700 -0.06072500

C -3.99323500 -1.17098000 -1.90268400

C -5.86731300 -0.79822500 -0.60424000

C -5.53950700 0.42300900 -1.26790400

C -4.38003000 0.19181000 -2.07011000

C -3.04093400 -0.84421300 1.67344700

C -4.00463400 0.14507600 1.99532200

C -3.63480800 1.34410900 1.32348800

C -2.43134500 1.12696200 0.57550400

C -2.04546200 -0.26062900 0.80560300

H -3.13065300 -1.64260700 -2.35284700

H -6.67727200 -0.94086500 0.10029500

H -6.05828300 1.36660300 -1.15376300

H -3.86229600 0.92636200 -2.67346100

H -3.04255700 -1.86219200 2.03547600

H -4.88726300 0.00070700 2.60564200

H -4.20446600 2.26194300 1.33762000

H -4.87195200 -2.80788300 -0.64805500

C 6.02146000 -1.66688300 -1.64923100

C 7.22924100 -1.69260300 -0.93155900

C 7.38267800 -0.95152900 0.24372000

C 6.33256800 -0.16045200 0.73957500

C 5.14533900 -0.13231800 0.02287200

H 5.90840900 -2.24569200 -2.56254300

H 8.05743500 -2.29293500 -1.29899600

H 8.32850400 -0.98280000 0.77824300

H 6.45690600 0.41272300 1.65509600

C 4.99072500 -0.87753800 -1.16053900

C 3.56393500 -0.62545900 -1.60531300

C 2.58915100 -1.27688500 -0.61347600

H 3.33291000 -0.70863700 -2.66576200

C 3.80860900 0.54687100 0.24351500

C 2.74072500 -0.49700800 0.60250400

H 3.80250200 1.48568100 0.79465400

O 3.38258700 0.77856400 -1.15845000

H 2.40143800 -2.34423400 -0.65103200

H 2.66423300 -0.89225300 1.60976900

Cl 0.01950400 0.33660900 -2.92610600

**M1-c-b3lyp**

Zero-point correction= 0.797221 (Hartree/Particle)

Thermal correction to Energy= 0.844435

Thermal correction to Enthalpy= 0.845379

Thermal correction to Gibbs Free Energy= 0.720059

Sum of electronic and zero-point Energies= -3079.684932

Sum of electronic and thermal Energies= -3079.637718

Sum of electronic and thermal Enthalpies= -3079.636774

Sum of electronic and thermal Free Energies= -3079.762095

P 0.49479400 -1.16630200 -0.01148100

C 0.17556600 -2.72997100 -0.96538700

C -0.08387700 -3.94919500 -0.31661700

C 0.09204900 -2.68987800 -2.36935500

C -0.39182000 -5.09891600 -1.04990100

H -0.04591800 -4.01679900 0.76464400

C -0.20542700 -3.84195100 -3.09832100

H 0.22856200 -1.74920900 -2.89037300

C -0.44655900 -5.05248200 -2.44311700

H -0.58557500 -6.03085800 -0.52468700

H -0.25805600 -3.78770700 -4.18282800

H -0.68130100 -5.94771600 -3.01323000

C 0.88466900 -1.86273500 1.66024500

C -0.05268900 -1.72833700 2.69382600

C 2.03730000 -2.63051600 1.89275200

C 0.16453700 -2.32571500 3.93959900

H -0.96044000 -1.16142100 2.52006000

C 2.25805200 -3.22504200 3.13614800

H 2.75618800 -2.77301500 1.09356100

C 1.32330000 -3.06977800 4.16494300

H -0.57306800 -2.20967000 4.72946400

H 3.15556500 -3.81604100 3.30002700

H 1.49515200 -3.53478600 5.13216400

C 1.84800600 1.91901600 0.37332800

H 1.86845500 1.41301600 1.34517500

C 2.80145000 3.12889500 0.46215200

H 2.90170100 3.65113600 -0.49003200

H 2.49383400 3.85759800 1.20651800

H 3.79687100 2.76915000 0.74111100

P -0.03920600 2.33652600 0.25928900

C -0.23447300 3.79673900 -1.01617600

C 0.18987800 5.18089600 -0.48186600

H 1.22347200 5.20992000 -0.13142900

H 0.11161100 5.89485300 -1.31315400

H -0.46152600 5.54866100 0.31522900

C 0.61802300 3.48190400 -2.26312900

H 1.68783100 3.59005900 -2.07479200

H 0.41750700 2.48315100 -2.65618700

H 0.35324800 4.20619500 -3.04602700

C -1.70779400 3.89093200 -1.46152000

H -2.38602800 4.09996700 -0.62930500

H -1.79776800 4.72218400 -2.17448000

H -2.02909400 2.97567900 -1.96199700

C -0.43560700 2.98329600 2.05890300

C -1.84864000 3.60731500 2.06801500

H -2.60737100 2.96995300 1.61341900

H -2.14730100 3.78411600 3.11006900

H -1.87034400 4.57455100 1.55799200

C -0.40039400 1.77484300 3.01523200

H -1.09085900 0.98887500 2.71952100

H 0.60110500 1.33758900 3.08748900

H -0.67647600 2.11639700 4.02211400

C 0.53360400 4.02422000 2.66546300

H 1.50337100 3.58687400 2.91652300

H 0.69117400 4.90380700 2.04174800

H 0.09297700 4.37165800 3.60991000

Rh -1.32831200 0.45110400 -0.33749900

C 5.29442100 -1.77383900 0.39594300

Fe 4.12534500 -0.25084700 -0.43736700

C 4.86167500 -0.89018900 1.43224700

C 6.06019700 -1.01218400 -0.53752000

C 6.09366600 0.34112500 -0.08232300

C 5.35684500 0.41260800 1.13879000

C 2.78872400 -1.03667100 -1.82428900

C 3.56496800 -0.00148000 -2.41299200

C 3.31415200 1.19948900 -1.68818100

C 2.36363600 0.92655900 -0.65257700

C 2.02484300 -0.47716800 -0.74378600

H 4.25007300 -1.15576600 2.28293900

H 6.50241900 -1.38605900 -1.45247300

H 6.57090000 1.17074800 -0.58894700

H 5.18686900 1.30209900 1.73123700

H 2.75948400 -2.06878100 -2.14436400

H 4.26231600 -0.11463500 -3.23346400

H 3.80115200 2.14651200 -1.87288800

H 5.07124400 -2.83042800 0.31980500

C -6.60135100 -0.25304100 -0.87941600

C -7.59503000 -0.78609200 -0.04151600

C -7.25771900 -1.44732100 1.14314200

C -5.91489800 -1.59611500 1.52855000

C -4.93767300 -1.07283100 0.69461700

H -6.86973100 0.25973600 -1.79966200

H -8.64051100 -0.68917400 -0.32234400

H -8.04393000 -1.85799100 1.77118500

H -5.65852500 -2.11368400 2.44973100

C -5.27644800 -0.40746300 -0.49745500

C -3.96087100 0.05346000 -1.08848400

C -3.33300600 1.14102900 -0.20216000

H -3.88826200 0.18544100 -2.16497300

C -3.42705300 -0.99862700 0.77644800

C -2.97285900 0.45052100 1.01117600

H -2.91023400 -1.78580000 1.32293800

O -3.06558200 -1.05939300 -0.66221500

H -3.66025300 2.17179100 -0.26607600

H -2.99528600 0.87775700 2.00619400

Cl -1.06656000 0.52190200 -3.02647300

**M1-d-b3lyp**

Zero-point correction= 0.796252 (Hartree/Particle)

Thermal correction to Energy= 0.844109

Thermal correction to Enthalpy= 0.845053

Thermal correction to Gibbs Free Energy= 0.716791

Sum of electronic and zero-point Energies= -3079.682212

Sum of electronic and thermal Energies= -3079.634355

Sum of electronic and thermal Enthalpies= -3079.633411

Sum of electronic and thermal Free Energies= -3079.761673

P -0.60910300 -1.07310700 0.52782900

C -0.25277600 -1.83644900 2.19501000

C 0.82361100 -2.74239700 2.25972300

C -0.96057300 -1.57088200 3.37636500

C 1.18789000 -3.34642400 3.46247900

H 1.36833000 -3.00040700 1.35596800

C -0.59372000 -2.17425300 4.58494300

H -1.81376000 -0.90304900 3.36603900

C 0.48197700 -3.05969700 4.63513500

H 2.01840200 -4.04752900 3.48127100

H -1.16114200 -1.95180000 5.48515300

H 0.76357800 -3.52967800 5.57365200

C -0.92459900 -2.65086600 -0.41176700

C -0.39675800 -2.82839600 -1.69817500

C -1.66593800 -3.69873000 0.16540700

C -0.61575500 -4.02144000 -2.39630500

H 0.16112100 -2.02380200 -2.16442700

C -1.88827300 -4.88466400 -0.53521600

H -2.06078500 -3.60110700 1.17194000

C -1.36172300 -5.04996000 -1.82052200

H -0.19966400 -4.14033200 -3.39366800

H -2.46385100 -5.68273200 -0.07288500

H -1.52895600 -5.97662700 -2.36374100

C -1.65637600 2.15025900 -0.30946700

H -1.39382800 1.70818100 -1.27769700

C -2.45995000 3.43028500 -0.59262000

H -2.62215600 4.04677200 0.29716000

H -1.96764100 4.03661700 -1.34974700

H -3.43977600 3.15838400 -0.99874800

P 0.13328200 2.38944500 0.35746200

C 0.10992700 2.80464100 2.26676800

C -0.21462400 4.27595800 2.59632800

H -1.18253700 4.58905300 2.19260200

H -0.27552800 4.36751000 3.68921700

H 0.55185300 4.97749200 2.26198400

C -0.94590300 1.95147700 2.98767800

H -1.96800100 2.22003300 2.71234400

H -0.80072900 0.88864300 2.80921700

H -0.83949800 2.11751700 4.06833500

C 1.47580200 2.43989200 2.88468700

H 2.31233000 2.97780000 2.43430700

H 1.45394100 2.70059600 3.95145000

H 1.67144300 1.36726700 2.80694200

C 0.90305700 3.94548000 -0.55314000

C 2.29607600 4.21288300 0.06924400

H 2.88847700 3.31255900 0.23128600

H 2.86049700 4.85955600 -0.61568400

H 2.22332700 4.74308300 1.02268300

C 1.06868000 3.60027500 -2.04534600

H 1.68598800 2.71890900 -2.21063500

H 0.10557900 3.41440700 -2.52897200

H 1.53439600 4.46030400 -2.54717100

C 0.12332800 5.28936400 -0.50233000

H -0.60415200 5.36978900 -1.31118400

H -0.39054000 5.49369500 0.43430600

H 0.84914300 6.09873000 -0.65694600

Rh 1.17307900 0.40013100 -0.34654400

C -4.42731700 -1.72785800 -1.74230800

Fe -3.91433600 -0.12478000 -0.51271800

C -3.52526900 -0.85935100 -2.43057400

C -5.59300600 -0.97437500 -1.40472200

C -5.40922100 0.36004200 -1.88025200

C -4.13164200 0.42842700 -2.51622900

C -3.39822100 -0.82167300 1.36475700

C -4.37446700 0.20142600 1.49089600

C -3.83760900 1.39013800 0.91726400

C -2.51353000 1.12781600 0.43199500

C -2.23352600 -0.26640000 0.72556900

H -2.53264100 -1.10850700 -2.77974900

H -6.44880800 -1.33836200 -0.84958300

H -6.10465800 1.18081000 -1.75478400

H -3.68403100 1.31009500 -2.95650400

H -3.50860000 -1.84752100 1.68803800

H -5.36881300 0.08863200 1.90490300

H -4.36896800 2.32747500 0.83443500

H -4.24412900 -2.76467300 -1.49275000

C 6.06198400 -1.60582800 0.53946000

C 7.25190400 -1.59659000 -0.20721200

C 7.26681000 -1.14047400 -1.52870500

C 6.09276900 -0.67803300 -2.14628900

C 4.91928200 -0.69433300 -1.40633800

H 6.05632700 -1.96304600 1.56627900

H 8.17165100 -1.95618000 0.24662500

H 8.19828500 -1.15008700 -2.08863500

H 6.10970600 -0.32598500 -3.17464100

C 4.90451000 -1.15375800 -0.07712300

C 3.47306000 -0.98828200 0.38836000

C 3.11112000 0.49880900 0.50323700

H 3.11738700 -1.65090400 1.17276500

C 3.49769700 -0.25902700 -1.69387200

C 3.14724800 0.97881600 -0.85340400

H 3.14468200 -0.27961800 -2.72239100

O 2.73840700 -1.24943300 -0.88148100

H 3.38013900 1.07346700 1.38097200

H 3.46845500 1.96173400 -1.17156200

Cl 0.12921600 0.37803100 -2.91296800

**TS_1-2_1A-b3lyp**

Zero-point correction= 0.793499 (Hartree/Particle)

Thermal correction to Energy= 0.840725

Thermal correction to Enthalpy= 0.841669

Thermal correction to Gibbs Free Energy= 0.716753

Sum of electronic and zero-point Energies= -3079.660502

Sum of electronic and thermal Energies= -3079.613276

Sum of electronic and thermal Enthalpies= -3079.612331

Sum of electronic and thermal Free Energies= -3079.737248

P 0.64260000 -1.16943900 -0.08137000

C 0.51833600 -2.76406200 -1.02447700

C 0.06827300 -3.92307700 -0.36592600

C 0.71785500 -2.82504200 -2.41405600

C -0.14058100 -5.11274200 -1.06766600

H -0.11888100 -3.91051000 0.70258800

C 0.52171800 -4.01921200 -3.11113800

H 1.00005100 -1.93609800 -2.96164700

C 0.09544000 -5.16828100 -2.44245000

H -0.48456300 -5.99494300 -0.53394600

H 0.69370400 -4.04315100 -4.18409700

H -0.05965800 -6.09511600 -2.98859400

C 1.17860300 -1.78156700 1.58929500

C 0.62604200 -1.25062600 2.76117900

C 2.15331300 -2.78981500 1.70448200

C 1.03333000 -1.70892900 4.01935100

H -0.12684300 -0.47611200 2.69576800

C 2.55080200 -3.25703500 2.95684300

H 2.59850400 -3.22068600 0.81369300

C 1.99233100 -2.71666200 4.12026700

H 0.59094400 -1.28203200 4.91567000

H 3.29794200 -4.04348300 3.02476700

H 2.30254100 -3.08229100 5.09550600

C 1.62460100 2.18023100 0.22390400

H 1.65070000 1.76445400 1.23844100

C 2.42175600 3.49892300 0.24549900

H 2.44691600 3.98491800 -0.73208800

H 2.01386000 4.21223900 0.95850100

H 3.45744000 3.29351400 0.53786600

P -0.27273500 2.30796100 -0.03909200

C -0.58318100 3.51341400 -1.52340600

C -0.39168300 5.01397000 -1.22669100

H 0.60633700 5.25812400 -0.85360000

H -0.53113700 5.56773000 -2.16600100

H -1.13093600 5.39964100 -0.51916900

C 0.37659100 3.11536100 -2.66516600

H 1.41994700 3.34368600 -2.43449800

H 0.28865400 2.05609100 -2.91853300

H 0.10785600 3.69475100 -3.55968900

C -2.02741300 3.30339100 -2.02803000

H -2.77653300 3.62671600 -1.30076400

H -2.16716700 3.91010400 -2.93437300

H -2.21613500 2.25825900 -2.28009700

C -0.92307900 3.14126900 1.59531000

C -2.41995800 3.46030700 1.38656100

H -2.96383600 2.63267300 0.92237700

H -2.88095100 3.66512600 2.36287400

H -2.56449100 4.35205700 0.76965800

C -0.80308800 2.10341800 2.72874900

H -1.36347500 1.19287500 2.51029000

H 0.23779300 1.82812300 2.93109500

H -1.20813600 2.53723300 3.65340500

C -0.21769000 4.41521200 2.10804900

H 0.77138000 4.19576700 2.52043700

H -0.11712000 5.20307000 1.36098800

H -0.81694900 4.82580500 2.93298500

Rh -1.35537000 0.00757900 -0.31155500

C 5.23976300 -1.23341500 0.86471000

Fe 4.08974200 0.14565500 -0.20829900

C 4.56892700 -0.36069600 1.77625400

C 6.07562400 -0.43495500 0.02711100

C 5.91563700 0.93012700 0.41551600

C 4.98691700 0.97310300 1.50016500

C 3.02845800 -0.79448200 -1.72035200

C 3.78725400 0.28219100 -2.25215700

C 3.34791700 1.47836400 -1.61761900

C 2.30007000 1.16629200 -0.69074000

C 2.08317000 -0.26636900 -0.77296500

H 3.85533700 -0.65681100 2.53214600

H 6.69101900 -0.79570400 -0.78764600

H 6.38917300 1.78420900 -0.05246000

H 4.64090600 1.86466500 2.00693700

H 3.13717200 -1.83538800 -1.98765600

H 4.59017800 0.20063400 -2.97404900

H 3.77525800 2.45756900 -1.77949200

H 5.12063200 -2.30727100 0.80605400

C -6.07555400 -0.34249800 2.01439700

C -7.34162200 -0.72849300 1.55402100

C -7.51281400 -1.17695200 0.24164700

C -6.42371600 -1.24048900 -0.64466800

C -5.16919100 -0.86565600 -0.18987100

H -5.93964900 -0.00131900 3.03769500

H -8.19708700 -0.67060800 2.22144200

H -8.50339400 -1.45984800 -0.10472200

H -6.56963400 -1.55582100 -1.67494200

C -4.99074700 -0.43330600 1.14084400

C -3.58723000 -0.16218600 1.47239300

C -2.58636100 -1.13077700 1.07465600

H -3.37503100 0.52960000 2.28410200

C -3.84935600 -0.71540100 -0.91244000

C -2.75330500 -1.58219500 -0.27266400

H -3.90429900 -0.81041900 -1.99961800

O -3.39277500 0.60010600 -0.51762900

H -1.99417800 -1.63123800 1.83379600

H -2.47600700 -2.56296500 -0.64307100

Cl -1.14367800 -0.11873600 -2.96393700

**TS_1-2_1B-b3lyp**

Zero-point correction= 0.793913 (Hartree/Particle)

Thermal correction to Energy= 0.841203

Thermal correction to Enthalpy= 0.842147

Thermal correction to Gibbs Free Energy= 0.716717

Sum of electronic and zero-point Energies= -3079.659074

Sum of electronic and thermal Energies= -3079.611784

Sum of electronic and thermal Enthalpies= -3079.610840

Sum of electronic and thermal Free Energies= -3079.736270

P 0.62298200 -1.17240900 -0.02109700

C 0.49259600 -2.80691300 -0.89588300

C 0.13622000 -3.96566400 -0.18131700

C 0.59618200 -2.89503100 -2.29485400

C -0.07187400 -5.18015400 -0.84013600

H 0.02066300 -3.93386400 0.89665400

C 0.40096000 -4.11326500 -2.94913800

H 0.79697100 -2.00754500 -2.88030900

C 0.07054600 -5.26136300 -2.22621600

H -0.34129600 -6.06121600 -0.26342900

H 0.49682100 -4.15739000 -4.03091900

H -0.08372400 -6.20709000 -2.73915800

C 1.17606900 -1.69951800 1.67065700

C 0.61908300 -1.12061500 2.81701800

C 2.16521500 -2.68755700 1.82959800

C 1.03734400 -1.51167200 4.09399400

H -0.14540100 -0.36280500 2.71328600

C 2.57338900 -3.08817900 3.10146500

H 2.61275100 -3.15394100 0.95821700

C 2.01121900 -2.49948500 4.23946100

H 0.59215200 -1.04805900 4.97046900

H 3.33235000 -3.85933100 3.20435600

H 2.33021000 -2.81272800 5.23004700

C 1.64437400 2.15733900 0.19828200

H 1.68406400 1.75393900 1.21756600

C 2.46606600 3.46143400 0.18384100

H 2.47172600 3.93706300 -0.79906600

H 2.09634100 4.18949300 0.90207600

H 3.50557300 3.23597800 0.44599000

P -0.25771900 2.31954100 -0.03913600

C -0.56248200 3.48041800 -1.56375200

C -0.32510000 4.98406700 -1.31714300

H 0.68960600 5.21243600 -0.98119300

H -0.47612500 5.51191200 -2.26952700

H -1.03167400 5.41264300 -0.60183100

C 0.36896000 3.02595100 -2.70719200

H 1.41990100 3.24088900 -2.50057800

H 0.25440800 1.96177000 -2.92715400

H 0.09603300 3.58301700 -3.61447300

C -2.01828100 3.29055700 -2.04174300

H -2.75055200 3.65717500 -1.31901900

H -2.15320700 3.86758700 -2.96784400

H -2.23279200 2.24226500 -2.25777200

C -0.88228900 3.22746300 1.56356600

C -2.35323400 3.63927700 1.32665600

H -2.93375700 2.85559400 0.83412900

H -2.82086800 3.85923200 2.29622500

H -2.42930900 4.54695900 0.72132500

C -0.83947600 2.21081100 2.72123700

H -1.45011900 1.32994400 2.51609500

H 0.18209600 1.88047500 2.93931200

H -1.22528100 2.69139900 3.63087400

C -0.10379100 4.46785400 2.05258100

H 0.86561900 4.19916200 2.48230100

H 0.05114200 5.22926400 1.28682600

H -0.68590200 4.93530000 2.85946700

Rh -1.35567700 0.03175300 -0.26491100

C 5.23787800 -1.30033100 0.75623000

Fe 4.08552300 0.08620700 -0.30272100

C 4.64801300 -0.37248900 1.66931200

C 6.05807800 -0.56364700 -0.15026700

C 5.97040400 0.81865000 0.19790600

C 5.10133000 0.93389700 1.32565100

C 2.94610100 -0.85357200 -1.75988200

C 3.69653300 0.20804600 -2.33205800

C 3.30097500 1.41532600 -1.69008400

C 2.28507200 1.12551800 -0.72093800

C 2.04829900 -0.30361900 -0.77976200

H 3.96255000 -0.61568200 2.46864400

H 6.61500200 -0.97522200 -0.98277400

H 6.45251500 1.63746900 -0.32162300

H 4.82132900 1.85382800 1.82224600

H 3.03018400 -1.89837600 -2.02081600

H 4.46863700 0.10902800 -3.08474100

H 3.73343100 2.38730900 -1.87932300

H 5.07776600 -2.37016700 0.74003900

C -6.23051500 -0.06197000 1.70162100

C -7.43016900 -0.65077100 1.26351400

C -7.46244100 -1.43387700 0.10718700

C -6.29405900 -1.65543900 -0.63471400

C -5.10014400 -1.09564100 -0.17888100

H -6.22251200 0.57924100 2.57985000

H -8.34777100 -0.47403700 1.81853400

H -8.40164500 -1.86552200 -0.22742700

H -6.31389900 -2.26701700 -1.53295300

C -5.07126900 -0.28946200 0.97762400

C -3.68531000 0.30349800 1.10674500

C -2.60297900 -0.76960400 1.27452300

H -3.62736600 1.10932700 1.84470800

C -3.77371000 -1.29660400 -0.76643900

C -2.67024000 -1.61257600 0.11242700

H -3.68396700 -1.52496600 -1.82638900

O -3.37854400 0.76421500 -0.23156100

H -2.23344600 -1.08791900 2.24350500

H -2.15149400 -2.55668200 -0.01883700

Cl -1.23692400 -0.16044700 -2.97600200

**TS_1-2_2A-b3lyp**

Zero-point correction= 0.792878 (Hartree/Particle)

Thermal correction to Energy= 0.840943

Thermal correction to Enthalpy= 0.841888

Thermal correction to Gibbs Free Energy= 0.711721

Sum of electronic and zero-point Energies= -3079.673103

Sum of electronic and thermal Energies= -3079.625038

Sum of electronic and thermal Enthalpies= -3079.624093

Sum of electronic and thermal Free Energies= -3079.754260

P 0.74421800 -1.11927300 -0.25066900

C 0.60926700 -2.53403900 -1.43736700

C 0.28446600 -3.83162500 -1.01120300

C 0.66915400 -2.27751700 -2.82015500

C 0.05651200 -4.85111900 -1.94118500

H 0.20859400 -4.06097600 0.04618200

C 0.44627100 -3.29719700 -3.74555500

H 0.89016200 -1.27750600 -3.18067300

C 0.14190900 -4.59020700 -3.30890900

H -0.18775500 -5.84983000 -1.58913600

H 0.50767100 -3.07949600 -4.80860400

H -0.03200900 -5.38429200 -4.02992400

C 1.09904300 -1.90455600 1.37854300

C 0.36494600 -1.49171900 2.49934600

C 2.06850200 -2.91174300 1.53048400

C 0.59453600 -2.06883100 3.75214100

H -0.39572700 -0.72468700 2.39140500

C 2.28935200 -3.49436700 2.77852400

H 2.64683900 -3.24582100 0.67440100

C 1.55419000 -3.07212700 3.89214800

H 0.00972000 -1.73980900 4.60623600

H 3.03534800 -4.27787100 2.88266400

H 1.72747800 -3.52927500 4.86274300

C 1.72049200 2.10073500 0.35784900

H 1.59012300 1.55136300 1.29711900

C 2.51665600 3.37780100 0.68446300

H 2.58285000 4.07103700 -0.15592500

H 2.08448800 3.91302000 1.53080000

H 3.53719600 3.09857300 0.96700200

P -0.11033000 2.28410900 -0.17846000

C -0.19730500 3.09378400 -1.92570800

C 0.77295300 4.26570500 -2.15862700

H 1.81737700 3.94219600 -2.13650500

H 0.58537300 4.68334900 -3.15775900

H 0.64178600 5.07536100 -1.43599300

C 0.12457800 1.97658700 -2.94509600

H 1.15537900 1.62185100 -2.86390900

H -0.54768100 1.11704800 -2.82032400

H -0.01314500 2.37034000 -3.96156300

C -1.64212800 3.56357500 -2.19649300

H -1.90698700 4.45502800 -1.62152500

H -1.72570800 3.82723100 -3.25965900

H -2.37503700 2.77952900 -1.98190600

C -0.92117400 3.40001200 1.16950600

C -2.45480800 3.23927300 1.11161400

H -2.76193500 2.20299000 1.26236300

H -2.89191100 3.84103100 1.91959700

H -2.88463300 3.58561400 0.17032400

C -0.46511000 2.87079600 2.54680300

H -0.67467200 1.80195100 2.66374700

H 0.59608400 3.04296500 2.74592400

H -1.03137200 3.39551900 3.32670600

C -0.58888500 4.90145300 1.05152500

H 0.48157100 5.11446400 1.07893000

H -1.00096800 5.34424800 0.14038300

H -1.04829300 5.42529600 1.90080300

Rh -1.21389400 0.10061300 -0.43171000

C 5.31123100 -1.41344800 0.84515400

Fe 4.22176300 0.09356800 -0.10966400

C 4.59169700 -0.65516200 1.81907100

C 6.19204500 -0.52350900 0.15899100

C 6.01144900 0.78392200 0.70381500

C 5.02345400 0.69975500 1.73205900

C 3.21778300 -0.67855500 -1.75782400

C 4.02323500 0.42763100 -2.14595400

C 3.57687800 1.57222700 -1.42110700

C 2.48059400 1.19103900 -0.58295600

C 2.24266000 -0.22002700 -0.80454000

H 3.83196200 -1.03621400 2.48631700

H 6.85088000 -0.78537700 -0.65953300

H 6.51457000 1.68490300 0.37561300

H 4.65478700 1.52228300 2.33086000

H 3.31378300 -1.69198100 -2.12192600

H 4.86063900 0.39487100 -2.83150300

H 4.02492100 2.55552500 -1.46674300

H 5.19827600 -2.47146600 0.64789800

C -6.11932500 -0.67979900 1.12293400

C -7.34332500 -0.80712800 0.45398600

C -7.38886500 -0.83664200 -0.94172500

C -6.21217300 -0.72961000 -1.70601300

C -4.99857300 -0.62304400 -1.04966700

H -6.07271000 -0.66169800 2.20778000

H -8.26395500 -0.87428500 1.02654500

H -8.34767800 -0.92080300 -1.44634500

H -6.26251800 -0.70978100 -2.79180300

C -4.94867900 -0.61335500 0.36272900

C -3.59507500 -0.56460200 0.90306900

C -2.59228200 -1.42790000 0.27831200

H -3.42717000 -0.18912100 1.91724700

C -3.60911300 -0.34654400 -1.58701000

C -2.61231700 -1.41772300 -1.12456800

H -3.57964400 -0.14860200 -2.66362800

O -3.16115700 0.78988300 -0.81369100

H -2.09725400 -2.16958600 0.89888900

H -2.22273600 -2.20380000 -1.76054700

Cl -3.33672600 -0.22061700 4.38136500

**TS_1-2_2B-b3lyp**

Zero-point correction= 0.793230 (Hartree/Particle)

Thermal correction to Energy= 0.840795

Thermal correction to Enthalpy= 0.841739

Thermal correction to Gibbs Free Energy= 0.714858

Sum of electronic and zero-point Energies= -3079.664089

Sum of electronic and thermal Energies= -3079.616524

Sum of electronic and thermal Enthalpies= -3079.615580

Sum of electronic and thermal Free Energies= -3079.742461

P -0.70344500 -1.11838800 0.38185200

C -0.41506100 -2.05340200 1.97171900

C 0.31116000 -3.25765600 1.89189200

C -0.83096200 -1.61695500 3.23819900

C 0.61089700 -3.99568900 3.03743900

H 0.63866400 -3.63193800 0.92660200

C -0.53369600 -2.35871700 4.38607100

H -1.40155700 -0.70320900 3.34225300

C 0.18787400 -3.54885000 4.29206900

H 1.16857200 -4.92406200 2.94562200

H -0.87575700 -2.00194300 5.35418100

H 0.41505300 -4.12521800 5.18489400

C -1.33329500 -2.54361200 -0.64048700

C -0.75511900 -2.86002200 -1.87646300

C -2.35590400 -3.37340100 -0.14435200

C -1.20236300 -3.96468300 -2.61044600

H 0.02599600 -2.22614800 -2.27580200

C -2.80469100 -4.47078000 -0.87919400

H -2.79673700 -3.18137400 0.82780800

C -2.22962600 -4.76928600 -2.11862300

H -0.74245400 -4.18971200 -3.56939100

H -3.59634900 -5.09861400 -0.47823800

H -2.57647600 -5.62682500 -2.68939300

C -1.38891600 2.32185300 -0.33191800

H -1.24433600 1.84109800 -1.30586900

C -2.09483300 3.66762100 -0.58176700

H -2.21115700 4.27090200 0.32186900

H -1.55782500 4.26661800 -1.31687800

H -3.09477700 3.48403900 -0.99215900

P 0.43933600 2.33443500 0.24524800

C 0.48160800 2.85147100 2.11738900

C -0.48536100 3.99327300 2.48988700

H -1.52896800 3.70075900 2.34374700

H -0.36169300 4.22721300 3.55704100

H -0.30311300 4.91389800 1.93228900

C 0.08669300 1.61878300 2.94980100

H -0.93819700 1.30591800 2.74775300

H 0.75270200 0.77086200 2.76506200

H 0.15075800 1.86975600 4.01785100

C 1.91210700 3.23671200 2.54534100

H 2.23367300 4.19874600 2.14074700

H 1.93738300 3.32114100 3.64030400

H 2.64792200 2.47639800 2.25968000

C 1.33508300 3.73070200 -0.77454900

C 2.86299600 3.52605700 -0.64372300

H 3.16636400 2.52513600 -0.95707100

H 3.36297400 4.25337200 -1.29908900

H 3.23176800 3.70375300 0.36851100

C 0.99824500 3.54911300 -2.26917100

H 1.21983800 2.53778600 -2.61482700

H -0.04876300 3.75809200 -2.50506000

H 1.60765600 4.25702300 -2.84817800

C 1.00957800 5.18104000 -0.36070700

H -0.05581800 5.41755900 -0.40417400

H 1.37316800 5.42209900 0.64167100

H 1.52155900 5.85938100 -1.05795400

Rh 1.22403300 -0.03877100 -0.40765400

C -5.11619100 -1.05929700 -1.21205900

Fe -3.95046800 0.36434600 -0.22523900

C -4.06529000 -0.61672500 -2.07105300

C -5.91000700 0.07720900 -0.86564600

C -5.34626900 1.22123200 -1.50891000

C -4.20562900 0.79096800 -2.25399300

C -3.26894600 -0.43333600 1.55343200

C -4.06190800 0.71218300 1.82342100

C -3.45002900 1.82447500 1.17883400

C -2.26321100 1.39312200 0.50049800

C -2.13928700 -0.03739000 0.74698900

H -3.27586000 -1.23112900 -2.48161100

H -6.76521000 0.07824100 -0.20119200

H -5.70282400 2.23967700 -1.41922100

H -3.54280200 1.42174000 -2.83195800

H -3.47447100 -1.43458800 1.90358700

H -4.99055600 0.72892000 2.38004900

H -3.84983100 2.82829700 1.16499200

H -5.26782000 -2.07267800 -0.86394600

C 6.01072900 -1.55460200 -1.86943900

C 7.27896600 -1.48376800 -1.26674100

C 7.43384800 -0.94617600 0.01353500

C 6.32273900 -0.47490500 0.72571400

C 5.06110700 -0.57174100 0.13639700

H 5.90320800 -1.94018700 -2.88036900

H 8.15207700 -1.83075000 -1.81311300

H 8.42329300 -0.88469800 0.45816200

H 6.43921200 -0.06418400 1.72547100

C 4.90876300 -1.09752800 -1.16458900

C 3.46068200 -0.93526500 -1.57457400

C 2.51470900 -1.70750100 -0.64316300

H 3.27130200 -1.10394600 -2.63912900

C 3.78660200 -0.22813200 0.77407600

C 2.69002300 -1.16448200 0.67741400

H 3.78003000 0.51655900 1.56777600

O 3.14580900 0.41946800 -1.18271500

H 2.16283500 -2.71079400 -0.85983600

H 2.27812600 -1.58639100 1.58816700

Cl 0.15989500 0.21277300 -2.81629700

**TS_1-2_3A-b3lyp**

Zero-point correction= 0.794430 (Hartree/Particle)

Thermal correction to Energy= 0.841625

Thermal correction to Enthalpy= 0.842569

Thermal correction to Gibbs Free Energy= 0.718171

Sum of electronic and zero-point Energies= -3079.661030

Sum of electronic and thermal Energies= -3079.613835

Sum of electronic and thermal Enthalpies= -3079.612891

Sum of electronic and thermal Free Energies= -3079.737288

P 0.38497400 -1.11075000 0.18085400

C 0.14329000 -2.82592100 -0.46125600

C 0.73999900 -3.94167000 0.15065800

C -0.63254800 -3.01452000 -1.61715700

C 0.56557900 -5.21979800 -0.38403400

H 1.34298600 -3.81975600 1.04473000

C -0.80111500 -4.29553300 -2.14916400

H -1.08860400 -2.15753800 -2.10021000

C -0.20687700 -5.39989400 -1.53452100

H 1.03497500 -6.07306200 0.09903400

H -1.40188800 -4.42762100 -3.04547500

H -0.34356500 -6.39563900 -1.94877900

C 0.80119500 -1.41197700 1.95736700

C -0.18063600 -2.01894200 2.76207800

C 2.00620300 -1.02203700 2.55489600

C 0.04428400 -2.23713900 4.12087100

H -1.12201000 -2.33744100 2.32342000

C 2.22960000 -1.23421100 3.91982400

H 2.77787600 -0.55927100 1.95337000

C 1.25140900 -1.84227100 4.70677800

H -0.72397500 -2.71582800 4.72255600

H 3.17302500 -0.92537400 4.36312600

H 1.42578900 -2.00964900 5.76626600

C 1.92832300 1.90838300 0.29731600

H 1.95332300 1.44567900 1.28950600

C 2.95715300 3.05566500 0.30650300

H 3.07081900 3.52368700 -0.67239100

H 2.70807600 3.83844800 1.01898800

H 3.93537100 2.64775100 0.58824200

P 0.05002900 2.38867900 0.20227800

C -0.11725600 3.83900000 -1.08497500

C 0.37196500 5.21560500 -0.59179700

H 1.41576400 5.21020700 -0.26973300

H 0.29619200 5.92167500 -1.43035500

H -0.24115500 5.61727200 0.21960200

C 0.67575900 3.46536300 -2.35566800

H 1.75573500 3.52261700 -2.20651500

H 0.41711100 2.46898900 -2.72197400

H 0.41965000 4.18938400 -3.14183200

C -1.59858900 3.97397500 -1.49458600

H -2.25054000 4.22869500 -0.65393000

H -1.68240200 4.78692400 -2.22877600

H -1.96418400 3.05794500 -1.96470000

C -0.29014100 3.06130700 2.00263100

C -1.67783700 3.73623100 2.04612400

H -2.48080100 3.08425800 1.69870600

H -1.90358900 4.00059500 3.08796100

H -1.70937600 4.66259500 1.46543700

C -0.29331700 1.85621200 2.96578900

H -0.95684500 1.05582800 2.63977200

H 0.70641200 1.43165000 3.09455600

H -0.62627200 2.19894100 3.95507000

C 0.73759700 4.06334800 2.57396700

H 1.70942900 3.59763100 2.75747300

H 0.88231700 4.94924200 1.95471500

H 0.36529000 4.40592500 3.54930300

Rh -1.38086000 0.46105400 -0.32957800

C 4.84703500 -1.76442300 0.69413600

Fe 3.97184500 -0.51625800 -0.74722800

C 5.21685600 -0.40495800 0.93653400

C 5.29982600 -2.11943500 -0.61085600

C 5.95372900 -0.98162100 -1.17543800

C 5.90249800 0.07766100 -0.21952500

C 2.37578900 -1.14179900 -1.91274800

C 3.15572300 -0.18394400 -2.61576800

C 3.13281500 1.03076000 -1.87082200

C 2.31092700 0.84939900 -0.71421600

C 1.84857100 -0.51708700 -0.73052700

H 5.01247200 0.16337300 1.83474600

H 5.14765800 -3.07358600 -1.09941500

H 6.37934400 -0.92107200 -2.16921300

H 6.28994100 1.07901800 -0.35894200

H 2.19673100 -2.16388200 -2.21549800

H 3.70787600 -0.35877000 -3.53049400

H 3.67007200 1.93199300 -2.13179600

H 4.29677100 -2.40672900 1.36827800

C -6.51609900 -0.32060000 -1.17463000

C -7.48845100 -1.11197100 -0.54695400

C -7.16761700 -1.86888500 0.58257800

C -5.86282200 -1.86080600 1.10588300

C -4.90230900 -1.07328400 0.49118400

H -6.76600800 0.27406200 -2.04956200

H -8.49747800 -1.14247900 -0.94888900

H -7.92909000 -2.48818500 1.04931800

H -5.61072400 -2.47984900 1.96367900

C -5.22962200 -0.29446300 -0.63642700

C -4.11272900 0.54829700 -1.08712200

C -3.37731200 1.29809500 -0.09767000

H -4.07410200 0.85949200 -2.12923300

C -3.41737400 -0.93631200 0.73763100

C -3.00775700 0.50994300 1.04994800

H -3.01588600 -1.67651600 1.43724900

O -2.83105800 -1.06126200 -0.58026800

H -3.35272100 2.37833000 -0.16335800

H -2.93732300 0.88628900 2.06418600

Cl -0.94780200 0.35504300 -2.96206600

**TS_1-2_3B-b3lyp**

Zero-point correction= 0.794671 (Hartree/Particle)

Thermal correction to Energy= 0.841778

Thermal correction to Enthalpy= 0.842723

Thermal correction to Gibbs Free Energy= 0.718210

Sum of electronic and zero-point Energies= -3079.657858

Sum of electronic and thermal Energies= -3079.610751

Sum of electronic and thermal Enthalpies= -3079.609806

Sum of electronic and thermal Free Energies= -3079.734319

P 0.34465300 -1.10768200 0.11804400

C 0.08098900 -2.80623700 -0.56413600

C 0.70326800 -3.93367100 -0.00040700

C -0.72772600 -2.96961600 -1.70014500

C 0.51998900 -5.19856200 -0.56239200

H 1.33050100 -3.83041800 0.87955000

C -0.90568800 -4.23764700 -2.26029700

H -1.21046900 -2.10330500 -2.13828200

C -0.28591300 -5.35341800 -1.69377500

H 1.00672300 -6.06159800 -0.11511100

H -1.53400900 -4.35077900 -3.14017900

H -0.42966100 -6.33889600 -2.12966400

C 0.64997900 -1.46188800 1.90773900

C -0.38435800 -2.08803500 2.62852600

C 1.80961000 -1.08322200 2.59582600

C -0.25310800 -2.33660300 3.99457100

H -1.29272000 -2.39693900 2.11760100

C 1.93856200 -1.32678300 3.96801100

H 2.61997300 -0.60560800 2.05916600

C 0.90989800 -1.95391200 4.67128700

H -1.05884900 -2.83121900 4.53085200

H 2.84821700 -1.02784300 4.48289300

H 1.01100500 -2.14588900 5.73611400

C 1.98528300 1.85849100 0.32997800

H 1.94396000 1.39383400 1.32066300

C 3.05815000 2.96375900 0.38718400

H 3.22624100 3.43206200 -0.58407300

H 2.81441800 3.75221400 1.09483000

H 4.00772400 2.51441900 0.70204500

P 0.13094700 2.41520800 0.15530000

C 0.07775000 3.85598600 -1.14401200

C 0.61845900 5.21163000 -0.64458600

H 1.64967000 5.15791000 -0.28888400

H 0.60446000 5.91255100 -1.49070600

H 0.00242200 5.65264300 0.14300600

C 0.89944700 3.43629000 -2.38168600

H 1.97448500 3.44336400 -2.19071800

H 0.60607500 2.45128200 -2.75195300

H 0.70904500 4.16590500 -3.18137300

C -1.38157600 4.05316700 -1.60618900

H -2.05619500 4.31796300 -0.78712400

H -1.40686600 4.87754500 -2.33218500

H -1.76122700 3.15630700 -2.10091700

C -0.25203000 3.13130300 1.92770300

C -1.57163300 3.93435100 1.87905000

H -2.36323200 3.44119200 1.31324400

H -1.93634300 4.08053500 2.90447800

H -1.42660600 4.92700800 1.44436400

C -0.40458300 1.93311900 2.89160200

H -1.12707400 1.19100600 2.55020700

H 0.54802200 1.41867000 3.04936300

H -0.73366300 2.30823300 3.87040100

C 0.81730400 4.04628700 2.56423300

H 1.72796000 3.49909800 2.82261400

H 1.08481900 4.90631300 1.94844500

H 0.40693500 4.43669900 3.50594100

Rh -1.39479500 0.53165800 -0.37444100

C 4.75052600 -1.88070200 0.90571500

Fe 3.99680800 -0.62903600 -0.60149300

C 5.15401000 -0.53074600 1.14584500

C 5.25850900 -2.27452100 -0.36756500

C 5.98085100 -1.16995600 -0.91450200

C 5.91664400 -0.09287200 0.02063000

C 2.45120400 -1.20911200 -1.85447900

C 3.29794500 -0.27774200 -2.51392400

C 3.27197700 0.93862200 -1.77182400

C 2.37991900 0.78673800 -0.66314300

C 1.87761800 -0.56493200 -0.70447200

H 4.92182100 0.05943600 2.02323800

H 5.10028500 -3.23194700 -0.84773700

H 6.45987500 -1.14114000 -1.88504500

H 6.34805300 0.89111700 -0.11369800

H 2.25723600 -2.22589500 -2.16580900

H 3.89378600 -0.47177000 -3.39682500

H 3.84902000 1.82252300 -2.00560300

H 4.14532000 -2.49094700 1.56255000

C -6.40066600 -0.70553200 -1.24304200

C -7.39994200 -1.33164400 -0.47792200

C -7.15435200 -1.71582800 0.84302100

C -5.90719100 -1.47302700 1.43357900

C -4.92533900 -0.82598100 0.68112000

H -6.58722400 -0.44264400 -2.28144800

H -8.36746100 -1.53939400 -0.92727200

H -7.93263900 -2.21371900 1.41481300

H -5.71671700 -1.76316100 2.46383300

C -5.16792800 -0.45784600 -0.65979200

C -3.87978500 0.07959300 -1.24427000

C -3.39227000 1.32861200 -0.49696800

H -3.87649800 0.18244900 -2.33241800

C -3.60702600 -0.40481100 1.16361300

C -3.15607800 0.93491200 0.85580600

H -3.16131800 -0.93590400 2.00248700

O -2.88626400 -0.87459400 -0.79976600

H -3.60635700 2.34026300 -0.82232600

H -2.96177200 1.60938000 1.67796600

Cl -0.89708700 0.45018800 -2.98692600

**TS_1-2_4A-b3lyp**

Zero-point correction= 0.793043 (Hartree/Particle)

Thermal correction to Energy= 0.840780

Thermal correction to Enthalpy= 0.841724

Thermal correction to Gibbs Free Energy= 0.715150

Sum of electronic and zero-point Energies= -3079.654598

Sum of electronic and thermal Energies= -3079.606861

Sum of electronic and thermal Enthalpies= -3079.605917

Sum of electronic and thermal Free Energies= -3079.732492

P -0.40709100 -1.05539800 0.56786000

C 0.08143000 -1.64133900 2.27076000

C 1.32136200 -2.30196000 2.37020800

C -0.67138100 -1.46902600 3.44116100

C 1.79340400 -2.76645900 3.59706600

H 1.91733200 -2.46659200 1.47671600

C -0.19389300 -1.92891700 4.67453000

H -1.63969700 -0.98328000 3.40538300

C 1.03794200 -2.57624700 4.75892900

H 2.74983700 -3.28091800 3.64476800

H -0.79597700 -1.78139000 5.56754300

H 1.40507200 -2.93557000 5.71659100

C -0.50775300 -2.71273900 -0.27254800

C -0.03731900 -2.87226500 -1.58400100

C -1.03287600 -3.83186000 0.39920300

C -0.10951000 -4.11901200 -2.21447900

H 0.37321600 -2.01856800 -2.11007200

C -1.10854200 -5.07305400 -0.23454300

H -1.37403200 -3.74398800 1.42611500

C -0.64710500 -5.21992200 -1.54630300

H 0.25953600 -4.22488700 -3.23171600

H -1.51894200 -5.92613500 0.30006900

H -0.69945200 -6.18792300 -2.03830500

C -1.96043100 1.98502100 -0.32955900

H -1.68886800 1.58238500 -1.31245700

C -2.95545300 3.13498100 -0.55691200

H -3.18794800 3.69139800 0.35732000

H -2.58034700 3.83607800 -1.29864800

H -3.89418200 2.73347100 -0.95330100

P -0.19565600 2.47408000 0.24534100

C -0.23563800 2.98398600 2.13158900

C -0.72986600 4.41731800 2.40830200

H -1.74345800 4.58803700 2.03197500

H -0.76103300 4.56198400 3.49691000

H -0.07006500 5.18716800 2.00390200

C -1.15917600 2.03836100 2.91752700

H -2.20821400 2.13619300 2.63005800

H -0.86239600 0.99589100 2.81301500

H -1.08276900 2.29078700 3.98416300

C 1.17802200 2.82379100 2.72850200

H 1.92225500 3.46514900 2.24998900

H 1.14244500 3.10124500 3.79071200

H 1.52007800 1.78637900 2.66875900

C 0.31957600 4.07132900 -0.76569300

C 1.70101900 4.52853400 -0.23760000

H 2.41934800 3.71295100 -0.14459300

H 2.11937000 5.25204100 -0.94989400

H 1.62955900 5.03547900 0.72874700

C 0.46947000 3.68412900 -2.24915900

H 1.19655600 2.88919700 -2.40998500

H -0.47751400 3.34861300 -2.68051500

H 0.79322700 4.57599900 -2.80462900

C -0.61938900 5.31104700 -0.73310300

H -1.15364100 5.46570200 0.20141800

H -0.00614100 6.20482200 -0.90860400

H -1.35574000 5.28043100 -1.53907400

Rh 1.16585800 0.55238200 -0.38619100

C -4.15617200 -2.29227000 -1.66999400

Fe -3.83115400 -0.61674900 -0.46802100

C -3.36166700 -1.33523300 -2.37264100

C -5.40096400 -1.67377500 -1.34002400

C -5.37405100 -0.33423300 -1.83643800

C -4.11274800 -0.12739400 -2.47593900

C -3.19053400 -1.20650100 1.40580100

C -4.31115700 -0.34519200 1.53762600

C -3.96995800 0.90550900 0.94652000

C -2.62692600 0.84660200 0.44333800

C -2.13012600 -0.48313600 0.75192000

H -2.34562200 -1.46983100 -2.71734500

H -6.20816100 -2.12707800 -0.77793500

H -6.15657800 0.40462900 -1.71509000

H -3.76602400 0.79450400 -2.92510700

H -3.13763300 -2.23431900 1.73667500

H -5.27182600 -0.60573200 1.96435100

H -4.64091500 1.74786500 0.85851000

H -3.85396600 -3.29744600 -1.40616000

C 6.02809300 -1.18386300 0.57316600

C 7.11870000 -1.49857500 -0.24859300

C 7.04385000 -1.30740400 -1.63085000

C 5.87305700 -0.80639000 -2.22567600

C 4.79546700 -0.48663400 -1.41452700

H 6.08757400 -1.32481400 1.64944100

H 8.02582800 -1.90195200 0.19296100

H 7.89325000 -1.56904200 -2.25641800

H 5.80936800 -0.69374900 -3.30523000

C 4.87673600 -0.65988700 -0.01636200

C 3.68021000 -0.18439700 0.68824700

C 3.14308000 1.11128000 0.32277100

H 3.44118700 -0.60109200 1.66342900

C 3.38901800 -0.04159100 -1.75090900

C 3.05174000 1.30134300 -1.08848800

H 3.12960700 -0.09075900 -2.81165200

O 2.55836500 -0.92473400 -0.96094300

H 3.11798900 1.89928200 1.06524000

H 3.14906400 2.25007900 -1.60213000

Cl 0.01781500 0.35056000 -2.83095100

**TS_1-2_4B-b3lyp**

Zero-point correction= 0.794413 (Hartree/Particle)

Thermal correction to Energy= 0.841793

Thermal correction to Enthalpy= 0.842737

Thermal correction to Gibbs Free Energy= 0.716811

Sum of electronic and zero-point Energies= -3079.650600

Sum of electronic and thermal Energies= -3079.603220

Sum of electronic and thermal Enthalpies= -3079.602276

Sum of electronic and thermal Free Energies= -3079.728202

P -0.41465300 -1.08926900 0.47642100

C 0.02520000 -1.89317900 2.10126300

C 1.05876300 -2.85047900 2.09264300

C -0.57659200 -1.58649600 3.33127700

C 1.48554700 -3.46067600 3.27222400

H 1.52488000 -3.13604100 1.15479200

C -0.15018600 -2.20034700 4.51394500

H -1.39800900 -0.88242300 3.37889300

C 0.88440600 -3.13526600 4.49153700

H 2.28224600 -4.19932900 3.23498200

H -0.63952700 -1.94828000 5.45137400

H 1.21317200 -3.61352000 5.41035700

C -0.59257700 -2.63526200 -0.54390800

C 0.04881200 -2.74834700 -1.78447900

C -1.33582500 -3.72917100 -0.06366400

C -0.06866400 -3.92220800 -2.53807800

H 0.62362100 -1.91421700 -2.16491200

C -1.45741500 -4.89506100 -0.81992100

H -1.81290800 -3.68430200 0.91002500

C -0.82394200 -4.99443600 -2.06311600

H 0.43504400 -3.99163700 -3.49902400

H -2.03890800 -5.72867200 -0.43391800

H -0.91426200 -5.90507500 -2.64998700

C -1.84869400 1.99503200 -0.39177000

H -1.51159900 1.52600600 -1.32235200

C -2.83684400 3.10921100 -0.77553000

H -3.08614200 3.78158400 0.05148700

H -2.44717900 3.70455500 -1.59878500

H -3.76772800 2.65483700 -1.13042400

P -0.11483900 2.51071700 0.27573100

C -0.24208000 3.01572200 2.15657000

C -0.90415000 4.38699500 2.40023900

H -1.90372400 4.45170400 1.95829100

H -1.02079500 4.52062700 3.48449700

H -0.30666200 5.22650100 2.03925800

C -1.06139100 1.97522000 2.93774200

H -2.11903400 1.97570500 2.67102400

H -0.66280800 0.97022000 2.80461800

H -0.98832400 2.21517600 4.00747200

C 1.16355100 3.00568600 2.79775000

H 1.88719500 3.65225600 2.30083200

H 1.07177100 3.35456500 3.83540000

H 1.56877300 1.99123100 2.82967800

C 0.44507600 4.13196900 -0.68720000

C 1.71548900 4.71015400 -0.01796700

H 2.47088200 3.96005900 0.22073300

H 2.17210600 5.42263700 -0.71744500

H 1.48954300 5.26224600 0.89750000

C 0.80445100 3.74127100 -2.13274000

H 1.63769200 3.03840600 -2.17972700

H -0.03366800 3.28162400 -2.66074500

H 1.09168400 4.65410800 -2.67375300

C -0.57291800 5.30113100 -0.78143300

H -1.24806300 5.18742200 -1.63063100

H -1.17508100 5.45714600 0.11209700

H -0.00612100 6.22520800 -0.95649300

Rh 1.21790900 0.55019900 -0.33789200

C -3.60392000 -1.50149200 -2.20095400

Fe -3.87919000 -0.51015000 -0.37709200

C -4.11078900 -0.18796700 -2.42828700

C -4.54714900 -2.20735400 -1.39449300

C -5.64104600 -1.32862700 -1.12504400

C -5.37099800 -0.07984600 -1.76360900

C -3.15812700 -1.07404500 1.48013100

C -4.22530100 -0.15150200 1.64231400

C -3.85632100 1.05951600 0.98920700

C -2.54687700 0.91431800 0.42294400

C -2.10176000 -0.43087700 0.74109600

H -3.60851600 0.59568000 -2.98049900

H -4.43858500 -3.22060800 -1.03020400

H -6.50663700 -1.55676300 -0.51543000

H -5.99799700 0.80217300 -1.72275700

H -3.13879400 -2.08824800 1.85347100

H -5.17006900 -0.34747700 2.13418900

H -4.48225300 1.93749000 0.91221300

H -2.65371900 -1.88083100 -2.54996600

C 5.87047100 -1.72033300 0.49705300

C 7.07538700 -1.77163800 -0.22521400

C 7.21358000 -1.08770600 -1.43587600

C 6.15380200 -0.32749200 -1.94874800

C 4.96956100 -0.25543900 -1.21420700

H 5.75835600 -2.28397300 1.42011100

H 7.90144300 -2.36879300 0.15194400

H 8.14625700 -1.15253600 -1.98944600

H 6.26008000 0.21064600 -2.88715500

C 4.82446700 -0.96089800 -0.00278100

C 3.39722800 -0.80159200 0.46655500

C 3.03455500 0.66029600 0.74771500

H 3.11458500 -1.48553000 1.26955800

C 3.79918400 0.56797000 -1.53610300

C 3.21490000 1.37349600 -0.48999400

H 3.60836300 0.82444100 -2.57690800

O 2.60701300 -1.01745100 -0.73357600

H 3.11598600 1.10058900 1.73467600

H 3.19768100 2.44725800 -0.61447300

Cl 0.13764100 0.48338100 -2.85006500

**M2-2a-b3lyp**

Zero-point correction= 0.796190 (Hartree/Particle)

Thermal correction to Energy= 0.844057

Thermal correction to Enthalpy= 0.845002

Thermal correction to Gibbs Free Energy= 0.718051

Sum of electronic and zero-point Energies= -3079.700450

Sum of electronic and thermal Energies= -3079.652583

Sum of electronic and thermal Enthalpies= -3079.651639

Sum of electronic and thermal Free Energies= -3079.778589

P -0.77254100 -1.19641300 0.05220800

C -0.53535000 -2.45689500 1.40605000

C -0.46296000 -3.83322400 1.13213500

C -0.33785600 -2.03156400 2.73134800

C -0.22452000 -4.75539500 2.15507700

H -0.59053100 -4.19707100 0.11836900

C -0.10137200 -2.95379000 3.75274800

H -0.37715700 -0.97552900 2.97555200

C -0.04652000 -4.32051400 3.46920700

H -0.18003300 -5.81522700 1.91844700

H 0.03871300 -2.60080900 4.77121200

H 0.13536200 -5.03817400 4.26463800

C -1.67458700 -2.19550800 -1.22253300

C -1.25604700 -2.16498800 -2.55832800

C -2.77643500 -2.99797300 -0.87015000

C -1.92911200 -2.92031800 -3.52703900

H -0.40579700 -1.55141400 -2.84440100

C -3.43751000 -3.75601000 -1.83508100

H -3.11774500 -3.03659000 0.15934400

C -3.01560400 -3.71776300 -3.16934300

H -1.59312200 -2.88460500 -4.56014200

H -4.28230300 -4.37666300 -1.54728400

H -3.53241900 -4.30925200 -3.92073900

C -1.34231500 2.20623800 -0.20758200

H -1.37955200 1.72146200 -1.18946400

C -2.06587400 3.56460700 -0.34081400

H -2.01790700 4.16660600 0.56665100

H -1.68187500 4.16250600 -1.16360000

H -3.12123100 3.36758900 -0.55193400

P 0.55876100 2.21695900 0.06954500

C 0.92427000 2.89507700 1.84199000

C 0.02543300 4.07727500 2.26333000

H -1.01643300 3.77153000 2.38900100

H 0.37739400 4.43352500 3.24107600

H 0.06441900 4.92469000 1.57794300

C 0.67239200 1.75823300 2.85258200

H -0.34694700 1.36768600 2.79027300

H 1.37038800 0.93257900 2.71999700

H 0.81274000 2.15777900 3.86597100

C 2.40188200 3.32684000 1.94686000

H 2.58521800 4.29443300 1.47312500

H 2.66038600 3.43186100 3.00902900

H 3.07684800 2.59820800 1.49706100

C 1.19093000 3.49613900 -1.27954000

C 2.69427900 3.31187800 -1.56771500

H 2.91272000 2.32151800 -1.96443100

H 2.97597300 4.06266700 -2.31937100

H 3.31748300 3.46950400 -0.68621100

C 0.44700600 3.23550900 -2.60905400

H 0.55750900 2.19895200 -2.93505400

H -0.61406400 3.49063900 -2.58095200

H 0.90588000 3.87277400 -3.37700700

C 0.98100000 4.97213500 -0.86724500

H -0.04975500 5.22739100 -0.61967800

H 1.62000700 5.27014400 -0.03300300

H 1.26774500 5.59366600 -1.72612200

Rh 1.35367900 -0.00398000 -0.39808900

C -5.49194000 -0.69036000 -0.45963900

Fe -3.99291200 0.51297700 0.36627700

C -4.64449200 -0.19712900 -1.49725500

C -6.07011600 0.43281900 0.20690100

C -5.57702200 1.61859600 -0.41744400

C -4.69694100 1.22750700 -1.47222700

C -2.98181700 -0.53566100 1.84689300

C -3.58761900 0.62484500 2.40036400

C -3.04581100 1.76166700 1.73232700

C -2.09079500 1.31717600 0.76034700

C -2.03609400 -0.12346700 0.84242900

H -4.04261000 -0.79709600 -2.16504400

H -6.73800400 0.39405200 1.05842000

H -5.80716200 2.63446400 -0.12171900

H -4.14435900 1.89337500 -2.12263800

H -3.18159000 -1.55624100 2.14213200

H -4.35756900 0.64134700 3.16153100

H -3.34401500 2.78643000 1.90408400

H -5.65159300 -1.73068000 -0.20834200

C 6.00872000 -2.27901200 -0.94048800

C 7.23819100 -1.64120100 -0.79835800

C 7.33040800 -0.46773400 -0.03976100

C 6.18856100 0.07658600 0.55611700

C 4.94920900 -0.54855900 0.41683900

H 5.92967900 -3.19987500 -1.51374300

H 8.12492500 -2.05800800 -1.26810000

H 8.29171400 0.02424200 0.08434100

H 6.26190400 0.99815300 1.12966200

C 4.85749300 -1.74904500 -0.32770100

C 3.59684100 -2.45446400 -0.34817600

C 2.53671000 -2.08707800 0.44197000

H 3.54279000 -3.38035600 -0.91742100

C 3.67118200 0.11017800 0.88869400

C 2.50583600 -0.83334100 1.17610300

H 3.88072800 0.76486900 1.74834400

O 3.20548500 0.85781200 -0.23203700

H 1.72783000 -2.79506300 0.56102400

H 2.06618800 -0.85264800 2.16909800

Cl 1.45778700 -0.05659900 -3.13695200

**M3-b3lyp**

Zero-point correction= 0.795940 (Hartree/Particle)

Thermal correction to Energy= 0.843604

Thermal correction to Enthalpy= 0.844548

Thermal correction to Gibbs Free Energy= 0.718193

Sum of electronic and zero-point Energies= -3079.718530

Sum of electronic and thermal Energies= -3079.670866

Sum of electronic and thermal Enthalpies= -3079.669922

Sum of electronic and thermal Free Energies= -3079.796277

P -0.67598100 -1.15127700 0.16249400

C -0.21836000 -2.28142800 1.56594000

C 0.27413400 -3.56225500 1.25686400

C -0.31741000 -1.91324000 2.91578300

C 0.65646500 -4.44508700 2.26789200

H 0.35323600 -3.88310400 0.22325100

C 0.06552600 -2.79823600 3.92690200

H -0.70138600 -0.93991000 3.19164000

C 0.55488600 -4.06571800 3.60833300

H 1.02932900 -5.43126200 2.00412500

H -0.02390600 -2.49168100 4.96579100

H 0.84980300 -4.75342800 4.39641900

C -1.55321600 -2.36863300 -0.93455700

C -1.15241200 -2.59123600 -2.25801800

C -2.59339600 -3.15492900 -0.40492500

C -1.78935900 -3.55962800 -3.04172200

H -0.35708900 -1.99439700 -2.68315200

C -3.22993600 -4.11699800 -1.18878700

H -2.90424100 -3.03748600 0.62659900

C -2.83115500 -4.32108100 -2.51318700

H -1.46494400 -3.71342500 -4.06756900

H -4.03047800 -4.71384600 -0.75957700

H -3.32451800 -5.07379900 -3.12236800

C -1.25200900 2.46711400 0.00932400

H -1.34734100 2.16375000 -1.03891700

C -1.87080300 3.87132600 0.14392400

H -1.82406200 4.26457300 1.16169800

H -1.37334300 4.58904800 -0.50829100

H -2.92523600 3.84118500 -0.15570400

P 0.63633500 2.30373300 0.19121200

C 1.13579300 2.60344300 2.03201600

C 0.48086500 3.82780000 2.70209800

H -0.60186400 3.70185500 2.79704100

H 0.88348900 3.92351400 3.72051900

H 0.67453500 4.77000500 2.18686200

C 0.71997800 1.35613500 2.83636600

H -0.36423900 1.22428000 2.85129100

H 1.19194700 0.45328900 2.44374900

H 1.04685300 1.48677500 3.87784600

C 2.67295700 2.70486100 2.13650900

H 3.07460400 3.62614000 1.70826500

H 2.94795000 2.69857800 3.20023100

H 3.16492300 1.84865800 1.66464100

C 1.44392100 3.69510100 -0.90736300

C 2.87952400 3.23087700 -1.24408200

H 2.87853700 2.28395300 -1.79287400

H 3.35485600 3.98647600 -1.88442800

H 3.50947400 3.10496500 -0.36057600

C 0.69390900 3.83528200 -2.24923200

H 0.60704600 2.88288500 -2.77377500

H -0.30987000 4.25472900 -2.13923800

H 1.26247900 4.52727700 -2.88575200

C 1.51869900 5.09222500 -0.25733000

H 0.53639600 5.47968700 0.02626700

H 2.16681200 5.12049900 0.62155600

H 1.94427600 5.79048700 -0.99136300

Rh 1.10396600 -0.03532500 -0.70285600

C -5.27700100 -0.63209200 -0.61992300

Fe -3.85623700 0.58394200 0.31579900

C -4.36780000 -0.11612600 -1.59200500

C -5.91493400 0.47559400 0.01815500

C -5.39710500 1.67502400 -0.55871900

C -4.43974500 1.30828700 -1.55401300

C -2.92104900 -0.47676100 1.82602700

C -3.58245700 0.64698100 2.38124100

C -3.02834700 1.81307200 1.78464500

C -2.01016000 1.44114400 0.84724800

C -1.93078100 -0.01736400 0.88006200

H -3.71118100 -0.69952100 -2.22308500

H -6.63592400 0.41778500 0.82410500

H -5.66019100 2.68410700 -0.26729600

H -3.84773800 1.98807300 -2.15316800

H -3.11488600 -1.50708100 2.08642400

H -4.39402300 0.61978800 3.09739000

H -3.36316400 2.82218000 1.97522600

H -5.43612700 -1.67755100 -0.39036000

C 6.22064100 -0.26217300 -1.16599000

C 7.09130000 -0.56216700 -0.11965000

C 6.63983700 -1.31206700 0.97207300

C 5.30791200 -1.73555800 1.02010400

C 4.42006000 -1.42704400 -0.01212700

H 6.57569800 0.29946900 -2.02765600

H 8.12335300 -0.22296300 -0.15941500

H 7.31948200 -1.56017100 1.78331000

H 4.94934500 -2.30354300 1.87643600

C 4.88293100 -0.69868800 -1.13563700

C 3.99584000 -0.50713700 -2.27092400

C 2.78850100 -1.14983500 -2.35520600

H 4.37561600 0.04870300 -3.12566500

C 2.94005900 -1.72569800 0.11551400

C 2.18462500 -1.81255500 -1.22204800

H 2.78766700 -2.62743700 0.72375100

O 2.34588000 -0.59488800 0.76643000

H 2.24635600 -1.13812600 -3.29692100

H 1.59872600 -2.70724500 -1.41977700

Cl -0.15713300 0.52173300 -2.80448500

**TS_3-4_a1-b3lyp**

Zero-point correction= 0.968885 (Hartree/Particle)

Thermal correction to Energy= 1.026250

Thermal correction to Enthalpy= 1.027195

Thermal correction to Gibbs Free Energy= 0.877682

Sum of electronic and zero-point Energies= -3427.694716

Sum of electronic and thermal Energies= -3427.637351

Sum of electronic and thermal Enthalpies= -3427.636406

Sum of electronic and thermal Free Energies= -3427.785919

P -1.33006900 -1.19034600 0.08943900

C -0.87844000 -2.29920700 1.51423000

C -0.43713400 -3.61253000 1.27613600

C -0.84267200 -1.82065200 2.83430400

C -0.00064300 -4.42837300 2.32482400

H -0.44223000 -4.01447000 0.26772700

C -0.41298200 -2.63627000 3.88286800

H -1.15539200 -0.80637100 3.05030600

C 0.00949200 -3.94499800 3.63449900

H 0.32163500 -5.44515000 2.11344900

H -0.40650300 -2.24435000 4.89699800

H 0.34118500 -4.57956500 4.45231600

C -2.11742200 -2.43994000 -1.04544300

C -1.78148700 -2.45249800 -2.40566800

C -3.03834100 -3.39342200 -0.57096500

C -2.35877400 -3.38702600 -3.27490000

H -1.07181100 -1.72497800 -2.78670500

C -3.60767200 -4.32775900 -1.43562000

H -3.30641600 -3.41699000 0.48050100

C -3.27098700 -4.32592500 -2.79419100

H -2.08703300 -3.37801000 -4.32762900

H -4.31280700 -5.05969700 -1.04923500

H -3.71532400 -5.05470500 -3.46745600

C -2.39763300 2.16492200 -0.38307800

H -2.34625800 1.66709400 -1.35810400

C -3.29954200 3.40595000 -0.54197300

H -3.39082300 3.98409200 0.37926100

H -2.93671600 4.08150400 -1.31424800

H -4.30660100 3.08889500 -0.83591200

P -0.52008700 2.44934100 -0.06928500

C -0.33598500 3.16168100 1.72754200

C -1.28643500 4.31236000 2.11789400

H -2.32823900 3.98144100 2.14978900

H -1.02660900 4.64177000 3.13446000

H -1.22143500 5.18785400 1.47054100

C -0.58725000 2.01046300 2.72639400

H -1.62134300 1.65907700 2.69522600

H 0.08995500 1.17398400 2.54316700

H -0.40515500 2.39182000 3.74265200

C 1.12574100 3.61501000 1.92099800

H 1.37636400 4.50847300 1.34280400

H 1.28139800 3.86186800 2.98074600

H 1.82959600 2.81937700 1.66076700

C -0.01044900 3.84580700 -1.34034900

C 1.51553400 3.72811100 -1.54900500

H 1.77259200 2.76988900 -2.00500200

H 1.84299500 4.52859500 -2.22798400

H 2.08287000 3.82964900 -0.62075800

C -0.66430300 3.58202200 -2.71562000

H -0.51244300 2.55367400 -3.04747400

H -1.73365800 3.80404300 -2.73650200

H -0.18824600 4.24512000 -3.45213400

C -0.32428600 5.29923000 -0.93037400

H -1.38486300 5.47406100 -0.73305300

H 0.24696000 5.62510200 -0.05713400

H -0.03844800 5.95893100 -1.76214100

Rh 0.70382100 0.19388600 -0.51608400

C -6.08823400 -1.39288900 -0.48311600

Fe -4.78063100 0.06903200 0.23730100

C -5.30282700 -0.88247500 -1.56053400

C -6.82156300 -0.30429800 0.08108300

C -6.48516200 0.87794900 -0.64607500

C -5.54544200 0.51926100 -1.66050400

C -3.65079000 -0.71287200 1.79715800

C -4.44195800 0.37164500 2.26140300

C -4.06468200 1.52884900 1.52094600

C -3.02401400 1.18165900 0.59520300

C -2.75069100 -0.23247000 0.78167200

H -4.62143400 -1.45156200 -2.17722800

H -7.48730900 -0.35812000 0.93347000

H -6.85250000 1.87564400 -0.44098800

H -5.08241000 1.19424300 -2.36909200

H -3.69869800 -1.73060700 2.15929500

H -5.22422400 0.31956500 3.00863700

H -4.52367100 2.50235600 1.61826300

H -6.10783000 -2.41895700 -0.14017800

C 4.55577300 2.01841400 -0.20981700

C 5.37147300 2.50632800 0.81593700

C 5.32929200 1.92663500 2.08825900

C 4.45283500 0.86121200 2.33244200

C 3.63019500 0.37649700 1.31720100

H 4.60206900 2.46595700 -1.20050800

H 6.04501500 3.33725100 0.61885000

H 5.96650400 2.30376300 2.88421900

H 4.40180500 0.41245100 3.32314000

C 3.67856400 0.94621000 0.02331600

C 2.81925400 0.36368300 -1.01847800

C 2.40659100 -1.02647400 -0.91586600

H 2.94317800 0.76757100 -2.01918800

C 2.55329200 -0.66568900 1.52337000

C 2.55510900 -1.67649300 0.37406500

H 2.68143000 -1.17416800 2.48961800

O 1.28383700 -0.06417400 1.46062200

H 2.29741100 -1.63744000 -1.80742600

H 1.94396900 -2.55621300 0.55213500

Cl 0.31207100 0.24923900 -3.00878000

C 4.04234400 -3.86394100 0.04176400

H 3.70455400 -3.89952000 -0.99847600

H 5.04924500 -4.27728900 0.12700300

H 3.35841800 -4.42082400 0.68321500

O 4.07960800 -2.49878100 0.52711100

H 4.81143000 -2.01812700 0.01389700

C 6.33665300 -1.09764900 -2.06372200

O 6.19891900 -1.69964200 -0.74825800

C 7.35262500 -1.38412400 0.07567800

C 8.40615500 -0.86078900 -0.89738800

C 7.54258600 -0.16444100 -1.96102500

H 6.50320100 -1.90449600 -2.78821200

H 5.40243000 -0.58687100 -2.31064400

H 7.06575100 -0.62459800 0.81244000

H 7.64934600 -2.29653300 0.60188700

H 9.11778200 -0.18751100 -0.41093400

H 8.96528100 -1.69291500 -1.34078400

H 7.22816400 0.82385700 -1.60800200

H 8.05232700 -0.04402900 -2.92134800

**M4-a1-b3lyp**

Zero-point correction= 0.969184 (Hartree/Particle)

Thermal correction to Energy= 1.026890

Thermal correction to Enthalpy= 1.027835

Thermal correction to Gibbs Free Energy= 0.877788

Sum of electronic and zero-point Energies= -3427.694885

Sum of electronic and thermal Energies= -3427.637178

Sum of electronic and thermal Enthalpies= -3427.636234

Sum of electronic and thermal Free Energies= -3427.786281

P 1.31024600 -1.16925700 -0.19120400

C 0.90947400 -2.17764900 -1.70627900

C 0.36322900 -3.46540000 -1.55644000

C 1.03148400 -1.66444400 -3.00706200

C -0.03268100 -4.21684900 -2.66637100

H 0.25552200 -3.89902200 -0.56687900

C 0.64068400 -2.41592500 -4.11772400

H 1.44003900 -0.67381900 -3.16347400

C 0.10653000 -3.69612600 -3.95456800

H -0.44040300 -5.21433600 -2.52036300

H 0.75491800 -1.99545100 -5.11390200

H -0.19399800 -4.28104500 -4.82004100

C 1.99489800 -2.53142900 0.88724700

C 1.55150800 -2.66583700 2.20963200

C 2.92587500 -3.46544900 0.39433400

C 2.03662400 -3.69590400 3.02569700

H 0.83908100 -1.95108300 2.60772600

C 3.40688400 -4.49249300 1.20603700

H 3.27067600 -3.40362100 -0.63265600

C 2.96461300 -4.61033200 2.52850800

H 1.68328400 -3.77794700 4.05072600

H 4.12346200 -5.20515600 0.80482700

H 3.33936200 -5.41175000 3.16031000

C 2.37332800 2.17654200 0.44912000

H 2.24224900 1.66599400 1.41042200

C 3.27431000 3.40394100 0.69309000

H 3.41743100 4.01104000 -0.20307500

H 2.87458800 4.05439500 1.46890800

H 4.26270800 3.07039400 1.02996400

P 0.52441200 2.47902400 0.00529000

C 0.46593600 3.13930000 -1.81958400

C 1.49531800 4.22691600 -2.18714700

H 2.51945300 3.84658100 -2.13508400

H 1.32098500 4.53083100 -3.22981800

H 1.42923400 5.12743100 -1.57447100

C 0.70832200 1.94044100 -2.76010200

H 1.72062600 1.54156600 -2.66108000

H -0.01814300 1.14523100 -2.57856200

H 0.59749800 2.28592200 -3.79930800

C -0.95713400 3.65380200 -2.12039000

H -1.19935000 4.58175500 -1.59587300

H -1.03342400 3.86325900 -3.19672900

H -1.71518300 2.90341400 -1.87644500

C -0.05658800 3.93119900 1.18164700

C -1.59872400 3.87722200 1.23876500

H -1.94271800 2.91830500 1.63111600

H -1.95717900 4.66983000 1.91125800

H -2.07200700 4.03233300 0.26712400

C 0.43540300 3.67676000 2.62382100

H 0.17239700 2.67590100 2.97019300

H 1.51291700 3.80862200 2.74908100

H -0.05249900 4.40612600 3.28614900

C 0.35747000 5.35929900 0.77222200

H 1.43919300 5.48777100 0.68541900

H -0.10343900 5.67905000 -0.16591700

H 0.01102700 6.05499000 1.55011100

Rh -0.68275500 0.20165900 0.46814500

C 5.96711800 -1.45780200 0.79098100

Fe 4.75796600 0.04742900 -0.01090500

C 5.07027500 -0.97574300 1.79167900

C 6.76849800 -0.35935000 0.35208900

C 6.36438900 0.80064200 1.08078700

C 5.31394100 0.41850800 1.97042700

C 3.77084100 -0.67578300 -1.68671900

C 4.61228300 0.40798600 -2.05188800

C 4.18303700 1.55210500 -1.32022200

C 3.06166700 1.19934500 -0.49649800

C 2.79203100 -0.20968900 -0.73860800

H 4.31157200 -1.55540200 2.29893000

H 7.52553000 -0.39183100 -0.42174900

H 6.76353500 1.79939600 0.95607100

H 4.77788200 1.07402900 2.64481500

H 3.84062200 -1.68486000 -2.06857400

H 5.45665400 0.36394300 -2.72867200

H 4.65942400 2.52142800 -1.35506000

H 6.01339200 -2.47083400 0.41331200

C -4.57280800 1.98198000 0.40640400

C -5.43065400 2.50548700 -0.56559800

C -5.43086600 1.98424500 -1.86387300

C -4.55578200 0.93879200 -2.18626600

C -3.69101800 0.41799700 -1.22459600

H -4.58767900 2.38504100 1.41692400

H -6.10409700 3.31941100 -0.30652600

H -6.10034200 2.39007200 -2.61814900

H -4.53860700 0.53327600 -3.19668400

C -3.69145000 0.93225300 0.09395000

C -2.78110600 0.32239800 1.07388000

C -2.33164300 -1.04804700 0.89731600

H -2.86248200 0.69027800 2.09349200

C -2.62282600 -0.61329500 -1.51592000

C -2.59041200 -1.67864700 -0.41059800

H -2.79505800 -1.08158400 -2.49675900

O -1.34943800 -0.02216100 -1.48406500

H -2.19907400 -1.69363500 1.76247600

H -1.93082400 -2.50589600 -0.66132200

Cl -0.12264200 0.25205600 2.92969700

C -3.94274400 -3.84230700 0.00597000

H -3.56864700 -3.87875100 1.03243100

H -4.95373400 -4.24704000 -0.05088400

H -3.28118900 -4.38645800 -0.66788600

O -4.00618500 -2.46517900 -0.46531700

H -4.75682700 -1.99502100 0.06019400

C -6.23084000 -1.13735800 2.12177400

O -6.09529100 -1.72943100 0.79826100

C -7.26371100 -1.42927500 -0.01537000

C -8.31936000 -0.94990600 0.97635400

C -7.46481000 -0.24029400 2.03867700

H -6.36312100 -1.95446900 2.84092600

H -5.30826300 -0.60095800 2.35568200

H -6.99896200 -0.65124800 -0.74012400

H -7.53898600 -2.34140200 -0.55289000

H -9.05542700 -0.29181200 0.50591600

H -8.84834100 -1.80404000 1.41475300

H -7.18268400 0.76002400 1.69301800

H -7.96822300 -0.14521800 3.00502900

**M5-b3lyp**

Zero-point correction= 0.970165 (Hartree/Particle)

Thermal correction to Energy= 1.028055

Thermal correction to Enthalpy= 1.028999

Thermal correction to Gibbs Free Energy= 0.878773

Sum of electronic and zero-point Energies= -3427.732328

Sum of electronic and thermal Energies= -3427.674438

Sum of electronic and thermal Enthalpies= -3427.673494

Sum of electronic and thermal Free Energies= -3427.823720

P -1.18104200 -1.11718600 0.11754400

C -0.54134500 -2.22498500 1.47544300

C -0.01647600 -3.47862000 1.10718500

C -0.49130400 -1.86624400 2.83117700

C 0.52861400 -4.34204000 2.05785900

H -0.05068000 -3.79619100 0.06958500

C 0.05093300 -2.73284300 3.78621000

H -0.89076800 -0.91531000 3.15997500

C 0.56334300 -3.97387600 3.40611600

H 0.91636600 -5.30817100 1.74441900

H 0.06333600 -2.43339300 4.83135200

H 0.97862800 -4.64921000 4.14963700

C -2.20988200 -2.38428100 -0.79017800

C -2.04877600 -2.55638600 -2.17178400

C -3.10954200 -3.22749900 -0.11177800

C -2.78105300 -3.52905900 -2.86310400

H -1.36402600 -1.91426900 -2.71170400

C -3.84045900 -4.19570200 -0.80028400

H -3.23514200 -3.14431300 0.96239800

C -3.68056400 -4.34831600 -2.18164300

H -2.64222200 -3.64165600 -3.93546900

H -4.52949100 -4.83724000 -0.25639400

H -4.24829400 -5.10516000 -2.71686700

C -1.86928500 2.40175700 -0.08126500

H -1.99702500 1.98221200 -1.08402400

C -2.61028900 3.75287200 -0.05281700

H -2.48386900 4.29751000 0.88583100

H -2.28244900 4.40622500 -0.86058900

H -3.68351600 3.58090900 -0.19681000

P 0.05503500 2.41851600 -0.00655400

C 0.54735400 2.79158000 1.83172900

C -0.27710000 3.88760600 2.53621600

H -1.33045400 3.60779900 2.62248500

H 0.10686300 4.01703000 3.55875900

H -0.21763600 4.85925500 2.04221900

C 0.35390700 1.48777400 2.62967600

H -0.70010200 1.21977700 2.71274000

H 0.89037800 0.64617400 2.18600100

H 0.74059000 1.62836800 3.64901800

C 2.04238700 3.15841500 1.91010100

H 2.25360700 4.15324000 1.51097100

H 2.34672300 3.16202800 2.96549400

H 2.67380300 2.43327800 1.38814000

C 0.63714100 3.93742300 -1.09009700

C 2.13894300 3.73618100 -1.39094600

H 2.32391600 2.76212000 -1.84873100

H 2.46464800 4.51052400 -2.09946300

H 2.77203100 3.81944800 -0.50542000

C -0.07669400 3.90435600 -2.45934600

H 0.07550600 2.95470700 -2.97478800

H -1.15397300 4.07694800 -2.39756500

H 0.34323900 4.70490700 -3.08451600

C 0.45093200 5.33786700 -0.47053300

H -0.58789400 5.56520000 -0.21979700

H 1.05907100 5.48506700 0.42555000

H 0.77908100 6.08810700 -1.20418700

Rh 0.64919400 0.05323800 -1.14214900

C -5.88440100 -0.78589800 -0.02785700

Fe -4.38750800 0.50715900 0.64165200

C -5.09777700 -0.34096500 -1.13314900

C -6.46489900 0.36383800 0.59062900

C -6.03545700 1.51812900 -0.13261400

C -5.18911200 1.08147500 -1.19788200

C -3.24440600 -0.45641000 2.07157900

C -3.86512000 0.68233400 2.64819900

C -3.41917000 1.82667300 1.92844600

C -2.50510300 1.42200700 0.89944800

C -2.38723900 -0.02871500 0.99403800

H -4.50174100 -0.96674600 -1.78280800

H -7.09270300 0.36384300 1.47312400

H -6.28200500 2.54497000 0.10678400

H -4.68217700 1.71652500 -1.91310400

H -3.38220900 -1.47760400 2.39744700

H -4.58220800 0.67859700 3.45990600

H -3.75438900 2.83831800 2.10703300

H -5.99672300 -1.81087700 0.30043500

C 4.42671100 1.23584400 -2.85273000

C 5.75196300 1.40482800 -2.44841100

C 6.28601500 0.61868900 -1.42207700

C 5.47576100 -0.33365900 -0.79426100

C 4.14778700 -0.49917500 -1.18627000

H 4.02818300 1.83172300 -3.67071300

H 6.37514700 2.14448100 -2.94544100

H 7.32201600 0.74185000 -1.11783700

H 5.88144800 -0.95745900 -0.00052700

C 3.60208100 0.28002900 -2.23295900

C 2.22422200 0.01518900 -2.66612400

C 1.62811200 -1.28008000 -2.41908700

H 1.89136900 0.57104600 -3.53871700

C 3.21185600 -1.47737200 -0.53832700

C 2.42271500 -2.27211500 -1.59994800

H 3.73155800 -2.13207100 0.16677200

O 2.17015900 -0.76748600 0.20935300

H 0.99480800 -1.71901800 -3.18733900

H 1.75176900 -2.97236000 -1.08374600

Cl -0.89485600 0.57401200 -2.95223000

C 3.82479000 -4.18785400 -1.88424300

H 3.03230500 -4.84221700 -1.48767100

H 4.35497300 -4.72161600 -2.67871400

H 4.53774800 -3.97453000 -1.07399500

O 3.28838800 -3.01020600 -2.46429900

H 2.61957900 -0.23787100 0.91234000

C 3.53576100 -1.02460600 3.37756200

O 3.64495000 0.01508900 2.38855900

C 4.82720600 0.80458400 2.66486900

C 5.64167900 0.02510900 3.71887000

C 4.97936300 -1.36493100 3.73271800

H 2.98559700 -0.65120600 4.25405100

H 2.97000500 -1.84690000 2.93419100

H 5.36089500 0.93349700 1.71932200

H 4.52463300 1.79230300 3.02986000

H 6.70595200 -0.01434200 3.46889700

H 5.54619000 0.49988600 4.70135600

H 5.41228000 -2.01187900 2.96087000

H 5.06890600 -1.87302800 4.69772600

**Product-b3lyp**

Zero-point correction= 0.207320 (Hartree/Particle)

Thermal correction to Energy= 0.218643

Thermal correction to Enthalpy= 0.219587

Thermal correction to Gibbs Free Energy= 0.170122

Sum of electronic and zero-point Energies= -576.608969

Sum of electronic and thermal Energies= -576.597645

Sum of electronic and thermal Enthalpies= -576.596701

Sum of electronic and thermal Free Energies= -576.646166

C 2.40591100 1.15623000 -0.28059500

C 3.30605400 0.12581500 -0.55875100

C 2.88006800 -1.20304600 -0.51722600
[truncated: 51,957 more chars]
